# Total Syntheses of Hosieines A–C

**DOI:** 10.1002/advs.202308164

**Published:** 2024-02-07

**Authors:** Jiayang Zhang, Xu Yan, Qing‐Bao Zhang, Fang Wang, Bin Yang, Yang Yang

**Affiliations:** ^1^ Hubei Key Laboratory of Natural Medicinal Chemistry and Resource Evaluation School of Pharmacy Huazhong University of Science and Technology 13 Hangkong Road Wuhan 430030 China; ^2^ Shandong Peninsula Engineering Research Center of Comprehensive Brine Utilization Weifang University of Science and Technology Shouguang 262700 China; ^3^ Baylor College of Medicine Houston TX 77030 USA

**Keywords:** [3+2] reaction, alkaloids, natural products, one pot, total synthesis

## Abstract

The collective total syntheses of (±)‐hosieines A–C with a cage‐like tetracyclic framework have been realized, which includes the first syntheses of hosieines B‐C. The key strategy of the synthesis employs a one‐pot domino reaction that involves Cu‐catalyzed [3+2] cycloaddition, 1,6‐enone formation, and 1,6‐aza‐Michael addition forming the 5/6/6‐aza‐tricyclic skeleton. Other salient synthetic tactics comprise a challenging double bond migration and a 1,4‐aza‐Michael addition reaction to afford the tetracyclic framework.

## Graphical abstract

The collective total syntheses of (±)‐hosieines A–C featuring a cage‐like tetracyclic framework have been realized. The key synthetic strategy features a one‐pot domino reaction that forms two bonds and three consecutive stereocenters to construct the desired 5/6/6‐aza tricyclic skeleton. Other salient synthetic tactics comprise a challenging double bond migration and a 1,4‐aza‐Michael addition reaction to afford the tetracyclic framework.

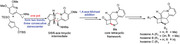

## 1 Introduction

**Figure 1:**
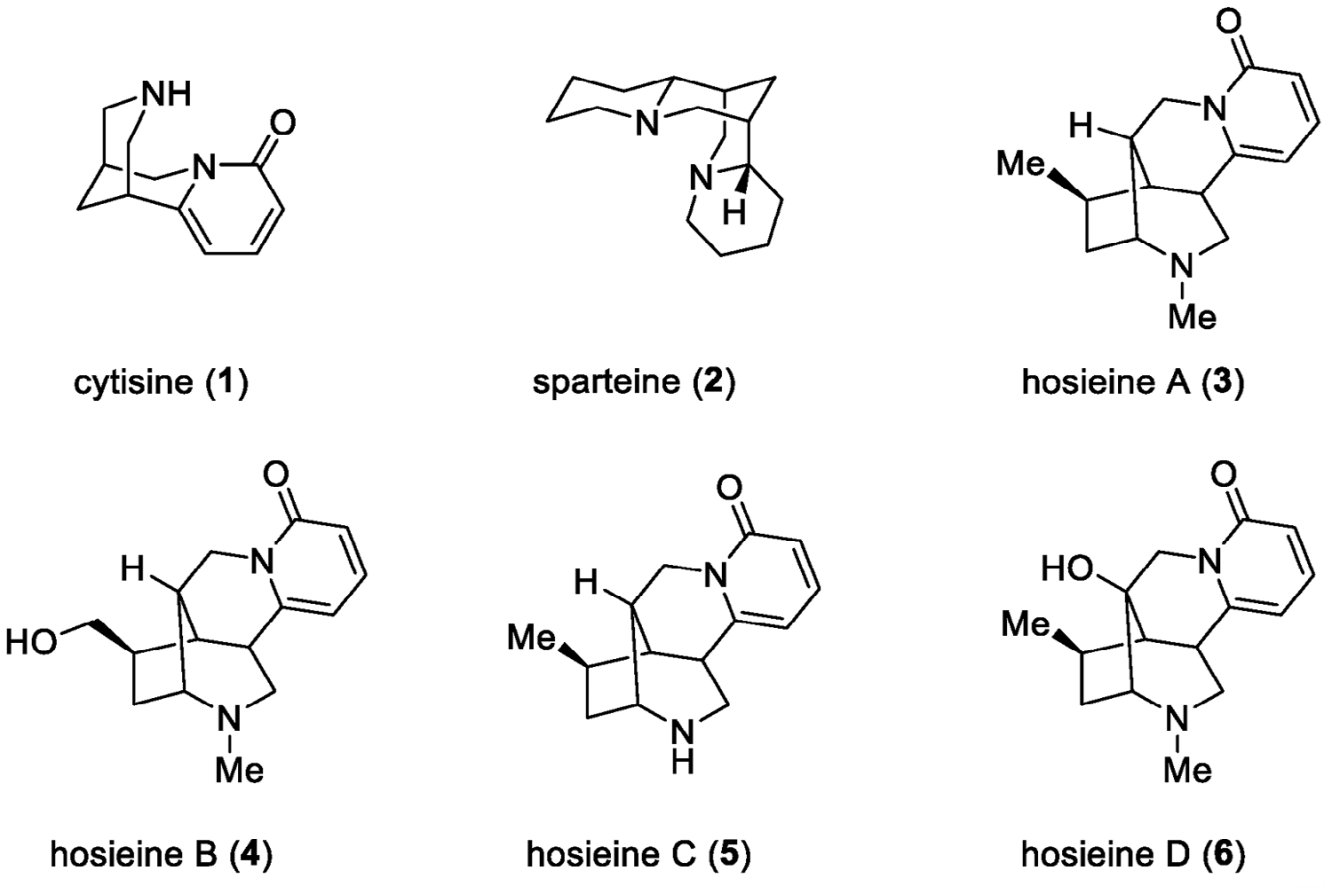
Representative lupin alkaloids 1–6.

Lupin alkaloids are a structurally diverse group of natural products. More than 200 structures within this category have been discovered so far, and most of them have shown interesting biological activities.^[^ ^1^, ^2^ ^]^ For example, cytisine (1), a partial agonist/antagonist of acetylcholine with a high affinity for nicotinic acetylcholine receptors (nAChRs), is approved for medical use as a smoking cessation aid (**Figure** **1**).^[^ ^3^ ^]^ Sparteine (2) exhibits anti‐arrhythmic, diuretic, and anti‐inflammatory activities and has been used as a Na^+^ and K^+^ channel blocker in biological studies, besides serving as a chiral auxiliary in asymmetric synthesis for a long time.^[^ ^4^ ^]^

**Scheme 1:**
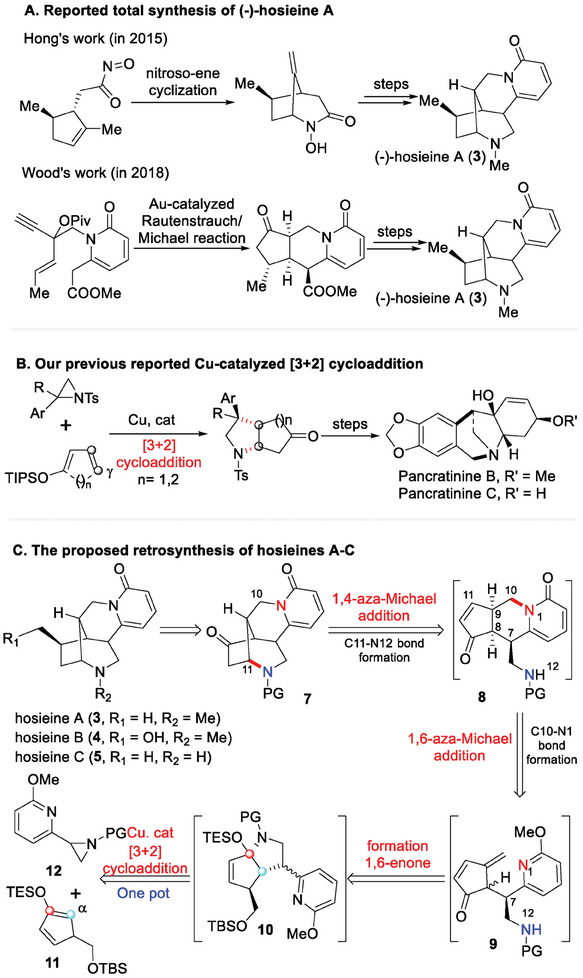
Collective Synthetic Design.

Hosieines A‐D are a new class of cytisine‐like lupin alkaloids, isolated by Massiot and co‐workers from the roots and stems of *Ormosia hosier Hemsl. & E.H. Wilson*.^[^ ^5^ ^]^ These four isolated congeners show high affinity toward α_4_β_2_–nAChRs, among which hosieine A is five times more active than nicotine with a nanomolar level potency (IC_50_ = 0.96 nM, K_i_ = 0.32 nM). Structurally, hosieines A‐D contain a unique cage‐like tetracyclic framework rarely found in natural products. Due to their promising bioactivity and remarkable structural complexity, hosieines A‐D have attracted the attention of synthetic chemists. Hong and Wood subsequently achieved the asymmetric synthesis of hosieine A, featuring a nitroso‐ene cyclization^[^ ^6^ ^]^ and a gold‐catalyzed Rautenstrauch/Michael reaction sequence,^[^ ^7^ ^]^ respectively (**Scheme** **1**A). Albeit these synthetic efforts, total syntheses of hosieines B‐D have yet been fulfilled. Herein, we report our collective syntheses of hosieines A–C using a novel and efficacious strategy.

Recently, our group developed a copper‐catalyzed [3+2] cycloaddition to prepare fused‐[5,n] carbocyclic pyrrolidines from simple and readily available cyclic dienes (γ‐position diene) and aziridines, which was further applied for syntheses of montanine‐type alkaloids pancratinines B–C (Scheme 1B).^[^ ^8^ ^]^ Inspired by this, we explored the possibility of using the copper‐catalyzed [3+2] cycloaddition to convert aziridine 12^[^ ^9^ ^]^ and α‐diene 11^[^ ^10^ ^]^ into^–^ [5, 5] aza‐bicycles 10 (Scheme 1C). The bicyclic compound 10 would serve as a pivotal intermediate, which might undergo desilylation and elimination to generate 1,6‐enone 9. The intermediate 9 could further participate in a regioselective 1,6‐Michael addition to form the C10‐N1 bond and give 5/6/6‐aza tricyclic intermediate 8, which would subsequently undergo a 1,4‐aza‐Michael addition to construct the C11‐N12 bond and afford the tetracyclic compound 7. The skeleton of tetracycle 7 closely resembles that of the hosieines family alkaloids, and the total syntheses of hosieines A–C could be completed after functional group transformations.

**Figure 2:**
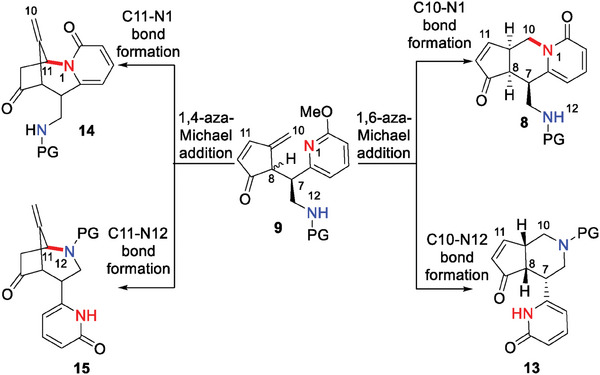
The chemo‐ and regioselectivity challenges.

By retrosynthetic analysis, the key synthetic difficulties of hosieines A–C include: 1) Whether aziridine 12 which contains an electron‐deficient pyridine ring and the unstable diene 11 may undergo the expected [3+2] cycloaddition reaction or not. 2) Starting from enone 9, the competing nucleophilicity of N1 and N12 created a chemoselectivity challenge, while both 1,6‐ and 1,4‐aza‐Michael cascade additions seem feasible, leading to a regioselective issue (**Figure** **2**). 3) Compound 9 is expected to undergo a 1,6‐aza‐Michael addition to produce tricycle 8, which has three stereocenters at C7, C8, and C9. How to control the 3D configuration of intermediate 8 is crucial for the following 1,4‐aza‐Michael addition to give tetracycle 7.

## 2 Results and Discussion

To start with, aziridine 12a and diene 11 were synthesized from known starting materials^[^ ^9^, ^10^ ^]^ in 1–2 steps (see Supporting Information for details), whereas the subsequent copper‐catalyzed [3+2] cycloaddition failed to proceed under various conditions (see **Table** **1**, entries 1 and 2, and Table S1, Supporting Information). Based on our prior experience in Cu‐catalyzed [3+2] cycloaddition,^[^ ^8^ ^]^ we speculated that the poor reactivity might arise from the low electron density on the pyridine ring of aziridine 12a. Consequently, we designed and prepared aziridine 12b‐c by introducing an electron‐donating OMe group onto the pyridine ring. This modification serves a dual purpose: first, it stabilizes the generated benzylic cation during the [3+2] cycloaddition process and accordingly increases the reactivity; second, it enhances the nucleophilicity of the pyridine nitrogen and therefore benefits the chemoselectivity of the competing 1,6‐aza‐Michael addition between N1 and N12. Gratifyingly, with copper catalysts, aziridine 12b and diene 11 underwent a domino reaction involving [3+2] cycloaddition, desilylation, 1,6‐enone formation, and 1,6‐aza‐Michael addition,^[^ ^11^ ^]^ and afforded tricyclic product 16b in one pot alongside side product 17 that derived from a rival 1,6‐aza‐Michael addition of N12 (Table 1, entries 3 and 4). The structure of 16b was determined by X‐ray crystallographic analysis (see Scheme 3).^[^ ^12^ ^]^ 1,4‐aza‐Michael addition was not observed, probably because it involves a bridged and strained ring structure that is energetically challenging to form. To improve the yield of tricyclic product 16, we synthesized aziridine 12c, featuring a stronger electron‐withdrawing Ns group in hopes of shutting down the undesired pathway of N12 1,6‐aza‐Michael addition. As envisioned, using aziridine 12c improved the chemoselectivity and accordingly gave tricycle 16c in an enriched 42% yield (Table 1, entries 5 and 6). Interestingly, lowering the reaction temperature gradually favored the tricyclic product 18 (Table 1, entries 7 and 8). Detailed optimization of reaction conditions can be found in Tables S1–S3, Supporting Information.

**Table 1:** Selected optimization of reaction conditions.

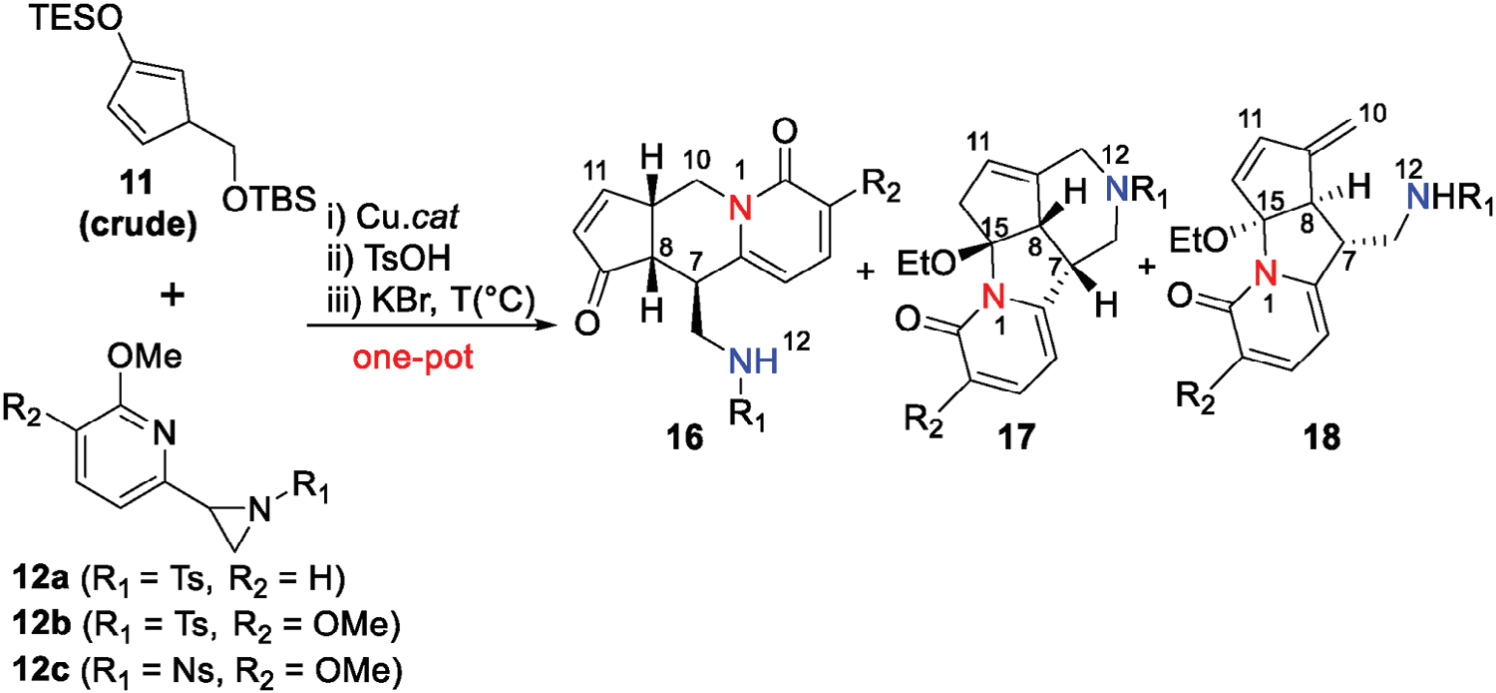							
Entry	R_1_	R_2_	Cu *cat*.	T (°C)	Yield (16)^a)^	Yield (17)^a)^	Yield (18)^a)^
1	Ts	H	Cu(MeCN)_4_BF_4_	–	–	–	–
2	Ts	H	Cu(MeCN)_4_OTf	–	–	–	–
3	Ts	OMe	Cu(MeCN)_4_OTf	100	16b (25%)	15%	–
4	Ts	OMe	Cu(MeCN)_4_BF_4_	100	16b (10%)	6%	–
5	Ns	OMe	Cu(MeCN)_4_OTf	100	16c (18%)	–	–
6	Ns	OMe	Cu(MeCN)_4_BF_4_	100	16c (42%)	–	–
7	Ns	OMe	Cu(MeCN)_4_BF_4_	80	16c (16%)	–	15%
8	Ns	OMe	Cu(MeCN)_4_BF_4_	60	16c (2%)	–	12%

All reaction were performed using 12 (0.1 mmol), 11 (0.3 mmol)

^a)^ Isolated Yield. Ts = p‐toluenesulfonyl, Ns = p‐nitrobenzenesulfonyl, TES = triethylsilyl.

**Scheme 2:**
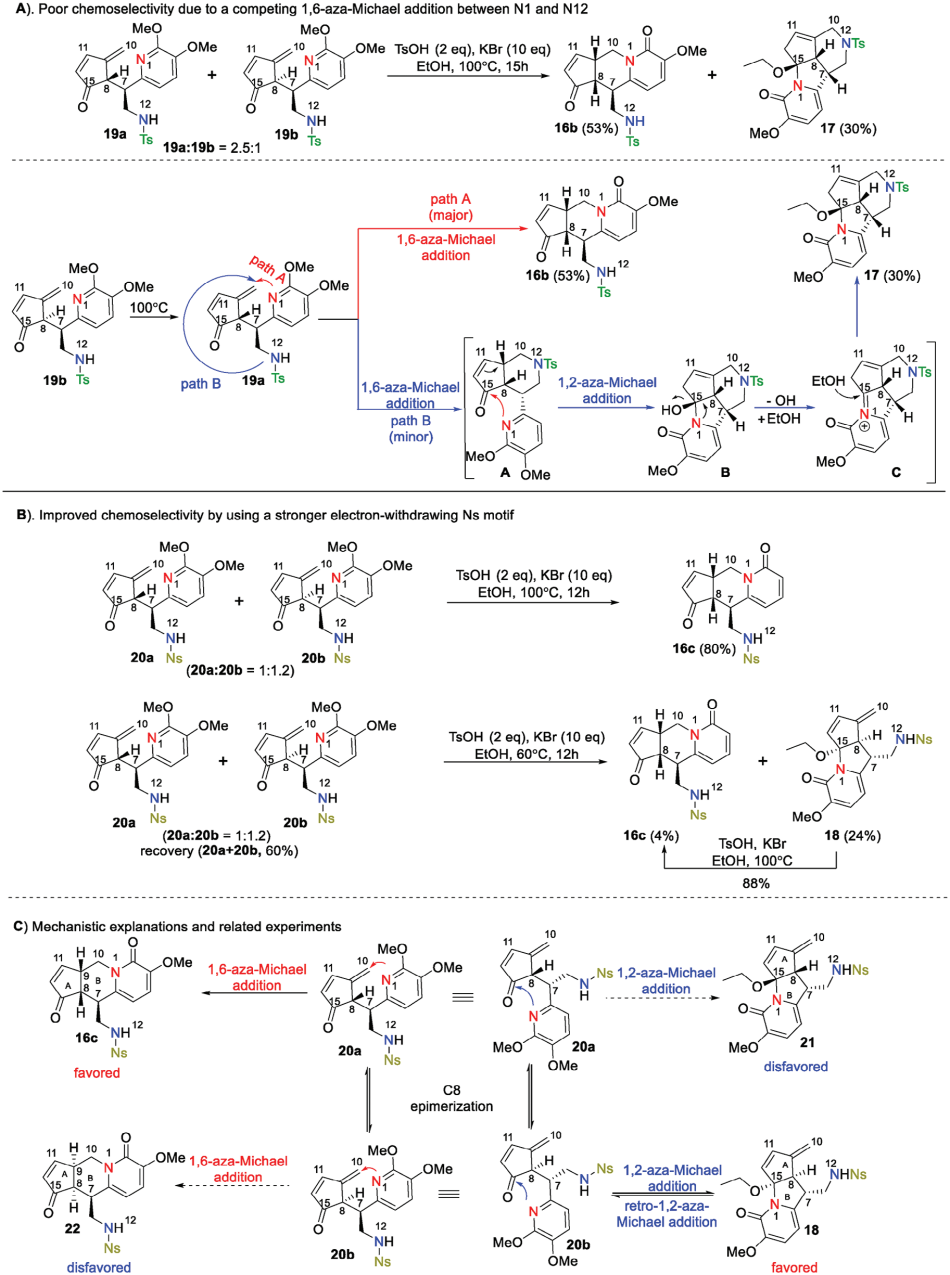
Mechanistic studies of the addition reactions of 1,6‐enone compounds 19a/b & 20a/b.

To figure out the mechanism of the intricating one‐pot cascade reaction, control experiments, reaction intermediate characterizations, and mechanism studies were carried out (**Scheme** **2**, and also see Tables S4, S5, and S13–S16, Supporting Information for more details). The Ts‐protected 1,6‐enone intermediates 19a/b were isolated as a 2.5:1 ratio. Subjecting this diastereomeric mixture to the reaction conditions provided the desired tricyclic product 16b together with the undesired tetracycle 17 in 53% and 30% yield, respectively (Scheme 2A). Intermediate 19b could quickly epimerize to its thermodynamically stable diastereomer 19a at a higher temperature, which is supported by our calculations that diastereomer 19a is 3.2 kcal/mol lower in energy than diastereomer 19b (see Table S13, Supporting Information). Compound 19a might undergo an anticipated N1 1,6‐aza‐Michael addition to give N12 1,6‐aza‐Michael addition (Scheme 2A, path B), yielding intermediate A that subsequently underwent a 1,2‐aza‐Michael addition to afford intermediate B and ultimately tetracycle 17. In contrast, using Ns‐masked substrates 20a/b at 100 °C, tricyclic compound 16c was solely isolated in 80% yield, likely because the improved electron‐withdrawing effect of Ns compared to that of Ts shut down the undesired pathway of the N12 1,6‐aza‐Michael addition (Scheme 2B). Intriguingly, lowering the reaction temperate to 60 °C not only slowed down the reactivity (60% recovered 20a/b) but also shifted the product distribution from the expected 16c to unexpected tricycle 18 (4% vs 24% yield) (Scheme 2B). After blocking the N12 1,6‐aza‐Michael addition with an Ns moiety, diastereomers 20a/b could theoretically undergo either a N1 1,6‐aza‐Michael addition giving products 16c/22 or a N1 1,2‐aza‐Michael addition leading to products 18/21 (Scheme 2C). Deuteration experiments proved that epimerization did occur at the C8 position at 60 °C (see Table S6, Supporting Information). And diastereomer 20b experimentally proved favorable to the N1 1,2‐aza‐Michael addition that afforded tricycle 18, whereas diastereomer 20a largely followed the N1 1,6‐aza‐Michael addition resulting in tricyclic product 16c (for more details, see Table S4, Supporting Information). We suspected the stereochemistry of the C7 side chain of diastereomers 20a/b played an important role in the product distribution during this process. Accordingly, energy minimizations for compounds 20a/b were carried out and the corresponding optimized geometry supported our assumption (See Section S6.3, Supporting Information). For diastereomer 20a, its C7 side chain placed the pyridine N1 close to C10 which is ready for the wanted 1,6‐aza‐Michael addition, whereas the C7 side chain of diastereomers 20b pushed the pyridine N1 close to C15 that set the stage for the unwanted 1,2‐aza‐Michael addition. Nevertheless, at a higher 100°C, the tricyclic byproduct 18 was found to cleanly transform to the desired tricycle 16c, likely via a process involving retro 1,2‐aza‐Michael addition, C8‐epimerization, and 1,6‐aza‐Michael addition (Scheme 2B, 18 → 16c, and for more details, see Table S5, Supporting Information).

**Scheme 3:**
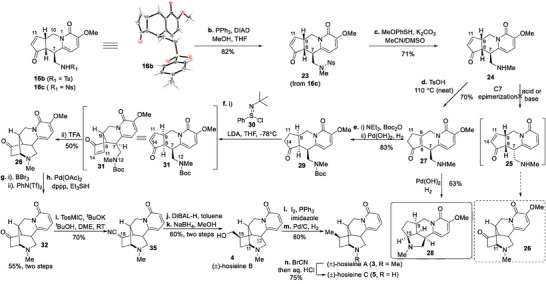
Total synthesis of hosieines A–C. Ts = p‐toluenesulfonyl, Ns = p‐nitrobenzenesulfonyl, TES = triethylsilyl, PPh_3_ = triphenylphosphine, DIAD = diisopropyl azodicarboxylate, LDA = lithium diisopropylamide, TFA = trifluoroacetic acid, dppp = 1,3‐bis(diphenylphosphino)propane, TosMIC = tosylmethyl isocyanide, DiBAL‐H = diisobutylaluminium hydride.

With tricycle 16c in hand, we faced the daunting challenge of constructing the required C7‐stereochemistry so as to form the key C11‐N12 bond (**Scheme** **3**). After multiple experiments, the study of direct epimerization of the C7 side chain proved fruitless. So, we attempted to first remove the Ns group of tricyclic compound 16c and then explored the isomerization of the C7 side chain. By using the Mitsunobu reaction^[^ ^13^ ^]^ to introduce a methyl group on nitrogen at 16c, followed by the removal of Ns groups with MeOPhSH and K_2_CO_3_,^[^ ^14^ ^]^ secondary amine 24 was obtained in 58% yield over two steps. We attempted to epimerize the C7‐stereochemistry of amine 24 to obtain 25 but failed. To our surprise, amine 24 underwent a double bond migration from C11–C14 to C8–C9 using TsOH at 110 °C, resulting in a more stable tetrasubstituted olefin 27. Next, we hoped that by reducing C8‐C9 olefin, the hydrogen atom would attack the double bond from the opposite side of the C7 side chain due to steric hindrance, providing us a product with the desired relative configuration at C7, C8, and C9 positions. However, under the conditions of Pd(OH)_2_ and H_2_, not only the tetrasubstituted alkene of compound 27 was reduced,^[^ ^15^ ^]^ but also an unwanted reductive amination occurred to exclusively yield byproduct 28. To avoid this problem, Boc protection was introduced on the secondary amine of compound 27, and then compound 29 was smoothly realized under hydrogenation conditions in 83% yield.

After obtaining 29, we expected to construct the cyclic double bond in order to obtain the Michael reaction precursor. However, popular methods, such as IBX oxidation,^[^ ^16^ ^]^ Saegusa‐Ito oxidation,^[^ ^17^ ^]^ and selenide‐selenium oxide elimination,^[^ ^18^ ^]^ failed to do so. Finally, under Mukaiyama's conditions,^[^ ^19^ ^]^ we successfully obtained compound 31 with a double bond being set at the C11–C14 position. Removal of the Boc protecting group followed by a 1,4‐aza‐Michael addition successfully provided us the tetracyclic compound 26 in 50% yield. Subsequent demethoxylation was achieved by stepwise demethylation with BBr_3_, triflation with PhN(Tf)_2_, and Pd‐catalyzed reduction with Et_3_SiH, affording compound 32 in 55% yield.

With compound 32 secured, we turned our attention to the total synthesis of hosieines A–C (3–5). In the attempt to convert the ketone functionality of 32 to exomethylene, the standard Wittig methylenation as well as the salt‐free variations failed, and the Lebel‐modified Wittig procedure gave the olefin 33 in 73% yield (**Table** **2**).^[^ ^20^ ^]^ The diastereoselective hydrogenation of the exocyclic olefin in 28 was found to be extremely challenging, commonly used transition metal catalysts, such as Pd/C, Rh,^[^ ^21^ ^]^ and PtO_2_,^[^ ^22^ ^]^ afforded the major product was epi‐hosieine A (34) (Table 2, entries 1–4). Additionally, the hydrogen atom transfer (HAT) reaction^[^ ^23^ ^]^ was also investigated, yet without improvement (Table 2, entry 5).

**Table 2:** Screening of reduction for exocyclic alkenes of compound 33.

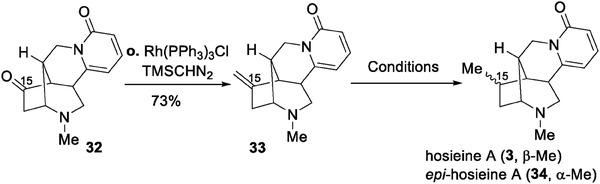			
Entry	Conditions	Yield of (3 + 34)^a)^	d.r. (3:34)^b)^
1	Pd/C, H_2_, MeOH, RT	70%	1:3.6
2	[Rh(cod)Cl]_2_, AgBF_4_, PPh_3_, H_2_, DCM	70%	1:2.9
3	Rh(PPh_3_)_3_Cl, H_2_, DCM	73%	1:2.7
4	PtO_2_, H_2_, MeOH, RT	80%	1:2.4
5	Fe(acac)_2_, PhSiH_3_, EtOH, 40 °C	67%	1:10

All reaction were performed using 33 (4 mg)

^a)^ Isolated Yield

^b)^ The d.r. (3:34) value was determined by **^1^** H NMR spectroscopy.

To optimize the synthesis route, we circled back to ketone 32. And Van Leusen reaction was performed to deliver nitrile 35 with a 70% yield (1:1 d.r. at C15).^[^ ^24^ ^]^ Reduction of the nitrile group of 35 by DiBAl‐H afforded an aldehyde intermediate, and we were pleased to find that the aldehyde group at C15 underwent isomerization to the desired configuration during the column chromatography purification. Subsequently, the reduction of the aldehyde intermediate with NaBH_4_ accomplished the total synthesis of hosieine B (4) with a 60% yield in two steps. Ultimately, hosieine B (4) was smoothly converted into hosieine A (3) by a sequence of Appel reaction (PPh_3_, I_2_) and reduction (Pd/C, H_2_).^[^ ^25^ ^]^Next, hosieine A (3) underwent von Braun demethylation to form hosieine C (5) in 75% yield.^[^ ^26^ ^]^ The characterization data of our synthetic hosieines A–C were in agreement with the reports of these natural products.

## 3 Conclusion

In summary, we have achieved the total syntheses of hosieines A–C (3‐5) in 11–14 steps from the easily prepared aziridine and diene, of which hosieines B–C are reported for the first time. Our synthetic strategy features a domino reaction to construct a 5/6/6‐aza tricyclic skeleton in one pot, involving sequential Cu‐catalyzed [3+2] cycloaddition, 1,6‐enone formation, and 1,6‐aza‐Michael addition. Additionally, constructing the desired stereochemistry of C15 is challenging but accessible. The asymmetric Cu‐catalyzed [3+2] cycloaddition reaction and further synthetic applications are currently underway and will be reported in due course.

## Conflict of Interest

The authors declare no conflict of interest.

## Supporting information

Supporting Information

## Data Availability

The data that support the findings of this study are available in the Supporting Information.
