# Supplementary material for: Total Syntheses of Hosieines A–C
Source: Adv Sci (Weinh). 2024 Feb 7;11(14):2308164. doi: 10.1002/advs.202308164 (PMC11005691; doi:10.1002/advs.202308164)
Supplement: Supplementary file 1 — Supporting Information [file ADVS-11-2308164-s001.pdf]

ADVANCED SCIENCE
Open Access

Supporting Information

for *Adv. Sci.*, DOI 10.1002/advs.202308164

Total Syntheses of Hosieines A–C

*Jiayang Zhang, Xu Yan, Qing-Bao Zhang, Fang Wang, Bin Yang and Yang Yang**

Supporting Information

Total Synthesis of Hosieines A-C

Jiayang Zhang, Xu Yan, Qing-Bao Zhang, Fang Wang, Bin Yang, and Yang Yang *

School of Pharmacy, Huazhong University of Science and Technology,

13 Hangkong Road, Wuhan, Hubei, 430030 (China)

yang_yang@hust.edu.cn

Table of Contents

1. General experimental 2

2. Detail of the screening conditions for constrcting 5/6/6 tricyclic skeleton compound 16 3

3. Experimental procedures and characterization data for compounds 8

4. NMR comparison of synthetic and natural hosieines 31

5. X-Ray Crystal Structure of 16b 37

6. DFT Calculations 38

7. Reference 62

8. NMR spectra: 63

1. General experimental

All reactions sensitive to air or moisture were carried out under argon or nitrogen atmosphere in dry, freshly distilled solvents under anhydrous conditions, unless otherwise noted. Anhydrous THF and toluene were distilled over sodium benzophenone ketyl under N_2. Anhydrous CH_2Cl_2 was distilled over calcium hydride under N_2. Anhydrous MeOH was distilled over magnesium under N_2. All other solvents and reagents were used as obtained from commercial sources without further purification. Reactions were magnetically stirred and monitored by thin layer chromatography (TLC) with 0.15-0.2 mm pre-coated silica gel (10-40 μm) plates, using UV light as the visualizing agent or aqueous potassium permanganate and ethanolic phosphomolybdic acid as developing agents. Column chromatography was performed with silica gel (200-300 mesh) under pressure. NMR spectra were recorded on (^1H at 400 MHz, 600 MHz and ^{13}C at 100 MHz, 150 MHz) Bruker spectrometers. Chemical shifts (δ) were given in ppm with reference to solvent signals [^1H NMR: $CHCl_3$ (7.26), CD_3OD (3.31); ^{13}C NMR: $CDCl_3$ (77.2), CD_3OD (49.0)]. The following abbreviations were used to explain multiplicities: s = singlet, d = doublet, t = triplet, q = quartet, m = multiplet, br = broad. IR spectra were collected on Perkin Elmer FT-IR L1600300 spectrometer. Optical rotations were determined on Rudolph Autopol IV automatic Polarimeter in the solvent indicated. High-resolution mass spectra were recorded on Bruker microTOF II.

2. Detail of the screening conditions for constrcting 5/6/6 tricyclic skeleton compound 16

Table S1. Screening conditions for the synthesis of 1,6-enone.

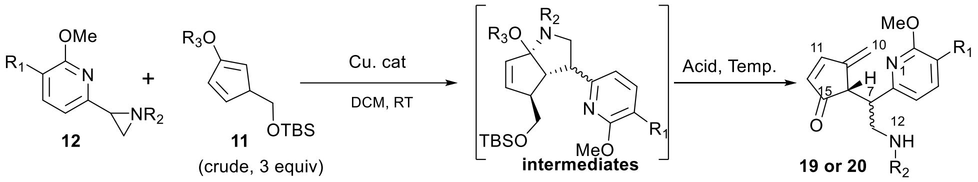

Entry	R			Catalyst (0.2 equiv)	Acid (2.0 equiv)	Temp (°C)	Yield[a] of **19 or 20**
	R_1	R_2	R_3				
1	H	Ts	TES	$Cu(MeCN)_4BF_4$	-	-	NR[b]
2	H	Ts	TES	$Cu(MeCN)_4OTf$	-	-	NR
3	H	Ts	TES	$Cu(OTf)_2$	-	-	NR
4	H	Ts	TES	$Cu(MeCN)_4PF_6$			NR
5	OMe	Ts	TES	$Cu(MeCN)_4BF_4$	$TsOH \cdot H_2O$	60	**19** (18%)
6	OMe	Ts	TES	$Cu(OTf)_2$	$TsOH \cdot H_2O$	60	**19** (31%)
7	OMe	Ts	TES	$Cu(MeCN)_4PF_6$	$TsOH \cdot H_2O$	60	**19** (38%)
8	OMe	Ts	TES	$Cu(MeCN)_4OTf$	$TsOH \cdot H_2O$	60	**19** (41%)
9	OMe	Ts	TBS	$Cu(MeCN)_4OTf$	$TsOH \cdot H_2O$	60	**19** (35%)
10	OMe	Ts	TES	$Cu(MeCN)_4OTf$	CSA	60	**19** (37%)
11	OMe	Ts	TES	$Cu(MeCN)_4OTf$	TFA	60	**19** (24%)
12	OMe	Ts	TES	$Cu(MeCN)_4OTf$	$TsOH \cdot H_2O$	50	**19** (53%)
13	OMe	Ts	TES	$Cu(MeCN)_4OTf$	$TsOH \cdot H_2O$	40	**19** (45%)
14	OMe	Ns	TES	$Cu(MeCN)_4OTf$	$TsOH \cdot H_2O$	50	**20** (24%)
15	OMe	Ns	TES	$Cu(MeCN)_4PF_6$	$TsOH \cdot H_2O$	50	**20** (32%)
16	OMe	Ns	TES	$Cu(MeCN)_4BF_4$	$TsOH \cdot H_2O$	50	**20** (51%)

All reaction were performed using **12** (0.1mmol). [a] Isolated Yield. [b] NR = no reaction. Ts = p-toluenesulfonyl; TES = triethylsilyl; TsOH = p-toluenesulfonic acid; TBS = tert-butyldimethysilyl; CSA = (+)-camphor-10-sulfonic acid; Ns = 4-nitrobenzene-1-sulfonyl.

Table S2. Screening conditions of 1,6-aza-Michael addition.

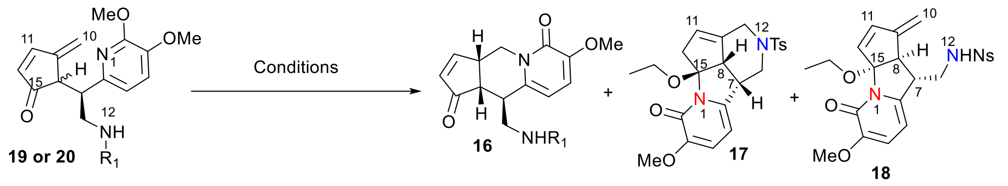

Entry	R1	Acids (equiv)	Additives (equiv)	Temp (℃)	solvent	Results
1	Ts	TMSI (5.0)	-	RT	MeCN	Complex
2	Ts	TMSCl (5.0)	NaI (10.0)	RT	MeCN	Complex
3	Ts	TsOH • H_2O (2.0)	-	80	toluene	NR[a]
4	Ts	TsOH • H_2O (2.0)	KBr (5.0)	50	EtOH	NR[a]
5	Ts	TsOH • H_2O (2.0)	KBr (5.0)	100	EtOH	**16b** (42%), **17** (27%)[b]
6	Ts	TsOH • H_2O (2.0)	KBr (10.0)	100	EtOH	**16b** (53%), **17** (30%)
7	Ts	TsOH • H_2O (2.0)	KBr (10.0)	110	EtOH	**16b** (40%), **17** (27%)
8	Ns	TsOH • H_2O (2.0)	KBr (10.0)	100	EtOH	**16c** (80%)
9	Ns	TsOH • H_2O (2.0)	KBr (10.0)	110	EtOH	**16c** (74%)
10	Ns	TsOH • H_2O (2.0)	KBr (10.0)	80	EtOH	**16c** (31%), **18** (30%)
11	Ns	TsOH • H_2O (2.0)	KBr (10.0)	60	EtOH	**16c** (4%), **18** (24%)

All reactions were performed using **19** or **20** (0.1mmol). [a] NR = no reaction. [b] Isolated Yield. TMSI = iodotrimethylsilane; TMSCl = chlorotrimethylsilane

Table S3. One pot to synthesis 5/6/6-azatricyclic skeleton

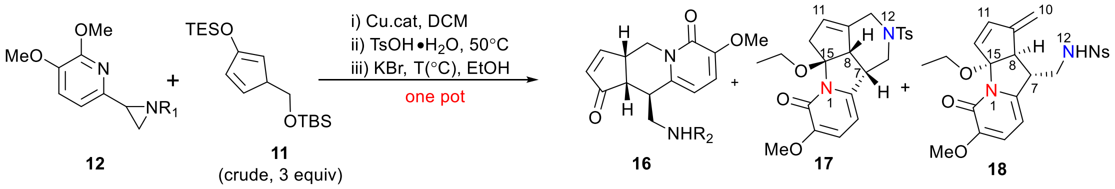

Entry	R2	Catalyst (0.2 equiv)	T (℃)	TsOH (equiv)	KBr (equiv)	Results
1	Ts	$Cu(MeCN)_4OTf$	100	2.0	10.0	**16b** (25%), **17** (15%)[a]
2	Ts	$Cu(MeCN)_4BF_4$	100	2.0	10.0	**16b** (10%), **17** (6%)
3	Ns	$Cu(MeCN)_4OTf$	100	2.0	10.0	**16c** (18%)
4	Ns	$Cu(MeCN)_4BF_4$	100	2.0	10.0	**16c** (42%)
5	Ns	$Cu(MeCN)_4BF_4$	80	2.0	10.0	**16c** (16%), **18** (15%)
6	Ns	$Cu(MeCN)_4BF_4$	60	2.0	10.0	**16c** (2%), **18** (12%)

All reaction were performed using **12** (0.1mmol). [a] Isolated Yield

To understand why tricyclic compound **16c** could be obtained with high regioselectivity and diastereoselectivity, we conducted a detailed study on the one-pot synthesis. separated and characterized the intermediates of the one pot method, namely [5.5]-aza-bicycles **36a/b**, ring opening compound **37a/b**, removing TBS protective compound **38a/b**, and 1,6-enone compounds **20a/b** (Scheme S1). Under the conditions of $Cu(MeCN)_4BF_4$ as catalyst and 25°C, aziridine **12c** and diene **11** undergo [3+2] cycloaddition reaction to obtain [5.5]-aza-bicycles **36a/b** (38%, **36a:36b** = 1:1.3) and compound **37a/b** (33%, **37a:37b** = 1.5:1) were isolated. Compound **36a/b** was separated by HPLC and the relative configuration was determined by 2D NMR spectra analysis (DEPT-135, COSY, HSQC, HMBC, NOESY). [5.5]-aza-bicycles **36a/b** would undergo a ring-opening reaction under the conditions of $Cu(MeCN)_4BF_4$ to obtain compound **37a/b**, which proves that compound **37a/b** were converted from compound **36a/b** through retro-1,2-aza-Michael reaction. Compound **36a** was subjected to a 0.5-hour treatment with $Cu(MeCN)_4BF_4$, resulting in the formation of compound **37a**. Subsequently, compound **37a** underwent a reaction with TsOH • H_2O, yielding compounds **38a** and **20a**. In a parallel synthesis, compound **36b** was treated with $Cu(MeCN)_4BF_4$ for 0.5 hours to produce compound **37b**. This was followed by a reaction of compound **36b** with TsOH, leading to the formation of compound **38b**. Finally, compound **38b** was treated with TFA, affording compound **20b**.

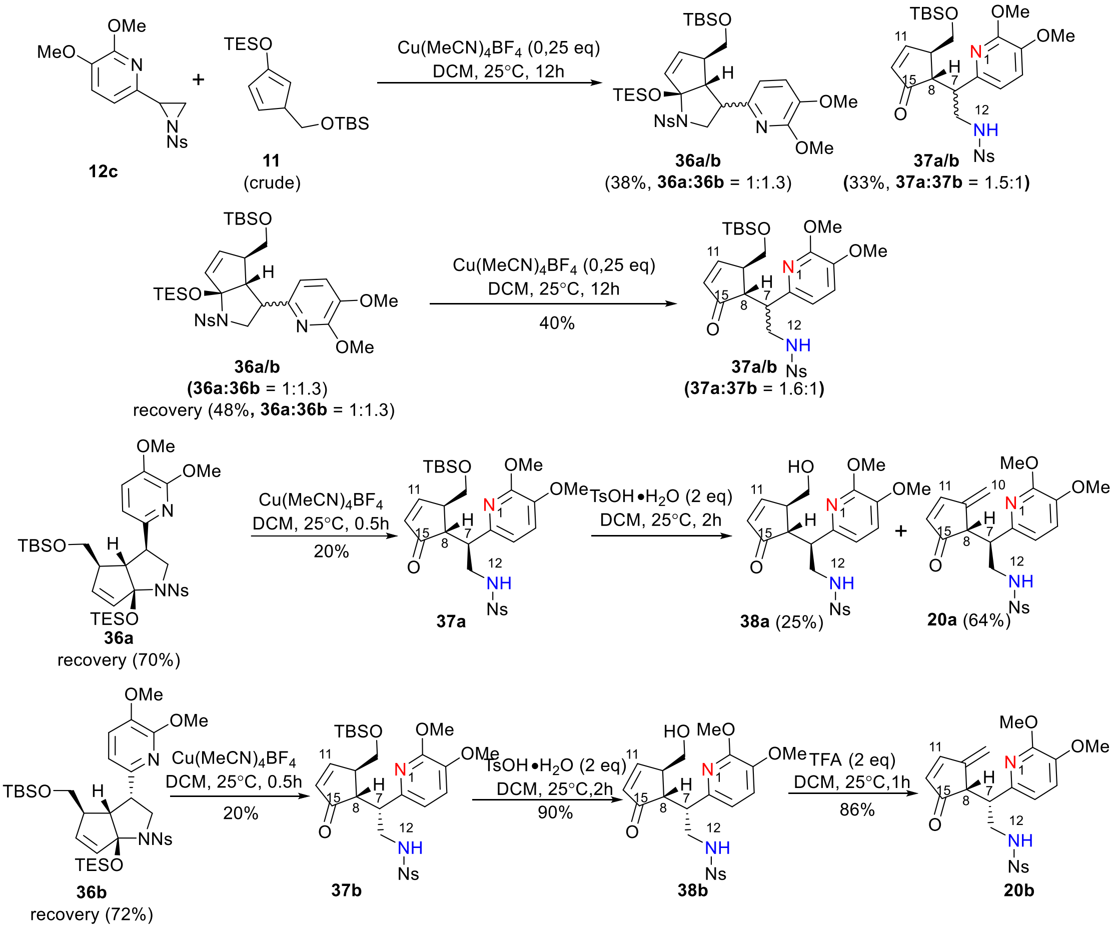

Scheme S1. Isolation and identification of intermediates **36a/b**, **37a/b**, **38a/b** and **20a/b**.

Table S4. Study on the regioselectivity and diasteroselectivity of compound 20a/b at different temperatures.

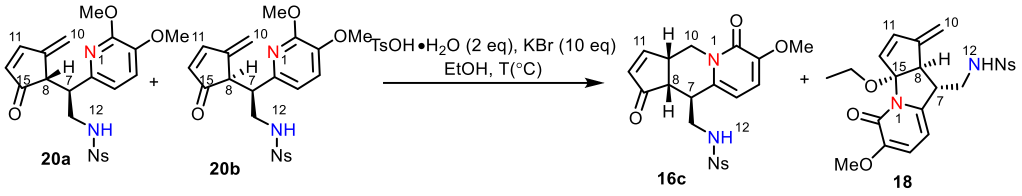

Entry	substrates	T(°C)	Yields (**20a** and **b**)[c] d.r. (**20a:20b**)[d]	Yields (**18**)	Yields (**16c**)
**1**	only **20a**	25	98% (only **20a**)	-	-
**2**	only **20b**	25	97% (3.2:1)	-	-
**3**	only **20a**	40	96% (10:1)	-	-
**4**	Only**20b**	40	95% (3.1:1)	-	-
**5**	only **20a**	60	58% (4.4:1)	25%	3%
**6**	Only**20b**	60	57% (2.9:1)	26%	3%
**7**	(**20a:20b** = 1:1.2)	60	60% (3.0:1)	24%	4%
**8**	(**20a:20b** = 1:1.2)	80	24% (2.7:1)	30%	31%
**9**	(**20a:20b** = 1:1.2)	100	-	-	80%

[a]Unless otherwise noted, the reaction was carried out in TsOH • H_2O (2.0 equiv), KBr (10.0 equiv), EtOH (0.028 M), 12h; [b]Entry 1-6 were run for (15 mg), Entry 7-9 were run for (50 mg); [c]Isolated; [d]The d.r. value was determined by 1H NMR spectroscopy; TsOH = p-toluenesulfonic acid; Ns = 4-nitrobenzene-1-sulfonyl.

After several preliminary experiments, we found that compound **20a** does not epimerize to **20b** at 25 °C (entry 1), but as the temperature increases, compound **20a** undergoes epimerization to **20b**, and the rate of epimerization accelerates with increasing temperature (entry 3, 5). Compound **20b** readily undergoes epimerization to **20a** at temperatures above 25°C (Entry 2, 4, 6). These results indicate that compound **20a/b** can undergo epimerization at high temperature. Under the condition of 60 °C, compound **20a/b** mainly undergoes 1,2 aza-Michael addition to obtain product **18** and a small amount of 1,6 aza-Michael addition product **16c** (entries 5, 6, 7). The yield of compound **16c** increased significantly with the elevation of temperature (Entry 7-9). At 100 °C, compound **20 a/b** can be completely transformed, resulting in compound **16c** with 80% yield (entry 9).

Table S5. Exploration of the conversion of compound 18 to 16c at different temperatures.

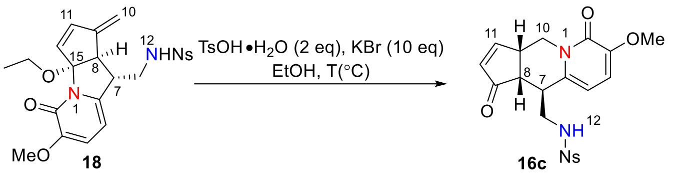

Entry	T(℃)	Recovery (**18**)[c]	Yields (**16c**)[c]
1[b]	60	84%	6%
2[b]	80	43%	45%
3[c]	100	-	88%

[a]Unless otherwise noted, the reaction was carried out in **18** (10 mg), TsOH • H_2O (2.0 equiv), KBr (10.0 equiv), EtOH (0.028 M); [b]Entry 1-2 were run for 12h, Entry 3 were run for 2h; [c]Isolated; TsOH = p-toluenesulfonic acid; Ns = 4-nitrobenzene-1-sulfonyl.

Compound **18** was converted to compound **16c** at high temperature, and the conversion rate increased with the temperature. At 100°C, compound **18** was completely converted into compound **16c** with 88% yield in 2 hours.

Table S6. Experiments with Deuterated Solvent

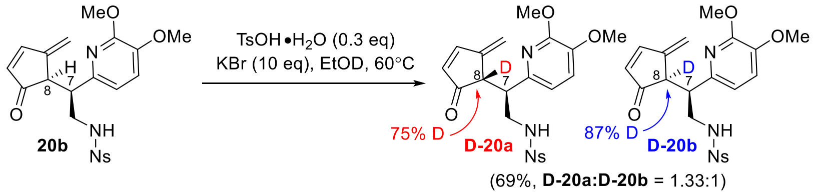

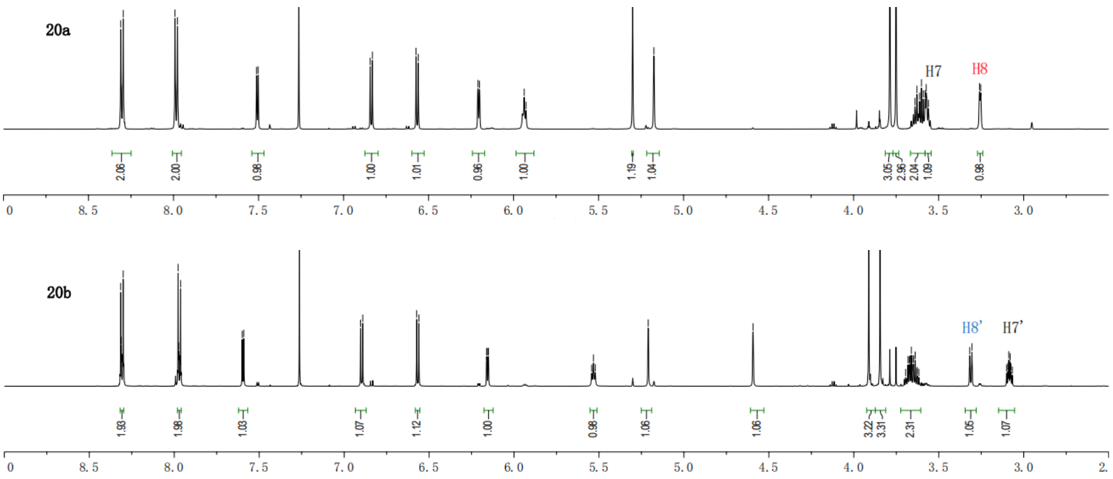

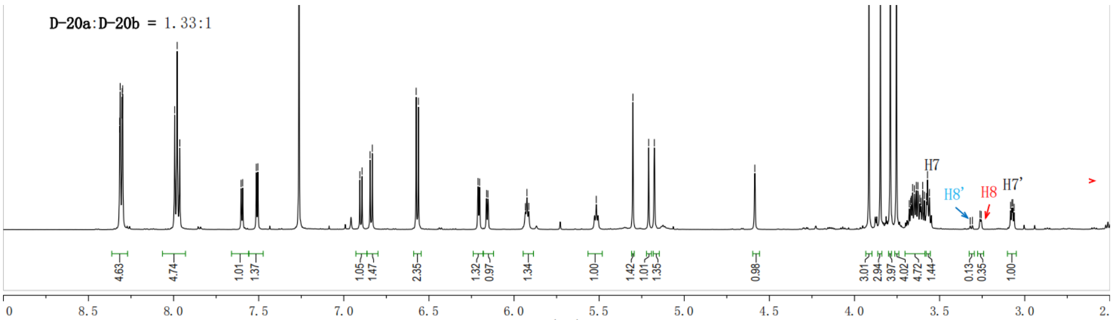

To a stirred solution of **20b** (13 mg, 0.028 mmol, 1.0 equiv) in EtOD (1 mL) was added TsOH • H_2O (1.6 mg, 0.0085 mmol, 0.3 equiv) and KBr (33.7 mg, 0.28 mmol, 10 eq) at room temperature, and the reaction mixture was then heated to 60℃ using an oil bath. After stirred for 12 h, the reaction was quenched with aq. $NaHCO_3$ (1 mL) and extracted with DCM (3 x 3 mL). The combined organic layers were washed with brine (4 mL), dried ($MgSO_4$), filtered and concentrated. The crude product was purified by column chromatography(petroleum ether/EtOAc/DCM, 8:2:3 → 6:3:3) to afford an inseparable diastereomeric mixture of compound **D-20a/b** (9 mg, 69%, **D-20a:D-20b** = 1.33 :1) as a pale-yellew solid. ^{1}H NMR indicated that the D-ratio of **compound D-20a** at **H8** (*red*) was 75%, the D-ratio of **compound D-20b** at **H8** (*Blue*) was 87%, The H7 position of **D-20a/b** were not deuterated. *Note: the ^{1}H NMR chemical shift of H7, H8 for* **D-20a** *and* **D-20b** *was assigned by (HSQC, ^{1}H-^{1}H-COSY)*

3. Experimental procedures and characterization data for compounds

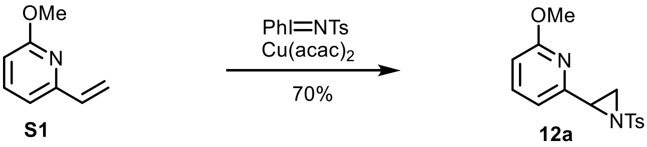

Following a reported procedure [1] with slight modifications, to a solution of alkene **S1**[2] (1.22 g, 8.88 mmol, 2.0 equiv) and PhI=NTs [3] (1.65g, 4.44 mmol, 1.0 equiv) in dry MeCN (25 ml) was added Cu(acac)$_2$ (92 mg, 0.36 mmol, 0.08 equiv) at RT. After stirring at RT for 40 min, the reaction mixture was filtered through a pad of Celite. The filtrate was concentrated *in vacuo* and purified by flash column chromatography (petroleum ether/EtOAc, 6:1 → 3:1) to afford aziridine **12a** (810 mg, 60%) as a pale-yellow solid.

Compound 12a: TLC (petroleum ether:EtOAc, 4:1 v/v): R_f = 0.30 (UV, $KMnO_4$); ^{1}H NMR (600 MHz, $CDCl_3$) δ: 7.86 (d, J = 8.5 Hz, 2H), 7.47 (dd, J = 8.5, 7.3 Hz, 1H), 7.32 (d, J = 8.5 Hz, 2H), 6.82 (d, J = 7.5 Hz, 1H), 6.62 (d, J = 8.5 Hz, 1H), 3.86 (s, 3H), 3.81 (dd, J = 7.3, 4.3 Hz, 1H), 2.92

(d, J = 7.3 Hz, 1H), 2.71 (d, J = 4.3 Hz, 1H), 2.42 (s, 3H); ^{13}C NMR (150 MHz, $CDCl_3$) δ: 163.8, 151.7, 144.7, 139.1, 134.9, 129.7 (2C), 128.1 (2C), 114.5, 110.6, 53.5, 41.5, 34.3, 21.7; IR (KBr): 1485, 1405, 1334, 1240, 1170, 1043, 932, 748, 613 cm^{-1}; HRMS (ESI, m/z) calcd for $C_{15}H_{17}N_2O_3S$[M+H]$^+$: 305.0954, found 305.0969.

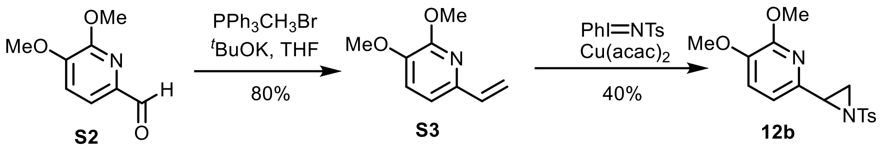

To a solution of PPh_3CH_3Br (100 g, 281 mmol, 1.3 equiv) in dry THF (600 ml) were added ^{t}BuOK (31.5 g, 281 mmol, 1.3 equiv) at 0℃. After stirring at 0℃ for 1h, a solution of **S2**[4] (36 g, 216 mmol, 1 equiv) in dry THF (300 ml) was added to the reaction at 0℃. The reaction was warmed to RT and stirred at this temperature until the starting material was completely consumed. The resulting mixture was filtered through a pad of Celite and the filtrate was concentrated in vacuo. The residue was purified by column chromatography (petroleum ether/EtOAc, 20:1 → 4:1) to afford **S3** (28.8 g, 81%) as pale-yellow oil.

To a solution of alkene **S3** (1.47 g, 8.88 mmol, 2.0 equiv) and PhI=NTs (1.65g, 4.44 mmol, 1.0 equiv) in dry MeCN (25 ml) was added $Cu(acac)_2$ (92 mg, 0.36 mmol, 0.08 equiv) at RT. After stirring at RT for 40 min, the reaction mixture was filtered through a pad of Celite. The filtrate was concentrated *in vacuo* and purified by column chromatography (petroleum ether/EtOAc, 10:1 → 3:1) to afford aziridine **12b** (1.05 g, 71%) as a pale-yellow solid.

Compound 12b: TLC (petroleum ether:EtOAc, 4:1 v/v): R_f = 0.30 (UV, $KMnO_4$); ^{1}H NMR (600 MHz, $CDCl_3$) δ: 7.87 (d, J = 8.5 Hz, 2H), 7.33 (d, J = 8.5 Hz, 2H), 6.94 (d, J = 7.7 Hz, 1H), 6.83 (d, J = 7.8 Hz, 1H), 3.93 (s, 3H), 3.84 (s, 3H), 3.79 (dd, J = 7.3, 4.7 Hz, 1H), 2.92 (d, J = 7.3 Hz, 1H), 2.75 (d, J = 4.7 Hz, 1H), 2.44 (s, 3H); ^{13}C NMR (150 MHz, $CDCl_3$) δ: 153.9, 144.6, 144.1, 141.1, 135.0, 129.7 (2C), 128.0 (2C), 117.2, 115.2, 55.8, 55.6, 46.4, 34.1, 21.7; IR (KBr): 1485, 1404, 1323, 1238, 1169, 1013, 892, 729, 560, cm^{-1}; HRMS (ESI, m/z) calcd for $C_{16}H_{19}N_2O_4S$[M+H]$^+$:335.1060, found 335.1072.

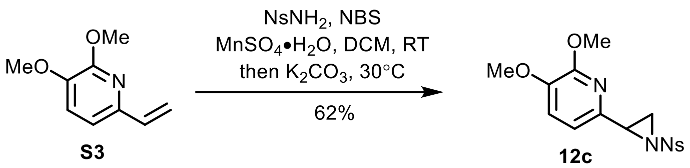

To a solution of **S3** (5.0 g, 30.0 mmol, 1 equiv), $NsNH_2$ (6.4 g, 31.5 mmol, 1.05 equiv) and NBS (5.90 g, 33.0 mmol, 1.1 equiv) in dry DCM (350 ml) were added $MnSO_4 \cdot H_2O$ (256 mg, 1.5 mmol, 0.05 equiv) at RT. After stirring at RT for 1h, K_2CO_3 (8.28 g, 60.0 mmol, 2.0 equiv) was added, the reaction stirred at 30℃ for 20h, another portion of K_2CO_3 (8.28 g, 60.0 mmol, 2.0 equiv) was added and stirring was continued for 20 h. The resulting mixture was filtered through a pad of Celite and the filtrate was concentrated in vacuo. The residue was dissolved in DCM (1000 ml) and washed with H_2O (300 ml*3), then the organic phase dried ($MgSO_4$), filtered and concentrated to afford crude product. The crude product was washed with DCM (10 ml*3) to afford **12c** (6.8 g, 62%) as a pale-yellow solid.

Compound 12c: TLC (PE:EA, 4:1 v/v):R_f = 0.3 (UV, $KMnO_4$); ^{1}H NMR (600 MHz, $CDCl_3$) δ: 8.38 (d, *J* = 8.9 Hz, 2H), 8.19 (d, *J* = 8.9 Hz, 2H), 6.96 (d, *J* = 7.7 Hz, 1H), 6.83 (d, *J* = 7.7 Hz, 1H), 3.94 (s, 3H) 3.90 (dd, *J* = 7.8, 3.0 Hz, 1H), 3.85 (s, 3H), 3.05 (d, *J* = 7.8 Hz, 1H), 2.91 (d, *J* = 3.0 Hz, 1H); ^{13}C NMR (150 MHz, $CDCl_3$) δ: 154.2, 150.6, 144.5, 144.1, 140.0, 129.3 (2C), 124.3 (2C), 117.1, 115.6, 55.8, 53.7, 42.3, 34.5; IR (KBr): 1585, 1539, 1485, 1410, 1352, 1335, 1236, 892, 789; HRMS (ESI, m/z) calcd for $C_{15}H_{16}N_3O_6S[M+H]^+$: 366.0754, found 366.0766.

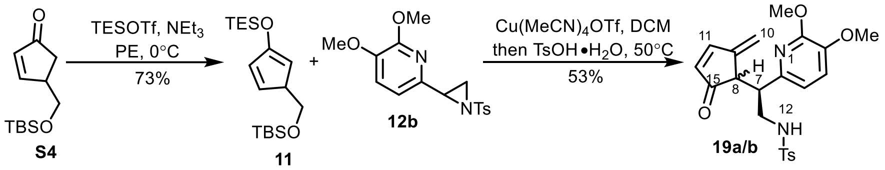

To a solution of **S4** (4.0 g, 17.7 mmol, 1 equiv) in dry petroleum ether (35 ml) were added NEt_3 (4.9 ml, 35.4 mmol, 2.0 equiv) and TESOTf (4.0 ml, 17.7 mmol, 1 equiv) at 0℃, After stirring at the same temperature for 2 h, decant the supernatant and dilute it with petroleum ether (50 ml). The organic phase was washed with aqueous pH = 7 buffer (50 ml*2), H_2O (50 ml*2) , and brine (50 ml*2), then dried ($MgSO_4$), filtered and concentrated under reduced pressure in 10℃. Further removal of volatile material under high vacuum at 0 ℃ for 4h to afford the crude **11** (4.3 g, 72%). The compound **11** is unstable and needs to be prepared and used immediately.

To a solution of **12b** (33.4 mg, 0.1 mmol, 1 equiv) and **11** (102 mg, 0.3 mmol, 3 equiv) in dry DCM (1 ml) was added Cu(MeCN)$_4$OTf (7.6 mg, 0.02 mmol, 0.2 equiv) at RT. After stirring at RT for 12h, TsOH • H_2O (38 mg, 0.2 mmol, 2 equiv) was added to the reaction system. The resulting mixture was warmed to 50℃ and stirred at this temperature for 2h. The reaction mixture was quenched with aq. $NaHCO_3$ (2 ml) and extracted with DCM (3 ml*3). The combined organic layers were washed with brine (4 mL), dried ($MgSO_4$), filtered and concentrated. The residue was purified by column chromatography (petroleum ether/EtOAc/DCM, 8:2:3→6:3:3) to afford compound **19a/b** (22.7 mg, 53%, **19a:19b** = 2.5 :1) as a white solid.

Compound 19a/b: TLC (PE:EA:DCM, 2:1:2 v/v):R_f = 0.5 (UV, $KMnO_4$); ^{1}H NMR (400 MHz, $CDCl_3$) δ:7.70 (major isomer, d, *J* = 8.0 Hz, 2H), 7.66(minor isomer, d, *J* = 8.4 Hz, 0.8H)*, 7.53 (d, *J* = 5.2 Hz, 0.4H)*, 7.45 (d, *J* = 5.2 Hz, 1H), 7.26-7.25 (m, 2H), 7.26-7.25 (m, 0.8 Hz, 0.8H)*, 6.86 (d, *J* = 7.7 Hz, 0.4H)*, 6.83 (d, *J* = 8.0 Hz, 1H), 6.56 (d, *J* = 8.0 Hz, 1H), 6.52 (d, *J* = 7.6 Hz, 0.4H)*, 6.14 (dd, *J* = 5.2, 0.8 Hz, 1H), 6.09 (dd, *J* = 5.2, 0.8 Hz, 0.4H)*, 5.42 (m, 1H), 5.25 (s, 1H), 5.22 (s, 0.4H)*, 5.16 (m, 0.4H)*, 5.12 (s, 1H), 4.80 (s, 0.4 H)*, 3.83 (s, 1.2H)*, 3.81 (s, 1.2H)*, 3.77 (s, 3H), 3.73 (s, 3H), 3.65-3.45 (m, 3H), 3.65-3.45 (m, 1.2H)*, 3.24 (m, 1H), 3.19 (m, 0.4H)*, 2.39 (s, 3H), 2.39 (s, 1.2H)*; ^{13}C NMR (150 MHz, $CDCl_3$) δ:207.8*, 207.6, 159.9*, 158.1, 153.3*, 153.2, 147.3, 146.7*, 145.4*, 145.2, 143.3*, 143.2, 142.9*, 142.7, 137.3, 137.0*, 134.8, 133.5*, 129.7 (2C), 129.6 (2C)*, 127.0 (2C)*, 126.9 (2C), 117.3*, 117.2, 116.9*, 116.6, 115.2*, 113.6, 55.7*, 55.6, 53.6, 53.5*, 49.8*, 48.3, 47.5, 46.5*, 44.8, 44.8*, 21.5, 21.5*; IR (KBr): 3441, 1698, 1594, 1481, 1435, 1403, 1327, 1271 cm^{-1}; HRMS (ESI, m/z) calcd for $C_{22}H_{25}N_2O_5S[M+H]^+$: 429.1479, found 429.1492.

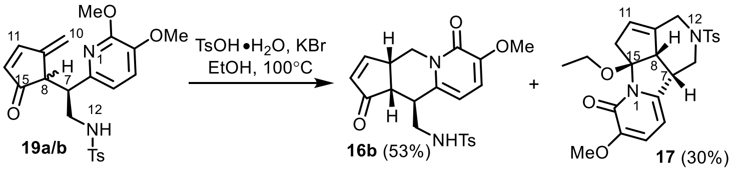

To a solution of **19a/b** (42.8 mg, 0.1 mmol, 1 equiv) in dry EtOH (3.6 ml) were added TsOH • H_2O (38.4 mg, 0.2 mmol, 2.0 equiv) and KBr (119 mg, 1.0 mmol, 10 equiv) at RT. After heating at 100℃ for 15 h, The resulting mixture was cooled to room temperature and filtered through Celite. The filtrate was concentrated *in vacuo* to afford crude product, which was purified

by column chromatography (DCM/MeOH, 100:1 → 40:1) to afford **16b** (21.9 mg, 53%) as a pale-yellow solid and **17** (13.3 mg, 30%) as a pale-yellow solid.

The structure of **16b** was confirmed by 2D NMR spectra analysis (DEPT-135, ^{1}H-^{1}H COSY, HSQC, HMBC, NOESY) and X-Ray Diffraction.

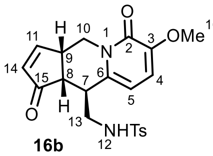

16b

Compound 16b: TLC (DCM:MeOH, 30:1 v/v):R_f = 0.5 (UV, $KMnO_4$); ^{1}H NMR (600 MHz, $CDCl_3$) δ: 7.74 (d, *J* = 8.5 Hz, 2H, **-Ts**), 7.54 (dd, *J* = 5.5, 2.5 Hz, 1H, **-H11**), 7.29 (d, *J* = 8.5 Hz, 2H, **-Ts**), 6.43 (d, *J* = 7.7 Hz, 1H, **-H4**), 6.17 (dd, *J* = 5.5, 2.7 Hz, 1H, **-H14**), 5.95 (d, *J* = 7.8 Hz, 1H, **-H5**), 5.89 (brs, 1H, **-N12H**), 4.95 (dd, *J* = 15.0, 2.4 Hz, 1H, **-H10**), 3.85 (dd, *J* = 14.4, 5.4 Hz, 1H, **-H10**), 3.74 (s, 3H, **-OMe**), 3.54 (m, 1H, **-H9**), 3.36 (m, 1H, **-H13**), 3.26 (m, 1H, **-H13**), 3.22 (m, 1H, **-H7**), 2.77 (dd, *J* = 6.7, 2.5 Hz, 1H, **-H8**), 2.42 (s, 3H, **-Ts**); ^{13}C NMR (150 MHz, $CDCl_3$) δ: 208.9 (**-C15**), 165.1 (**-C11**), 158.1 (**-C2**), 148.6 (**-C3**), 143.7 (**-Ts**), 137.3 (**-Ts**), 136.1 (**-C14**), 133.6 (**-C6**), 129.9 (2C, **-Ts**), 127.1 (2C, **-Ts**), 111.9 (**-C4**), 105.8 (**-C5**), 55.8 (**-OMe**), 44.6 (**-C13**), 44.0 (**-C8**), 42.1 (**-C7**), 40.7 (**-C9**), 39.9 (**-C10**), 21.8 (**-Ts**); IR (KBr): 3441, 1706, 1655, 1598, 1328, 1159, 897, 552 cm^{-1}; HRMS (ESI, m/z) calcd for $C_{21}H_{23}N_2O_5S[M+H]^+$: 415.1322, found 415.1332

The structure of **17** was confirmed by 2-D NMR spectra analysis (DEPT-135, ^{1}H-^{1}H COSY, HSQC, HMBC, NOESY)

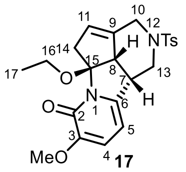

17

Compound 17: TLC (DCM:MeOH, 30:1 v/v):R_f = 0.45 (UV, $KMnO_4$); ^{1}H NMR (600 MHz, $CDCl_3$) δ: 7.64 (d, *J* = 8.5 Hz, 2H, **-Ts**), 7.33 (d, *J* = 8.5 Hz, 2H, **-Ts**), 6.59 (d, *J* = 7.3 Hz, 1H, **-H4**), 5.97 (dd, *J* = 7.3, 1.2 Hz, 1H, **-H5**), 5.55 (brs, 1H, **-H11**), 4.07 (d, *J* = 12.5 Hz, 1H, **-H10**), 3.88 (d, *J* = 18.1 Hz, 1H, **-H14**), 3.82 (dd, *J* = 9.1, 6.1 -Hz, 1H, **-H13**), 3.80 (s, 3H, **-OMe**), 3.59 (m, 2H, **-H16**, **-H7**), 3.43 (dt, *J* = 15.6, 7.2 Hz, 1H, **-H16**), 3.18 (m, 2H, **H8**, **-H10**), 3.05 (dd, *J* = 12.6, 4.2 Hz, 1H, **-H13**), 2.95 (d, *J* = 18.0 Hz, 1H, **-H14**), 2.44 (s, 3H, **-Ts**), 1.17 (t, *J* = 6.6 Hz, 3H **-H17**); ^{13}C NMR (150 MHz, $CDCl_3$) δ: 157.6 (**-C2**), 150.1 (**-C3**), 144.1 (**-Ts**), 139.7 (**-C6**), 133.6

(**-Ts**), 132.7 (**-C9**), 130.0 (2C, **-Ts**), 127.8 (2C, **-Ts**), 126.7 (**-C11**), 113.0 (**-C4**), 108.1 (**-C15**), 99.3 (**-C5**), 61.8 (**-C16**), 56,2 (**-OMe**), 51.2 (**-C8**), 47.1 (**-C10**), 44.9 (**-C13**), 40.3 (**-C7**), 40.1 (**-C14**), 21.8 (**-Ts**), 15.6 (**-C17**); IR (KBr): 2925, 1854, 1703, 1647, 1592, 1543, 1473, 1274, 1196 cm^{-1}; HRMS (ESI, m/z) calcd for $C_{23}H_{27}N_2O_5S[M+H]^+$: 443.1635, found 443.1643.

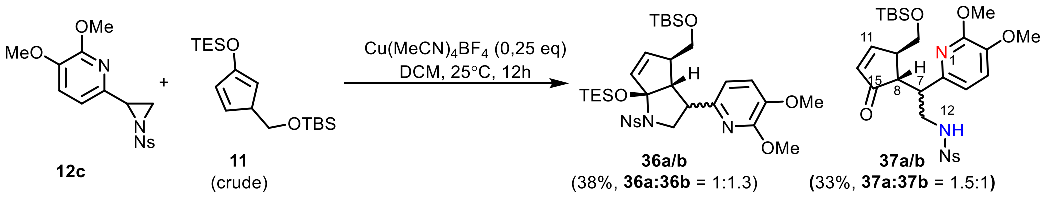

To a solution of **12c** (214 mg, 0.59 mmol, 1 equiv) and **11** (600 mg, 1.77 mmol, 3 equiv) in dry DCM (5.6 ml) was added $Cu(MeCN)_4BF_4$ (58mg, 0.15 mmol, 0.25 equiv) at RT. After stirring at RT for 12h, The reaction mixture was quenched with aq. $NaHCO_3$ (10 ml) and extracted with DCM (15 ml*3). The combined organic layers were washed with brine (12 mL), dried ($MgSO_4$), filtered and concentrated. The residue was purified by column chromatography (petroleum ether/EtOAc, 9:1→2:1) to afford an inseparable diastereomeric mixture of compound **36** (157 mg, 38%, **36a:36b** = 1:1.3) as a yellow oil, **37a** (69 mg, 20%) as a yellow solid and **37b** (45 mg, 13%) as a yellow solid.

Compound **36a/b** was separated by HPLC and the relative configuration was determined by 2D NMR spectra analysis (DEPT-135, ^1H-^1H COSY, HSQC, HMBC, NOESY)

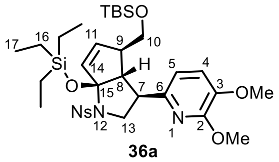

36a

Compound 36a: TLC (PE:EA, 9:1 v/v):R_f = 0.5 (UV, $KMnO_4$); ^1H NMR (600 MHz, $CDCl_3$) δ:8.29 (d, J = 8.4 Hz, 2H, **-Ns**), 8.04 (d, J = 8.4 Hz, 2H, **-Ns**), 6.88 (d, J = 7.8 Hz, 1H, **-H4**), 6.69 (d, J = 7.8 Hz, 1H, **-H5**), 6.41 (d, J = 5.4 Hz, 1H, **-H11**), 5. 89 (d, J = 5.4 Hz, 1H, **-H14**), 3.92 (s, 3H, **-OMe**), 3.84 (s, 3H, **-OMe**), 3.84 (m, 1H), **-H13**), 3.79 (m, 1H, **-H13**), 3.60 (m, 1H, **-H10**), 3.47 (m, 1H, **-H10**), 3.02 (m, 1H, **-H7**), 2.80 (m, 1H, **-H8**), 2.65 (m, 1H, **-H9**), 0.87 (t, J = 8.4 Hz, 9H, **-H17**), 0.76 (s, 9H, **-OTBS**), 0.59 (q, J = 8.4 Hz, 6H, **-H16**), -0.06 (s, 3H, **-OTBS**), -0.07 (s, 3H, **-OTBS**); ^{13}C NMR (150 MHz, $CDCl_3$) δ:153.8 (**-C2**), 149.7 (**-Ns**), 146.5 (**-Ns**), 145.9 (**-C6**), 143.0 (**-C3**), 134.3 (**-C14**), 134.2 (**-C11**), 128.8 (2C, **-Ns**), 123.8 (2C, **-Ns**), 117.3 (**-C4**), 115.7 (-

C5), 107.9 (-**C15**), 65.7 (-**C10**), 60.4 (-**C8**), 55.8 (-**OMe**), 54.2 (-**C9**), 54.1 (-**C13**), 53.4 (-**OMe**), 51.3 (-**C7**), 25.7 (3C, -**OTBS**), 18.1 (-**OTBS**), 6.8 (3C, -**C17**), 6.0 (3C, -**C16**), -5.5 (-**OTBS**), -5.6 (-**OTBS**); IR (KBr):2960, 1531, 1481, 1350, 1260, 1165, 1014, 838, 738; HRMS (ESI, m/z) calcd for $C_{33}H_{51}N_3NaO_8SSi_2[M+Na]^+$: 728.2833, found 728.2838.

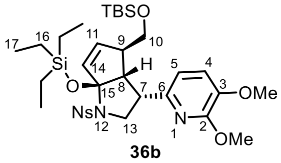

36b

Compound 36b: TLC (PE:EA, 9:1 v/v):R_f = 0.5 (UV, $KMnO_4$); ^{1}H NMR (600 MHz, $CDCl_3$) δ:8.31 (d, *J* = 9.0 Hz, 2H, -**Ns**), 8.17 (d, *J* = 9.0 Hz, 2H, -**Ns**), 6.96 (d, *J* = 7.8 Hz, 1H, -**H4**), 6.66 (d, *J* = 7.8 Hz, 1H, -**H5**), 6.29 (dd, *J* = 6.0, 2.4 Hz, 1H, -**H11**), 5.89 (d, *J* = 6.0 Hz, 1H, -**H14**), 4.13 (dd, *J* = 10.8, 10.8 Hz, 1H, -**H13**), 3.94 (s, 3H, -**OMe**), 3.85 (s, 3H, -**OMe**), 3.67 (m, 1H, -**H7**), 3.60 (m, 1H, -**H13**), 3.10 (dd, *J* = 9.6, 3.6 Hz, 1H, -**H10**), 2.96 (m, 1H, -**H9**), 2.82 (dd, *J* = 9.6, 7.8 Hz, 1H, -**H10**), 2.63 (m, 1H, -**H**8), 0.97 (t, *J* = 7.8 Hz, 9H, -**H17**), 0.78 (s, 9H, -**OTBS**), 0.67 (m, 6H, -**H16**), -0.14 (s, 3H, -**OTBS**), -0.17 (s, 3H, -**OTBS**); ^{13}C NMR (150 MHz, $CDCl_3$) δ: 153.6 (-**C2**), 149.7 (-**Ns**), 146.9 (-**Ns**), 144.2 (-**C6**), 143.0 (-**C3**), 135.5 (-**C14**), 134.7 (-**C11**), 128.7 (2C, -**Ns**), 123.7 (2C, -**Ns**), 117.5 (-**C4**), 115.4 (-**C5**), 107.1 (-**C15**), 64.6 (-**C10**), 59.4 (-**C8**), 55.8(-**OMe)**, 53.6 (-**OMe**), 51.1 (-**C13**), 45.6 (-**C9**), 42.5 (-**C7**), 25.8 (3C, -**OTBS**), 18.3 (-**OTBS**), 6.8 (3C, -**C17**), 5.9 (3C, -**C16**), -5.6 (2C, -**OTBS**); IR (KBr):2962, 1531, 1482, 1350, 1253, 1159, 1092, 1014, 839, 737, 618; HRMS (ESI, m/z) calcd for $C_{33}H_{51}N_3NaO_8SSi_2[M+Na]^+$: 728.2833, found 728.2838.

Compound 37a: TLC (PE:EA, 2:1 v/v):R_f = 0.5 (UV, $KMnO_4$); ^{1}H NMR (600 MHz, $CDCl_3$) δ:8.31 (d, J = 8.4 Hz, 2H), 7.97 (d, J = 8.4 Hz, 2H), 7.46 (d, J = 5.4 Hz, 1H), 6.88 (d, J = 7.8 Hz, 1H), 6.56 (d, J = 7.8 Hz, 1H), 6.03 (d, J = 5.4 Hz, 1H), 5.60 (t, J = 6.0 Hz, 1H), 3.89 (s, 3H), 3.82 (s, 3H), 3.64-3.61 (m, 2H), 3.49 (dd, J = 9.6, 5.4 Hz, 1H), 3.42 (dd, J = 9.6, 6.6 Hz, 1H), 3.20 (m, 1H), 3.03 (m, 1H), 2.46 (d, J = 7.2 Hz, 1H), 0.82 (s, 9H), -0.02 (s, 3H), -0.03 (s, 3H); ^{13}C NMR (150 MHz, $CDCl_3$) δ:210.2, 165.7, 153.6, 149.9, 146.3, 145.3, 143.2, 133.9, 128.1(2C), 124.2 (2C), 117.6, 117.3, 64.7, 55.7, 53.5, 49.8, 48.0, 45.7, 45.6, 25.8 (3C), 18.3, -5.5, -5.6; IR (KBr):2953, 1696, 1531, 1482, 1403, 1349, 1262, 1165, 838, 737; HRMS (ESI, m/z) calcd for

$C_{27}H_{37}N_3NaO_8SSi[M+Na]^+$: 614.1968, found 614.1962.

Compound 37b: TLC (PE:EA, 2:1 v/v):R_f = 0.45 (UV, $KMnO_4$); ^{1}H NMR (600 MHz, $CDCl_3$) δ:8.27 (d, J = 9.0 Hz, 2H), 7.92 (d, J = 9.0 Hz, 2H), 7.46 (dd, J = 6.0, 2.4 Hz, 1H), 6.87 (d, J = 7.8 Hz, 1H), 6.61 (d, J = 7.8 Hz, 1H), 6.16 (dd, J = 6.0, 1.8 Hz, 1H), 6.06 (t, J = 6.0 Hz, 1H), 3.81 (s, 3H), 3.78 (s, 3H), 3.59 (dd, J = 9.6, 6.0 Hz, 1H), 3.54-3.49 (m, 3H), 3.41 (m, 1H), 2.83 (m, 1H), 2.46 (dd, J = 4.2, 2.4 Hz, 1H), 0.85 (s, 9H), 0.00 (s, 6H); ^{13}C NMR (150 MHz, $CDCl_3$) δ:210.5, 164.6, 153.5, 149.7, 146.4, 145.5, 143.0, 134.9, 128.0 (2C), 124.2 (2C), 117.4, 116.1, 64.2, 55.7, 53.7, 49.8, 48.6, 46.6, 45.7, 25.8(3C), 18.2, -5.5, -5.6; IR (KBr):2962, 1700, 1531, 1403, 1349, 1262, 109, 838; HRMS (ESI, m/z) calcd for $C_{27}H_{37}N_3NaO_8SSi[M+Na]^+$: 614.1968, found 614.1962.

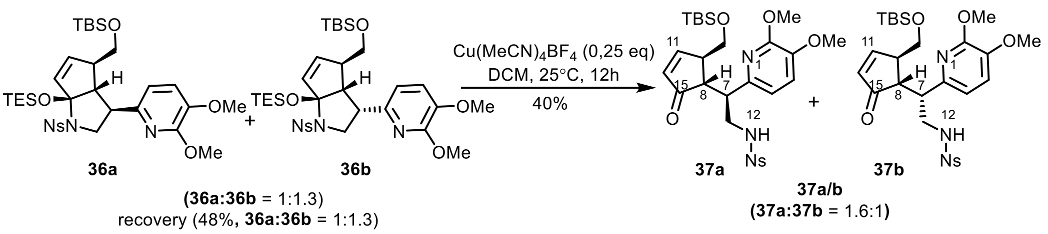

To a solution of **36a/b** (36 mg, 0.05 mmol, 1 equiv, **36a:36b** = 1:1.3) in dry DCM (1 ml) were added $Cu(MeCN)_4BF_4$ (4 mg, 0.013 mmol, 0.25 equiv) at RT, After stirring at RT for 12 h, The reaction mixture was quenched with aq. $NaHCO_3$ (3 ml) and extracted with DCM (5 ml*3). The combined organic layers were washed with brine (10 mL), dried ($MgSO_4$), filtered and concentrated. The residue was purified by column chromatography (petroleum ether/EtOAc, 2:1) to afford compound **37a/b** (12 mg, 40%, **37a:37b** = 1.6:1) as a yellow solid and recover the compound **36a/b** (17.2 mg, 48%, **36a:36b** = 1.3:1) as a yellow oil.

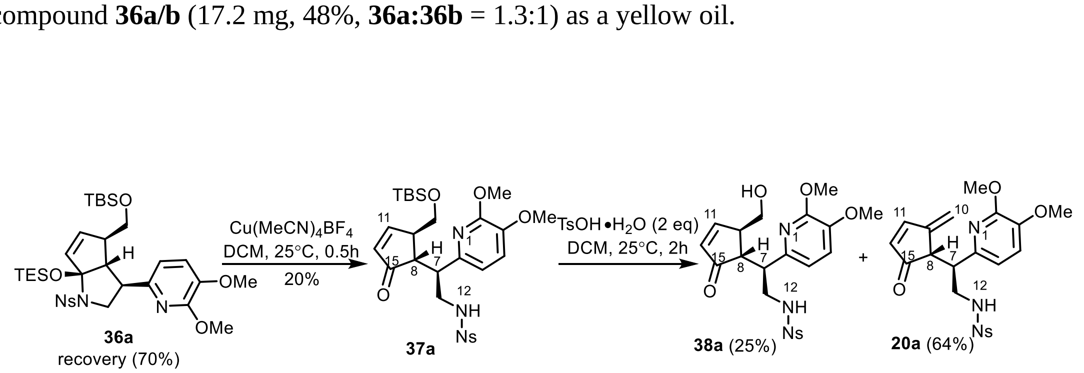

To a solution of **36a** (5 mg, 0.007 mmol, 1 equiv) in dry DCM (0.4 ml) were added $Cu(MeCN)_4BF_4$ (1.6 mg, 0.005 mol, 0.7 equiv) at RT, After stirring at RT for 0.5 h, The reaction

mixture was quenched with aq. $NaHCO_3$ (0.5 ml) and extracted with DCM (2 ml*3). The combined organic layers were washed with brine (3 mL), dried ($MgSO_4$), filtered and concentrated. The residue was purified by column chromatography (petroleum ether/EtOAc, 10:1→ 2:1) to afford compound **37a** (0.8 mg, 19%) as a yellow solid and recover the product **36a** (3.5 mg, 70%) as a yellow oil.

To a solution of **37a** (10 mg, 0.017 mmol, 1 equiv) in dry DCM (1 ml) were added TsOH • H_2O (6.5 mg, 0.034 mmol, 2 equiv) at RT, After stirring at RT for 2 h, The reaction mixture was quenched with aq. $NaHCO_3$ (2 ml) and extracted with DCM (3 ml*3). The combined organic layers were washed with brine (5 mL), dried ($MgSO_4$), filtered and concentrated. The residue was purified by column chromatography (DCM/EtOAc, 10:1→ DCM/MeOH, 30:1) to afford compound **38a** (2 mg, 25%) as a yellow solid and compound **20a** (5 mg, 64%) as a yellow solid.

Compound 38a: TLC (PE:EA, 1:2 v/v):R_f = 0.3 (UV, $KMnO_4$); ^{1}H NMR (600 MHz, $CDCl_3$) δ:8.34 (d, J = 9.0 Hz, 2H), 8.00 (d, 9.0 Hz, 2H), 7.45 (dd, J = 6.0, 2.4 Hz, 1H), 6.88 (d, J = 7.8 Hz, 1H), 6.58 (d, J = 7.8 Hz, 1H), 6.06 (dd, J = 6.0, 1.2 Hz, 1H), 6.01 (t, J = 6.0 Hz, 1H), 3.88 (s, 3H), 3.82 (s, 3H), 3.70-3.62 (m, 2H), 3.54 (m, 1H), 3.32 (m, 1H), 3.24 (m, 1H), 2.62 (dd, J = 6.0, 1.2 Hz, 1H), 2.07 (m, 1H); ^{13}C NMR (150 MHz, $CDCl_3$) δ: 210.2, 164.6, 153.5, 149.9, 146.1, 145.1, 143.1, 134.6, 128.1 (2C), 124.3 (2C), 117.5, 117.2, 64.5, 55.7, 53.6, 50.1, 47.1, 45.5, 45.4; IR (KBr):3424, 2925, 1697, 1531, 1482, 1403, 1350, 1163, 1264; HRMS (ESI, m/z) calcd for $C_{21}H_{23}N_3NaO_8S$ $[M+Na]^+$: 500.1104, found 500.1103.

Compound 20a: TLC (DCM/EA, 10:1 v/v):R_f = 0.6 (UV, $KMnO_4$); ^{1}H NMR (600 MHz, $CDCl_3$) δ:8.31 (d, J = 8.4 Hz, 2H), 7.98 (d, J = 8.4 Hz, 2H), 7.51 (d, J = 5.4 Hz, 1H), 6.84 (d, J = 7.8 Hz, 1H), 6.57 (d, J = 7.8 Hz, 1H), 6.20 (d, J = 5.4 Hz, 1H), 5.94 (t, J = 6.0 Hz, 1H), 5.30 (s, 1H), 5.18 (s, 1H), 3.79 (s, 3H), 3.75 (s, 3H), 3.64-3.58 (m, 2H), 3.56 (m, 1H, H7), 3.26 (d, J = 4.8 Hz, 1H, H8); ^{13}C NMR (150 MHz, $CDCl_3$) δ:207.7, 158.4, 153.3, 149.8, 147.0, 146.3, 144.7, 142.9, 134.8, 128.0 (2C), 124.3 (2C), 117.1, 116.4, 113.8, 55.6, 53.7, 48.6, 47.2, 45.5; IR (KBr):2928, 1703, 1530, 1482, 1350, 1164, 668; HRMS (ESI, m/z) calcd for $C_{21}H_{22}N_3O_7S[M+H]^+$: 460.1173, found 460.1181.

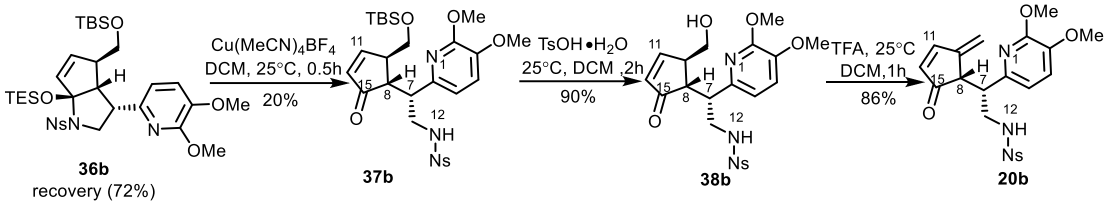

To a solution of **36b** (5 mg, 0.007 mmol, 1 equiv) in dry DCM (0.4 ml) were added $Cu(MeCN)_4BF_4$ (1.6 mg, 0.005 mol, 0.7 equiv) at RT, After stirring at RT for 0.5 h, The reaction mixture was quenched with aq. $NaHCO_3$ (2 ml) and extracted with DCM (3 ml*3). The combined organic layers were washed with brine (5 mL), dried ($MgSO_4$), filtered and concentrated. The residue was purified by column chromatography (petroleum ether/EtOAc, 10:1→ 2:1) to afford compound **37b** (0.8 mg, 20%) as a yellow solid and recover the product **36b** (3.6 mg, 72%) as a yellow oil.

To a solution of **37b** (10 mg, 0.017 mmol, 1 equiv) in dry DCM (1 ml) were added TsOH • H_2O (6.5 mg, 0.034 mmol, 2 equiv) at RT, After stirring at RT for 2 h, The reaction mixture was quenched with aq. $NaHCO_3$ (2 ml) and extracted with DCM (3 ml*3). The combined organic layers were washed with brine (5 mL), dried ($MgSO_4$), filtered and concentrated. The residue was purified by column chromatography (DCM/MeOH, 30:1) to afford **38b** (7.2 mg, 90%) as a yellow solid.

To a solution of **38b** (7.2 mg, 0.015 mmol, 1 equiv) in dry DCM (1 ml) was added TFA (2.2 ul, 0.03mmol, 2 equiv) at RT, After stirring at RT for 1 h, The reaction mixture was quenched with aq. $NaHCO_3$ (2 ml) and extracted with DCM (3 ml*3). The combined organic layers were washed with brine (5 mL), dried ($MgSO_4$), filtered and concentrated. The residue was purified by column chromatography (DCM/EtOAc, 10:1) to afford **20b** (6 mg, 86%) as a yellow solid

Compound 38b: TLC (PE:EA, 1:2 v/v):R_f = 0.25 (UV, $KMnO_4$); ^1H NMR (600 MHz, $CDCl_3$) δ:8.28 (d, J = 8.4 Hz, 2H), 7.95 (d, J = 8.4 Hz, 2H), 7.49 (dd, J = 6.0, 2.4 Hz, 1H), 6.88 (d, J = 7.8 Hz, 1H), 6.64 (d, J = 7.8 Hz, 1H), 6.20 (dd, J = 6.0, 1.8 Hz, 1H), 6.02 (t, J = 6.0 Hz, 1H), 3.81 (s, 3H), 3.79 (s, 3H), 3.69 (dd, J = 10.2, 6.0 Hz, 1H), 3.62 (dd, J = 10.2, 6.0 Hz, 1H), 3.56-3.51 (m, 2H), 3.43 (m, 1H), 2.88 (m, 1H), 2.56 (dd, J = 4.8, 3.0 Hz, 1H); ^{13}C NMR (150 MHz, $CDCl_3$) δ:210.2, 163.7, 153.6, 149.8, 146.2, 145.3, 143.2, 135.4, 128.0 (2C), 124.2 (2C), 117.4, 116.2, 63.7, 55.7, 53.8, 49.3, 48.3, 46.6, 45.5; HRMS (ESI, m/z) calcd for $C_{21}H_{23}N_3NaO_8S[M+Na]^+$: 500.1104, found 500.1103.

Compound 20b: TLC (DCM/EA, 10:1 v/v):R_f = 0.55 (UV, $KMnO_4$); ^{1}H NMR (600 MHz, $CDCl_3$) δ:8.31 (d, J = 9.0 Hz, 2H), 7.97 (d, J = 9.0 Hz, 2H), 7.60 (d, J = 5.4 Hz, 1H), 6.90 (d, J = 7.8 Hz, 1H), 6.57 (d, J = 7.8 Hz, 1H), 6.16 (dd, J = 5.4, 1.2 Hz, 1H), 5.53 (t, J = 6.0 Hz, 1H), 5.21 (s, 1H), 4.59 (s, 1H), 3.91 (s, 3H), 3.85 (s, 3H), 3.70-3.62 (m, 2H), 3.31 (d, J = 7.8 Hz, 1H, H8), 3.08 (m ,1H, H7); ^{13}C NMR (150 MHz, $CDCl_3$) δ:206.7, 159.0, 152.5 148.9, 145.7, 145.1, 144.5, 142.2, 132.3, 127.1 (2C), 123.2 (2C), 116.4, 116.1, 114.3, 54.7, 52.7, 48.3, 45.6, 44.2; IR (KBr):2926, 1697, 1531, 1482, 1350, 1263, 1165, 1096, 1012, 823, 707; HRMS (ESI, m/z) calcd for $C_{21}H_{22}N_3O_7S[M+H]^+$: 460.1173, found 460.1181.

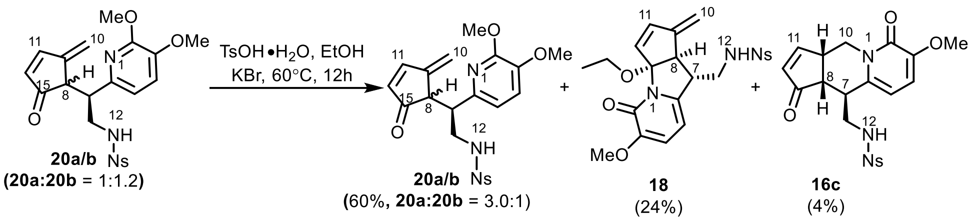

To a solution of **20a/b** (50 mg, 0.11 mmol, 1 equiv) in dry EtOH (3.9 ml) were added TsOH • H_2O (42 mg, 0.22 mmol, 2.0 equiv) and KBr (130 mg, 1.1 mmol, 10 equiv) at RT. After heating at 60℃ for 12 h, The resulting mixture was cooled to room temperature and filtered through Celite. The filtrate was concentrated *in vacuo* to afford crude product, which was purified by column chromatography (PE/EA/DCM = 2:1:1→ DCM/MeOH = 100:1→40:1) to afford **20a/b** (30 mg, 60%, **20a:20b** = 3.0:1) as a pale-yellow solid, **18** (11.6 mg, 24%) as a pale-yellow solid and **16c** (1.9 mg, 4%) as a pale-yellow solid.

Compound **18** was separated by HPLC and the relative configuration was determined by 2D NMR spectra analysis (DEPT-135, ^{1}H-^{1}H COSY, HSQC, HMBC, NOESY)

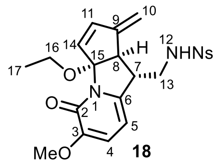

Compound 18: TLC (DCM/MeOH, 30:1 v/v):R_f = 0.7 (UV, $KMnO_4$); ^{1}H NMR (600 MHz, $CDCl_3$) δ:8.28 (d, *J* = 9.0 Hz, 2H, **-Ns**), 7.90 (d, *J* = 9.0 Hz, 2H, **-Ns**), 7.10 (d, *J* = 6.0 Hz, 1H, **-H14**), 6.38 (d, *J* = 6.0 Hz, 1H, **-H11**), 6.28 (d, *J* = 7.2 Hz, 1H, **-H4**), 6.12 (m, 1H, **-H**Ns), 5.65 (d, *J* = 7.2 Hz, 1H, **-H5**), 5.18 (d, J = 1.8 Hz, 1H, **-H10**), 4.99 (s, 1H, **-H10**), 4.12 (m, 1H, **-H16**), 3.87

(m, 1H, **-H16**), 3.72 (s, 3H, **-OMe**), 3.48 (m, 1H, **-H13**), 3.42 (m, 1H, **-H13**), 3.36 (m, 1H, **-H7**), 3.17 (m, 1H, **-H8**), 1.32 (t, *J* = 6.6 Hz, 3H, **-H17**); ^{13}C NMR (150 MHz, $CDCl_3$) δ:156.4 (**-C2**), 150.0 (**-Ns**), 149.8 (**-C6**), 149.6 (**-C9**), 146.0 (**-Ns**), 139.3 (**-C3**), 138.4 (**-C11**), 132.5 (**-C14**), 128.0 (2C, **-Ns**), 124.3 (2C, **-Ns**), 112.7 (**-C4**), 109.4 (**-C15**), 109.3 (**-C10**), 99.8 (**-C5**), 63.5 (**-C16**), 56.0 (**-OMe**), 54.5 (**-C8**), 47.7 (**-C13**), 44.9 (**-C7**), 14.6 (**-C17**); IR (KBr):3439, 2927, 1654, 1529, 1597, 1458, 1385, 1350, 1217, 1164; HRMS (ESI, m/z) calcd for $C_{22}H_{23}N_3NaO_7S[M+Na]^+$: 496.1154, found 496.1167.

Compound 18: ^{1}H NMR (600 MHz, CD_2Cl_2) δ:8.29 (d, J = 9.0 Hz, 2H), 7.92 (d, J = 9.0 Hz, 2H), 7.00 (d, J = 6.0 Hz, 1H), 6.40 (d, J = 6.0 Hz, 1H), 6.33 (d, J = 7.8 Hz, 1H), 5.94 (m, 1H), 5.68 (d, J = 7.8 Hz, 1H), 5.18 (d, J = 2.4 Hz, 1H), 5.02 (s, 1H), 4.01 (m, 1H), 3.73 (m, 1H), 3.68 (s, 3H), 3.45 (m, 1H), 3.39 (m, 1H), 3.34 (m, 1H), 3.23 (m, 1H), 1.29 (t, J = 6.6 Hz, 3H); ^{13}C NMR (150 MHz, CD_2Cl_2) δ:156.4, 150.6, 150.3, 150.2, 146.3, 140.0, 138.6, 133.3, 128.5 (2C), 124.7 (2C), 113.3, 109.9, 109.3, 100.0, 63.0, 56.3, 53.5, 47.9, 45.7, 15.7.

Compound 16c: TLC (DCM/MeOH, 30:1 v/v):R_f = 0.4 (UV, $KMnO_4$); ^{1}H NMR (600 MHz, $CDCl_3$) δ: 8.31 (d, *J* = 9.2 Hz, 2H), 8.08 (d, *J* = 9.2 Hz, 2H), 7.47 (dd, *J* = 5.9, 2.9 Hz, 1H), 7.08 (brs, 1H), 6.40 (d, *J* = 7.2 Hz, 1H), 6.17 (dd, *J* = 6.0, 2.4 Hz, 1H), 6.03 (d, *J* = 7.8 Hz, 1H), 4.95 (dd, *J* = 14.4, 1.2 Hz, 1H), 3.84 (dd, *J* = 14.4, 5.4 Hz, 1H), 3.73 (s, 3H), 3.53 (m, 1H), 3.41-3.35 (m, 3H), 2.64 (dd, *J* = 6.6, 1.2 Hz, 1H); ^{13}C NMR (150 MHz, $CDCl_3$) δ: 208.0, 164.5, 157.7, 149.9, 148.1, 146.6, 136.2, 133.1, 128.3 (2C), 124.3 (2C), 111.7, 106.9, 55.5, 44.4, 44.2, 42.7, 40.2, 39.7; IR (KBr): 2924, 2853, 1708, 1654, 1595, 1530, 1467, 1350, 1164 cm^{-1}; HRMS (ESI, m/z) calcd for $C_{20}H_{20}N_3O_7S[M+H]^+$: 446.1016, found 446.1025.

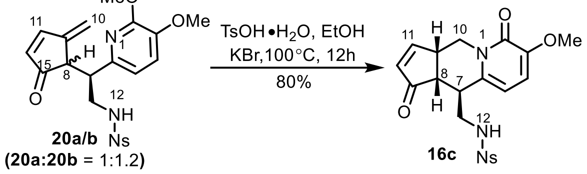

To a solution of **20a/b** (50 mg, 0.11 mmol, 1 equiv) in dry EtOH (3.9 ml) were added TsOH • H_2O (42 mg, 0.22 mmol, 2.0 equiv) and KBr (130 mg, 1.1 mmol, 10 equiv) at RT. After heating at 100℃ for 12 h, The resulting mixture was cooled to room temperature and filtered through Celite. The filtrate was concentrated *in vacuo* to afford crude product, which was purified

by column chromatography (DCM/MeOH = 100:1→40:1) to afford **16c** (39 mg, 80%) as a pale-yellow solid.

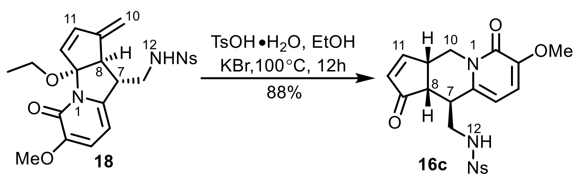

To a solution of **18** (10 mg, 0.022 mmol, 1 equiv) in dry EtOH (3.9 ml) were added TsOH • H_2O (8.6 mg, 0.45 mmol, 2.0 equiv) and KBr (26.7 mg, 0.22 mmol, 10 equiv) at RT. After heating at 100℃ for 2 h, The resulting mixture was cooled to room temperature and filtered through Celite. The filtrate was concentrated *in vacuo* to afford crude product, which was purified by column chromatography (DCM/MeOH = 100:1→40:1) to afford **16c** (8.8 mg, 88%) as a pale-yellow solid.

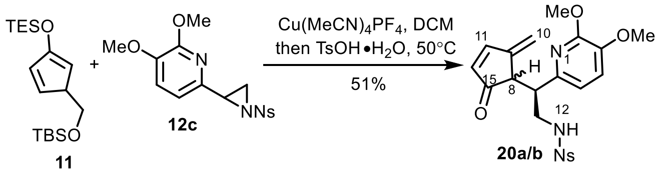

To a solution of **12c** (36.5 mg, 0.1 mmol, 1 equiv) and crude **11** (102 mg, 0.3 mmol, 3 equiv) in dry DCM (1.0 ml) was added $Cu(MeCN)_4BF_4$ (6.3 mg, 0.02 mmol, 0.2 equiv) at RT. After stirring at RT for 12h, TsOH • H_2O (38 mg, 0.2 mmol, 2 equiv) was added to the reaction system. The resulting mixture was warmed to 50℃ and stirred at this temperature for 2h. The reaction mixture was quenched with aq. $NaHCO_3$ (2 ml) and extracted with DCM (3 ml*3). The combined organic layers were washed with brine (4 mL), dried ($MgSO_4$), filtered and concentrated. The residue was purified by column chromatography (petroleum ether/EtOAc/DCM, 8:2:3 → 6:3:3) to afford an unseparable diastereomeric mixture of compound **20a/b** (23.4 mg, 51%, **20a:20b** = 1 :1.7) as a pale-yellew solid.

Compound 20a/b: TLC (PE:EA:DCM, 2:1:2 v/v):R_f = 0.5 (UV, $KMnO_4$); ^1H NMR (600 MHz, $CDCl_3$) δ:8.30 (major isomer, d, *J* = 9.2 Hz, 2H), 8.29 (minor isomer, d, *J* = 9.2 Hz, 1.2H)*, 7.98 (d, *J* = 9.2 Hz, 1.2H)*, 7.96 (d, *J* = 9.2 Hz, 2H), 7.58 (d, *J* = 5.6 Hz, 1H), 7.50 (d, *J* = 6.0 Hz, 0.6H)* 6.88 (d, *J* = 7.8 Hz, 1H), 6.83 (d, *J* = 7.8 Hz, 0.6H)*, 6.56 (d, *J* = 7.8 Hz, 0.6H)*, 6.55 (d, *J*

= 7.8 Hz, 1H), 6.20 (dd, J = 5.6, 1.2 Hz, 0.6H)*, 6.14 (dd, J = 5.4, 1.2 Hz, 1H), 5.99 (t, J = 6.0 Hz, 0.6H)*, 5.61 (t , J = 6.0 Hz, 1H), 5.30 (s, 0.6H)*, 5.21 (s, 1H), 5.17 (s, 0.6 H)*, 4.62 (s, 1H), 3.90 (s, 3H), 3.83 (s, 3H), 3.78 (s, 1.8 H)*, 3.74 (s, 1.8 H)*, 3.69-3.69 (m, 2H), 3.69-3.59 (m, 1.2 H)*, 3.57 (m, 0.6 H)*, 3.30 (d, J = 7.8 Hz, 1H), 3.25 (d, J = 4.8 Hz, 0.6 H)*, 2.90 (m, 1H); ^{13}C NMR (150 MHz, $CDCl_3$) δ:207.8, 207.8*, 160.1, 158.4*, 153.4, 153.2*, 149.9, 149.8*, 147.0*, 146.7, 146.3* 146.1, 145.4, 144.7*, 143.2, 142.9*, 134.8*, 133.4, 128.2 (2C), 128.0 (2C)*, 124.3 (2C)*, 124.2 (2C), 117.3, 117.2*, 117.1, 116.5*, 115.3, 113.9*, 55.7, 55.6*, 53.7*, 53.6, 49.3, 48.6*, 47.2*, 46.6, 45.4*, 45.2; IR (KBr): 2922, 1851, 1699, 1530, 1481, 1463, 1436, 1403, 1350, 1164 cm^{-1}; HRMS (ESI, m/z) calcd for $C_{21}H_{22}N_3O_7S[M+H]^+$: 460.1173, found 460.1181.

Operation of one pot synthesis of compound 16c

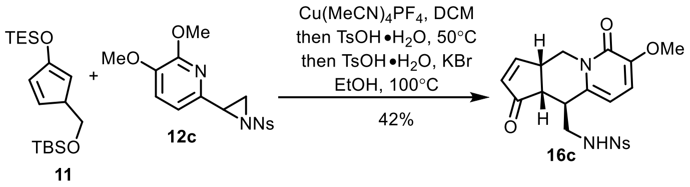

To a solution of **12c** (4.0 g, 11.0 mmol, 1 equiv) and crude **11** (11.2 g, 33.0 mmol, 3 equiv) in dry DCM (110 ml) was added $Cu(MeCN)_4BF_4$ (691 mg, 2.2 mmol, 0.2 equiv) at RT. After stirring at RT for 12h, TsOH • H_2O (4.2 g, 22 mmol, 2 equiv) was added to the reaction system. The resulting mixture was warmed to 50℃ and stirred at this temperature for 2h. The reaction mixture was concentrated *in vacuo* to afford crude product, which was redissolved in EtOH (250 ml). TsOH • H_2O (4.2 g, 22 mmol, 2 equiv) and KBr (13.2 g, 110 mmol, 10 eq) was added to the reaction system. After heating at 100℃ for 20 h, The resulting mixture was cooled to room temperature and filtered through Celite. The filtrate was concentrated *in vacuo* to afford crude product. The crude product was redissolved in DCM (1000 ml) and washed with H_2O (300 ml) and brine (300 ml), dried ($MgSO_4$), filtered and concentrated. The residue was purified by column chromatography (DCM/MeOH, 100:1 → 40:1) to afford **16c** (2.05 g, 42%) as a yellow solid.

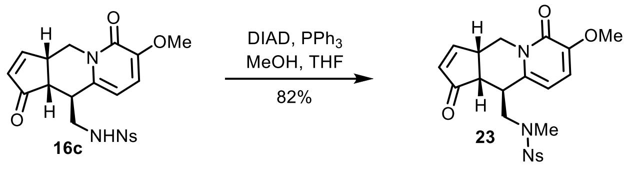

To a solution of **16c** (2.0 g, 4.5 mmol, 1 equiv), PPh_3 (3.53g, 13.5 mmol, 3 equiv) and MeOH (0.55 ml, 13.5 mmol, 3 equiv) in dry THF (200 ml) was added DIAD (2.67 ml, 13.5 mmol, 3 equiv) at RT. After stirring at RT for 4h, the reaction mixture was quenched with sat. aq. $NaHCO_3$ (100 ml) and extracted with EtOAc (300 ml*3). The combined layers were washed with bring (200 ml), dried ($MgSO_4$), filtered and concentrated. The residue was purified by column chromatography (DCM/MeOH, 100:1 → 50:1) to afford **23** (1.69 g, 82%) as a pale-yellow solid.

Compound 23: TLC (DCM:MeOH, 30:1 v/v):R_f = 0.7 (UV, $KMnO_4$); ^{1}H NMR (600 MHz, $CDCl_3$) δ: 8.35 (d, J = 8.4 Hz, 2H), 7.93 (d, J = 8.4 Hz, 2H), 7.60 (dd, J = 6.0, 1.8 Hz, 1H), 6.43 (d, J = 7.8 Hz, 1H), 6.19 (d, J = 5.4 Hz, 1H), 5.88 (d, J = 7.8 Hz, 1H), 5.37 (d, J = 12.6 Hz, 1H), 3.78 (m, 1H), 3.75 (m, 1H), 3.74 (s, 3H), 3.69 (dd, J = 13.8, 10.6 Hz, 1H), 3.36 (t, J = 8.4 Hz, 1H), 3.02 (dd, J = 13.8, 7.2 Hz, 1H), 2.93 (d, J = 6.6 Hz, 1H), 2.91 (s, 3H); ^{13}C NMR (150 MHz, $CDCl_3$) δ: 209.2, 165.7, 158.3, 150.3, 149.1, 143.6, 136.1, 133.0, 128.4 (2C), 124.7 (2C), 111.8, 105.3, 55.9, 52.1, 42.9, 40.5, 40.2, 39.8, 35.0; IR (KBr): 2926, 1706, 1656, 1603, 1531, 1350, 1312, 1215, 1254, 1165 cm^{-1}; HRMS (ESI, m/z) calcd for $C_{21}H_{22}N_3O_7S[M+H]^+$: 460.1173, found 460.1178.

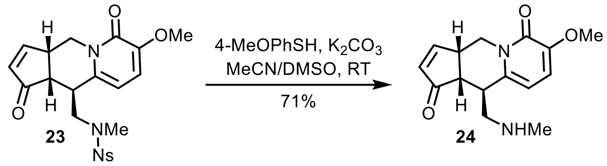

To a solution of **23** (1.6 g, 3.49 mmol, 1 equiv) in dry MeCN/DMSO (80/1.6 ml) was added K_2CO_3 (963 mg, 6.98 mmol, 2 equiv) and 4-MeOPhSH (2.6 ml, 21 mmol, 6 equiv) at RT. After stirring at RT for 12h, the reaction mixture was quenched with sat. aq. $NaHCO_3$ (50 ml) and extracted with EtOAc (100 ml*3). The combined layers were washed with bring (100 ml), dried ($MgSO_4$), filtered and concentrated. The residue was purified by column chromatography (DCM/MeOH/NH_4OH, 100:2.5:1 → 100:5:1) to afford **24** (678 mg, 71%) as a pale-yellow oil.

Compound 24: TLC (DCM:MeOH:NH_4OH, 100:5:1 v/v):R_f = 0.4 (UV, $KMnO_4$); ^{1}H NMR (600 MHz, $CDCl_3$) δ: 7.54 (dd, J = 5.9, 2.9 Hz, 1H), 6.51 (d, J = 7.9 Hz, 1H), 6.17 (dd, J = 6.0, 2.4 Hz, 1H), 5.94 (d, J =7.9 Hz, 1H), 5.13 (dd, J = 14.5, 2.5 Hz, 1H), 3.82 (dd, J = 14.4, 6.0 Hz, 1H), 3.76 (s, 3H), 3.57 (m, 1H), 3.21 (td, J = 7.8, 2.4 Hz, 1H), 2.91 (dd, J = 8.4, 3.6 Hz, 2H), 2.80 (dd, J = 6.6, 2.4 Hz, 1H), 2.48 (s, 3H); ^{13}C NMR (150 MHz, $CDCl_3$) δ: 209.5, 164.9, 158.3, 148.4, 136.2,

135.3, 112.1, 104.7, 55.8, 53.7, 44.5, 41.8, 40.8, 39.7, 36.2; IR (KBr): 3449, 1702, 1654, 1595, 1561, 1477, 1254, 1221, 1051, 772 cm^{-1}; HRMS (ESI, m/z) calcd for $C_{15}H_{19}N_2O_3[M+H]^+$: 275.1390, found 275.1402.

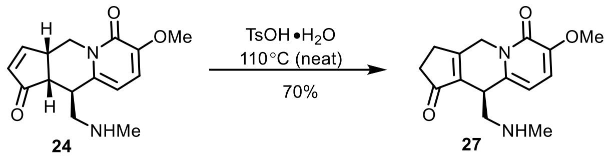

To a solution of **24** (700 mg, 2.55 mmol, 1 equiv) in 2,2,2-trifluoroethanol (5 ml) was added TsOH·H_2O (4.9 g, 25.5 mmol, 10 equiv) at RT. The reaction mixture was warmed to 110°C (TFE was completely evaporated in this process). After stirring at 110°C for 8h, The resulting mixture was cooled to room temperature and filtered through Celite. The filtrate was concentrated *in vacuo* to afford crude product, which was purified by column chromatography ($CHCl_3$/MeOH/NH_4OH, 100:2:1 → 100:10:1) to afford **27** (490 mg, 70%) as a pale-yellew oil.

Compound 27:TLC (DCM:MeOH:NH_4OH, 100:5:1 v/v):R_f = 0.5 (UV, $KMnO_4$); ^1H NMR (600 MHz, $CDCl_3$) δ: 6.69 (d, J = 7.8 Hz, 1H), 6.16 (d, J = 7.8 Hz, 1H), 5.11 (d, J = 20.4 Hz, 1H), 4.62 (d, J = 20.4 Hz, 1H), 3.88 (m, 1H), 3.83 (s, 3H), 2.82 (dd, J = 18.0, 7.8 Hz, 1H), 2.78 (d, J = 5.4 Hz, 2H), 2.67 (dd, J = 18.0, 6.6 Hz, 1H), 2.60 (m, 1H), 2.54 (m, 1H), 2.33 (s, 3H); ^{13}C NMR (150 MHz, $CDCl_3$) δ: 205.7, 165.9, 158.6, 148.0, 136.4, 135.4, 112.6, 104.6, 56.8, 55.9, 46.7, 36.8, 36.4, 34.8, 27.6; IR (KBr): 3443, 1705, 1651, 1597, 1564, 1461, 1251, 1208, 1052 cm^{-1}; HRMS (ESI, m/z) calcd for $C_{15}H_{19}N_2O_3[M+H]^+$: 275.1390, found 275.1404.

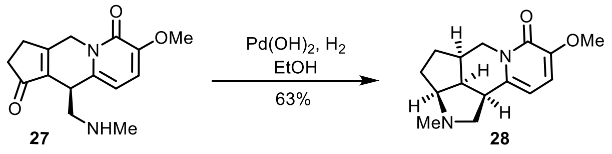

To a solution of **27** (15 mg, 0.055 mmol, 1.0 equiv) in EtOH (1.5 mL) was added $Pd(OH)_2$ (10.2 mg, 15 wt. %) at RT. The reaction mixture was bubbled with H_2 at room temperature for five minutes. After stirring under a balloon with H_2 for 12 h, The reaction was filtered through Celite. The filtrate was concentrated *in vacuo* to afford crude product which was purified by column chromatography ($CHCl_3$/MeOH/NH_4OH, 100:2:1 → 100:5:1) to afford **28** (9 mg, 63%) as a white solid.

Compound 28:TLC (DCM:MeOH:NH_4OH, 100:2:1 v/v):R_f = 0.5 (UV, $KMnO_4$); ^{1}H NMR (600 MHz, CD_3OD) δ:6.91 (d, *J* = 7.8 Hz, 1H), 6.35 (dd, *J* = 7.8, 0.6 Hz, 1H), 4.25 (dd, *J* = 13.8, 4.8 Hz, 1H), 4.11 (dd, *J* = 13.8, 8.4 Hz, 1H), 3.80 (s, 3H), 3.39 (m, 1H), 3.18 (m, 1H), 3.09 (dd, *J* = 10.2, 4.2 Hz, 1H), 2.98 (m, 1H), 2.76 (dd, J = 10.2, 7.2 Hz, 1H), 2.37 (m, 1H), 2.32 (s, 3H), 1.75-1.65 (m, 3H), 1.40 (m, 1H); ^{13}C NMR (150 MHz, CD_3OD) δ: 158.9, 147.1, 138.3, 114.0, 104.3, 72.3, 60.6, 55.0, 48.1, 45.3, 42.2, 39.1, 37.2, 28.2, 27.7; IR (KBr): 2942, 2777, 1651, 1596, 1560, 1470, 1247, 1058, 805; HRMS (ESI, m/z) calcd for $C_{15}H_{21}N_2O_2[M+H]^+$: 261.1598, found 261.1607.

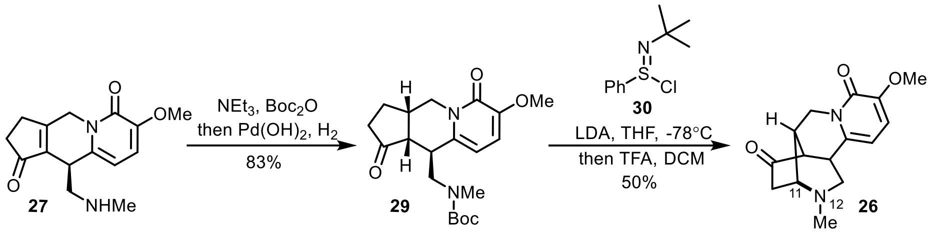

To a solution of **27** (900 mg, 3.28 mmol, 1.0 equiv) in dry DCM (90 ml) was added NEt_3 (1.37 ml, 9.85 mmol, 3.0 equiv) and Boc_2O (1.43 g, 6.57 mmol, 2.0 equiv) at RT. After stirring at RT for 2h, the reaction mixture was concentrated *in vacuo* to afford crude product. To a solution of the crude product obtained above in EtOH (90 ml) was added $Pd(OH)_2$ (610 mg, 15 wt. %) at RT. The reaction mixture was bubbled with H_2 at room temperature for ten minutes. After stirring under a balloon with H_2 for 5 h, The reaction was filtered through Celite. The filtrate was concentrated *in vacuo* to afford crude product which was purified by column chromatography ($CHCl_3$/MeOH/NH_4OH, 100:2:1→100:5:1) to afford **29** (1.02 g, 83%) as a white solid.

To a solution of **29** (1.02 g, 2.71 mmol, 1.0 equiv) in dry THF (35 ml) was added LDA (2.05 ml, 2 M in THF/heptane, 4.1 mmol, 1.5 equiv) at -78℃, After stirring at same temperature for 0.5 h, the solution of **30**[5] (1.75 g, 8.1 mmol, 3.0 equiv) in dry THF (35 ml) was added at -78℃. After stirring at the same temperature for 1 h, the reaction mixture was quenched with sat. aq. $NaHCO_3$ and extracted with EtOAc (3 x 90 mL). The combined organic layers were washed with brine, dried ($MgSO_4$), filtered, and concentrated to afford crude product. To a solution of the crude product obtained above in dry TFA/DCM (33 ml, 1:10) at RT and stirred at this temperature for 1 h. Removal of the solvent under reduced pressure, purified the residue by column chromatography ($CHCl_3$/MeOH/NH_4OH, 100:1:1 → 100:5:1) to afford **26** (371 mg, 50%) as a white solid.

Compound 26: TLC (DCM:MeOH:NH_4OH, 100:3:1 v/v):R_f = 0.6 (UV, $KMnO_4$); ^{1}H NMR (600 MHz, $CDCl_3$) δ: 6.63 (d, *J* = 7.8 Hz, 1H), 5.92 (d, *J* = 7.8 Hz, 1H), 4.25 (dd, *J* = 15.0, 7.8 Hz, 1H), 4.18 (d, *J* = 15.0 Hz, 1H), 3.82 (s, 3H), 3.44 (t, *J* = 4.8 Hz, 1H), 2.92 (m, 1H), 2.79 (m, 1H), 2.64 (dd, *J* = 12.0, 4.2 Hz, 1H), 2.59-2.55 (m, 2H), 2.52 (t, *J* = 4.8 Hz, 1H), 2.18 (s, 3H), 2.11 (dd, *J* = 18.6, 5.4 Hz, 1H); ^{13}C NMR (150 MHz, $CDCl_3$) δ: 216.1, 158.3, 148.0. 139.2, 112.4, 102.7, 63.1, 55.8, 54.8, 44.2, 43.9, 43.1, 37.1, 36.3, 35.7; IR (KBr): 2925, 2853, 1745, 1651, 1594, 1558, 1464, 1263, 1224; HRMS (ESI, m/z) calcd for $C_{15}H_{19}N_2O_3$[M+H]$^+$: 275.1390, found 275.1400.

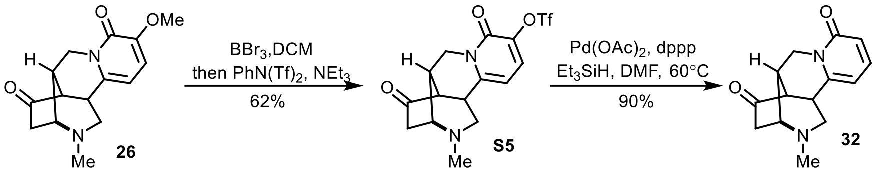

To a solution of **26** (200 mg, 0.73 mmol, 1.0 equiv) in dry DCM (20 ml) was added BBr_3 (2.2 ml, 17% in methylene chloride, 2.2 mmol, 3.0 equiv) at -78℃. The reaction mixture was warmed to RT and stirred at this temperature for 2 h. The resulting mixture was cooled to -78℃ and quenched with NH_4OH (2 ml). Removal of the solvent under reduced pressure to afford crude product. To a solution of the crude product obtained above in dry DCM (20 ml) was added NEt_3 (0.2 ml, 1.46 mmol, 2.0 equiv) and $PhN(Tf)_2$ (391 mg, 1.1 mmol, 1.5 equiv) at RT. After stirring at RT for 12 h, the reaction mixture was quenched with sat. aq. $NaHCO_3$ and extracted with DCM (3 x 20 mL). The combined organic layers were washed with brine, dried ($MgSO_4$), filtered and concentrated. The residue was purified by column chromatography (DCM/MeOH/NH_4OH, 100:2.5:1 → 100:5:1) to afford **S5** (177mg, 62%) as a white solid.

To a solution of **S5** (177 mg, 0.45 mmol, 1.0 equiv) in dry DMF (10 ml) was added $Pd(OAc)_2$ (25 mg, 0.09 mmol, 0.2 equiv) and dppp (37 mg, 0.09 mmol, 0.2 equiv) at RT. The reaction mixture was subjected to freeze-pump-thaw cycles with Argon gas twice, then bubbling with Argon gas at room temperature for 3 min before Et_3SiH (0.19 ml, 1.35 mmol, 3.0 equiv) was added. After heating at 60℃ for 2 h, the reaction mixture was filtered through a pad of Celite and the combined filtrate was concentrated *in vacuo*. The residue was purified by column chromatography ($CHCl_3$/MeOH/NH_4OH, 100:1:1 → 100:5:1) to afford **32** (99 mg, 90%) as a

white solid.

The structure of **32** was confirmed by 2D-NMR spectra analysis (DEPT-135, COSY, HSQC, HMBC, NOESY)

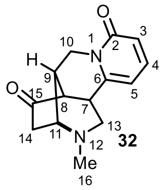

Compound 32: TLC (DCM:MeOH:NH_4OH, 100:3:1 v/v):R_f = 0.6 ($KMnO_4$); ^1H NMR (600 MHz, $CDCl_3$) δ: 7.30 (dd, *J* = 9.0, 6.6 Hz, 1H, **-H4**), 6.51 (d, *J* = 9.0 Hz, 1H, **-H3**), 5.98 (d, *J* = 6.6 Hz, 1H, **-H5**), 4.19 (dd, *J* = 15.6, 8.4 Hz, 1H, **-H10**), 4.11 (d, *J* = 15.6 Hz, 1H, **-H10**), 3.46 (t, *J* = 4.8 Hz, 1H, **-H11**), 2.95 (m, 1H, **-H7**), 2.82 (m, 1H, **-H9**), 2.67 (dd, *J* = 12.0, 4.2 Hz, 1H, **-H13**), 2.62 (d, *J* = 12.0, 1H, **-H13**), 2.57 (d, *J* = 18.6 Hz, 1H, **-H14**), 2.53 (t, *J* = 4.8 Hz, 1H, **-H8**), 2.19 (s, 3H, **-H16**), 2.12 (dd, *J* = 18.6, 6.0 Hz, 1H, **-H14**); ^{13}C NMR (150 MHz, $CDCl_3$) δ: 215.8 (**-C15**), 163.2 (**-C2**), 149.0 (**-C6**), 138.9 (**-C4**), 117.7 (**-C3**), 104.5 (**-C5**), 63.1 (**-C11**), 54.4 (**-C13**), 43.8 (**-C10**), 43.3 (**-C8**), 43.1 (**-C16**), 37.5 (**-C7**), 36.5 (**-C9**), 35.7 (**-C14**); IR (KBr): 2853, 1743, 1652, 1548, 1479, 1163, 1146; HRMS (ESI, m/z) calcd for $C_{14}H_{17}N_2O_2[M+H]^+$: 245.1285, found 245.1296.

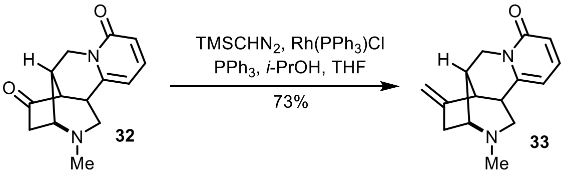

To a solution of **32** (11 mg, 0.045 mmol, 1 equiv), PPh_3 (78 mg, 0.298 mmol, 6.6 equiv), $Rh(PPh_3)Cl$ (2.1 mg, 0.0023 mmol, 0.05 equiv) in dry THF (2 ml) was added *i*-PrOH (0.31 ml, 4.05 mml, 90 equiv) at RT. $TMSCHN_2$ (0.45 ml, 2.0 M in ether, 0.90 mmol, 20 equiv) was then added dropwise over 5 minutes at RT. After stirring at RT for 24 h, the reaction mixture was diluted with EtOAc and filtered through a a pad of Celite and the combined filtrate was concentrated *in vacuo*. The residue was purified by column chromatography ($CHCl_3$/MeOH/NH_4OH, 100:1:1→100:5:1) to afford **33** (8.0 mg, 73%) as a colorless oil.

Compound 33:TLC (DCM:MeOH:NH_4OH, 100:3:1 v/v):R_f = 0.65 ($KMnO_4$); ^1H NMR (600 MHz, $CDCl_3$) δ: 7.28 (dd, *J* = 9.0, 6.6 Hz, 1H), 6.47 (dd, *J* = 9.0, 1.2 Hz, 1H), 5.95 (dd, *J* = 6.6, 1.2 Hz, 1H), 5.06 (brs, 1H), 5.04 (brs, 1H), 4.13 (dd, *J* = 15.6, 7.8 Hz, 1H), 4.07 (d, J = 15.6 Hz,

1H), 3.11 (t, J = 4.8 Hz, 1H), 2.70-2.62 (m, 4H), 2.48-2.44 (m, 2H), 2.16 (m, 1H), 2.12 (s, 3H); ^{13}C NMR (150 MHz, $CDCl_3$) δ: 163.5, 151.3, 150.7, 138.8, 116.9, 107.3, 104.5, 65.4, 54.4, 44.1, 43.0, 41.4, 39.7, 38.6, 28.3; IR (KBr): 2927, 1851, 2799, 1651, 1591, 1559, 1461, 1225, 1052; HRMS (ESI, m/z) calcd for $C_{15}H_{19}N_2O$ $[M+H]^+$: 243.1492, found 243.1499.

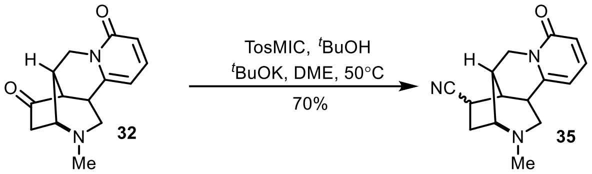

To a solution of **32** (40 mg, 0.17 mmol, 1.0 equiv) in dry DME (8 ml) was added TosMIC (64 mg, 0.33 mmol, 2.0 equiv) and ^{t}BuOH (63 ul, 0.66 mmol, 4.0 equiv). Then the raction mixture was added ^{t}BuOK (46 mg, 0.41 mmol, 2.5 equiv) at 0℃. The resulting mixture was stirred at the same temperature for 30 min before it was heated at 40 ℃ for 12h. After the solution was cooled to RT, the reaction was quenched with saturated NH_4Cl (5 ml) and extracted with EtOAc (3 x 10 mL). The combined organic layers were washed with brine, dried ($MgSO_4$), filtered and concentrated. The residue was purified by column chromatography (DCM/MeOH/NH_4OH, 100:2.5:1 → 100:5:1) to afford inseparable diastereomeric mixture of compound **35** (29 mg, 62%, dr: 1:1) as a white solid.

Compound 35 (1:1 mixture**):** TLC (DCM:MeOH:NH_4OH, 100:3:1 v/v):R_f = 0.7 ($KMnO_4$); ^{1}H NMR (600 MHz, $CDCl_3$) δ: 7.30 (dd, J = 9.0, 7.2 Hz, 1H), 7.28 (dd, J = 9.0, 6.6 Hz, 1H), 6.48 (d, J = 9.0 Hz, 2H), 6.02 (d, J = 6.6 Hz, 1H), 5.94 (d, J = 6.6 Hz, 1H), 4.14 (m, 2H), 4.11-4.07 (m, 2H), 3.23 (t, J = 4.8 Hz, 1H), 3.19-3.15 (m, 2H), 2.99 (brs, 1H), 2.86 (dd, J = 9.6, 5.4 Hz, 1H), 2.74 (dd, J = 12.0, 4.2 Hz, 1H), 2.72 (m, 1H), 2.67-2.64 (m, 2H), 2.64-2.61 (m, 1H), 2.56 (d, J = 12.0 Hz, 1H), 2.52 (m, 1H), 2.40-2.35 (m, 2H), 2.33 (m, 1H), 2.15 (dd, J = 15.0, 5.4 Hz, 1H), 2.11 (s, 3H), 2.09 (s, 3H), 2.04 (ddd, J = 14.4, 12.6, 5.4 Hz, 1H), 1.88 (ddd, J = 14.4, 5.4, 4.8 Hz, 1H); ^{13}C NMR (150 MHz, $CDCl_3$) δ: 163.3, 163.2, 149.9, 149.5, 139.0, 138.8, 122.3, 120.1, 117.7, 117.6, 104.7, 104.5, 65.8, 65.4, 54.2, 54.0, 43.6, 43.5, 42.8, 42.7, 38.9, 38.8, 38.4, 38.0, 37.1, 35.6, 29.9, 29.2, 26.9, 25.5; IR (KBr): 2924, 2851, 1735, 1650, 1549, 1455, 1261, 811; HRMS (ESI, m/z) calcd for $C_{15}H_{18}N_3O$ $[M+H]^+$: 256.1444, found 256.1450.

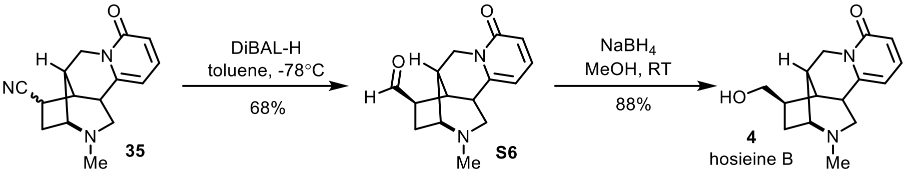

To a solution of **35** (15 mg, 0.059 mmol, 1.0 equiv) in dry toluene (3 ml) was added DiBAL-H (70 ul, 1.1 M in cyclohexane, 0.102 mmol, 1.3 equiv) at -78℃. After stirring at same temperature for 1h, the reaction the reaction mixture was quenched with 10% aq. potassium sodium tartrate (5 mL) and extracted with $CHCl_3$ (3 x 10 mL). The combined layers were washed with brine (10 mL), dried ($MgSO_4$), filtered and concentrated. The residue was purified by column chromatography (DCM/MeOH/NH_4OH, 100:1:1 → 100:5:1) to afford **S6** (10.3 mg, 68%).

To a solution of **S6** (10 mg, 0.039 mmol, 1.0 equiv) in dry MeOH (2 ml) was added $NaBH_4$ (3 mg, 0.078 mmol, 2 equiv) at 0℃. After stirring at RT for 1h, the reaction the reaction mixture was quenched with saturated NH_4Cl (1 ml) and extracted with $CHCl_3$ (3 x 5 mL). The combined layers were washed with brine (5 mL), dried ($MgSO_4$), filtered and concentrated. The residue was purified by column chromatography (DCM/MeOH/NH_4OH, 100:1.7:1 → 100:10:1) to afford hosieine (**4**) (8.9 mg, 88%) as a white solid.

Hosieine B (**4**): TLC (DCM:MeOH:NH_4OH, 100:3:1 v/v):R_f = 0.3 ($KMnO_4$); ^{1}H NMR (600 MHz, CD_3OD) δ: 7.46 (dd, *J* = 9.0, 7.2 Hz, 1H), 6.43 (dd, *J* = 9.0, 1.2 Hz, 1H), 6.25 (dd, *J* = 7.2, 1.2 Hz, 1H), 4.07-4.00 (m, 2H), 3.49 (dd, *J* = 10.8, 6.0 Hz, 1H), 3.44 (dd, *J* = 10.8, 8.4 Hz, 1H), 3.12 (m, 1H), 2.80 (m, 1H), 2.66 (dd, *J* = 11.4, 4.2 Hz, 1H), 2.51 (d, *J* = 12.0 Hz, 1H), 2.45 (m, 1H), 2.17 (t, *J* = 4.8 Hz, 1H), 2.14 (m, 1H), 2.11 (s, 3H), 2.08 (m, 1H), 1.17 (dt, *J* = 13.8, 4.8 Hz, 1H); ^{13}C NMR (150 MHz, CD_3OD) δ:165.7, 153.9, 141.7, 116.9, 107.9, 67.9, 66.1, 56.2, 45.6, 44.5, 43.2, 41.3, 37.9, 35.4, 25.5; IR (KBr): 2924, 2853, 1741, 1647, 1548, 1465, 1364, 1261, 1027; HRMS (ESI, m/z) calcd for $C_{15}H_{21}N_2O_2$ $[M+H]^+$: 261.1598, found 261.1606.

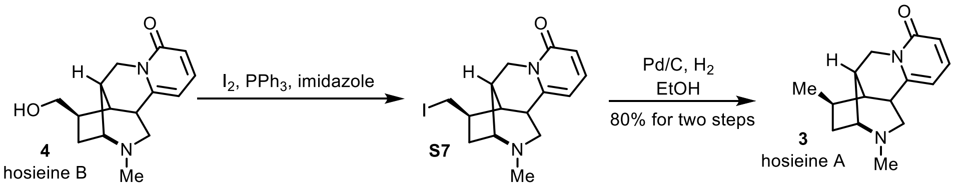

To a solution of **4** (10 mg, 0.038 mmol, 1.0 equiv), imidazole (5.2 mg, 0.077 mmol, 2.0

equiv), and PPh_3 (20.2 mg, 0.077 mmol, 2.0 equiv) in dry DCM (1.5 ml) was added I_2 (19.5 mg, 0.077 mmol, 2.0 equiv) at 0℃. After stirring at RT for 12 h, the reaction mixture was filtered through Celite and concentrated *in vacuo.* The residue was vacuum filtered through a short plug of silica gel using DCM/MeOH/NH_4OH (100:10:1) as the eluent, the combined eluent was concentrated *in vacuo* to afford the crude **S7**, which was used directly into next step without intensive purification.

To a solution of crude **S7** obtained above in dry EtOH (1.5 mL) was added Pd/C (8 mg, 5wt%.) at RT. The reaction mixture was bubbled with H_2 at RT for 5 minutes, then stirred under a balloon for 5 h. The reaction was filtered through Celite. The filtrate was concentrated *in vacuo* to afford crude product which was purified by column chromatography ($CHCl_3$/MeOH/NH_4OH, 100:1:1 → 90:9:1) to afford hosieine A (**3**) (7.5 mg, 80%) as a white solid.

Hosieine A (**3**): TLC (DCM:MeOH:NH_4OH, 100:2:1 v/v):R_f = 0.4 ($KMnO_4$); ^{1}H NMR (600 MHz, CD_3OD) δ: 7.47 (dd, *J* = 9.0, 7.2 Hz, 1H), 6.43 (dd, *J* = 9.0, 1.2 Hz, 1H), 6.25 (dd, *J* = 7.2, 1.2 Hz, 1H), 4.10-4.03 (m, 2H), 3.12 (t, *J* = 4.8 Hz, 1H), 2.81 (m, 1H), 2.63 (dd, *J* = 11.4, 4.2 Hz, 1H), 2.54 (m, 1H), 2.48 (d, *J* = 12.0 Hz, 1H), 2.23 (dd, *J* = 13.8, 8.4 Hz, 1H), 2.12 (m, 1H), 2.09 (s, 3H), 1.87 (t, *J* = 4.2 Hz, 1H), 1.12 (d, *J* = 7.2 Hz, 3H), 1.09 (dt, == 14.4, 4.8 Hz, 1H); ^{13}C NMR (150 MHz, CD_3OD) δ: 165.7, 154.2, 141.6, 116.8, 107.8, 68.8, 56.3, 45.9, 43.2, 41.5, 40.5, 37.6, 36.2, 31.1, 22.7; IR (KBr): 2924, 2853, 1740, 1651, 1549, 1464, 1377, 1251; HRMS (ESI, m/z) calcd for $C_{15}H_{21}N_2O$ $[M+H]^+$: 245.1648, found 245.1655.

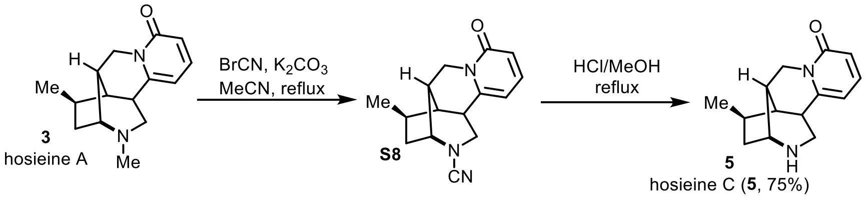

To a solution of **3** (5 mg, 0.02 mmol, 1.0 equiv) and K_2CO_3 (8.5 mg, 0.061 mmol, 3.0 equiv), in dry MeCN (1.0 ml) was added BrCN (6.5 mg, 0.061, 3.0 equiv) at RT. After stirring at 88℃ for 12 h, the reaction mixture was quenched with brine (2 ml) and extracted with $CHCl_3$ (3 x 3 ml). The combined layers were dried ($MgSO_4$), filtered and concentrated to afford crude **S8**, which was used directly into next step without purification.

To a solution of crude **S8** obtained above in aq.HCl (1 ml, 3N) were added MeOH (0.1 ml) at

RT, After stirring at 110℃ for 15h, the reaction mixture was concentrated in *vacuo* to remove the solvent. The residue was purified by column chromatography ($CHCl_3$/MeOH/NH_4OH, 100:1:1 → 90:9:1) to afford hosieine C (**5**) (3.5 mg, 75%) as a white solid.

Hosieine C (**5**): TLC (DCM:MeOH:NH_4OH, 100:3:1 v/v):R_f = 0.4 ($KMnO_4$); ^{1}H NMR (600 MHz, $CDCl_3$) δ: 7.48 (dd, *J* = 9.0, 7.2 Hz, 1H), 6.45 (dd, *J* = 9.0, 1.2 Hz, 1H), 6.24 (d, *J* = 7.2 Hz, 1H), 4.17 (d, *J* = 16.2 Hz, 1H), 4.10 (dd, *J* = 16.2, 7.8 Hz, 1H), 3.39 (m, 1H), 3.36 (m, 1H), 2.80 (m, 1H), 2.61 (d, *J* = 12.6 Hz, 1H), 2.49 (m, 1H), 2.34 (m, 1H), 2.12 (dd, *J* = 13.8, 8.4 Hz, 1H), 1.93 (t, *J* = 4.8 Hz, 1H), 1.53 (dt, *J* = 13.8, 5.4 Hz, 1H), 1.12 (d, *J* = 7.2 Hz, 3H); ^{13}C NMR (150 MHz, CD_3OD) δ: 165.9, 153.9, 141.7, 116.8, 107.9, 61.3, 47.6, 45.6, 41.8, 41.2, 39.3, 37.1, 36.0, 22.8; IR (KBr): 2918, 2850, 1547, 1470, 1335, 1217, 1148, 1079, 801; HRMS (ESI, m/z) calcd for $C_{14}H_{19}N_2O$ $[M+H]^+$: 231.1492, found 231.1498.

4. NMR comparison of synthetic and natural hosieines

Table S7. ^{1}H NMR Spectroscopic (CD_3OD, 25℃) Comparison of Natural [6], Hong's [7] and Our Synthetic (±)-hosieine A (3)

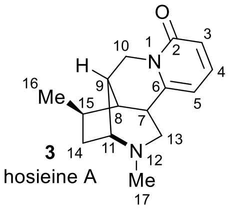

No.	Natural (500 MHz) δ ^{1}H [ppm, mult, J (Hz)]	Hong's (400 MHz) δ ^{1}H [ppm, mult, J (Hz)]	Ours (600 MHz) δ ^{1}H [ppm, mult, J (Hz)]
1			
2			
3	6.43 (1H, dd, J = 9.0, 1.3 Hz)	6.45 (1H, dd, J = 8.8, 1.2 Hz)	6.43 (1 H, dd, J = 9.0, 1.2 Hz)
4	7.46 (1H, dd, J = 9.0, 7.0 Hz)	7.49 (dd, J = 8.8, 7.2 Hz, 1H)	7.47 (1H, dd, J = 9.0, 7.2 Hz)
5	6.25 (1H, dd, J = 7.0, 1.3 Hz)	6.27 (1H, dd, J = 7.2, 0.8 Hz)	6.25 (1H, dd, J = 7.2, 1.2 Hz)
6			
7	2.81 (1H, tt, J = 4.2, 2.0 Hz)	2.86-2.79 (m, 1H)	2.81 (1H, m)
8	1.87 (1H, t, J = 4.5 Hz)	1.89 (1H, t, J = 4.4 Hz)	1.87 (1H, t, J = 4.2 Hz)
9	2.53 (1H, m)	2.59-2.52 (1H, m)	2.54 (1H, m)
10	4.06 (2H, m)	4.10-4.08 (2H, m)	4.10-4.03 (2H, m)
11	3.12 (1H, t, J = 5.0 Hz)	3.14 (1H, t, J = 4.8 Hz)	3.12 (1H, t, J = 4.8 Hz)
12			
13	2.63 (1H, dd, J = 11.6, 4.2 Hz)<br>2.48 (1H, brd, J = 11.6 Hz)	2.65 (1H, dd, J = 11.6, 4.2 Hz)<br>2.50 (1H, d, J = 11.6 Hz, 1H)	2.63 (1H, dd, J = 11.4, 4.2 Hz)<br>2.48 (1H, d, J = 12.0 Hz)
14	2.23 (1H, dd, J = 14.2, 8.8 Hz)<br>1.09 (1H, dt, J = 14.2, 5.0 Hz)	2.25 (dd, J = 14.2, 8.8 Hz)<br>1.14-1.09 (m, 1H)	2.23 (1H, dd, J = 13.8, 8.4 Hz)<br>1.09 (1H, dt, J = 14.4, 4.8 Hz)
15	2.12 (1H, m)	2.13-2.11 (1H, m)	2.12 (1H, m)
16	1.11 (3H, d, J = 7.0 Hz)	1.14 (3H, d, J = 6.8 Hz)	1.12 (1H, d, J = 7.2 Hz)
17	2.09 (3H, s)	2.09 (3H, s)	2.09 (3H, s)

Table S8. ^{13}C NMR Spectroscopic (CD_3OD, 25℃) Comparison of Natural [6], Hong's [7] and Our Synthetic (±)-hosieine A (3)

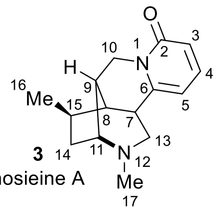

3
hosieine A

No.	Natural (125 MHz) δ ^{13}C (ppm)	Hong's (100 MHz) δ ^{13}C (ppm)	Ours (150 MHz) δ ^{13}C (ppm)
1			
2	165.7	165.7	165.7
3	116.8	116.7	116.8
4	141.7	141.6	141.6
5	107.8	107.7	107.8
6	154.3	154.2	154.2
7	41.5	41.2	41.5
8	40.5	40.5	40.5
9	37.6	37.6	37.6
10	45.9	45.9	45.9
11	68.8	68.7	68.8
12			
13	56.3	56.4	56.3
14	31.1	31.1	31.1
15	36.2	36.1	36.2
16	22.7	22.7	22.7
17	43.2	43.1	43.2

Table S9. ^{1}H NMR Spectroscopic (CD$_3$OD, 25℃) Comparison of Natural [6] and Our Synthetic (±)-hosieine B (4)

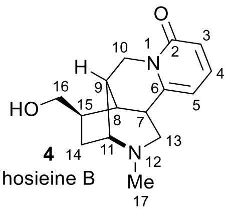

No.	Natural (500 MHz) δ ^{1}H [ppm, mult, *J* (Hz)]	Ours (600 MHz) δ ^{1}H [ppm, mult, *J* (Hz)]	△δ
1			
2			
3	6.44 (1H, dd, *J* = 9.0, 1.4 Hz)	6.43 (1H, dd, *J* = 9.0, 1.2 Hz)	0.01
4	7.47 (1H, dd, *J* = 9.0, 7.0 Hz)	7.46 (1H, dd, *J* = 9.0, 7.2 Hz)	0.01
5	6,26 (1H, dd, *J* = 7.0, 1.4 Hz)	6.25 (1H, dd, *J* = 7.2, 1.2 Hz)	0.01
6			
7	2.80 (1H, tt, *J* = 4.0, 1.8 Hz)	2.80 (1H, m)	0
8	2.14 (1H, m)	2.14 (1H, m)	0
9	2.45 (1H, m)	2.45 (1H, m)	0
10	4.05 (2H, m)	4.07-4.00 (2H, m)	0
11	3.11 (1H, d, *J* = 4.9 Hz)	3.12 (1H, m)	-0.01
12			
13	2.65 (1H, dd, *J* = 11.8, 4.2 Hz)	2.66 (1H, dd, *J* = 11.4, 4.2 Hz)	-0.01
	2.51 (1H, brd, *J* = 11.6 Hz)	2.51 (1H, d, *J* = 12.0 Hz)	0
14	2.09 (1H, m)	2.08 (1H, m)	0.01
	1.16 (1H, dt, *J* = 13.9, 4.9 Hz)	1.17 (1H, dt, *J* = 13.8, 4.8 Hz)	-0.01
15	2.18 (1H, t, *J* = 4.4 Hz)	2.17 (1H, t, *J* = 4.8 Hz)	0.01
16	3.51 (1H, dd, *J* = 10.6, 6.0 Hz)	3.49 (1H, dd, *J* = 10.8, 6.0 Hz)	0.02
	3.45 (1H, dd, *J* = 10.6, 8.2 Hz)	3.44 (1H, dd, *J* = 10.8, 8.4 Hz)	0.01
17	2.11 (3H, s)	2.11 (3H, s)	0

Table S10. ^{13}C NMR Spectroscopic (CD_3OD, 25℃) Comparison of Natural [6] and Our Synthetic (±)-hosieine B (4)

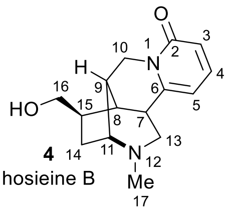

4
hosieine B

No.	Natural (125 MHz) δ ^{13}C (ppm)	Ours (150 MHz) δ ^{13}C (ppm)	△δ
1			
2	165.7	165.7	0
3	116.9	116.9	0
4	141.7	141.7	0
5	107.8	107.9	-0.1
6	154.1	153.9	0.2
7	41.4	41.3	0.1
8	44.5	44.5	0
9	37.9	37.9	0
10	45.7	45.6	0.1
11	67.9	67.9	0
12			
13	56.3	56.2	0.1
14	25.4	25.5	-0.1
15	35.5	35.4	0.1
16	66.2	66.1	0.1
17	43.3	43.2	0.1

Table S11. **^{1}H NMR Spectroscopic (CD_3OD, 25℃) Comparison of Natural [6], and Our Synthetic (±)-hosieine C (5)**

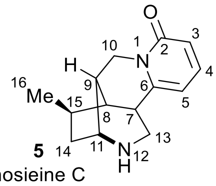

No.	Natural (500 MHz) δ ^{1}H [ppm, mult, *J* (Hz)]	Ours (600 MHz) δ ^{1}H [ppm, mult, *J* (Hz)]	△δ
1			
2			
3	6.45 (1H, dd, *J* = 9.0, 1.3 Hz)	6.45 (1H, dd, *J* = 9.0, 1.2 Hz)	0
4	7.48 (1H, dd, *J* = 9.0, 7.0 Hz)	7.48 (1 H, dd, *J* = 9.0, 7.2 Hz)	0
5	6.25 (1H, dd, *J* = 7.0, 1.3 Hz)	6.24 (1H, d, *J* = 7.2 Hz)	0.01
6			
7	2.80 (1H, t, *J* = 4.2 Hz)	2.80 (1H, m)	0
8	1.93 (1H, t, *J* = 4.6 Hz)	1.93 (1H, t, *J* = 4.8 Hz)	0
9	2.49 (1H, m)	2.49 (1H, m)	0
10	4.16 (1H, d, *J* = 16.2 Hz)	4.17 (1H, d, *J* = 16.2 Hz)	-0.01
	4.11 (1H, dd, *J* = 16.2, 7.7 Hz)	4.10 (1H, dd, *J* = 16.2, 7.8 Hz)	0.01
11	3.38 (1H, m)	3.39 (1H, m)	-0.01
12			
13	3.34 (1H, m)	3.36 (1H, m)	-0.02
	2.60 (1H, d, *J* = 13.0 Hz)	2.61 (1H, d, *J* = 12.6 Hz)	-0.01
14	2.12 (1H, dd, *J* = 14.0, 8.7 Hz)	2.12 (1H, dd, *J* = 13.8, 8.4 Hz)	0
	1.52 (1H, dt, *J* = 14.0, 5.4 Hz)	1.53 (1H, dt, *J* = 13.8, 5.4 Hz)	-0.01
15	2.33 (1H, m)	2.34 (1H, m)	-0.01
16	1.11 (3H, d, *J* = 7.0 Hz)	1.12 (3H, d, *J* = 7.2 Hz)	-0.01

Table S12. ^{13}**C NMR Spectroscopic (CD_3OD, 25℃) Comparison of Natural** [6]**, and Our Synthetic (±)-hosieine C (5)**

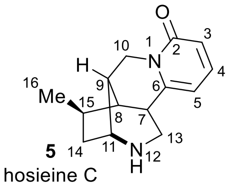

No.	Natural (125 MHz) δ ^{13}C (ppm)	Ours (150 MHz) δ ^{13}C (ppm)	△δ
1			
2	165.9	165.9	0
3	116.8	116.8	0
4	141.7	141.7	0
5	107.9	107.9	0
6	153.9	153.9	0
7	41.8	41.8	0
8	41.1	41.2	-0.1
9	37.0	37.1	-0.1
10	45.6	45.6	0
11	61.3	61.3	0
12			
13	47.6	47.6	0
14	39.3	39.3	0
15	36.0	36.0	0
16	22.8	22.8	0

5. X-Ray Crystal Structure of 16b.

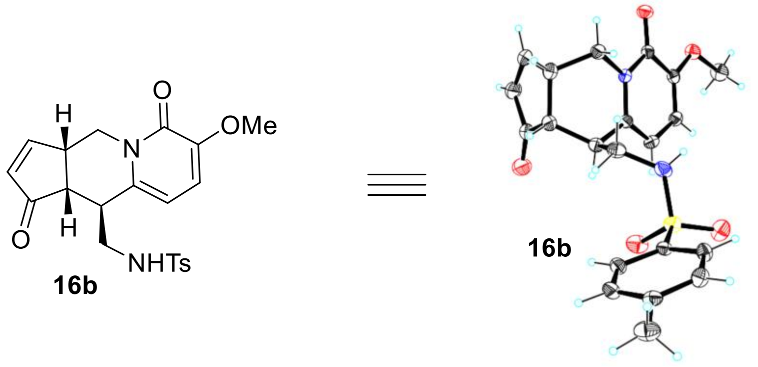

Table S. Crystal data and structure refinement for CCDC 2293123.

Identification code	CCDC 2293123	
Chemical formula	$C_{42}H_{44}N_4O_{10}S_2$	
Formula weight	828.93 g/mol	
Temperature	280(2) K	
Wavelength	0.71073 Å	
Crystal size	0.100 x 0.180 x 0.190 mm	
Crystal habit	clear light colourless plate	
Crystal system	triclinic	
Space group	P -1	
Unit cell dimensions	a = 9.712(3) Å	α = 84.044(16) °
	b = 13.851(6) Å	β = 76.080(11) °
	c = 16.492(7) Å	γ = 69.600(11) °
Volume	2017.8(13) $Å^3$	
Z	2	
Density (calculated)	1.364 g/cm^3	
Absorption coefficient	0.196 mm^{-1}	
F(000)	872	
Diffractometer	d8 venture	
Theta range for data collection	2.04 to 28.43°	
Index ranges	-12<=h<=12, -18<=k<=18, -22<=l<=22	
Reflections collected	75504	
Independent reflections	10107 [R(int) = 0.0782]	
Coverage of independent reflections	99.6%	
Absorption correction	Multi-Scan	
Max. and min. transmission	0.9810 and 0.9640	

Structure solution technique	direct methods	
Structure solution program	SHELXT 2018/2 (Sheldrick, 2018)	
Refinement method	Full-matrix least-squares on F^2	
Refinement program	SHELXL-2018/3 (Sheldrick, 2018)	
Function minimized	Σ w $(Fo^2 - Fc^2)^2$	
Data / restraints / parameters	10107 / 42 / 533	
Goodness-of-fit on F2	1.019	
Δ/σ_{max}	0.046	
Final R indices	6141 data; I>2σ(I)	R1 = 0.0527, wR2 = 0.1200
	all data	R1 = 0.1028, wR2 = 0.1459
Weighting scheme	$w=1/[\sigma^2(Fo^2)+(0.0545P)^2+0.8104P]$ where $P=(Fo^2+2Fc^2)/3$	
Largest diff. peak and hole	0.303 and -0.514 eÅ^{-3}	
R.M.S. deviation from mean	0.045 eÅ^{-3}	

6. DFT Calculations

6.1 Computational Methodology

All DFT calculations were performed using Gaussian 16 Version C.01 suite of programs[8]. The geometries listed below were optimized at the M062X/6-31+G(d) level of theory. Frequency analyses revealed no virtual frequencies in all the optimized structures. Thermochemical corrections were obtained from frequency calculations at the same level of theory without scaling.

6.2 Calculated Energies

Table S13. Energy differences of compounds 19a/b calculated with M062X/6-311+G(d,p)

Structure	M062X/6-31+G(d)	
	Gibbs free energies[a]	ΔG(**19a-19b**)[b]
**19a**	-1735.577546	-3.2
**19b**	-1735.572496	

[a] in Hartree. [b] kcal/mol.

Table S14. Energy differences of compounds 20a/b calculated with M062X/6-311+G(d,p)

Structure	M062X/6-31+G(d)	
	Gibbs free energies[a]	ΔG(**20a-20b**)[b]
**20a**	-1900.738748	-4.7
**20b**	-1900.731285	

[a] in Hartree. [b] kcal/mol.

Table S15. Energy differences of compounds 16c, 22 calculated with M062X/6-311+G(d,p)

Structure	M062X/6-31+G(d)，	
	Gibbs free energies[a]	ΔG(**16c-22**)[b]
**16c**	-1861.503367	-2.8
**22**	-1861.498898	

[a] in Hartree. [b] kcal/mol.

Table S16. Energy differences of compounds 18, 21 calculated with M062X/6-311+G(d,p)

Structure	M062X/6-31+G(d)，	
	Gibbs free energies[a]	ΔG(**18-21**)[b]
**18**	-1940.007367	-2.7
**21**	-1940.003090	

[a] in Hartree. [b] kcal/mol.

6.3 The geometries listed below were optimized at the M062X/6-31+G(d) level of theory.

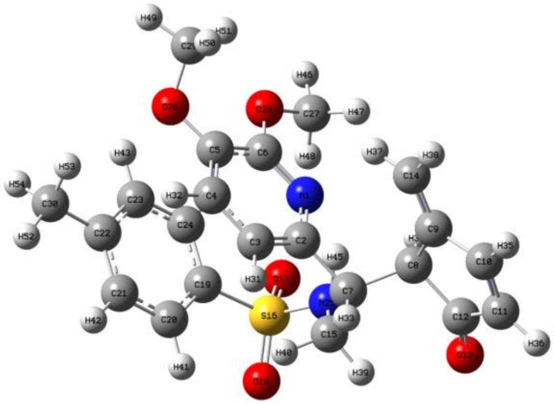

19a

7	1.64769891	-0.54957116	0.41325079
6	-0.48032006	-1.92365888	0.69931976
6	-2.38771952	-1.18709898	2.33069381
6	-2.08219243	1.04966350	3.71319377
6	0.08362234	2.47010448	3.41699737
6	1.91396176	1.57546112	1.68353638
6	-0.72820104	-4.22431786	-0.98406400
6	1.68519350	-5.88141228	-0.91108160
6	2.23416576	-6.94264129	1.70210820
6	1.98406989	-9.70365057	1.55110736
6	1.49603583	-10.49361430	-0.81273314
6	1.36909274	-8.28126997	-2.52600481

8	1.06410436	-8.33616054	-4.79328219
6	2.87892901	-5.70697713	3.81125657
6	-1.50282067	-3.37264994	-3.66341516
16	-0.51743355	0.83824505	-6.34341105
8	1.79529903	2.00502382	-7.27258906
8	-2.57083764	0.05686701	-7.99383900
6	-1.82580257	2.95574033	-4.06120411
6	-4.36252026	2.76360574	-3.39070819
6	-5.31165158	4.34521515	-1.50913381
6	-3.76866622	6.12319691	-0.31337622
6	-1.24487018	6.33347311	-1.08258144
6	-0.26379843	4.76040528	-2.94068835
7	0.41361021	-1.72939617	-4.81369477
8	3.97070640	3.02436689	1.31803468
6	5.75485178	2.16947960	-0.51178954
8	0.33396400	4.74475997	4.60277174
6	2.32751574	4.88211303	6.41337030
6	-4.75304799	7.75927701	1.79684256
1	-4.08986473	-2.31617300	2.51845594
1	-3.53440700	1.73026106	4.99542432
1	-2.29703239	-5.37620818	-0.26348420
1	3.29363270	-4.79393229	-1.63234312
1	2.21635377	-10.90484064	3.19983094
1	1.25369791	-12.41593971	-1.47338407
1	3.10408238	-3.66998660	3.86756786
1	3.22195772	-6.74263437	5.55245082
1	-1.79725573	-5.00363130	-4.89097711
1	-3.30668578	-2.36603676	-3.48994889
1	-5.58043750	1.43087273	-4.36713847
1	-7.28849072	4.19599707	-0.96858812
1	-0.02363119	7.71437612	-0.17477369
1	1.68836623	4.93478022	-3.54999034
1	1.88029963	-1.28371642	-3.64658706
1	7.25840042	3.57518250	-0.50179003
1	6.49460181	0.30991692	0.00208975
1	4.89310188	2.06621685	-2.39021936
1	2.25071558	6.78340996	7.20355174
1	2.01817091	3.49069897	7.91648291
1	4.16171231	4.56759698	5.51724395
1	-6.78383520	7.49676884	2.07218141
1	-3.77961720	7.29025674	3.56422995
1	-4.40677129	9.76218727	1.41083011

Frequencies

22.2469	32.6475	34.5420	41.0284	47.8742	51.2481
70.4453	79.6942	86.1299	88.2500	103.4493	120.2283
121.1244	148.2597	149.5500	168.1223	170.6856	182.0652
194.5608	213.2605	224.6266	241.8657	256.2918	269.6045
285.5878	297.9985	303.1367	325.0657	343.5352	348.9955
361.2165	376.8012	391.0661	419.0065	433.6870	456.9631
465.6694	488.3561	520.0079	526.8479	539.1127	554.1284
567.6869	576.8342	586.7338	614.4127	622.1321	632.4122
645.4061	676.3510	688.2356	715.3837	720.5598	722.6315
740.4242	762.7239	796.4295	818.0061	827.3070	839.0992
854.2821	857.4349	868.3747	900.8259	915.6211	931.4927
950.9277	961.7818	964.8544	977.9140	988.7051	993.1118
1005.4931	1011.2227	1019.4041	1037.2915	1058.9213	1074.3191
1076.6987	1096.0721	1099.9502	1111.1485	1121.3227	1142.9604
1144.8401	1158.7447	1173.5035	1187.1089	1187.4904	1195.1235
1209.8646	1211.5304	1213.3447	1226.2938	1237.1197	1251.7595
1255.7369	1284.4476	1307.4041	1313.6474	1318.8588	1327.1434
1333.0037	1340.9789	1356.4947	1366.4217	1380.6913	1392.9398
1420.7135	1432.8414	1450.5486	1457.6590	1467.8963	1484.9576
1493.0558	1507.1802	1513.0909	1515.3279	1516.9008	1520.6719
1530.0288	1531.2518	1533.5541	1549.7906	1551.5387	1657.4321
1658.5701	1676.7927	1684.1836	1686.0912	1748.0050	1857.3882
3075.8635	3076.0096	3076.3715	3086.3041	3091.7271	3110.8075
3140.9038	3159.6542	3160.3682	3162.8292	3170.7306	3190.7578
3198.7635	3208.4360	3212.6857	3218.6165	3229.2984	3235.9292
3240.3106	3243.1618	3244.7489	3279.6354	3284.2158	3540.5297

E(RM062X) = -1735.95401156

Zero-point Energy Correction = 0.436466 (Hartree/Particle)

Thermal Correction to Energy =	0.464821
Thermal Correction to Enthalpy =	0.465765
Thermal Correction to Free Energy =	0.376465
Sum of electronic and zero-point Energies =	-1735.517545
Sum of electronic and thermal Energies =	-1735.489191
Sum of electronic and thermal Enthalpies =	-1735.488247
Sum of electronic and thermal Free Energies =	-1735.577546

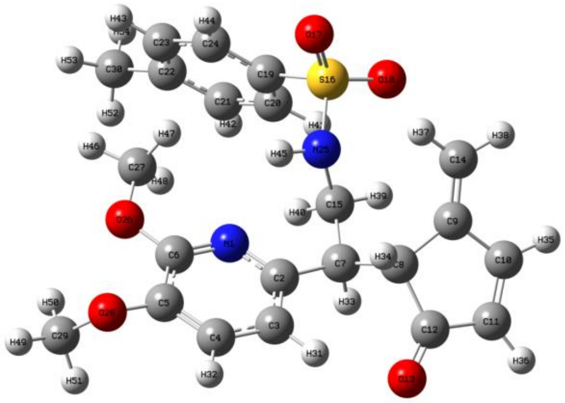

19b

7	2.82262864	-0.16012355	-0.10964508
6	2.92690894	-2.24049039	-1.57475260
6	5.21321411	-3.14479216	-2.49307218
6	7.43297830	-1.87521131	-1.82103233
6	7.33383814	0.23535105	-0.28584051
6	4.91825859	1.03730776	0.51004682
6	0.40738429	-3.57268129	-2.06749623
6	0.14555831	-4.51230175	-4.87212129
6	-2.51884317	-4.59963248	-5.95411096
6	-3.27447712	-7.26268769	-6.09755199
6	-1.35359766	-8.83379735	-5.54488499
6	0.92413109	-7.32904834	-4.95966747
8	3.02128652	-8.16292625	-4.54564181
6	-3.92601324	-2.70723793	-6.86087526
6	-1.82470477	-2.00939461	-1.09532077
16	-4.60502449	2.11420143	-2.04261023
8	-4.27945999	4.34366926	-3.61830560
8	-6.70989076	0.37635746	-2.38554514
6	-4.62871778	3.14693921	1.16551378
6	-5.41373182	1.48907256	3.05648543
6	-5.28542880	2.26438787	5.56546614
6	-4.41084867	4.67823110	6.20609312
6	-3.66899662	6.31354575	4.26594414

6	-3.77192124	5.56552570	1.74472670
7	-1.98760882	0.42064267	-2.41099736
8	4.79224724	3.13340461	1.95829307
6	2.33546357	4.08106685	2.49631964
8	9.47173740	1.54817711	0.29584881
6	10.15367564	1.50901316	2.90092688
6	-4.34772304	5.51562219	8.92753538
1	5.27629444	-4.83538284	-3.64951487
1	9.26677291	-2.51229727	-2.48766024
1	0.41472656	-5.26786695	-0.86219911
1	1.39492362	-3.39518860	-6.08485360
1	-5.16169906	-7.82480555	-6.67917098
1	-1.34639621	-10.88037585	-5.57251134
1	-3.28508748	-0.76235178	-6.84221692
1	-5.79296871	-3.08169174	-7.63174642
1	-3.58810819	-3.02371048	-1.44364180
1	-1.59332970	-1.73949540	0.94971123
1	-6.14388969	-0.36352880	2.55712638
1	-5.89118327	0.98267458	7.05287158
1	-3.01394367	8.20447530	4.73315814
1	-3.21946252	6.83511343	0.22914430
1	-0.43285399	1.51287520	-2.07643881
1	2.62615670	5.70711851	3.72691608
1	1.40006573	4.68388603	0.75097828
1	1.15932483	2.66067695	3.43270686
1	11.90534879	2.58385501	3.04802231
1	8.68958828	2.38958648	4.06314657
1	10.47888638	-0.44054066	3.52295979
1	-3.78152458	3.96737337	10.17435153
1	-3.03171695	7.08443143	9.20060946
1	-6.22224302	6.15297702	9.53451870

Frequencies

12.1058	22.4774	31.3212	37.3291	38.0555	51.3472
55.6889	65.9083	80.1166	86.7439	97.9037	120.7174
127.9091	134.9526	161.1121	168.0793	170.8028	185.3544
195.9969	207.4461	223.3835	227.8272	247.5967	271.6791
275.6850	291.1694	308.0604	325.2239	340.4991	357.5318
361.5570	374.9638	396.1838	419.6160	421.4622	458.2005
476.2696	500.3609	507.7578	538.7651	546.0725	561.5158

576.5301	581.8491	591.8422	619.4022	629.6802	644.5401
655.7471	680.0616	693.5333	717.0388	726.4220	729.5372
755.1211	766.0068	797.0809	824.6350	826.9339	844.5407
849.4797	875.6369	881.5721	887.8037	896.1212	925.1504
955.0856	968.5374	990.9469	994.0061	995.8206	1007.6224
1011.9276	1013.6418	1018.6907	1036.8002	1064.2845	1072.8721
1079.8907	1094.6308	1101.5039	1107.8611	1123.0277	1142.8131
1153.6121	1168.3332	1180.6796	1187.9857	1188.4541	1195.9670
1210.1502	1213.0169	1215.3951	1222.0804	1240.8330	1251.2762
1258.9366	1275.5287	1305.1731	1310.3795	1321.8988	1333.7528
1340.5243	1342.0881	1354.6495	1369.2622	1382.8445	1385.5095
1403.6869	1435.7215	1448.8427	1450.9168	1464.1688	1468.4786
1485.4545	1504.4466	1509.1217	1512.2042	1515.0238	1515.5443
1515.7494	1522.2283	1533.7518	1550.1699	1550.8647	1652.1802
1659.8909	1666.9364	1684.9877	1686.0732	1749.1606	1840.8529
3040.9573	3068.2251	3072.6497	3080.6597	3081.5791	3120.0369
3146.2192	3152.7028	3152.8175	3168.5066	3168.7816	3196.2995
3199.0934	3200.8248	3213.2965	3213.8654	3237.6631	3237.8878
3238.3097	3240.6031	3260.0924	3278.7952	3299.1802	3536.3393

E(RM062X) =	-1735.94696921
Zero-point Energy Correction =	0.436449 (Hartree/Particle)
Thermal Correction to Energy =	0.464922
Thermal Correction to Enthalpy =	0.465866
Thermal Correction to Free Energy =	0.374473
Sum of electronic and zero-point Energies =	-1735.510520
Sum of electronic and thermal Energies =	-1735.482048
Sum of electronic and thermal Enthalpies =	-1735.481103
Sum of electronic and thermal Free Energies =	-1735.572496

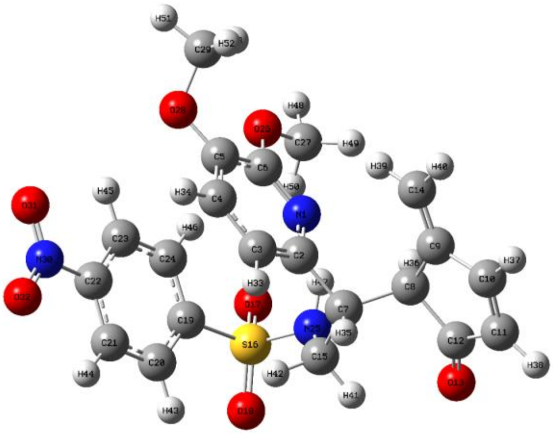

20a

7	1.58094462	-0.42651894	0.99546285
6	-0.92094295	-0.87809810	1.20651856
6	-2.60498687	1.01038295	1.87600381
6	-1.66254888	3.43310445	2.38661542
6	0.89650367	3.89978818	2.18408286
6	2.45846411	1.86563611	1.42118912
6	-1.76751300	-3.54519968	0.58897900
6	-0.16136257	-5.52420345	2.03606774
6	-0.40907680	-5.29391383	4.89002553
6	-1.62907052	-7.61169537	5.81030240
6	-2.03516857	-9.28750375	3.94540901
6	-1.10217762	-8.23132003	1.52693969
8	-1.07994845	-9.29212255	-0.50135995
6	0.34379671	-3.41312790	6.40214280
6	-1.79510022	-3.94124866	-2.30027146
16	0.92860739	-1.86504038	-6.05207809
8	3.61987891	-1.46848619	-6.45657389
8	-0.70953069	-3.06180442	-7.89908481
6	-0.43520730	1.17532116	-5.40409131
6	-2.98198997	1.58394457	-5.94309887
6	-4.08083378	3.87875216	-5.27930191
6	-2.57780757	5.68614167	-4.10925767
6	-0.02253224	5.33223114	-3.61774468
6	1.05938939	3.03076350	-4.28292378
7	0.71321074	-3.49311855	-3.40462779
8	4.92219841	2.37058715	1.05826348
6	6.46531454	0.36195225	0.12921702
8	1.82118605	6.28177145	2.46365620
6	3.52139882	6.63518766	4.52766238
7	-3.75846752	8.09226062	-3.33164343
8	-2.40874053	9.66585766	-2.33820613

8	-6.01602992	8.33813176	-3.71163264
1	-4.61247018	0.61318106	2.01774048
1	-2.90203068	4.98599881	2.90525026
1	-3.73213687	-3.75293199	1.22337162
1	1.81563531	-5.37802235	1.43571196
1	-2.08604274	-7.88152609	7.79347004
1	-2.86754301	-11.15222802	4.08684621
1	1.29317198	-1.73972487	5.69356265
1	0.03680550	-3.53167666	8.43019453
1	-2.39821096	-5.85505237	-2.77265566
1	-3.17910682	-2.61239206	-3.08765693
1	-4.06560971	0.12762504	-6.89910543
1	-6.05500351	4.27632034	-5.65012189
1	1.06535661	6.80969157	-2.70276784
1	3.05453241	2.68125664	-3.94704836
1	2.02047688	-2.83681191	-2.14679237
1	8.36413179	1.14718613	0.01663693
1	6.42049181	-1.25018185	1.41985579
1	5.83808090	-0.24083110	-1.74931337
1	4.03713681	8.62890975	4.50079205
1	2.57031460	6.18587717	6.31181444
1	5.21016587	5.46691888	4.30357054

Frequencies

14.5703	27.6008	34.3042	34.6171	44.0502	44.5975
51.5748	54.6282	81.3453	85.3394	98.6116	117.7091
122.4136	139.1066	145.0934	153.2177	163.9718	180.1629
195.4498	210.2126	220.9233	240.9071	244.2333	259.2636
266.6652	270.1673	291.9966	303.9465	343.0763	350.9122
360.3294	372.0450	380.6629	427.8852	434.5525	454.2746
462.4588	474.3535	482.8905	522.0453	528.1881	543.0432
555.4873	564.8548	576.8331	582.7119	596.1136	615.2509
634.2961	634.7995	671.0815	677.4758	705.3735	716.4660
720.5275	740.9386	759.4398	762.0403	773.0595	796.6897
820.2303	854.1197	864.0888	878.6466	887.4741	894.5562
901.8673	916.5013	937.1430	950.3432	962.0173	966.6799
986.9152	993.6976	1011.6957	1015.4810	1025.2264	1035.9095

1058.6859	1074.4759	1094.3019	1100.2388	1109.9512	1119.9880
1135.6262	1145.1553	1152.0387	1156.5905	1177.3525	1188.0625
1189.5156	1196.3427	1206.9543	1210.3815	1212.1474	1226.2992
1236.8726	1255.4418	1284.4869	1307.7560	1314.5671	1319.7839
1320.6886	1331.0761	1356.1848	1360.5136	1374.7883	1381.2034
1392.1597	1417.9473	1453.3468	1458.3359	1469.6198	1478.3107
1485.6123	1497.2759	1513.1819	1517.1568	1519.2939	1530.0927
1531.8196	1532.6395	1533.8585	1552.7690	1657.9213	1668.9452
1672.2395	1673.5477	1680.9710	1733.2859	1749.9546	1855.6670
3076.1118	3077.0704	3085.2991	3086.5733	3111.1699	3158.5218
3161.9905	3178.2646	3191.8192	3203.4212	3212.6973	3229.7692
3237.2513	3238.8241	3242.3447	3246.1683	3262.3956	3272.1076
3281.3611	3283.4034	3512.9664			

E(RM062X) =	-1901.08816231
Zero-point correction=	0.411761 (Hartree/Particle)
Thermal correction to Energy=	0.440864
Thermal correction to Enthalpy=	0.441808
reThermal Correction to Fe Energy =	0.349414
Sum of electronic and zero-point Energies =	-1900.676401
Sum of electronic and thermal Energies=	-1900.647299
Sum of electronic and thermal Enthalpies=	-1900.646355
Sum of electronic and thermal Free Energies =	-1900.738748

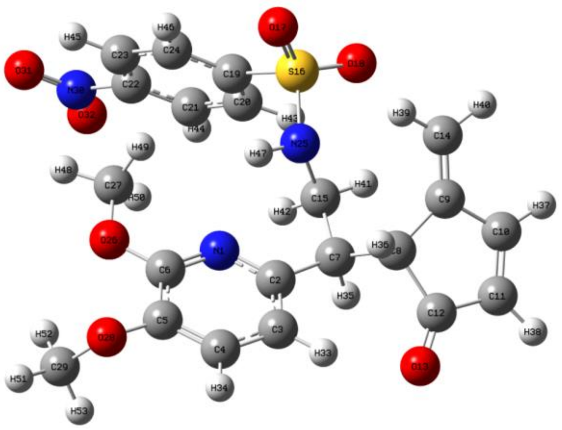

20b

7	2.66536933	0.28992138	0.62143202
6	2.45917261	-1.24385164	2.64536453
6	4.57156632	-1.98923530	4.01126612
6	6.93929359	-1.07228042	3.27372470
6	7.15311533	0.53233204	1.22427516
6	4.90303552	1.14279049	-0.07090496
6	-0.22557248	-2.02044024	3.38882549
6	-0.40983695	-4.88297565	4.15736543
6	-2.90557413	-6.20553745	3.61314147
6	-4.21398535	-6.54860463	6.03373400
6	-2.75070567	-5.90683299	8.01088498
6	-0.26639786	-5.04247820	7.07365095
8	1.54362268	-4.47979499	8.36496268
6	-3.75582266	-7.14725296	1.42847155
6	-2.16502492	-1.15928914	1.42250428
16	-3.89579402	-2.15735912	-3.23332186
8	-2.93997847	-3.54282164	-5.40296916
8	-6.28061571	-2.77725428	-2.01891508
6	-4.05010342	1.10321752	-4.14139576
6	-5.39035340	2.79299991	-2.62835083
6	-5.38556194	5.34871773	-3.24741817
6	-4.05074523	6.11029415	-5.37659484
6	-2.75010283	4.43934658	-6.93009546
6	-2.75256910	1.88805492	-6.29414622
7	-1.68137384	-2.33417078	-1.03850485
8	5.10083646	2.66132147	-2.11114966
6	2.86646771	3.06104644	-3.54224226
8	9.45623464	1.35319394	0.42272473
6	9.87015268	4.00489531	0.66774846
7	-4.03082108	8.82533705	-6.03274310
8	-2.81884944	9.45761370	-7.88190004
8	-5.21962425	10.25066249	-4.67589976

1	4.37626878	-3.20260571	5.65142325
1	8.64642621	-1.59779624	4.28471501
1	-0.68980867	-0.91029502	5.08505441
1	1.17023926	-5.91839014	3.31451084
1	-6.11136539	-7.32565000	6.14124808
1	-3.19228566	-6.03821330	10.00509768
1	-2.69805993	-6.98293675	-0.31776414
1	-5.57020921	-8.10689655	1.34778616
1	-4.05880389	-1.72307005	2.01883789
1	-2.08292788	0.91040237	1.28724499
1	-6.45055172	2.10607331	-1.01145283
1	-6.39702333	6.73278972	-2.12740137
1	-1.77687925	5.13662359	-8.59196605
1	-1.78521795	0.50253400	-7.45822299
1	0.03988027	-1.83000071	-1.75082112
1	3.38773013	4.38886677	-5.02843864
1	2.22551897	1.28143427	-4.38577339
1	1.34811297	3.83827099	-2.37115915
1	11.79108205	4.34425336	0.00538682
1	8.53037914	5.07350965	-0.48624498
1	9.70804411	4.56952605	2.65441306

Frequencies

12.4832	22.4699	24.0384	37.3695	39.1510	49.3321
55.5548	70.4547	79.6530	85.3358	95.9219	107.0489
125.9175	132.8270	145.6141	163.4733	170.1971	181.4975
192.8292	206.3754	220.5044	226.2896	235.3314	253.6401
267.4670	272.6387	276.5938	307.1131	333.2723	349.4544
357.0134	369.1412	389.2282	419.2455	423.1378	449.4146
472.9510	475.9405	492.9554	507.1833	533.4106	542.3356
559.8216	575.4225	580.3420	589.4041	599.0863	619.7095
633.9720	641.1694	677.3604	692.2829	696.8557	705.8480
727.0105	752.4255	759.3097	764.6700	766.7277	796.5667
824.8965	849.1206	875.1314	880.1660	888.7135	890.2910
891.5869	897.0251	925.2975	955.5501	968.3028	986.1433
994.9412	1008.9676	1011.4824	1013.9670	1024.6873	1036.1875

1063.9717	1079.3402	1095.7034	1103.0577	1109.3561	1120.4682
1132.7590	1150.0464	1153.3852	1168.0536	1184.0096	1187.8128
1192.7889	1195.9693	1206.3985	1210.9941	1216.5959	1223.4163
1241.2522	1259.5054	1276.2142	1306.6000	1310.6134	1319.8483
1322.2884	1343.2638	1354.7194	1359.2664	1377.6843	1382.6428
1386.7680	1403.4687	1449.4632	1453.5997	1465.1631	1468.1546
1479.9868	1486.0425	1505.5139	1510.0241	1514.9320	1516.1944
1522.4792	1532.5345	1533.3724	1550.4849	1652.6817	1667.4014
1670.8631	1672.4468	1685.5511	1737.8079	1749.5354	1843.2381
3043.5560	3067.7092	3074.0989	3075.2376	3120.8751	3145.1234
3154.8864	3169.7206	3198.9204	3199.3792	3199.8026	3238.8038
3238.9593	3244.5613	3245.6056	3259.2569	3271.6353	3272.1662
3279.5621	3297.4932	3532.1789			

E(RM062X) =	-1901.07977829
Zero-point Energy Correction =	0.411591 (Hartree/Particle)
Thermal Correction to Energy =	0.440742
Thermal Correction to Enthalpy =	0.441687
Thermal Correction to Free Energy =	0.348493
Sum of electronic and zero-point Energies =	-1900.668187
Sum of electronic and thermal Energies =	-1900.639036
Sum of electronic and thermal Enthalpies =	-1900.638092
Sum of electronic and thermal Free Energies =	-1900.731285

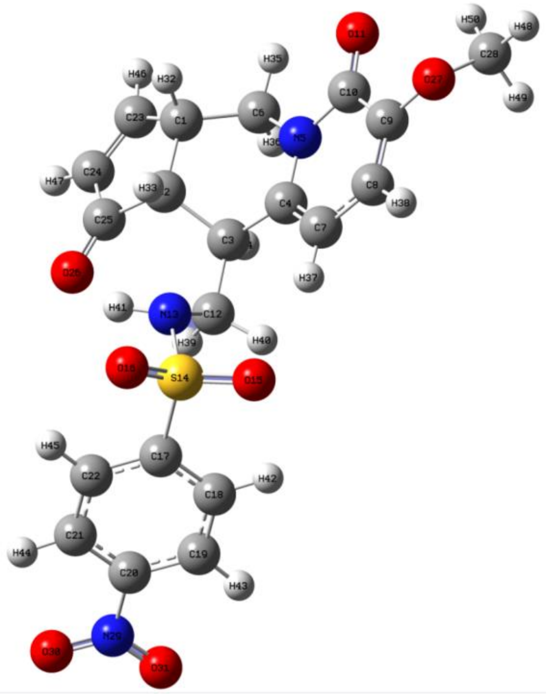

16c

6	-1.00613937	-4.32073834	4.72946519
6	-1.91025941	-2.56263330	2.58731894
6	0.23328244	-0.75614474	1.75657197
6	2.42141594	-2.40567748	0.95638361
7	3.19847093	-4.08548651	2.80438723
6	1.84579082	-4.08238289	5.21767740
6	3.54443111	-2.42732191	-1.35559307
6	5.54951494	-4.16480400	-1.80875360
6	6.33322927	-5.81122273	0.00461299
6	5.11194395	-5.89200211	2.47915289
8	5.64959182	-7.38269935	4.17693437
6	-0.61220351	1.20520195	-0.19660857
7	-2.23504981	0.07928935	-2.13409376
16	-2.13763390	1.26126334	-5.02951860
8	0.50655436	1.28697308	-5.78352461
8	-4.07692852	-0.05619116	-6.44978948
6	-3.09878303	4.49652911	-4.74842441
6	-1.26366202	6.37167060	-4.54213537
6	-2.02029616	8.87182707	-4.22876211
6	-4.59094154	9.39075580	-4.13621949
6	-6.44078473	7.53865792	-4.35734177
6	-5.67408985	5.04532301	-4.67139810
6	-2.59364519	-3.53268986	6.96670383
6	-4.33002169	-1.77524092	6.40476631
6	-4.15543523	-1.13132184	3.69076807

8	-5.51595182	0.28327923	2.49237957
8	8.14197821	-7.55122378	-0.52658777
6	10.30163003	-7.55100037	1.09150787
7	-5.39894334	12.04333209	-3.79700333
8	-7.65875435	12.46149772	-3.74055529
8	-3.75050860	13.63345567	-3.59224192
1	-1.40143313	-6.30097243	4.25926035
1	-2.52295263	-3.62134075	0.91813223
1	0.82917111	0.33473977	3.42414548
1	2.57246584	-5.66378195	6.31821107
1	2.27339678	-2.33097771	6.23947836
1	2.90094477	-1.18623693	-2.85198917
1	6.47349084	-4.22330464	-3.64033198
1	-1.61762832	2.73533931	0.77063808
1	1.05267630	2.01479483	-1.11548209
1	-4.06221086	-0.11350597	-1.53158844
1	0.72269267	5.87567331	-4.66998488
1	-0.66376884	10.39745479	-4.07012999
1	-8.42027437	8.05818262	-4.29305131
1	-7.05222715	3.54041653	-4.88749398
1	-2.31909331	-4.36270187	8.82597167
1	-5.70757623	-0.93478787	7.66536769
1	11.59952822	-8.93353910	0.28607413
1	11.19221772	-5.67934604	1.07861728
1	9.78278504	-8.08637600	3.01408052

Frequencies

12.8716	13.8594	24.8919	45.9316	52.8232	53.9518
58.2210	63.5973	76.3260	94.3497	104.0133	117.9854
145.3714	153.7917	176.5041	187.5402	207.8872	228.0840
236.8500	251.1944	259.7364	268.5846	309.2913	319.2509
336.7533	342.7637	354.9602	386.7868	403.1805	408.0608
420.0944	454.1864	468.5272	473.7005	488.4392	496.6118
520.8473	533.9966	543.6694	549.5805	564.9988	588.2311
607.8398	634.7908	637.1842	668.0986	671.5962	702.3395
707.2839	733.6394	743.5580	751.1289	763.5343	768.5074
771.3807	777.6112	829.1735	856.1240	865.0694	871.0006
887.7286	889.6877	910.2275	931.8337	956.6815	963.2389

966.8857	1007.2217	1010.1309	1014.4951	1021.1578	1035.5435
1039.8401	1058.6291	1083.3725	1098.3577	1105.0263	1118.5793
1128.0734	1132.8159	1149.8503	1156.9946	1175.5145	1181.1544
1187.7564	1196.0006	1206.6391	1212.3673	1220.3979	1221.9023
1245.5160	1266.9631	1284.4662	1297.0304	1305.3733	1319.7673
1329.0673	1346.1959	1353.2458	1358.5623	1367.2132	1372.2693
1378.7326	1389.8121	1397.4524	1409.1514	1424.8453	1454.1845
1467.9723	1475.8709	1480.0921	1497.5915	1512.8756	1517.7240
1533.1025	1536.3392	1540.5345	1651.5974	1671.8965	1674.2475
1680.4938	1710.1535	1738.2026	1766.6243	1835.0242	3042.0570
3072.9924	3080.5759	3092.1374	3096.4433	3119.4553	3157.0413
3168.3229	3196.2734	3206.1538	3231.2943	3236.4972	3244.9352
3248.5672	3265.4068	3273.5764	3273.9889	3276.0184	3501.2083

E(RM062X) =	-1861.82977274
Zero-point Energy Correction =	0.386831 (Hartree/Particle)
Thermal Correction to Energy =	0.413321
Thermal Correction to Enthalpy =	0.414265
Thermal Correction to Free Energy =	0.326405
Sum of electronic and zero-point Energies =	-1861.442941
Sum of electronic and thermal Energies =	-1861.416452
Sum of electronic and thermal Enthalpies =	-1861.415508
Sum of electronic and thermal Free Energies =	-1861.503367

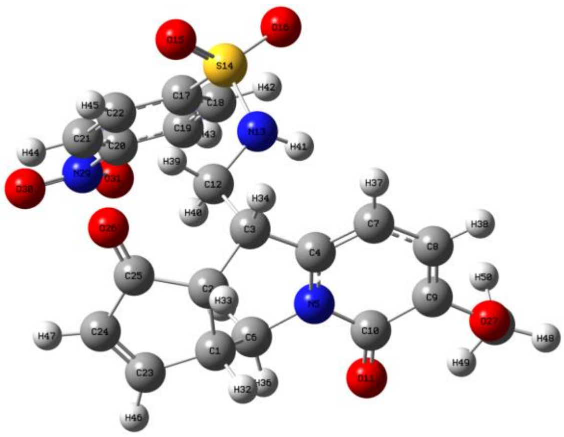

22

6	-2.52028503	0.46060726	5.33022308
6	-3.46171767	2.73839927	3.76916039
6	-1.64634156	3.60013329	1.64917803
6	1.06581113	3.31697584	2.50507425
7	1.74633965	1.06044089	3.62641393
6	-0.21580778	-0.82497124	4.14658881
6	2.84014782	5.14509380	2.13623269
6	5.38198776	4.70592886	2.89864343
6	6.06177333	2.48741805	4.00779414
6	4.19651859	0.50563137	4.49088605
8	4.65625209	-1.49987564	5.56221211
6	-2.12628238	2.25178566	-0.89306891
7	-0.24500375	3.06973920	-2.73680909
16	-0.79705327	2.57065767	-5.77929511
8	-3.23679649	3.68180826	-6.34953166
8	1.51578857	3.28043330	-7.07430299
6	-1.15885885	-0.78243086	-6.09402299
6	0.98208613	-2.26181219	-6.49862928
6	0.72261351	-4.87337275	-6.64704922
6	-1.67968225	-5.90179038	-6.38008191
6	-3.83084542	-4.44741147	-5.99633249
6	-3.56085484	-1.83435662	-5.85443442
6	-4.76946382	-1.29274214	5.43601859
6	-6.74050643	-0.47666194	4.07711022
6	-6.10534291	1.96449173	2.85768456
8	-7.40265124	3.16256174	1.40271326
8	8.45381958	2.16131885	4.86167890
6	9.76270045	-0.03697333	3.99485146
7	-1.96037939	-8.67792001	-6.52355301
8	-4.07450419	-9.54233857	-6.25832447
8	-0.05787424	-9.91541795	-6.89001676
1	-1.97980730	1.04751098	7.24385224

1	-3.74267304	4.35658368	5.03232373
1	-1.95174863	5.61061877	1.28307405
1	-0.72682771	-1.80361184	2.39561633
1	0.61699882	-2.21368709	5.42029515
1	2.30144210	6.90347189	1.23472423
1	6.82256227	6.14089211	2.62173997
1	-3.97846959	2.83115482	-1.59871067
1	-2.13702929	0.18426036	-0.66954062
1	1.59066911	2.68987002	-2.29290641
1	2.81393586	-1.36744735	-6.73356522
1	2.32871347	-6.10092219	-6.97346575
1	-5.66014922	-5.35232076	-5.82910830
1	-5.19170095	-0.61492183	-5.60031119
1	-4.71607116	-3.04052677	6.51386581
1	-8.55221030	-1.39809598	3.82675671
1	11.70261359	0.18255365	4.65200523
1	8.92368966	-1.75087155	4.77288087
1	9.74046956	-0.09987204	1.92345354

Frequencies

5.9863	12.0104	24.9781	27.9356	31.9572	44.0855
58.7523	59.5321	72.3179	83.9580	108.0195	123.0088
144.2471	150.8144	161.2815	177.8498	195.9274	216.9340
232.4315	233.9184	248.7672	267.8571	280.6996	321.0911
329.8673	341.6127	366.8256	378.9984	396.6194	407.6606
420.6858	453.4012	463.0868	473.8262	484.8024	490.9370
503.1466	539.3699	542.3791	552.2580	558.3825	588.6296
597.7757	627.2580	633.0050	638.6795	650.2531	692.2472
703.5047	706.6937	747.7811	754.6935	758.6660	763.3587
774.5827	779.8675	812.7023	842.0625	850.3294	872.4529
874.7672	889.2274	892.6851	923.3500	954.9197	960.9160
1000.7420	1002.1336	1010.8795	1018.1435	1022.0630	1029.2876
1036.4063	1044.3362	1090.8941	1092.2316	1111.5335	1120.2199
1127.5419	1133.0900	1144.8895	1149.7271	1177.7849	1184.7210
1186.5313	1192.7645	1206.4507	1207.3710	1217.3447	1220.9607

1246.1201	1256.7495	1271.3759	1286.3601	1305.7320	1320.1331
1325.1228	1334.2820	1346.0671	1357.4706	1359.0758	1366.6981
1381.0752	1389.2540	1390.6768	1402.1464	1421.1215	1453.1219
1466.2266	1479.7196	1480.0510	1500.2614	1513.0093	1528.2186
1532.9960	1539.5830	1547.9235	1648.5460	1671.4077	1673.1217
1688.4974	1708.5530	1738.7172	1769.4752	1859.0081	3047.1063
3074.8167	3080.6583	3096.4453	3106.8144	3132.1521	3167.5520
3172.8082	3188.2885	3211.7549	3232.7462	3238.4188	3244.6298
3245.0316	3259.5416	3272.3233	3274.0289	3274.4142	3551.9301

E(RM062X) =	-1861.82227691
Zero-point Energy Correction =	0.386004 (Hartree/Particle)
Thermal Correction to Energy =	0.412905
Thermal Correction to Enthalpy =	0.413849
Thermal Correction to Free Energy =	0.323379
Sum of electronic and zero-point Energies =	-1861.436273
Sum of electronic and thermal Energies =	-1861.409372
Sum of electronic and thermal Enthalpies =	-1861.408428
Sum of electronic and thermal Free Energies =	-1861.498898

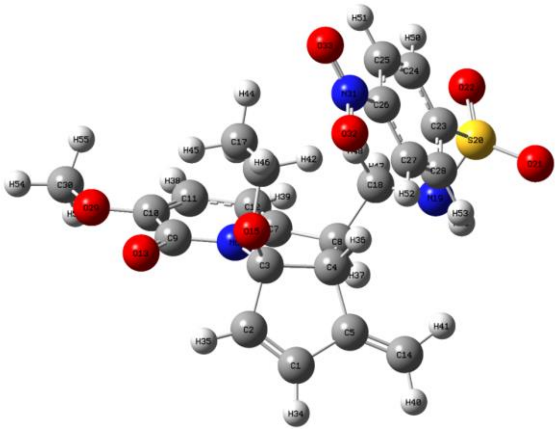

18

6	−6. 58673797	2. 80893273	0. 30385947
6	−6. 14266484	0. 42149689	−0. 39842666
6	−3. 37238722	−0. 22085236	−0. 17169180
6	−2. 11049908	2. 21729827	0. 87916142
6	−4. 26241073	4. 10844015	1. 08087068

7	-2.87879868	-2.13122433	1.81137805
6	-1.47058489	-1.23292192	3.80376420
6	-0.81989211	1.51695311	3.40054008
6	-3.86530927	-4.56855160	1.62365211
6	-3.12964612	-6.21985212	3.74172459
6	-1.69855710	-5.34477767	5.69964863
6	-0.85262810	-2.77503524	5.75207582
8	-5.19304400	-5.21347444	-0.14729294
6	-4.04046939	6.51382650	1.81659186
8	-2.51436732	-0.99689543	-2.53921496
6	0.03316096	-1.80708825	-2.72485784
6	0.42363366	-2.72494092	-5.40963147
6	2.04727238	1.89911561	3.32963108
7	2.60314507	4.54807780	2.73924105
16	5.45476373	5.17679446	1.54749816
8	5.64179565	7.91659341	1.52235482
8	7.40704448	3.62985187	2.71630016
6	5.04249123	4.00949576	-1.59841598
6	6.56718265	2.03264823	-2.42005854
6	6.23523549	1.09762761	-4.85915811
6	4.38134636	2.18205079	-6.36272918
6	2.84426568	4.15884385	-5.56020799
6	3.19474906	5.09596125	-3.13151118
8	-4.05413647	-8.57567555	3.47984151
6	-3.49495942	-10.32241285	5.41582832
7	3.99567767	1.15856557	-8.93478216
8	2.35914193	2.13030947	-10.22275269
8	5.32950585	-0.59771125	-9.58349373
1	-8.41888820	3.73195551	0.29926242
1	-7.47754603	-0.97166793	-1.08175377
1	-0.70589685	2.90577735	-0.47407866
1	-1.60810658	2.65568520	4.94395024
1	-1.19336715	-6.58028456	7.25502049
1	0.24212468	-2.07238461	7.33452124
1	-5.67394459	7.75197043	1.92397612
1	-2.21530976	7.31606386	2.31274510
1	1.32318414	-0.22775553	-2.28768142
1	0.40185810	-3.33367121	-1.36612899
1	2.38746128	-3.30536018	-5.70676919
1	-0.81048625	-4.33709272	-5.78789771
1	-0.04858911	-1.22644833	-6.75598901
1	2.93563080	1.29861034	5.10159140
1	2.84762848	0.72934977	1.81717099
1	2.24032319	5.79515422	4.16534022

1	7.98043560	1.24678522	-1.15822254
1	7.37147801	-0.44280815	-5.58721584
1	1.41728875	4.92034882	-6.81617825
1	2.03766597	6.63556888	-2.41953785
1	-4.39543119	-12.08920661	4.86397943
1	-1.44834179	-10.60672738	5.59052367
1	-4.26249240	-9.68533874	7.23363958

Frequencies

19.7247	25.8482	35.9766	37.1703	51.9006	54.6387
62.7268	75.5693	88.8396	102.4386	112.6714	126.4059
132.5473	138.9363	148.2239	156.2723	168.7139	183.8362
200.8705	227.3717	231.2049	256.2597	262.7716	267.8293
272.5754	286.4076	298.5170	320.6269	337.5623	342.9651
352.7015	366.1783	377.5191	387.6405	409.7458	417.3340
447.3529	467.7232	468.5865	477.7534	485.6523	495.8454
510.0640	542.7265	552.7243	573.2800	578.8644	601.7807
617.7707	632.9558	638.5334	652.9860	677.4297	690.9325
700.1472	709.7337	723.0197	750.8962	764.3908	776.9801
786.3354	809.7090	826.6045	831.0466	850.8549	861.4104
869.5278	883.3856	890.9237	894.4445	912.9080	915.6638
930.8702	937.5551	952.0673	983.9282	1007.7704	1008.4194
1022.4854	1035.6480	1062.8658	1076.6263	1081.7981	1101.6265
1108.3240	1110.5796	1119.9371	1127.2688	1132.9256	1137.2504
1150.8756	1157.8653	1174.2813	1177.7728	1188.8355	1189.0061
1202.5347	1206.2650	1213.6488	1227.3842	1246.6334	1249.6159
1270.0018	1294.1057	1307.7818	1319.2285	1320.5777	1321.9151
1337.8198	1344.7312	1358.4574	1369.5529	1372.5823	1383.1317
1402.0575	1410.8857	1431.1338	1446.2874	1450.6253	1454.9385
1462.3414	1471.8657	1481.6661	1500.4153	1508.2752	1516.5870
1521.6751	1527.6236	1531.0351	1533.9689	1544.2667	1650.9247
1672.5095	1673.7602	1689.3358	1717.3137	1740.3701	1756.0907

1797.9930	3025.6802	3056.4283	3079.3869	3080.6391	3085.9571
3087.4527	3119.6180	3128.1013	3133.2646	3161.2684	3174.4903
3187.2426	3204.6245	3237.1122	3242.3117	3243.7803	3253.8643
3255.5203	3271.1460	3273.3200	3275.4971	3289.2731	3540.6378

E(RM062X) =	-1940.38777401
Zero-point correction=	0.441583 (Hartree/Particle)
Thermal correction to Energy=	0.470993
Thermal correction to Enthalpy=	0.471937
Thermal correction to Gibbs Free Energy=	0.380407
Sum of electronic and zero-point Energies=	-1939.946191
Sum of electronic and thermal Energies=	-1939.916781
Sum of electronic and thermal Enthalpies=	-1939.915837
Sum of electronic and thermal Free Energies=	-1940.007367

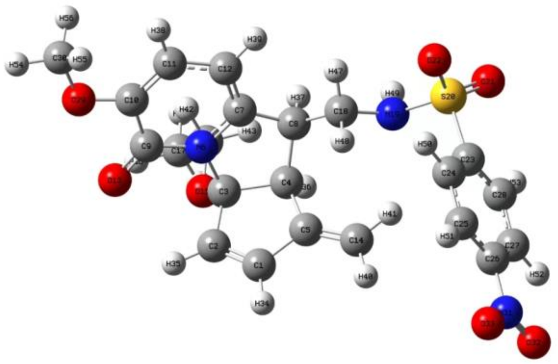

21

6	−2. 26786974	−1. 47141474	−4. 44243148
6	−4. 22339610	−2. 33079657	−3. 09701746
6	−4. 32359420	−1. 08155324	−0. 53758809
6	−2. 23837149	1. 00961557	−0. 61172193
6	−0. 87872224	0. 52666952	−3. 11038017
7	−3. 41530791	−2. 81831969	1. 46529005
6	−1. 35477424	−1. 92106069	2. 76969955
6	−0. 75805293	0. 73677045	1. 90517300
6	−4. 55960539	−5. 16168155	1. 84703343
6	−3. 35121829	−6. 66357592	3. 85552680
6	−1. 29598677	−5. 78612915	5. 13908142
6	−0. 25827829	−3. 34685026	4. 59327331
8	−6. 37154676	−5. 85404196	0. 60120894
6	1. 08329066	1. 81243529	−4. 05029750

8	-6.77385743	-0.24000770	-0.07780043
6	-7.23358200	0.88362632	2.31352325
6	-10.01711522	1.54366502	2.42890517
6	2.07246446	1.26695123	1.91003546
7	2.52578971	3.98744912	1.56948762
16	5.53766993	4.94007823	1.77567628
8	5.42156167	7.61501984	2.39344947
8	6.99739999	3.16074335	3.28074279
6	6.56012505	4.61215913	-1.42002894
6	7.66934412	2.35238188	-2.17978005
6	8.32114336	2.04462933	-4.71050145
6	7.84777427	4.02530516	-6.36440192
6	6.77583450	6.30484269	-5.61542397
6	6.11860442	6.59917253	-3.08925504
8	-4.49565133	-8.91111746	4.18785328
6	-3.48008029	-10.52626419	6.05127618
7	8.53327336	3.70061853	-9.05493387
8	8.12608069	5.46627675	-10.46943216
8	9.45345943	1.68723597	-9.67554258
1	-1.74694509	-2.09526387	-6.32614549
1	-5.59082117	-3.76359708	-3.60772168
1	-3.16045078	2.85780475	-0.71625619
1	-1.63416393	2.02938676	3.28218830
1	-0.41372501	-6.92360177	6.59802627
1	1.35108367	-2.65858169	5.65383855
1	1.84269791	1.35158745	-5.90321392
1	1.93584507	3.36899309	-3.02565360
1	-6.73185986	-0.44449589	3.82807460
1	-6.06503339	2.59268128	2.53507739
1	-10.47420634	2.40977500	4.24966479
1	-10.50849476	2.86749710	0.92070598
1	-11.15320750	-0.16561299	2.19864989
1	2.91066229	0.60847936	3.68375541
1	2.98474218	0.25264379	0.35379440
1	1.47649286	5.11501017	2.73185622
1	8.02911331	0.87184870	-0.80627466
1	9.18176879	0.31941347	-5.39998528
1	6.47377031	7.79210092	-6.99000331
1	5.27442919	8.34013242	-2.40722686
1	-4.64903068	-12.22042289	6.03293889
1	-1.51736411	-11.02725067	5.60843106
1	-3.56210277	-9.64136041	7.92478160

Frequencies

9.6013	17.5786	26.0966	36.5884	41.7877	51.1644
60.1492	64.4357	68.3215	84.2742	96.8849	105.8096
135.0672	140.8452	144.1355	158.4570	168.0471	181.3130
213.2895	231.2046	241.7827	247.3511	251.0627	265.5778
270.9213	273.9815	289.5496	321.1793	331.2242	349.7910
356.0638	359.8749	380.7709	389.9431	396.3280	419.2039
441.1469	458.7158	465.3753	479.2829	488.5713	502.7889
523.0372	536.9414	543.8115	560.3952	593.0401	606.3900
615.3246	628.0453	634.0413	665.5472	680.7752	696.4812
703.2150	716.7927	725.8367	750.8919	760.4333	768.3431
785.6593	796.6117	821.7317	831.7156	853.1395	869.7387
872.2414	883.9117	887.5181	892.4838	912.1202	913.9543
934.1717	945.2282	949.4186	968.3584	1003.9911	1008.0990
1019.6980	1036.1339	1049.3958	1062.3058	1080.1276	1101.4132
1111.7819	1118.2908	1123.5708	1133.5388	1139.3303	1150.5281
1151.5654	1166.7373	1173.3833	1180.7753	1188.2593	1192.1516
1203.8300	1207.3045	1218.7353	1229.2012	1250.7812	1257.3193
1271.2302	1293.6542	1311.4828	1314.2077	1320.3920	1321.1429
1344.9499	1357.4188	1358.6087	1372.4791	1376.5018	1384.0356
1410.0545	1415.3060	1430.9313	1450.1256	1452.0455	1457.8298
1460.6054	1472.1862	1480.4610	1499.8868	1507.9914	1521.1180
1523.1369	1526.6840	1530.8335	1534.1152	1543.6724	1652.4982
1672.7234	1674.6282	1695.7007	1718.4118	1739.2717	1751.1865
1798.3578	3023.8318	3027.9273	3056.8489	3083.7706	3091.9163
3097.7928	3119.8213	3120.3504	3145.5523	3168.8277	3178.1033
3192.7056	3204.8841	3244.2006	3247.4450	3248.6174	3249.0531
3263.8386	3274.7170	3275.2120	3294.8769	3298.8351	3530.7568

E(RM062X) =	-1940.38086353
Zero-point correction=	0.441479 (Hartree/Particle)
Thermal correction to Energy=	0.471184
Thermal correction to Enthalpy=	0.472128
Thermal correction to Gibbs Free Energy=	0.377774
Sum of electronic and zero-point Energies=	-1939.939385
Sum of electronic and thermal Energies=	-1939.909680
Sum of electronic and thermal Enthalpies=	-1939.908736
Sum of electronic and thermal Free Energies=	-1940.003090

7. Reference

1. P.-J. Yang, L. Qi, Z. Liu, G. Yang, Z. Chai, *J. Am. Chem. Soc.* **2018,** *140*, 17211−17217.

2. M. Su, X. Huang, C. Lei, J. Jin, *Org. Lett.* **2022**, *24*, 354-358.

3. Y. Yorinobu, Y. Tamotsu, O. Makoto, *Chem. Lett.* **1975**, *4*, 361−362.

4. H. S. Kim, M. Hong, S.-C. Lee, H.-Y. Lee, Y.-G. Suh, D.-C. Oh, J. H. Seo, H. Choi, J. Y. Kim, K.-W. Kim, J. H. Kim, J. Kim, Y.-M. Kim, S.-j. Park, H.-J. Park and J. Lee, *Eur. J. Med. Chem.*, **2015**, *104*, 157-164.

5. (*a*) T. Mukaiyama, J. I. Matsuo and H. Kitagawa, *Chem. Lett.* **2000**, *29*, 1250-1251; (*b*) G. Li, N. Gaeng, C. Piemontesi, Q. Wang and J. Zhu, *Angew. Chem. Int. Ed*., **2021**, *60*, 12392-12395.

6. I. Pouny, M. Batut, L. Vendier, B. David, S. Yi, F. Sautel, P. B. Arimondo and G. Massiot, *Phytochemistry.*, **2014**, *107*, 97-101.

7. J. Ouyang, R. Yan, X. Mi and R. Hong, *Angew. Chem. Int. Ed*., **2015**, *54*, 10940-10943.

8. Gaussian 16, Revision C.01, M. J. Frisch, G. W. Trucks, H. B. Schlegel, G. E. Scuseria, M. A. Robb, J. R. Cheeseman, G. Scalmani, V. Barone, G. A. Petersson, H. Nakatsuji, X. Li, M. Caricato, A. V. Marenich, J. Bloino, B. G. Janesko, R. Gomperts, B. Mennucci, H. P. Hratchian, J. V. Ortiz, A. F. Izmaylov, J. L. Sonnenberg, D. Williams-Young, F. Ding, F. Lipparini, F. Egidi, J. Goings, B. Peng, A. Petrone, T. Henderson, D. Ranasinghe, V. G. Zakrzewski, J. Gao, N. Rega, G. Zheng, W. Liang, M. Hada, M. Ehara, K. Toyota, R. Fukuda, J. Hasegawa, M. Ishida, T. Nakajima, Y. Honda, O. Kitao, H. Nakai, T. Vreven, K. Throssell, J. A. Montgomery, Jr., J. E. Peralta, F. Ogliaro, M. J. Bearpark, J. J. Heyd, E. N. Brothers, K. N. Kudin, V. N. Staroverov, T. A. Keith, R. Kobayashi, J. Normand, K. Raghavachari, A. P. Rendell, J. C. Burant, S. S. Iyengar, J. Tomasi, M. Cossi, J. M. Millam, M. Klene, C. Adamo, R. Cammi, J. W. Ochterski, R. L. Martin, K. Morokuma, O. Farkas, J. B. Foresman, and D. J. Fox, Gaussian, Inc., Wallingford CT, 2016.

8. NMR spectra:

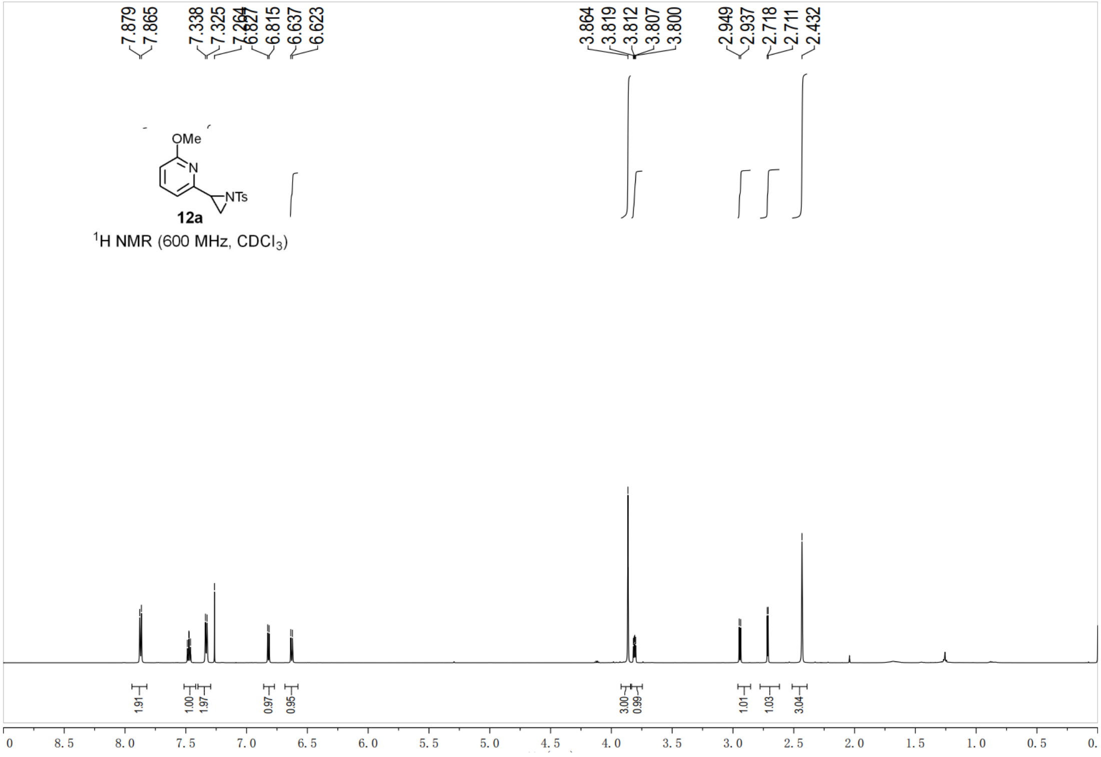

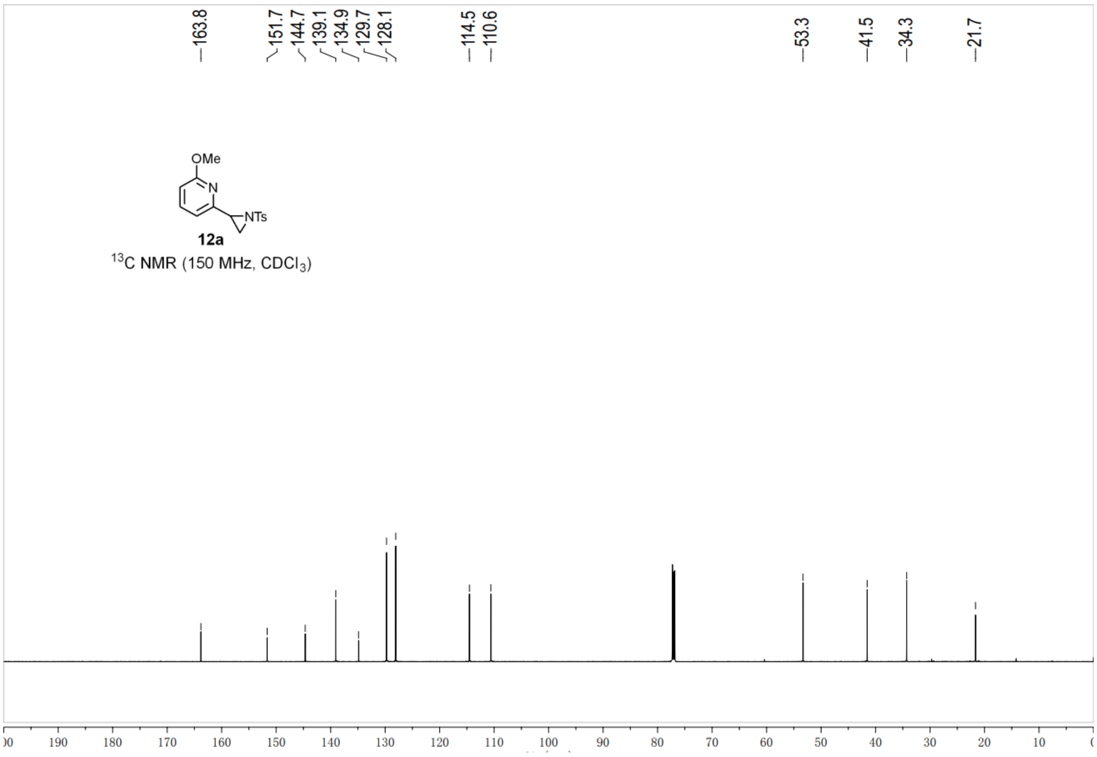

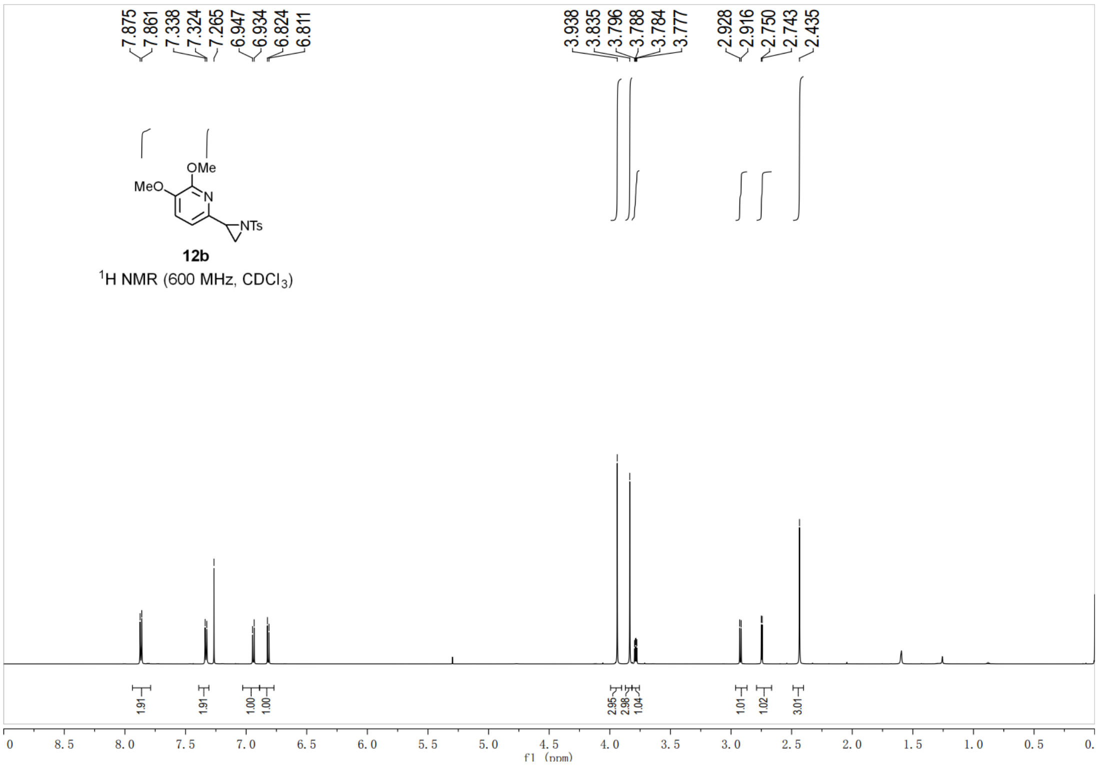
7.875
7.861
7.338
7.324
7.265
6.947
6.934
6.824
6.811
3.938
3.835
3.796
3.788
3.784
3.777
2.928
2.916
2.750
2.743
2.435
OMe
MeO
N
NTs
12b
1H NMR (600 MHz, CDCl3)
1.91
1.91
1.00
1.00
2.95
2.98
1.04
1.01
1.02
3.01
f1 (ppm)

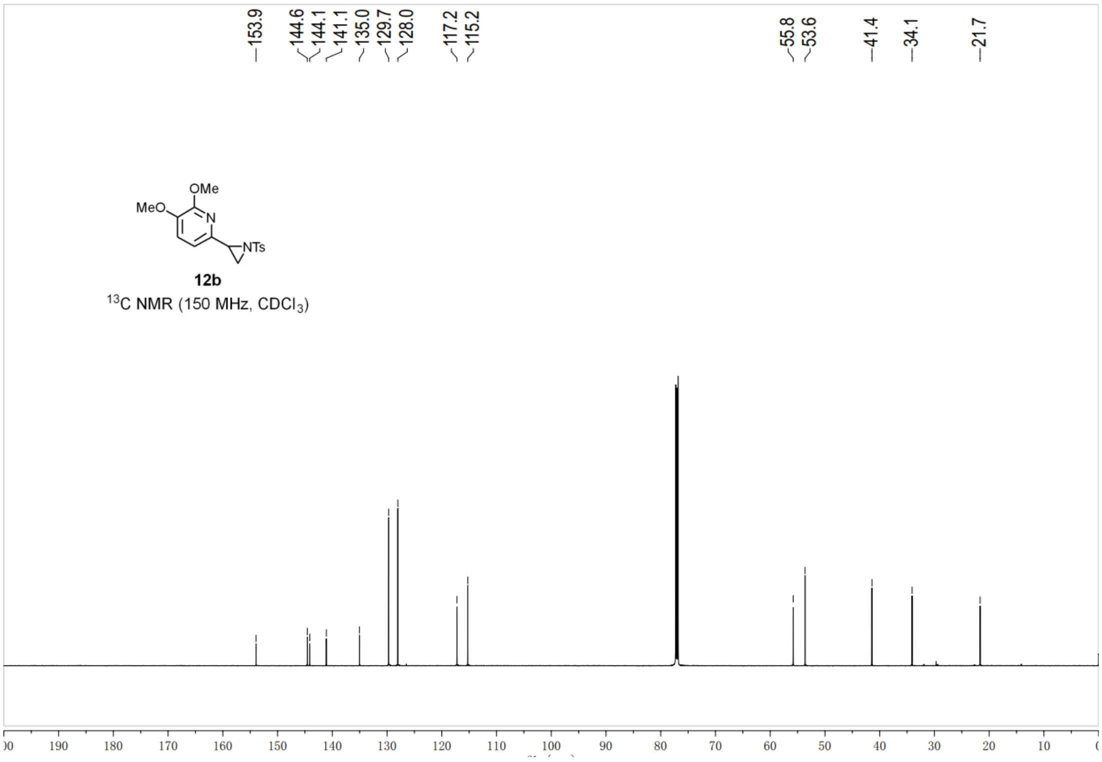
153.9
144.6
144.1
141.1
135.0
129.7
128.0
117.2
115.2
55.8
53.6
41.4
34.1
21.7
OMe
MeO
N
NTs
12b
13C NMR (150 MHz, CDCl3)

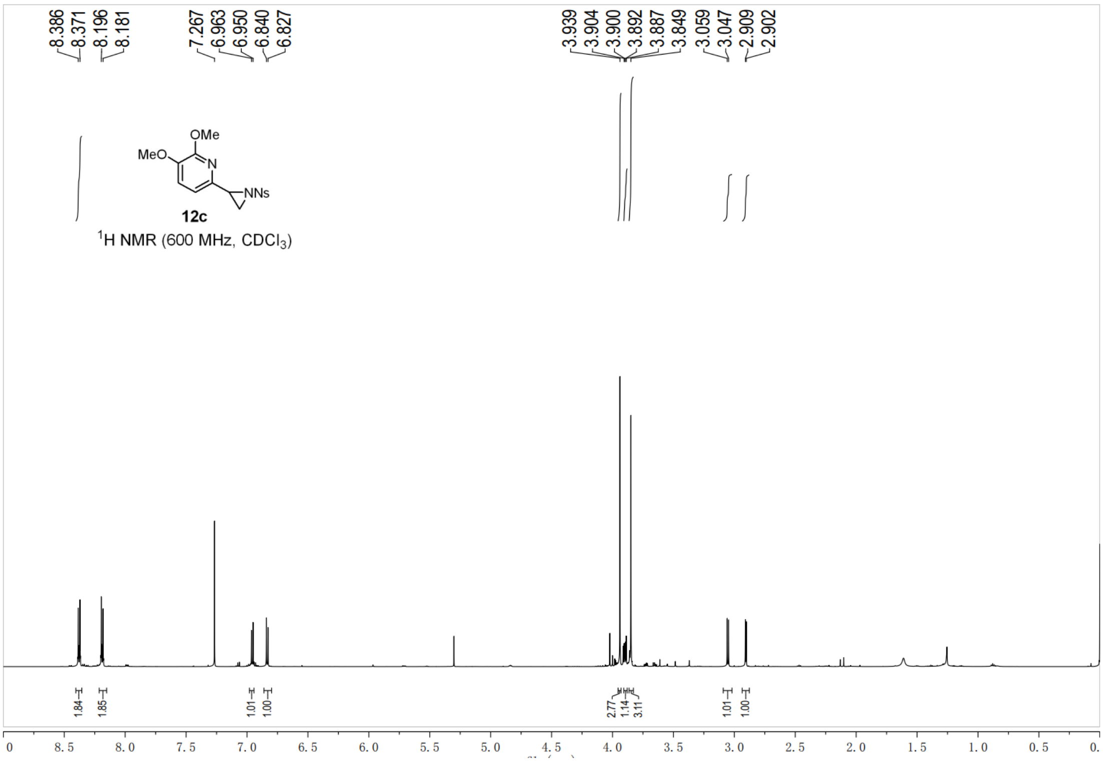
8.386
8.371
8.196
8.181
7.267
6.963
6.950
6.840
6.827
3.939
3.904
3.900
3.892
3.887
3.849
3.059
3.047
2.909
2.902
OMe
MeO
N
NNs
12c
^{1}H NMR (600 MHz, CDCl$_3$)
1.84
1.85
1.01
1.00
2.77
1.14
3.11
1.01
1.00
8.5
8.0
7.5
7.0
6.5
6.0
5.5
5.0
4.5
4.0
3.5
3.0
2.5
2.0
1.5
1.0
0.5

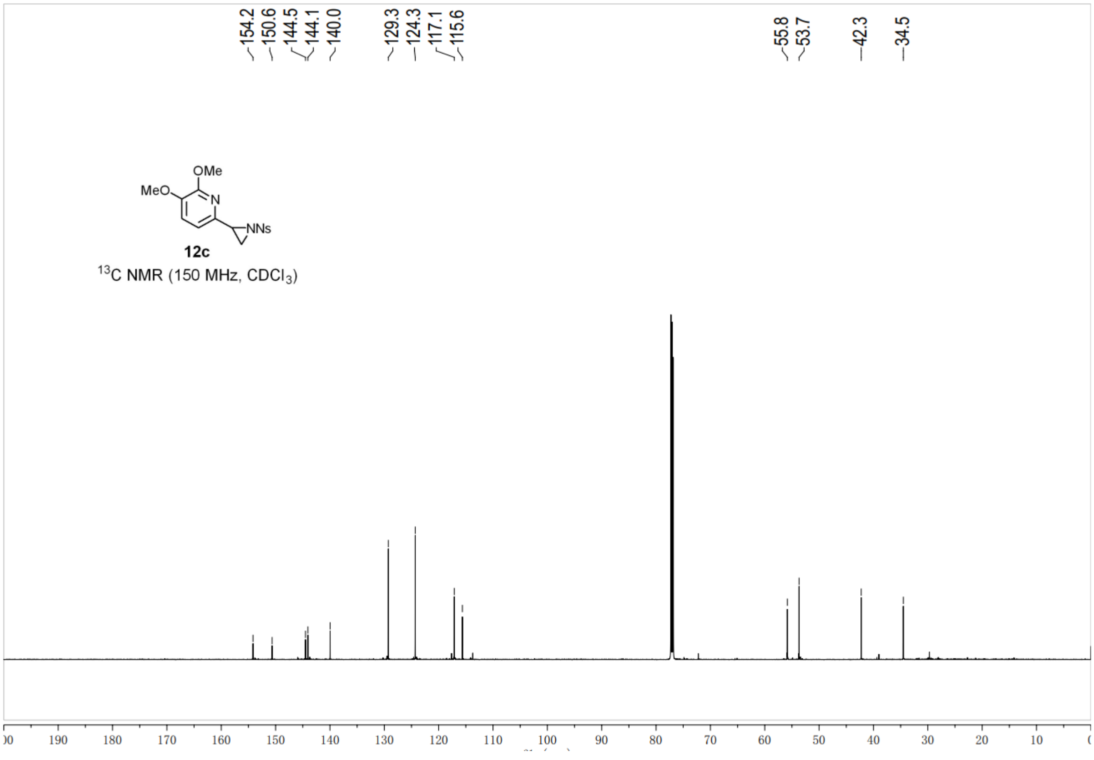
154.2
150.6
144.5
144.1
140.0
129.3
124.3
117.1
115.6
55.8
53.7
42.3
34.5
OMe
MeO
N
NNs
12c
^{13}C NMR (150 MHz, CDCl$_3$)
190
180
170
160
150
140
130
120
110
100
90
80
70
60
50
40
30
20
10

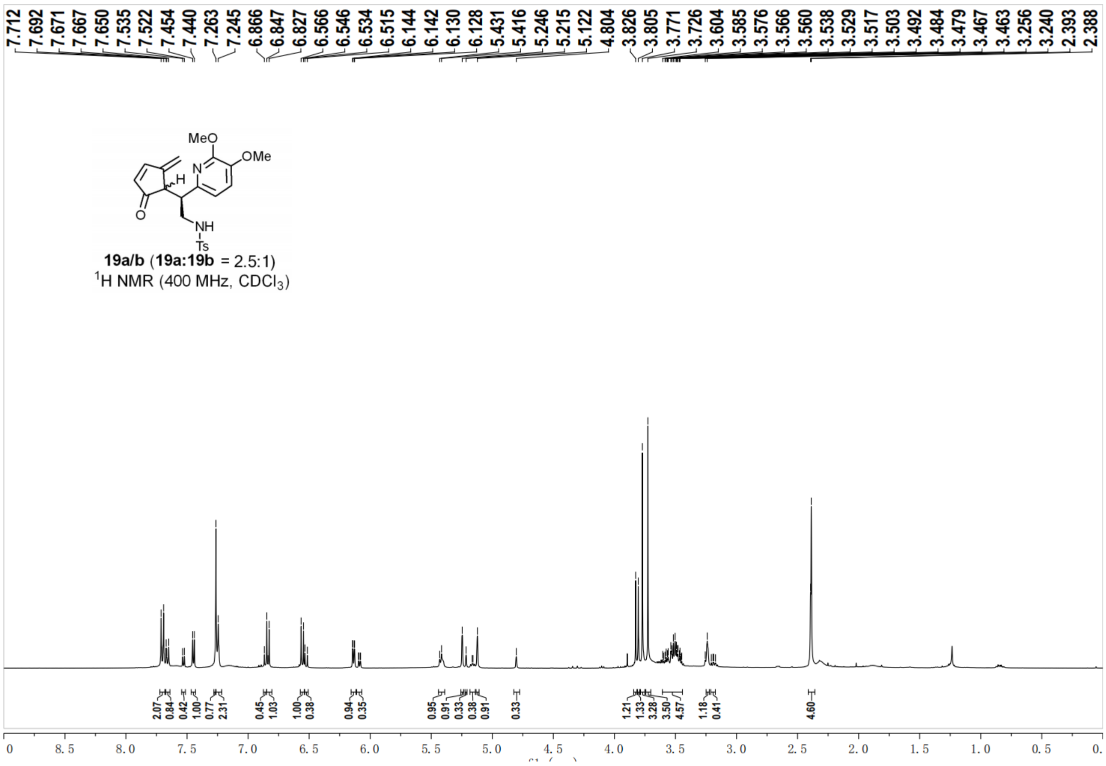
7.712 7.692 7.671 7.667 7.650 7.535 7.522 7.454 7.440 7.263 7.245 6.866 6.847 6.827 6.566 6.546 6.534 6.515 6.144 6.142 6.130 6.128 5.431 5.416 5.246 5.215 5.122 4.804 3.826 3.805 3.771 3.726 3.604 3.585 3.576 3.566 3.560 3.538 3.529 3.517 3.503 3.492 3.484 3.479 3.467 3.463 3.256 3.240 2.393 2.388
MeO
OMe
N
H
O
NH
Ts
19a/b (19a:19b = 2.5:1)
1H NMR (400 MHz, CDCl3)
2.07 0.84 0.42 1.00 0.77 2.31 0.45 1.03 1.00 0.38 0.94 0.35 0.95 0.91 0.33 0.38 0.91 0.33 1.21 1.33 3.28 3.50 4.57 1.18 0.41 4.60
8.5 8.0 7.5 7.0 6.5 6.0 5.5 5.0 4.5 4.0 3.5 3.0 2.5 2.0 1.5 1.0 0.5

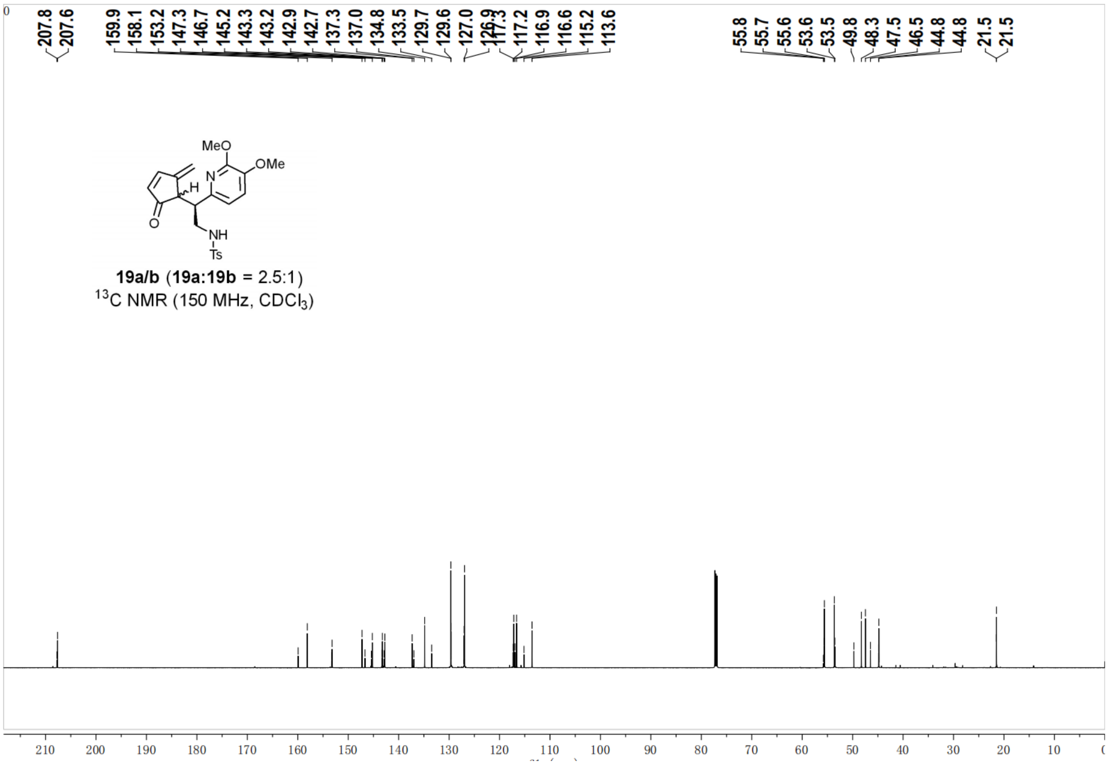
207.8 207.6 159.9 158.1 153.2 147.3 146.7 145.2 143.3 143.2 142.9 142.7 137.3 137.0 134.8 133.5 129.7 129.6 127.0 126.9 117.3 117.2 116.9 116.6 115.2 113.6 55.8 55.7 55.6 53.6 53.5 49.8 48.3 47.5 46.5 44.8 44.8 21.5 21.5
MeO
OMe
N
H
O
NH
Ts
19a/b (19a:19b = 2.5:1)
13C NMR (150 MHz, CDCl3)
210 200 190 180 170 160 150 140 130 120 110 100 90 80 70 60 50 40 30 20 10

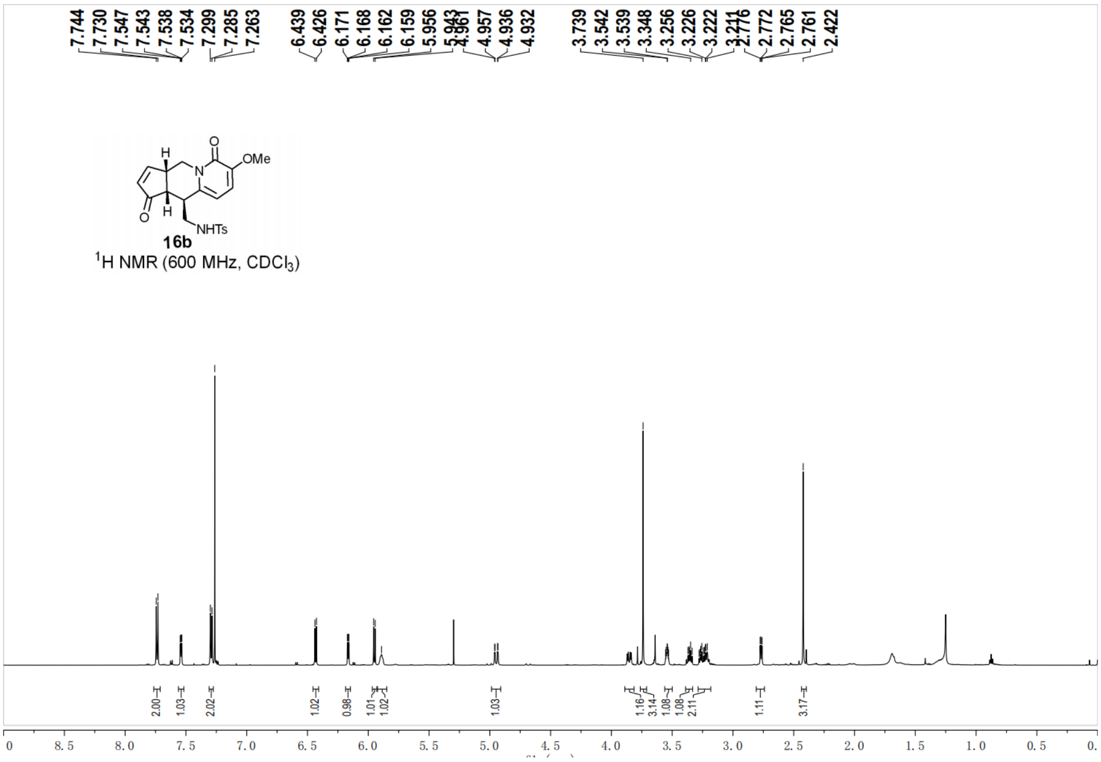
7.744 7.730 7.547 7.543 7.538 7.534 7.299 7.285 7.263 6.439 6.426 6.171 6.168 6.162 6.159 5.956 5.943 4.961 4.957 4.936 4.932 3.739 3.542 3.539 3.348 3.256 3.226 3.222 3.211 2.776 2.772 2.765 2.761 2.422
OMe
NHTs
16b
^{1}H NMR (600 MHz, CDCl$_3$)
2.00 1.03 2.02 1.02 0.98 1.01 1.02 1.03 1.16 3.14 1.08 1.08 2.11 1.11 3.17
8.5 8.0 7.5 7.0 6.5 6.0 5.5 5.0 4.5 4.0 3.5 3.0 2.5 2.0 1.5 1.0 0.5

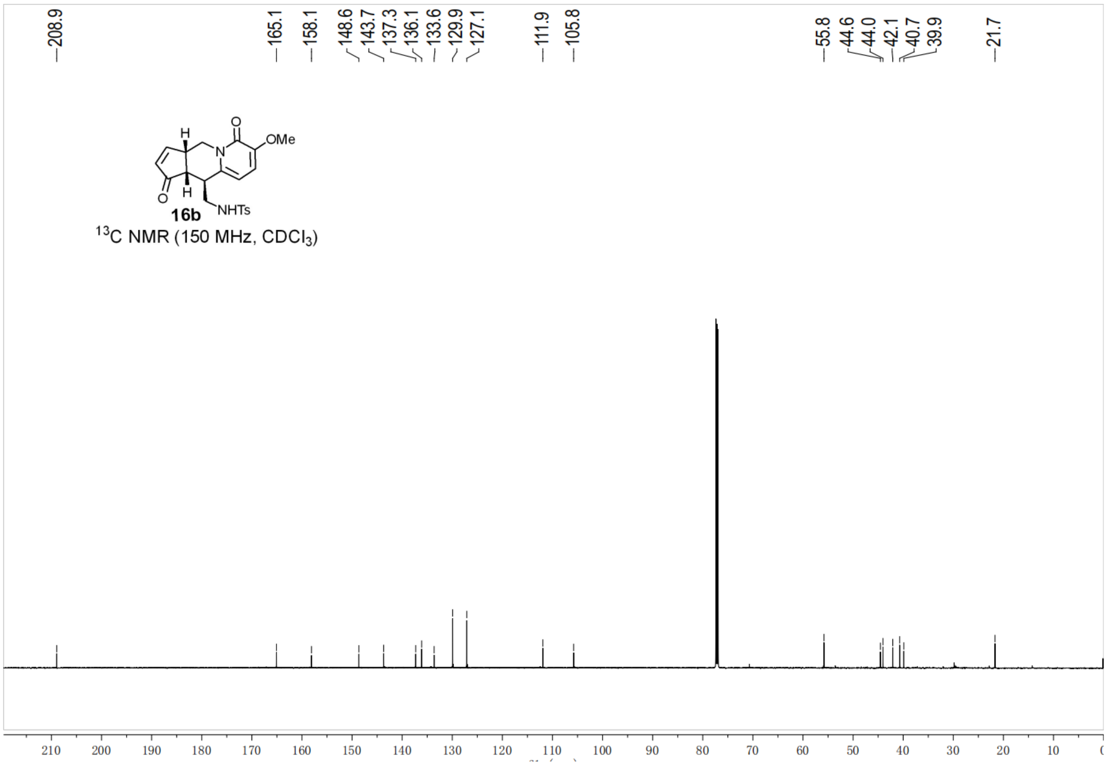
208.9 165.1 158.1 148.6 143.7 137.3 136.1 133.6 129.9 127.1 111.9 105.8 55.8 44.6 44.0 42.1 40.7 39.9 21.7
OMe
NHTs
16b
^{13}C NMR (150 MHz, CDCl$_3$)
210 200 190 180 170 160 150 140 130 120 110 100 90 80 70 60 50 40 30 20 10

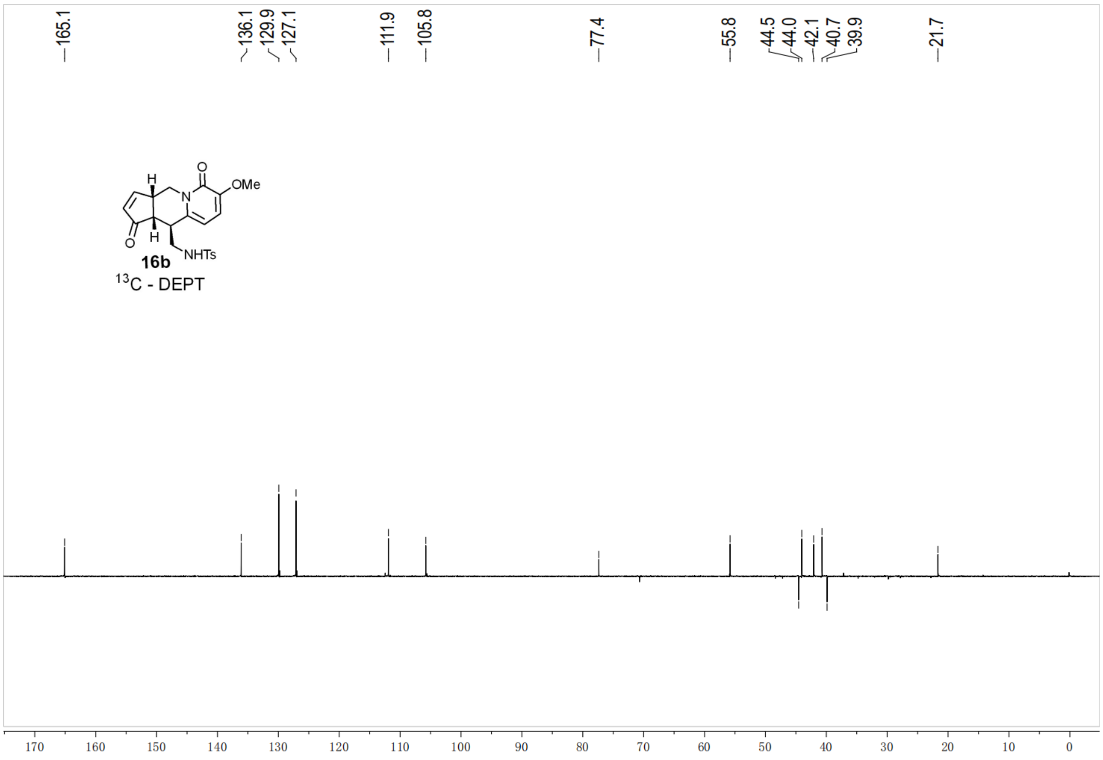
165.1
136.1
129.9
127.1
111.9
105.8
77.4
55.8
44.5
44.0
42.1
40.7
39.9
21.7
OMe
NHTs
16b
^{13}C - DEPT
170 160 150 140 130 120 110 100 90 80 70 60 50 40 30 20 10 0

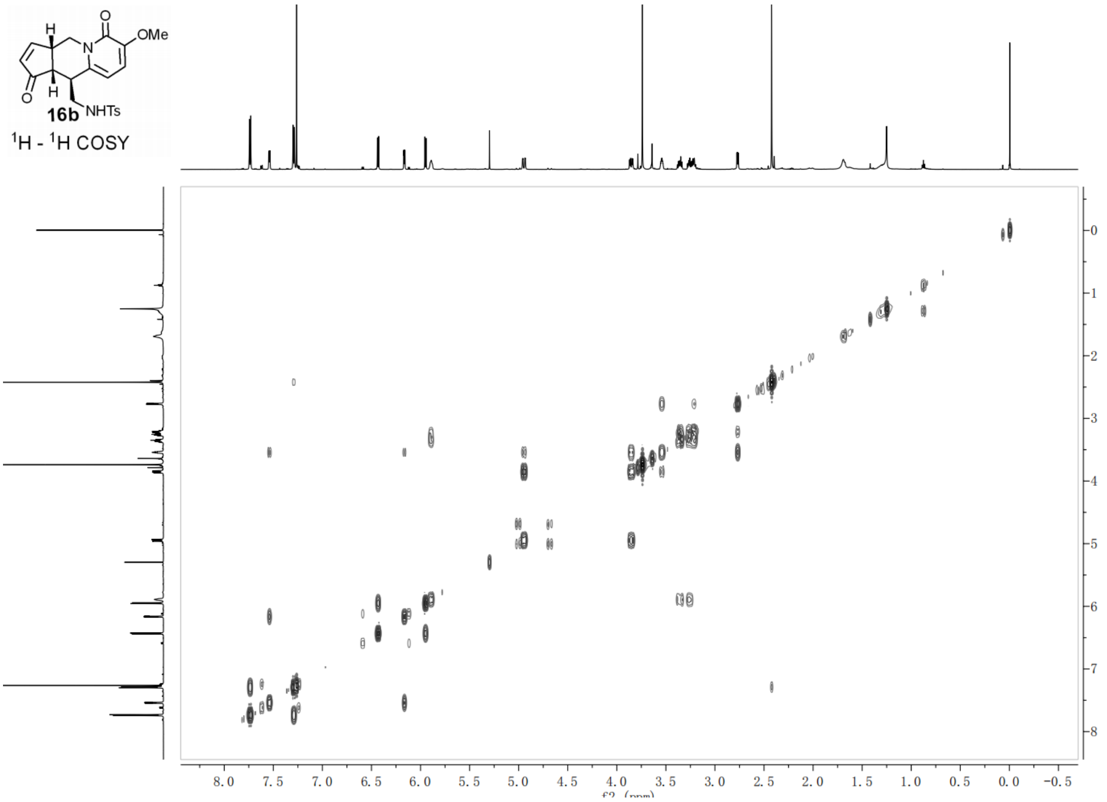
OMe
NHTs
16b
^{1}H - ^{1}H COSY
8.0 7.5 7.0 6.5 6.0 5.5 5.0 4.5 4.0 3.5 3.0 2.5 2.0 1.5 1.0 0.5 0.0 -0.5

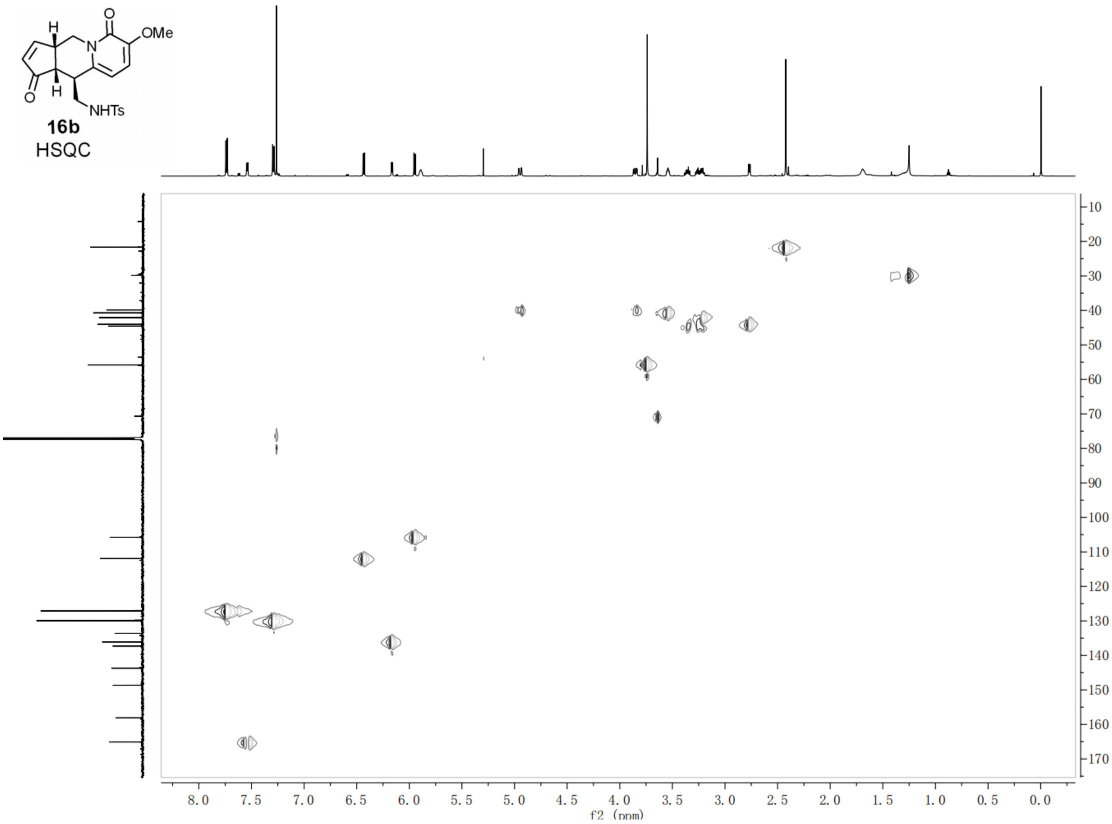
H
O
N
OMe
H
O
NHTs
16b
HSQC
f2 (ppm)

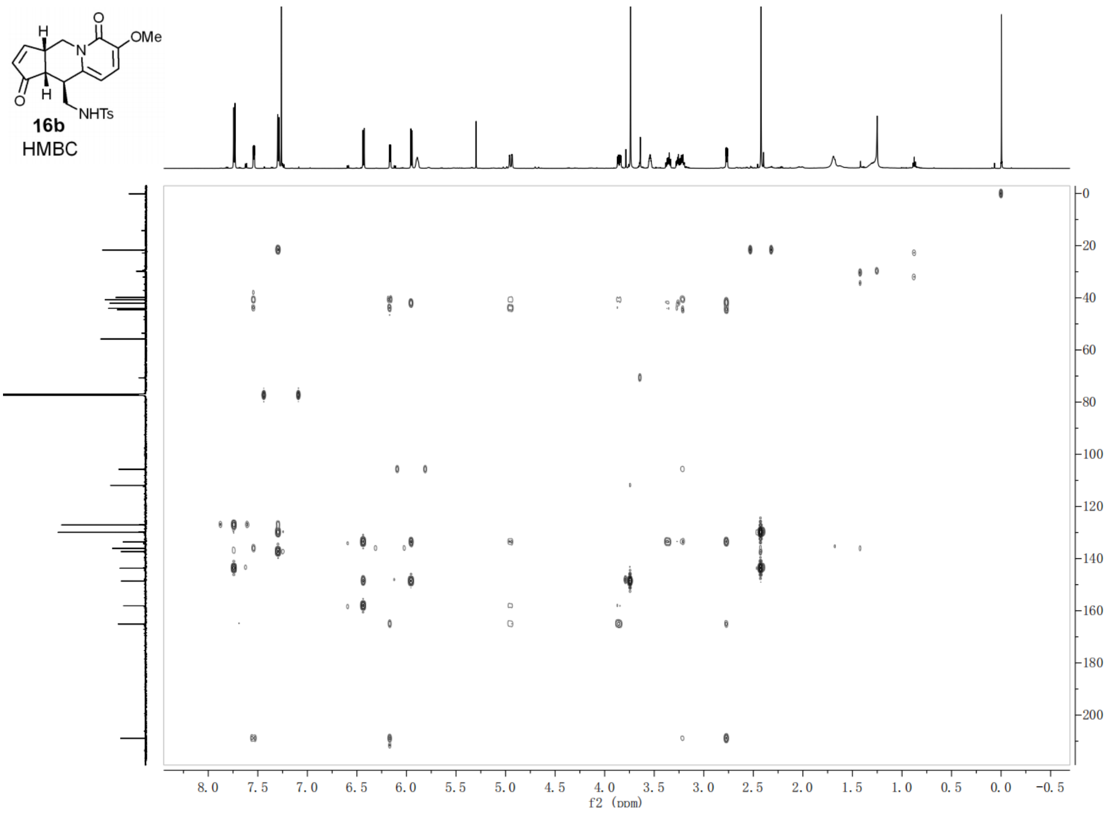
H
O
N
OMe
H
O
NHTs
16b
HMBC
f2 (ppm)

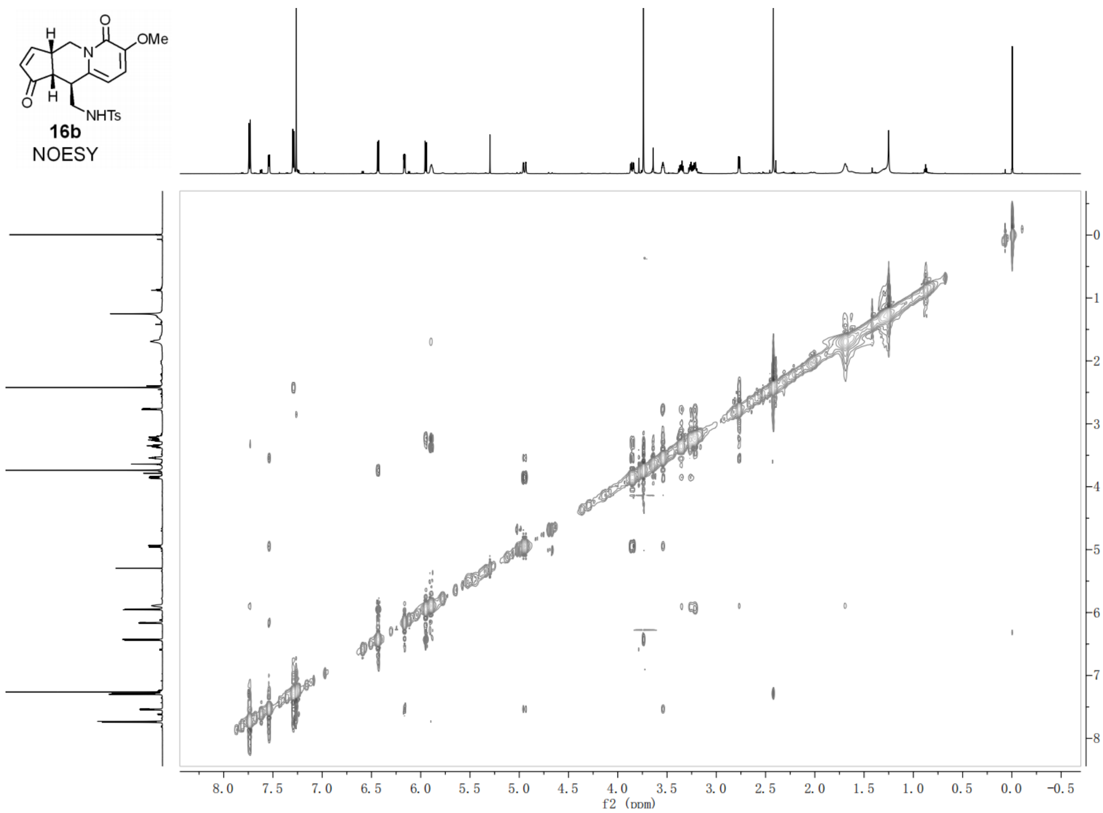
OMe
NHTs
16b
NOESY
f2 (ppm)

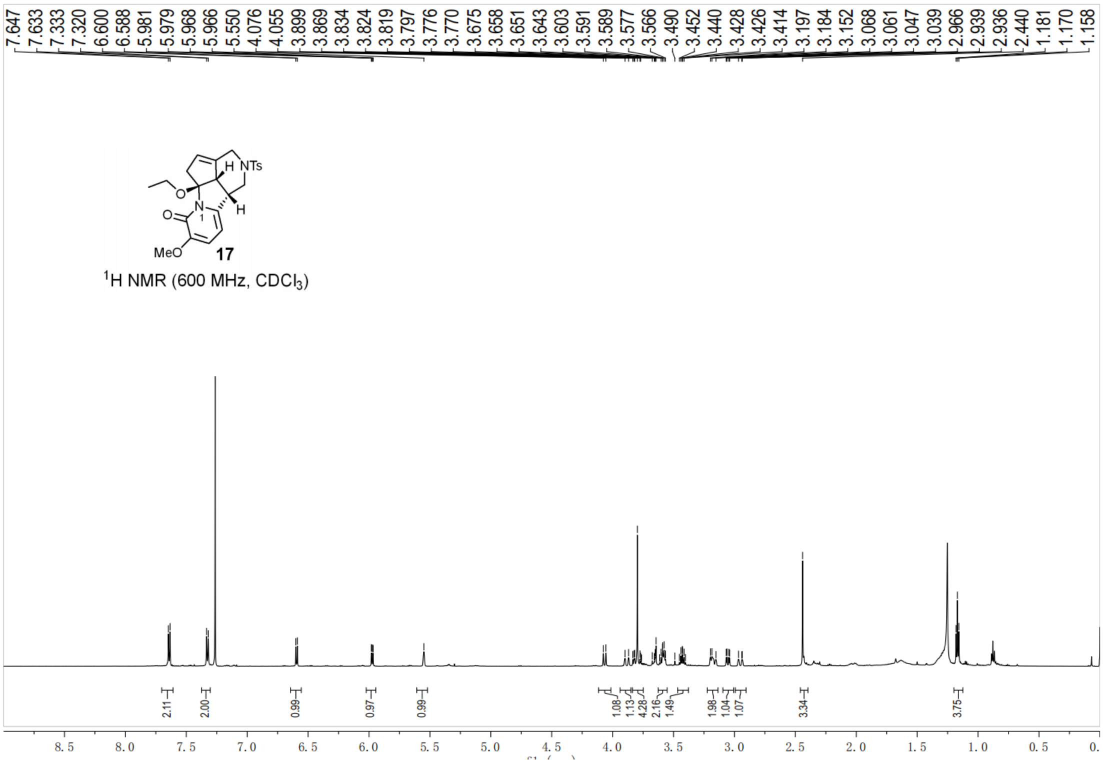
7.647 7.633 7.333 7.320 6.600 6.588 5.981 5.979 5.968 5.966 5.550 4.076 4.055 3.899 3.869 3.834 3.824 3.819 3.797 3.776 3.770 3.675 3.658 3.651 3.643 3.603 3.591 3.589 3.577 3.566 3.490 3.452 3.440 3.428 3.426 3.414 3.197 3.184 3.152 3.068 3.061 3.047 3.039 2.966 2.939 2.936 2.440 1.181 1.170 1.158
H
NTs
O
N
H
O
MeO
17
1H NMR (600 MHz, CDCl3)
2.11 2.00 0.99 0.97 0.99 1.08 1.13 4.28 2.16 1.49 1.98 1.04 1.07 3.34 3.75
8.5 8.0 7.5 7.0 6.5 6.0 5.5 5.0 4.5 4.0 3.5 3.0 2.5 2.0 1.5 1.0 0.5

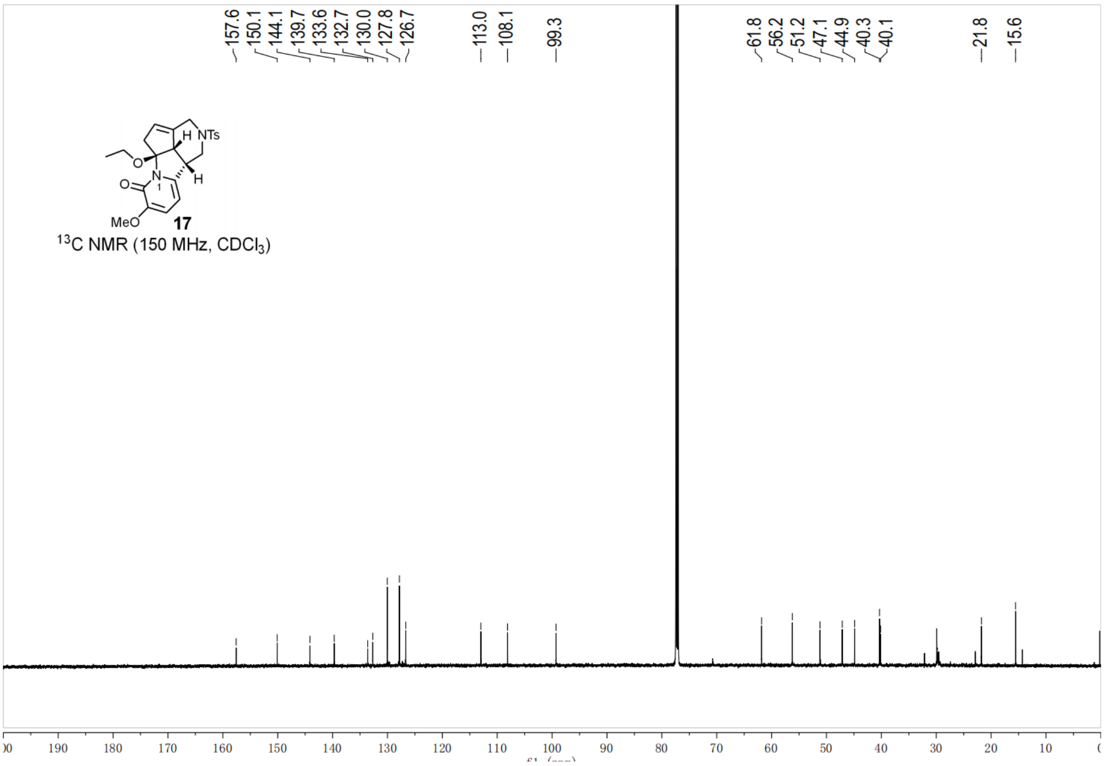
157.6 150.1 144.1 139.7 133.6 132.7 130.0 127.8 126.7 113.0 108.1 99.3 61.8 56.2 51.2 47.1 44.9 40.3 40.1 21.8 15.6
H
NTs
O
N
H
O
MeO
17
13C NMR (150 MHz, CDCl3)
190 180 170 160 150 140 130 120 110 100 90 80 70 60 50 40 30 20 10

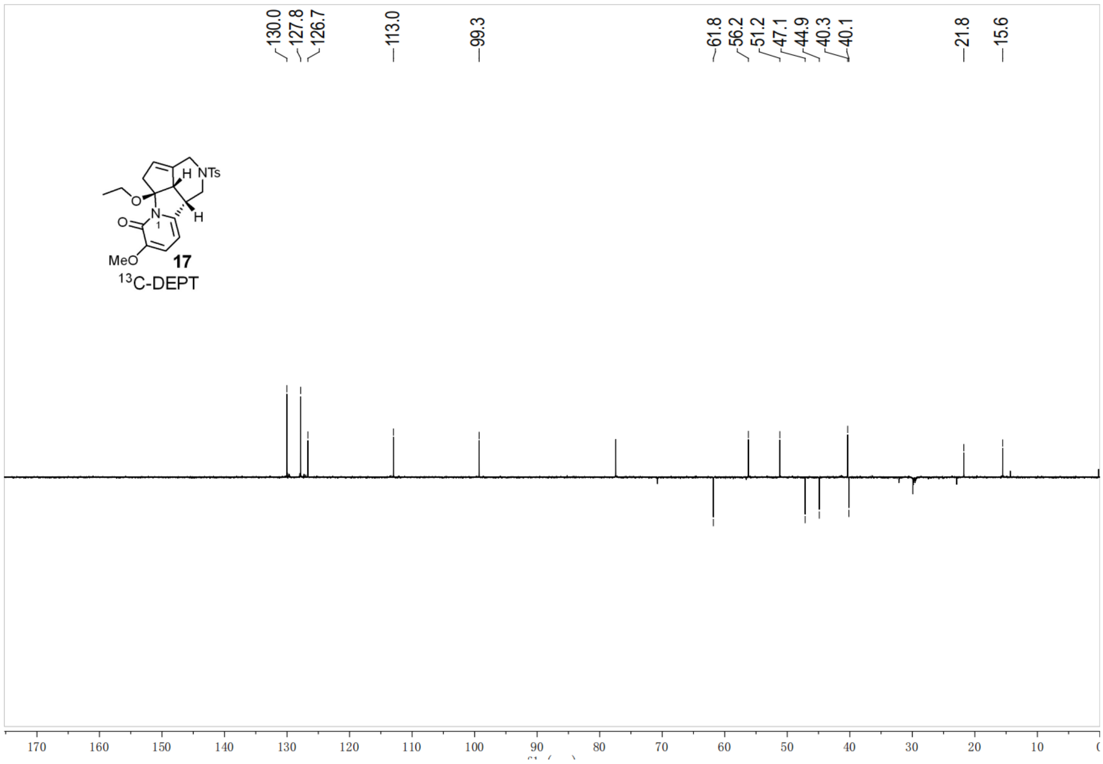
130.0
127.8
126.7
113.0
99.3
61.8
56.2
51.2
47.1
44.9
40.3
40.1
21.8
15.6
NTs
H
H
O
O
N
MeO
17
^{13}C-DEPT

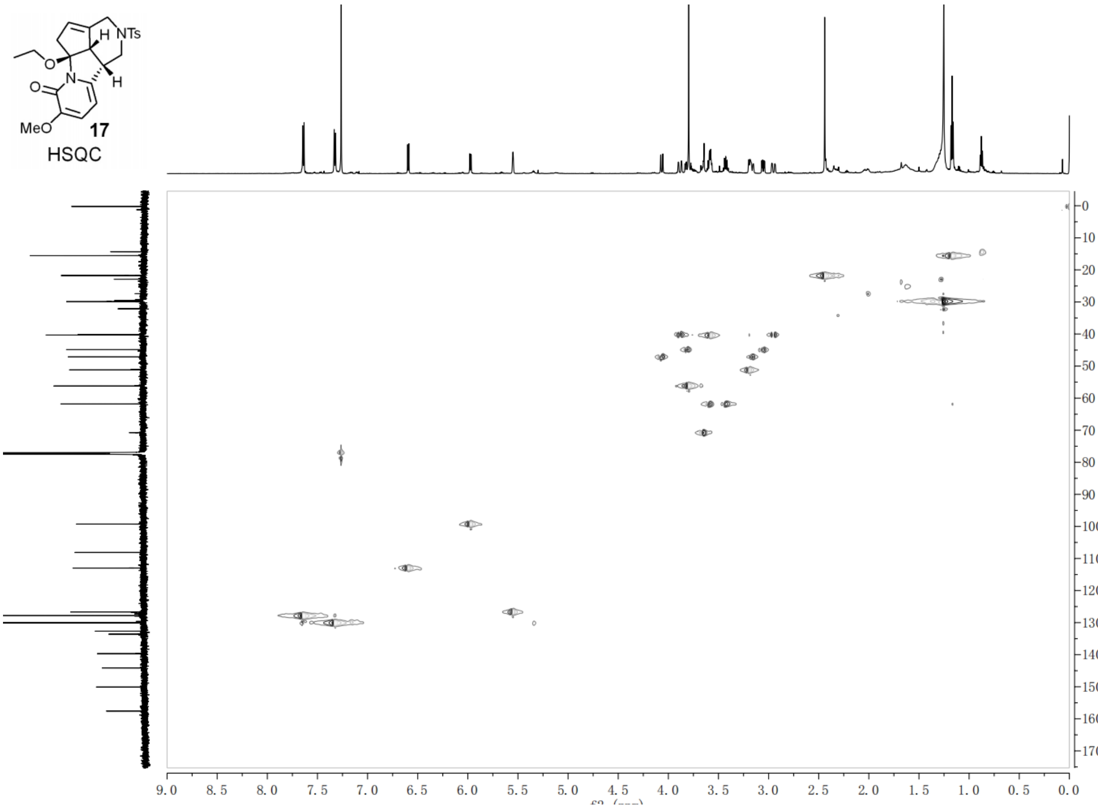
NTs
H
H
O
O
N
MeO
17
HSQC

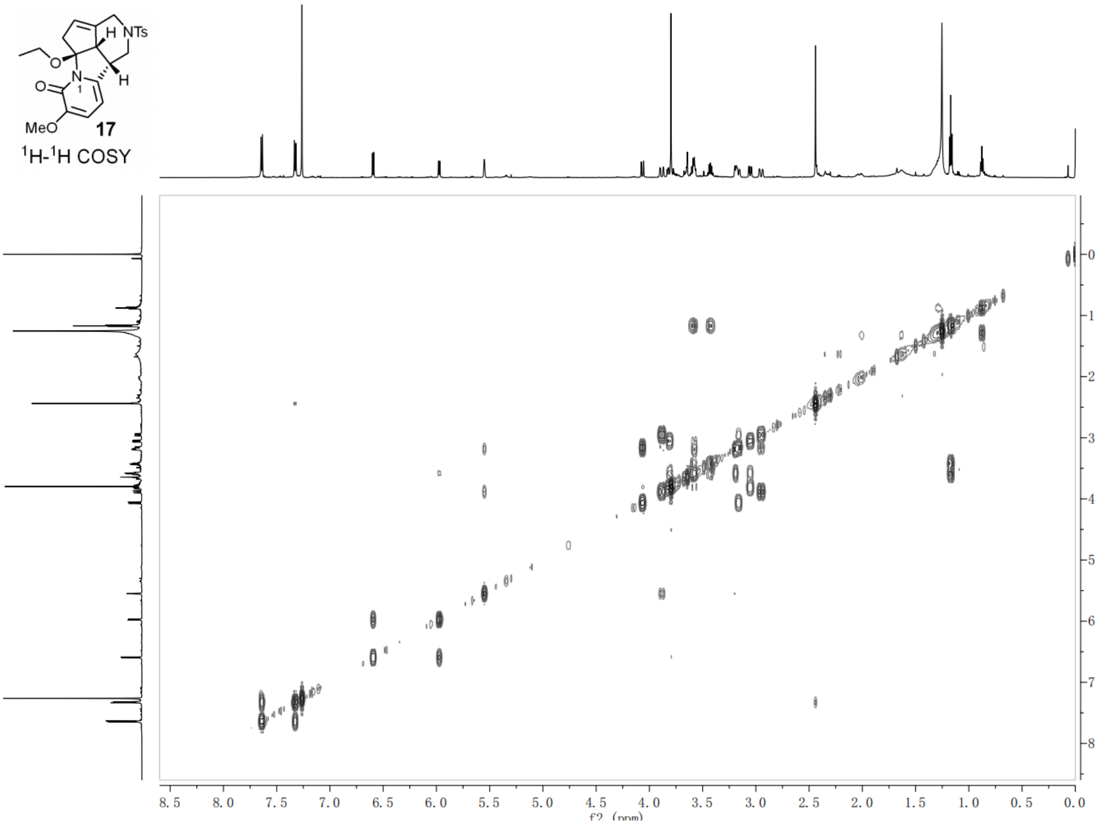
NTs
H
O
N
O
H
MeO
17
^{1}H-^{1}H COSY
f2 (ppm)

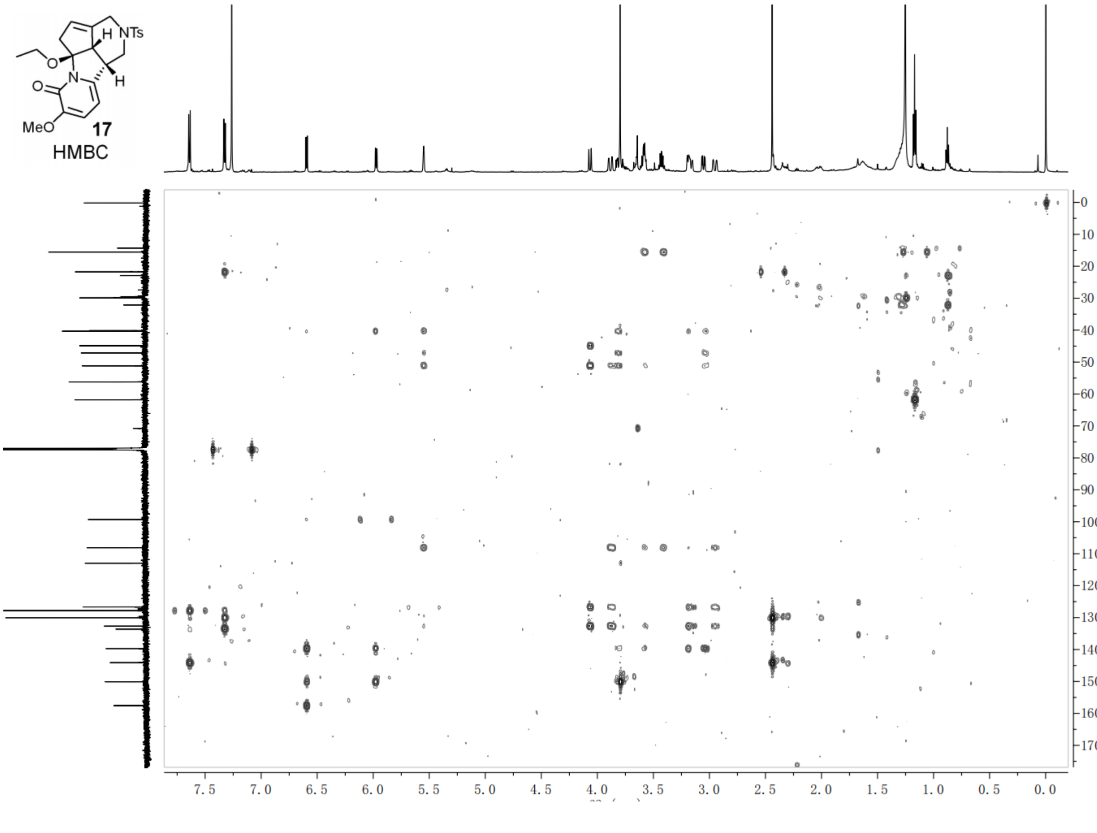
NTs
H
O
N
O
H
MeO
17
HMBC

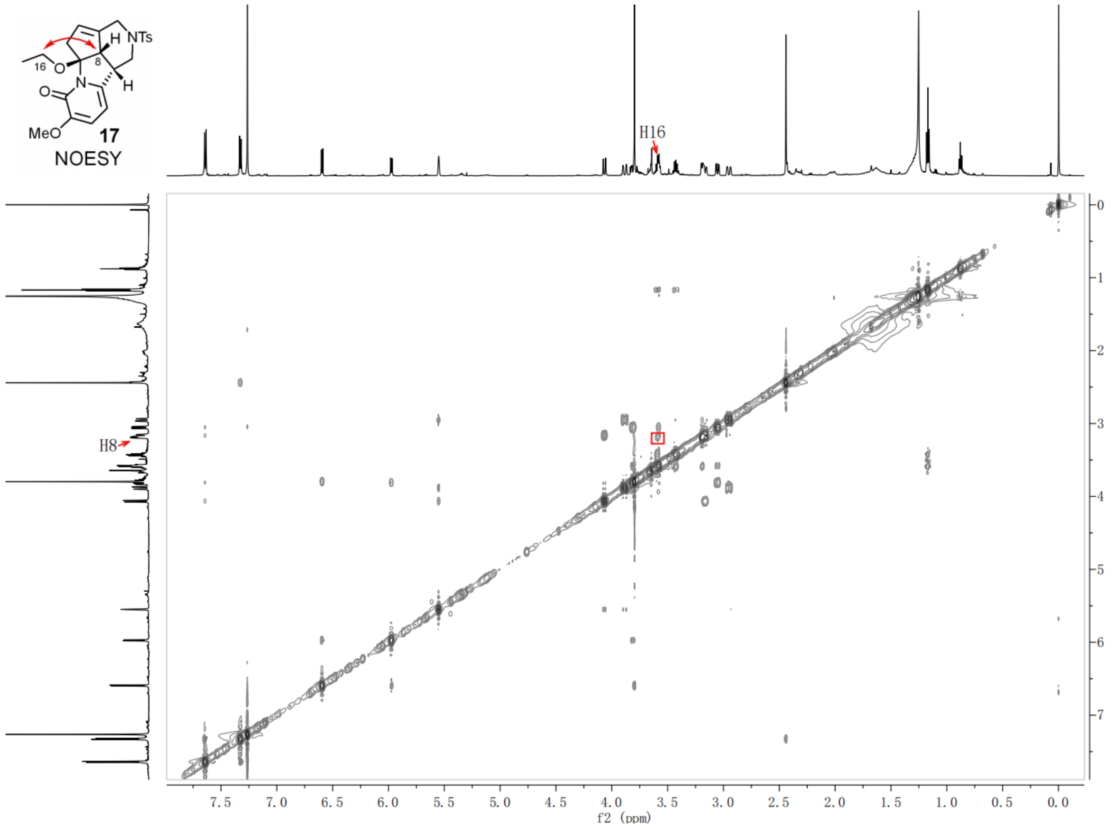
NTs
H
16
O
8
H
O
N
MeO
17
NOESY
H16
H8
f2 (ppm)

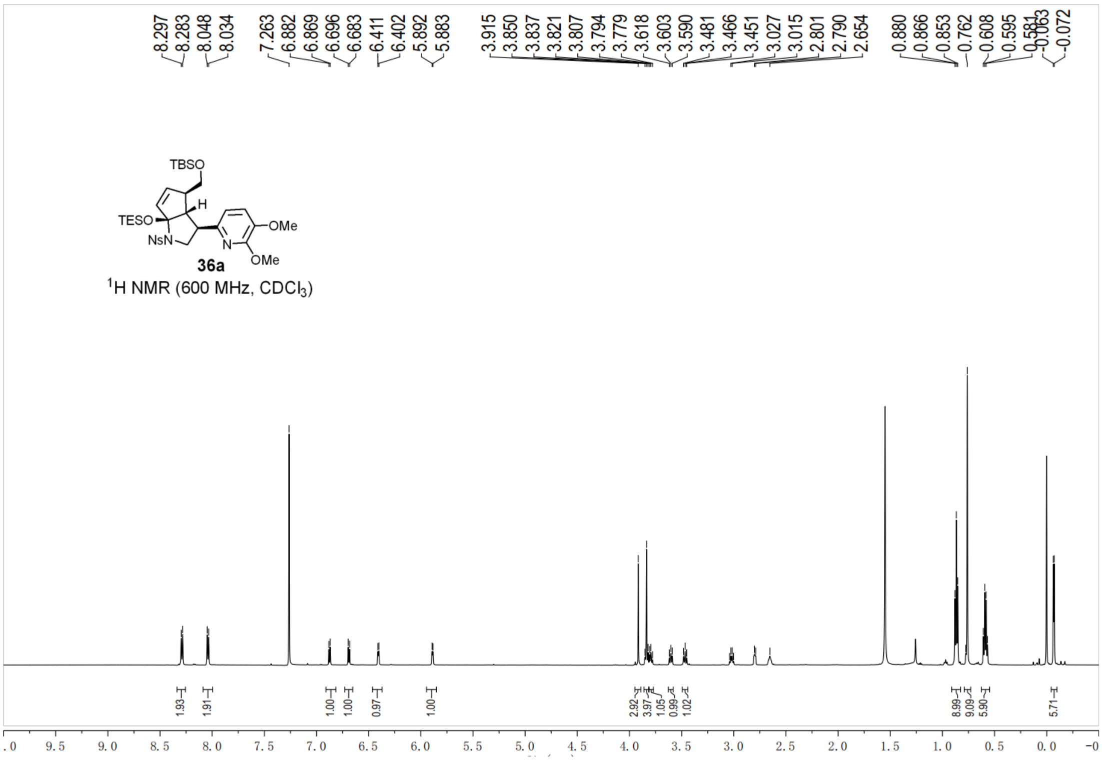

TBSO
TESO
NsN
H
OMe
OMe
36a
¹H NMR (600 MHz, CDCl₃)

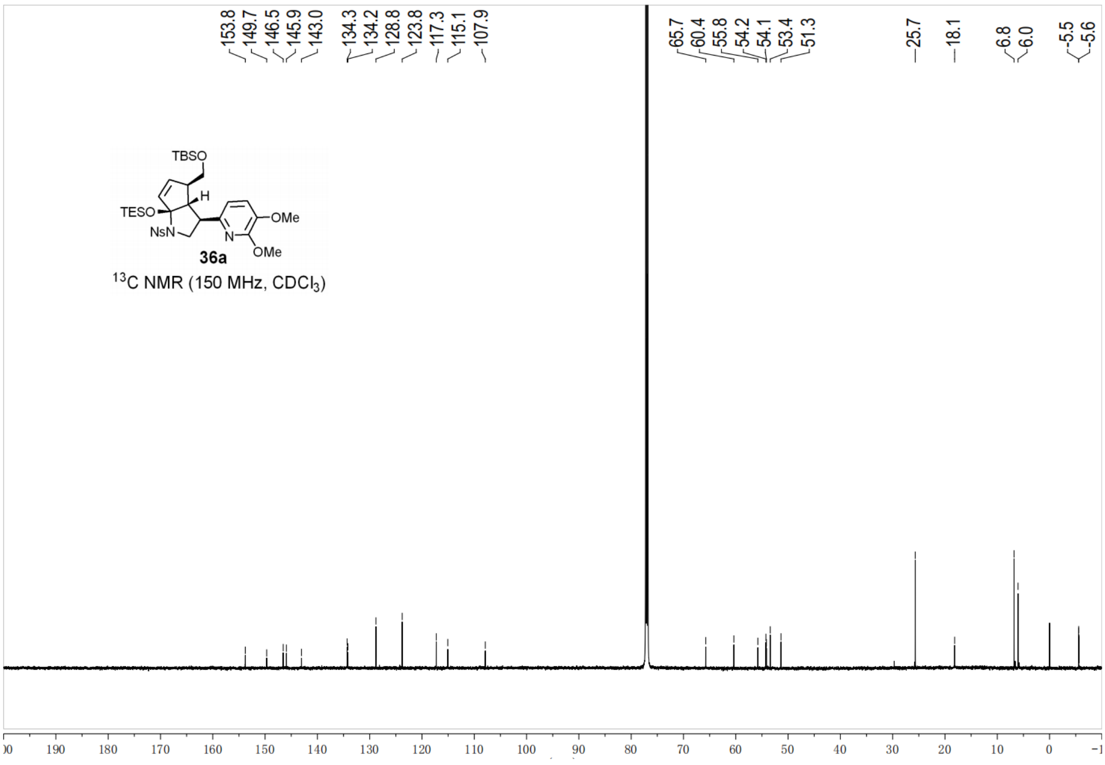

TBSO
TESO
NsN
H
OMe
OMe
36a
¹³C NMR (150 MHz, CDCl₃)

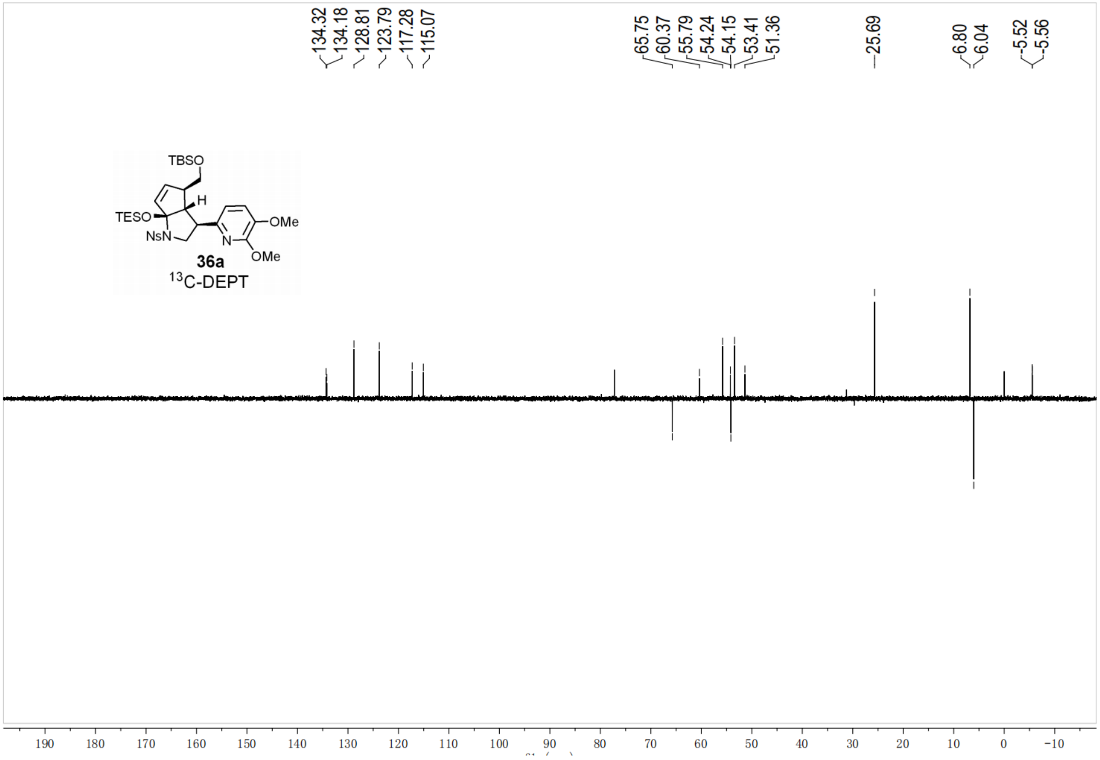
134.32
134.18
128.81
123.79
117.28
115.07
65.75
60.37
55.79
54.24
54.15
53.41
51.36
25.69
6.80
6.04
-5.52
-5.56
TBSO
H
TESO
NsN
N
OMe
OMe
36a
13C-DEPT
190 180 170 160 150 140 130 120 110 100 90 80 70 60 50 40 30 20 10 0 -10

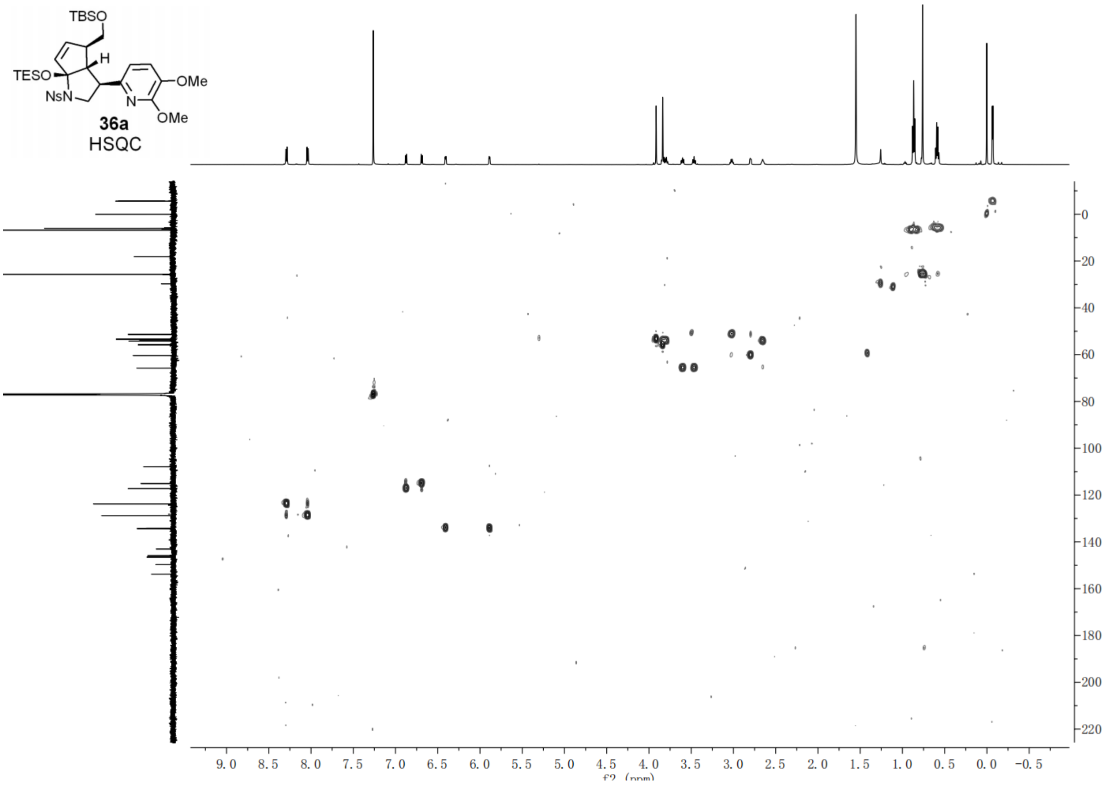
TBSO
H
TESO
NsN
N
OMe
OMe
36a
HSQC
9.0 8.5 8.0 7.5 7.0 6.5 6.0 5.5 5.0 4.5 4.0 3.5 3.0 2.5 2.0 1.5 1.0 0.5 0.0 -0.5
0 20 40 60 80 100 120 140 160 180 200 220

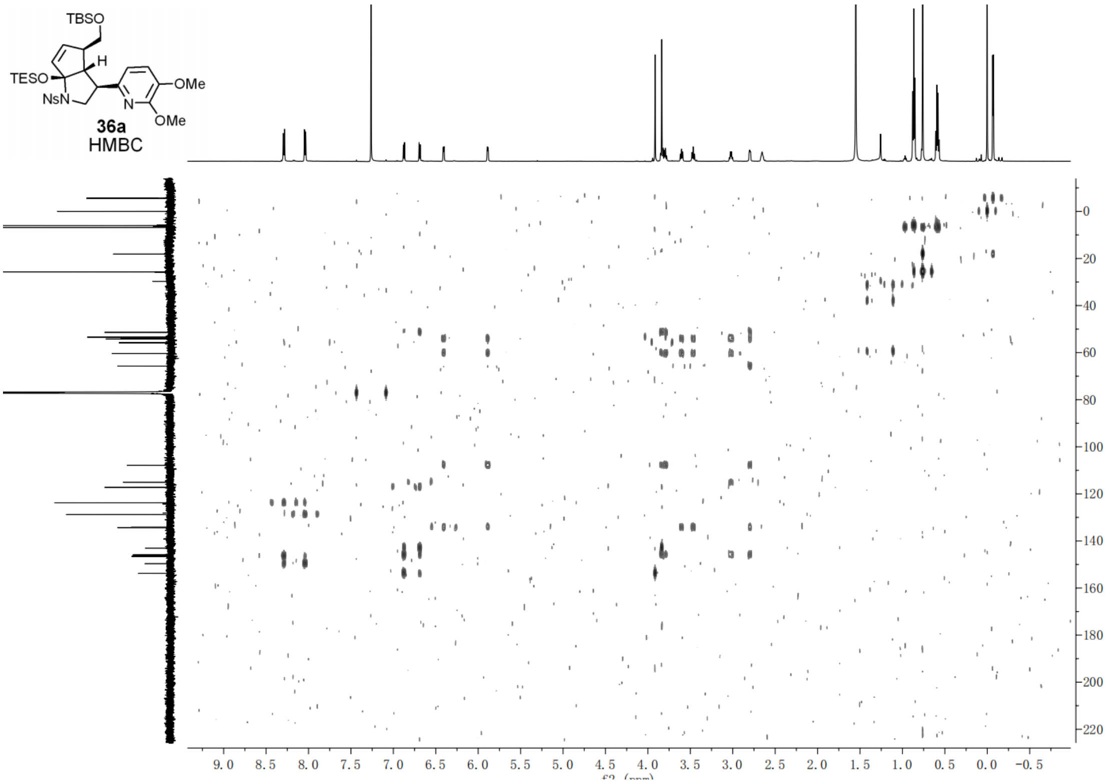
TBSO
H
TESO
OMe
NsN
N
OMe
36a
HMBC
f2 (ppm)

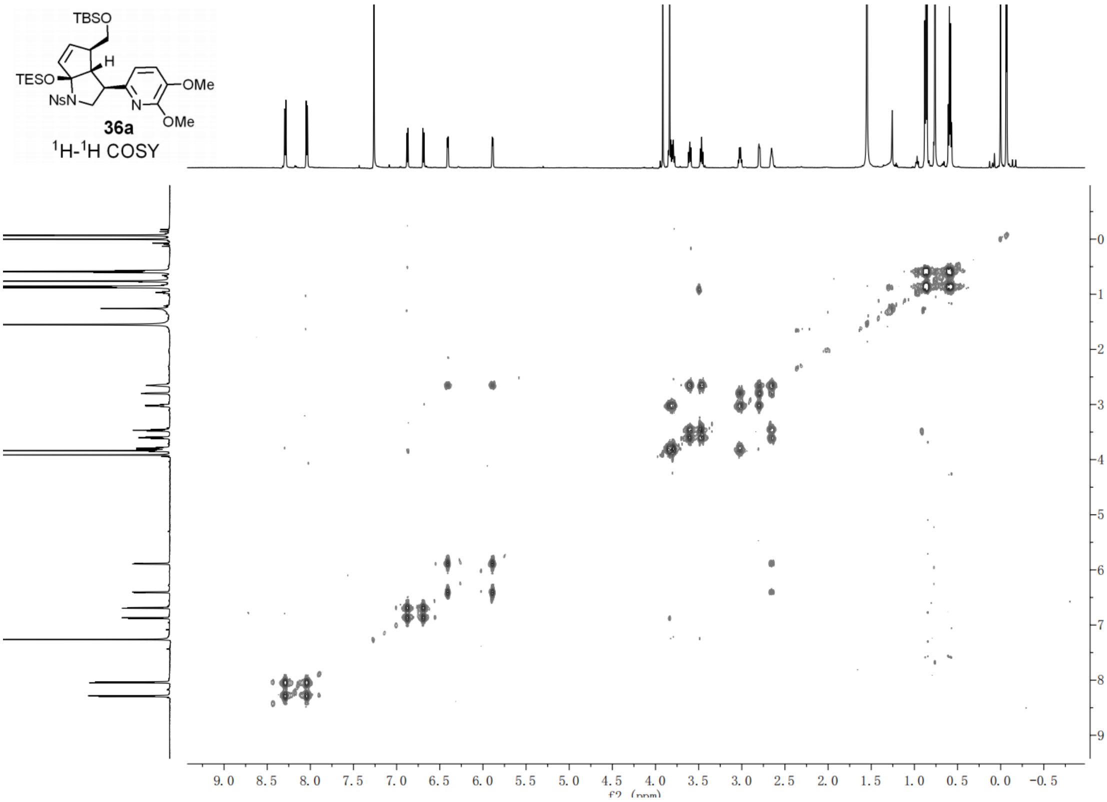
TBSO
H
TESO
OMe
NsN
N
OMe
36a
1H-1H COSY
f2 (ppm)

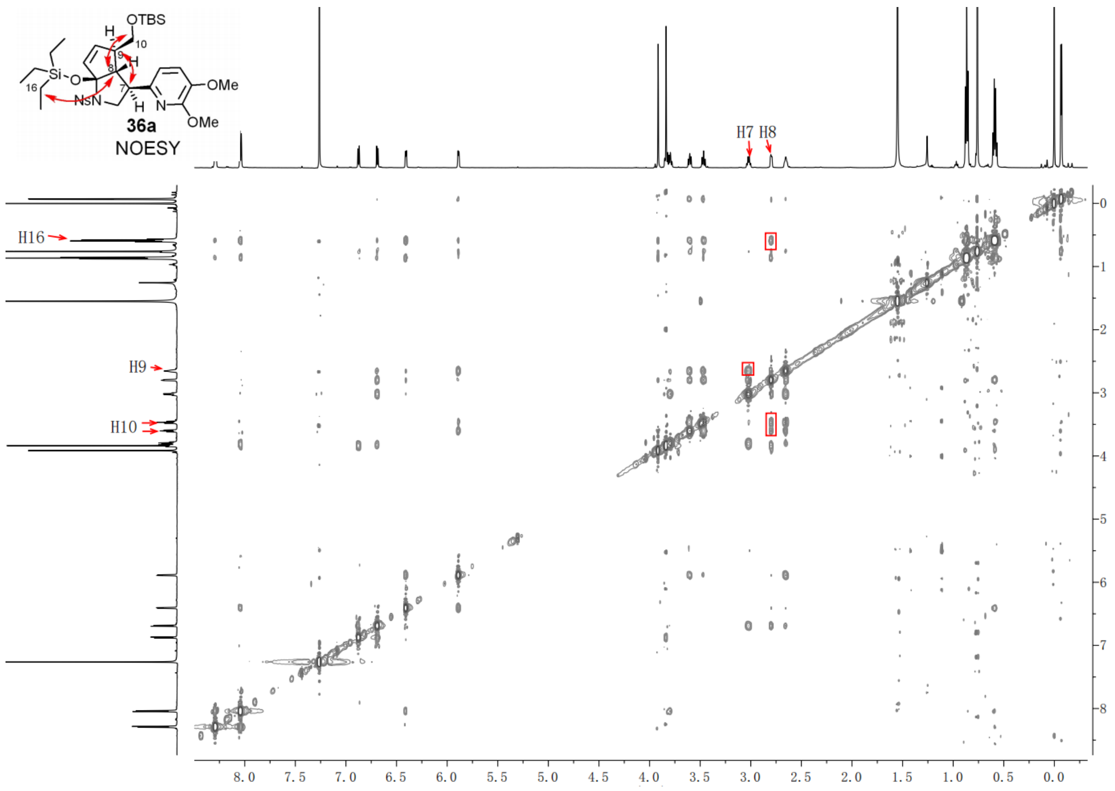

OTBS
10
Si
16
O
NsN
H
N
OMe
OMe
36a
NOESY
H7 H8
H16
H9
H10

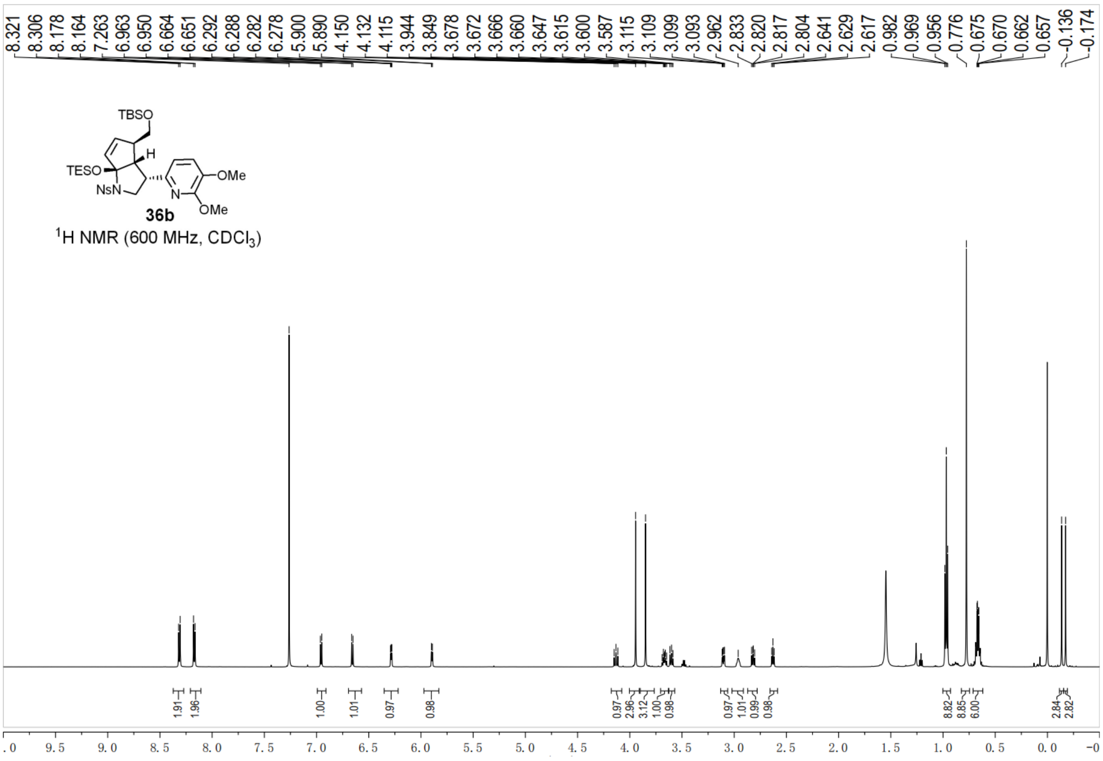
8.321
8.306
8.178
8.164
7.263
6.963
6.950
6.664
6.651
6.292
6.288
6.282
6.278
5.900
5.890
4.150
4.132
4.115
3.944
3.849
3.678
3.672
3.666
3.660
3.647
3.615
3.600
3.587
3.115
3.109
3.099
3.093
2.962
2.833
2.820
2.817
2.804
2.641
2.629
2.617
0.982
0.969
0.956
0.776
0.675
0.670
0.662
0.657
-0.136
-0.174
TBSO
H
TESO
NsN
N
OMe
OMe
36b
^{1}H NMR (600 MHz, $CDCl_3$)
1.91
1.96
1.00
1.01
0.97
0.98
0.97
2.96
3.12
1.00
0.98
0.97
1.01
0.99
0.98
8.82
8.85
6.00
2.84
2.82

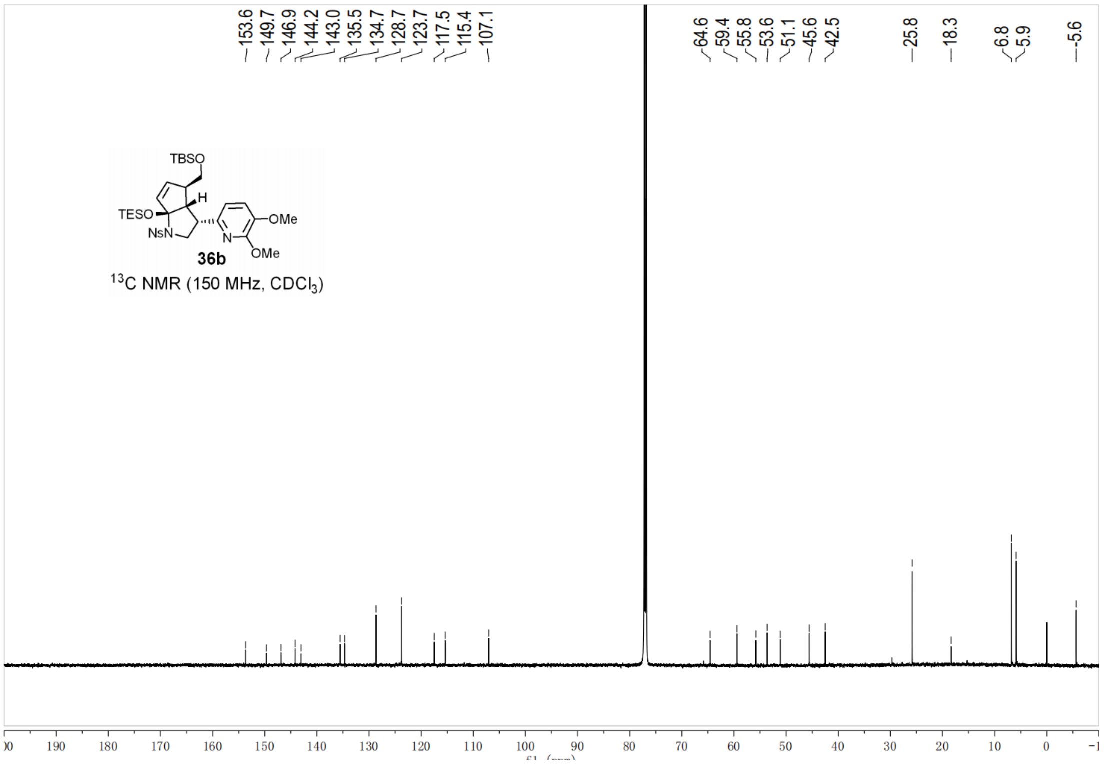
153.6
149.7
146.9
144.2
143.0
135.5
134.7
128.7
123.7
117.5
115.4
107.1
64.6
59.4
55.8
53.6
51.1
45.6
42.5
25.8
18.3
6.8
5.9
-5.6
TBSO
H
TESO
NsN
N
OMe
OMe
36b
^{13}C NMR (150 MHz, $CDCl_3$)

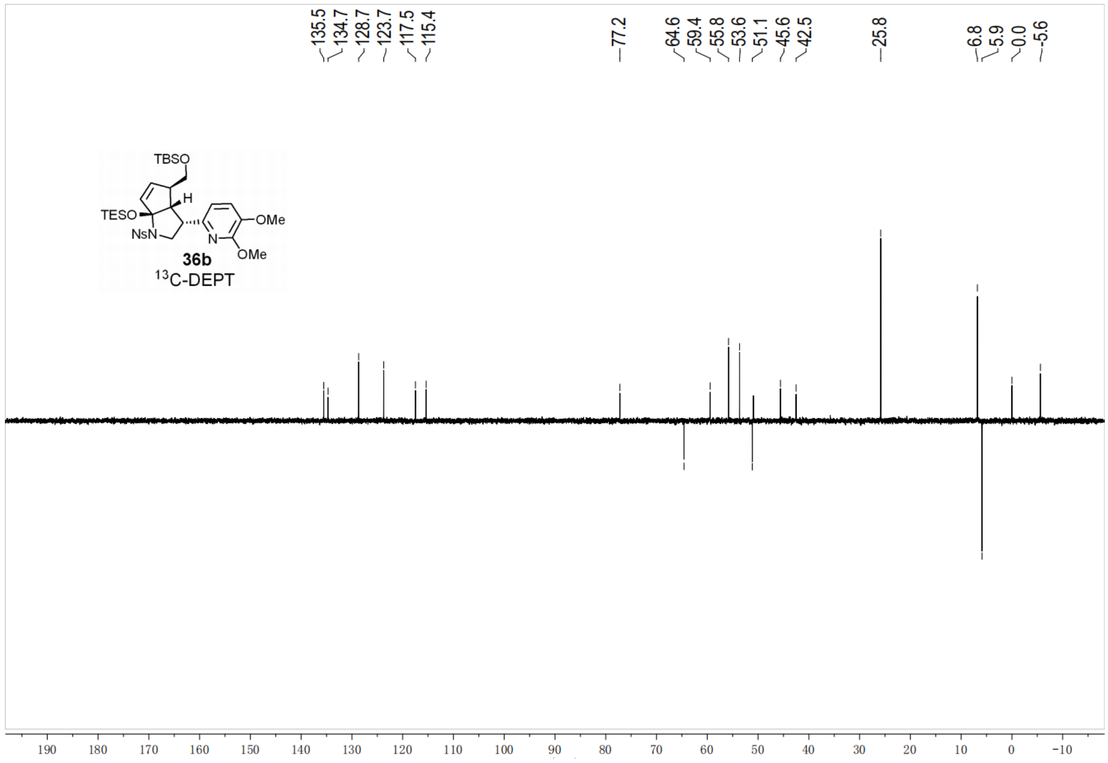
135.5
134.7
128.7
123.7
117.5
115.4
77.2
64.6
59.4
55.8
53.6
51.1
45.6
42.5
25.8
6.8
5.9
0.0
-5.6
TBSO
H
TESO
OMe
NsN
N
OMe
36b
^{13}C-DEPT

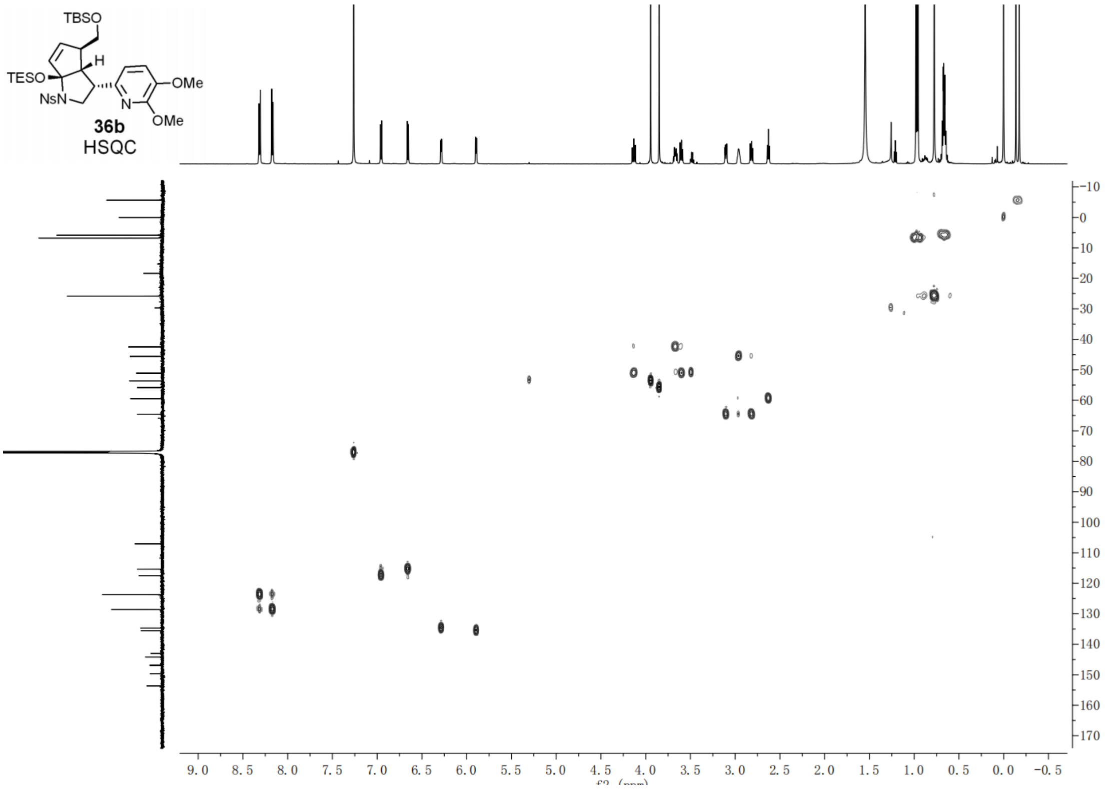
TBSO
H
TESO
OMe
NsN
N
OMe
36b
HSQC

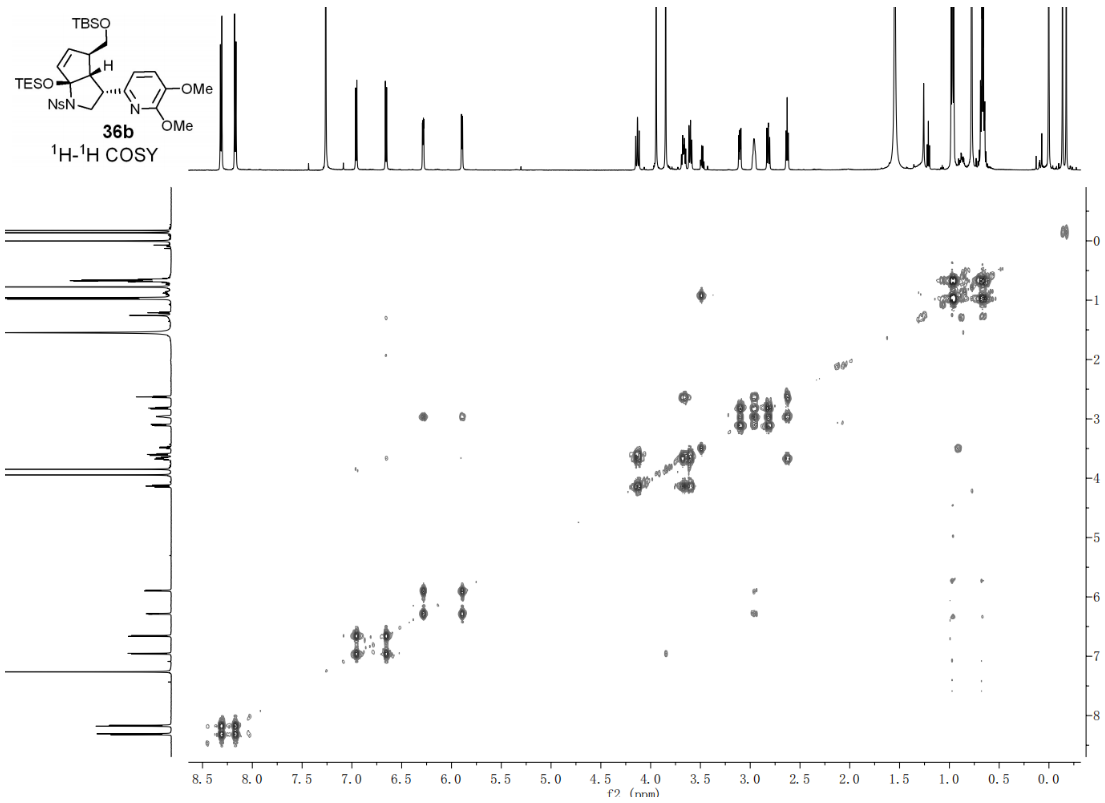
TBSO
TESO
NsN
OMe
OMe
N
H
36b
¹H-¹H COSY
f2 (ppm)

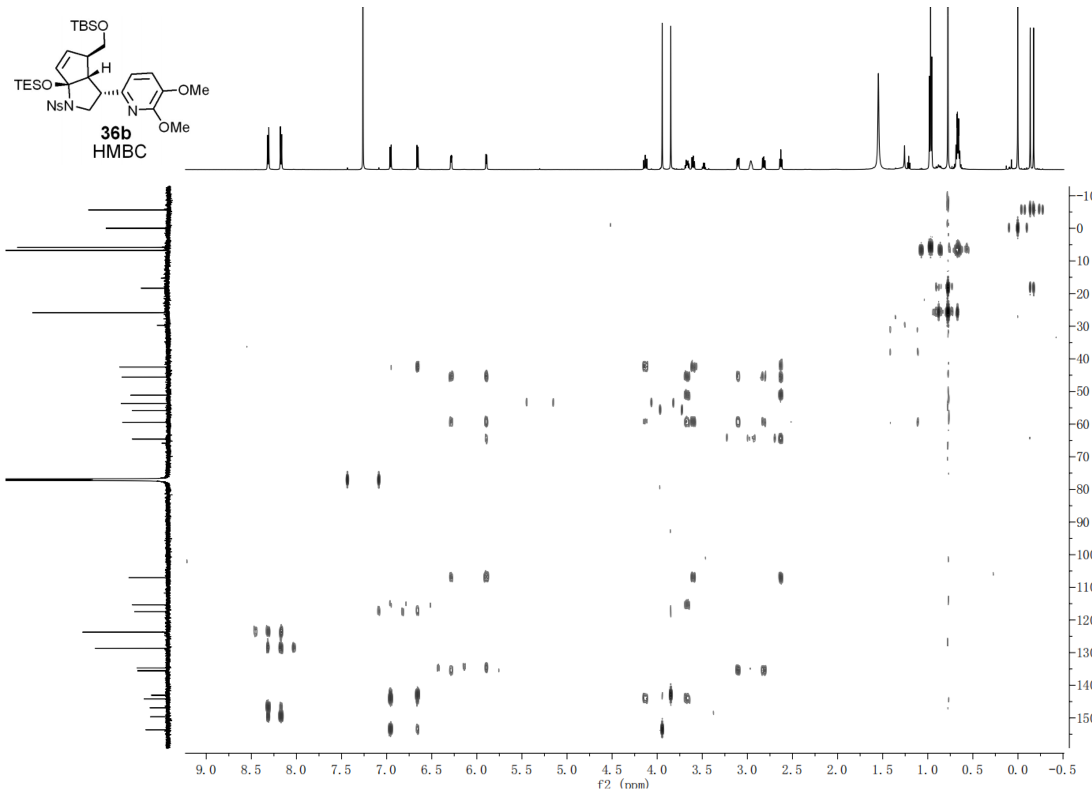
TBSO
TESO
NsN
OMe
OMe
N
H
36b
HMBC
f2 (ppm)

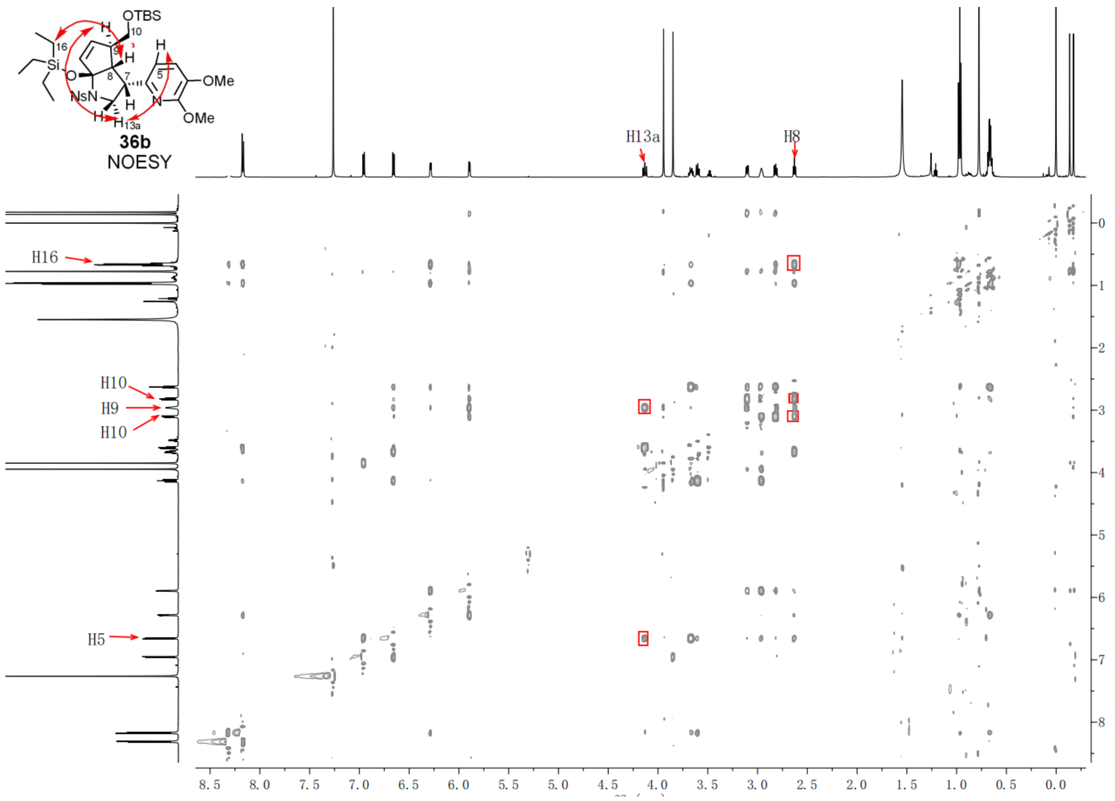
OTBS
OMe
OMe
NsN
H13a
36b
NOESY
H13a
H8
H16
H10
H9
H10
H5

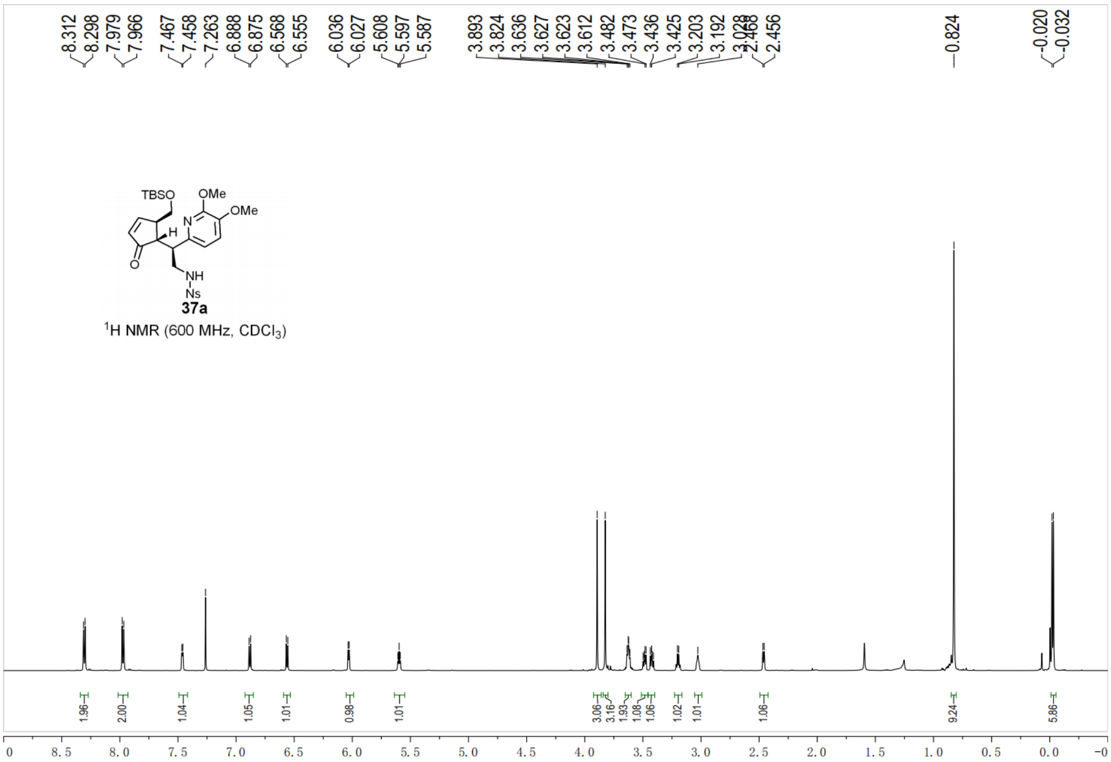
8.312
8.298
7.979
7.966
7.467
7.458
7.263
6.888
6.875
6.568
6.555
6.036
6.027
5.608
5.597
5.587
3.893
3.824
3.636
3.627
3.623
3.612
3.482
3.473
3.436
3.425
3.203
3.192
2.468
2.456
0.824
-0.020
-0.032
TBSO
OMe
OMe
N
H
O
NH
Ns
37a
1H NMR (600 MHz, CDCl3)

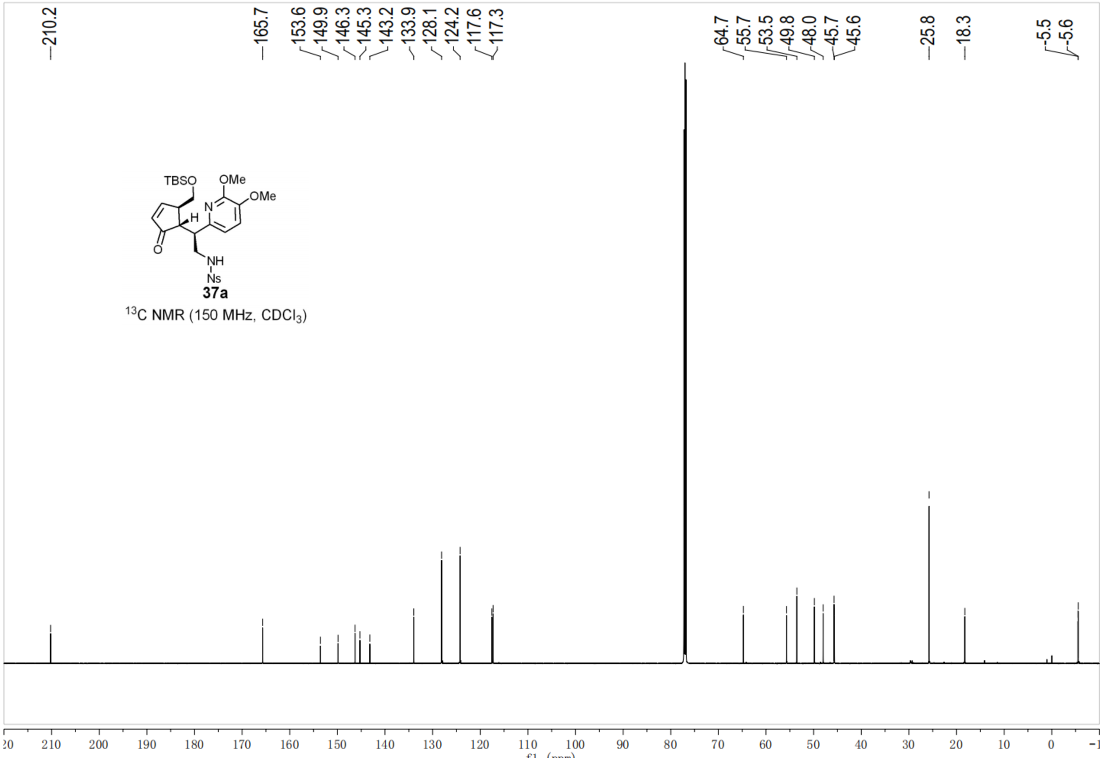
210.2
165.7
153.6
149.9
146.3
145.3
143.2
133.9
128.1
124.2
117.6
117.3
64.7
55.7
53.5
49.8
48.0
45.7
45.6
25.8
18.3
-5.5
-5.6
TBSO
OMe
OMe
N
H
O
NH
Ns
37a
13C NMR (150 MHz, CDCl3)

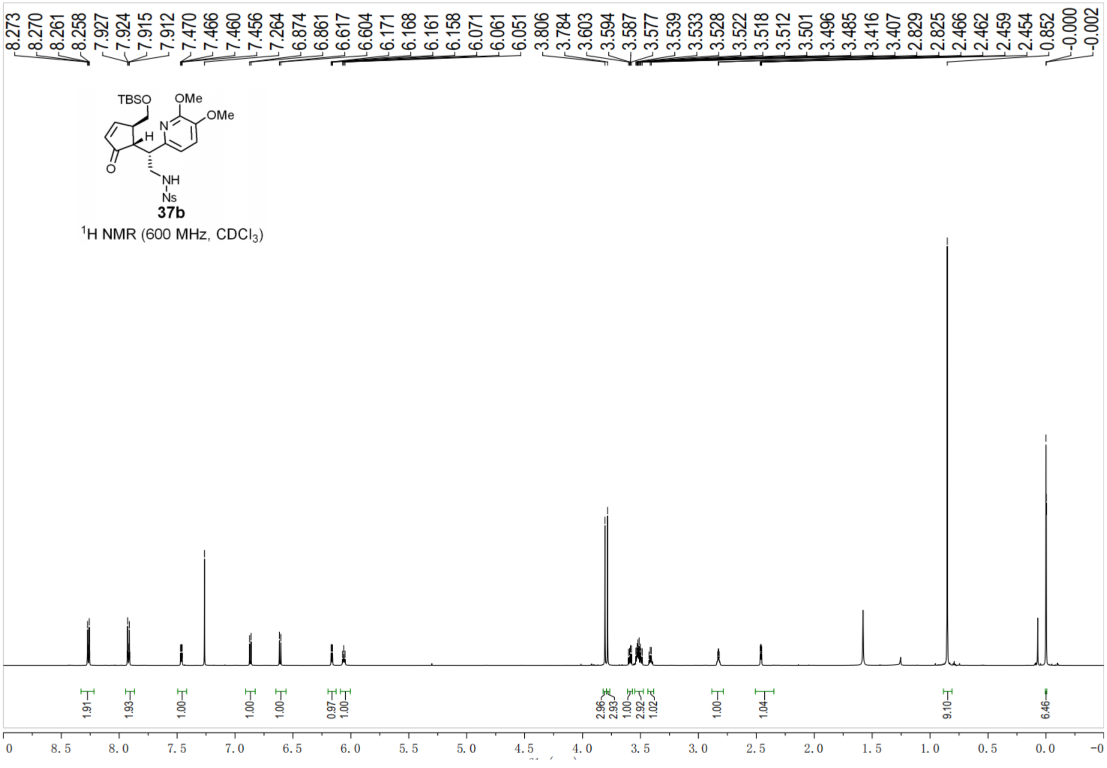
8.273
8.270
8.261
8.258
7.927
7.924
7.915
7.912
7.470
7.466
7.460
7.456
7.264
6.874
6.861
6.617
6.604
6.171
6.168
6.161
6.158
6.071
6.061
6.051
3.806
3.784
3.603
3.594
3.587
3.577
3.539
3.533
3.528
3.522
3.518
3.512
3.501
3.496
3.485
3.416
3.407
2.829
2.825
2.466
2.462
2.459
2.454
0.852
-0.000
-0.002
TBSO
OMe
OMe
N
H
O
NH
Ns
37b
1H NMR (600 MHz, CDCl3)

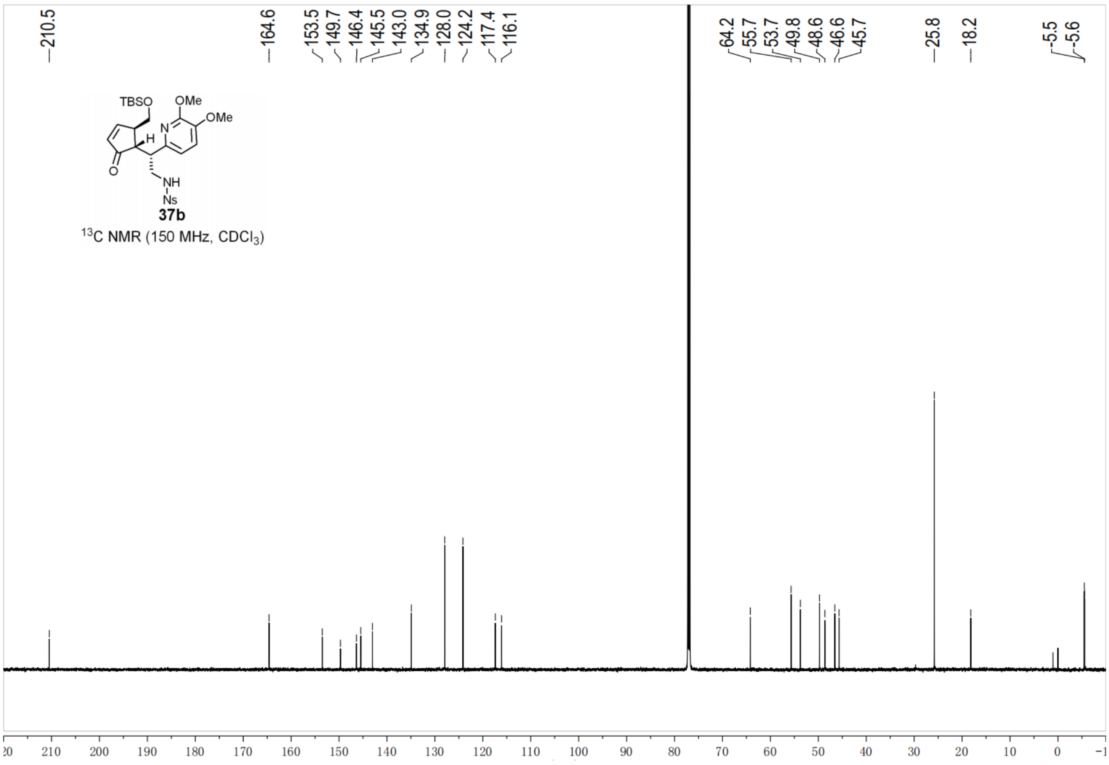
210.5
164.6
153.5
149.7
146.4
145.5
143.0
134.9
128.0
124.2
117.4
116.1
64.2
55.7
53.7
49.8
48.6
46.6
45.7
25.8
18.2
-5.5
-5.6
TBSO
OMe
OMe
N
H
O
NH
Ns
37b
13C NMR (150 MHz, CDCl3)

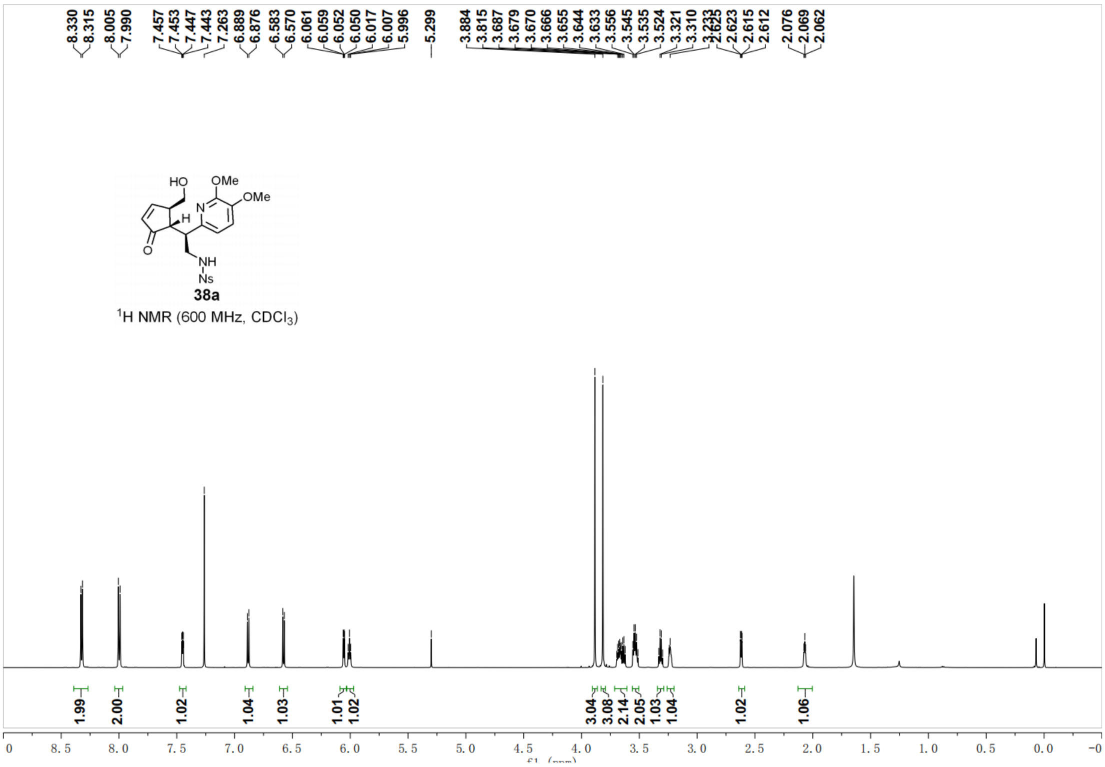

^{1}H NMR (600 MHz, CDCl$_3$)

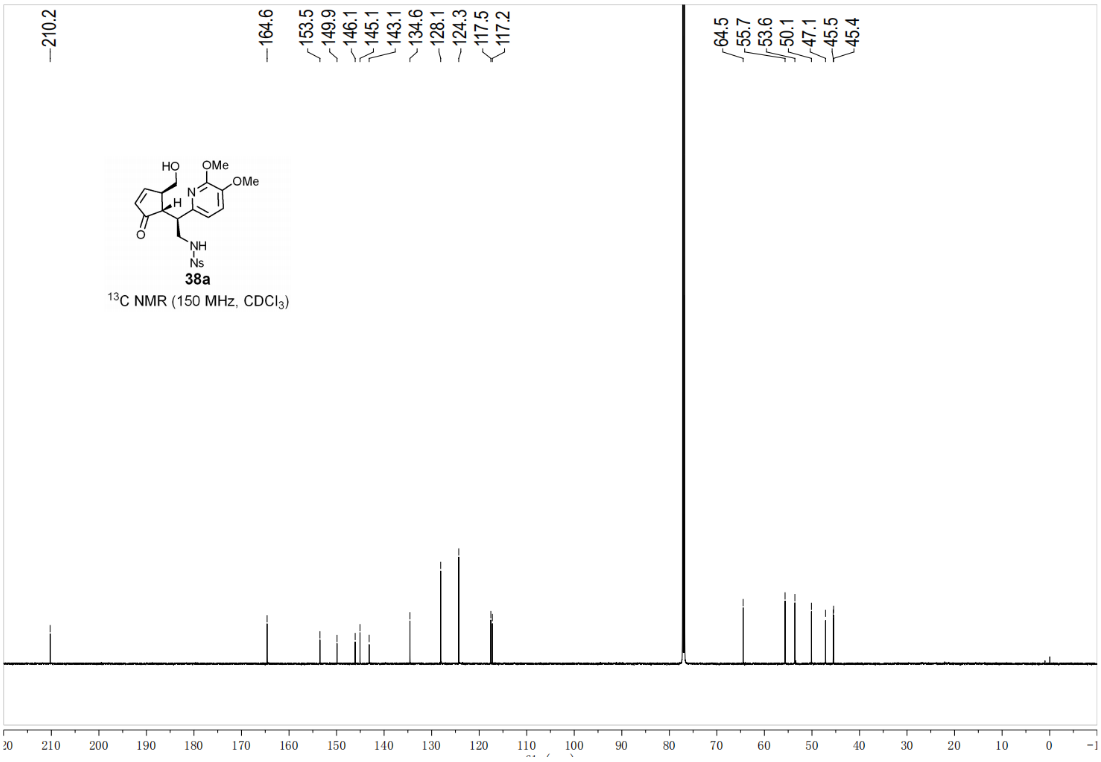

^{13}C NMR (150 MHz, CDCl$_3$)

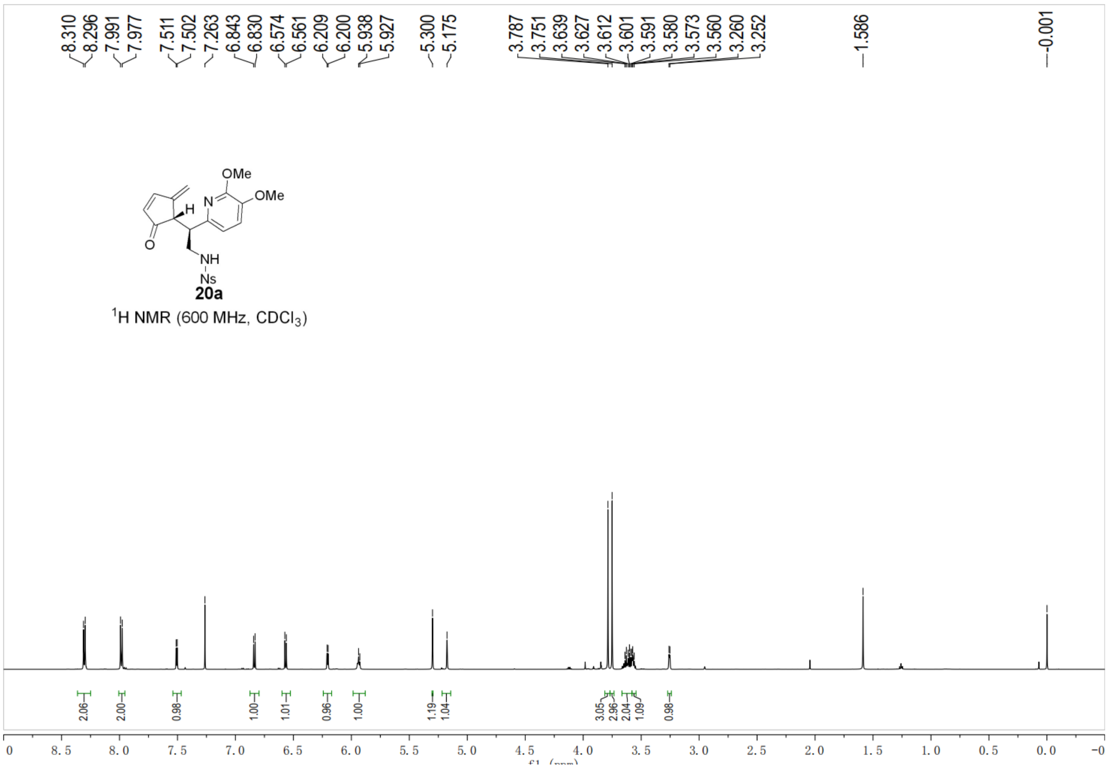

8.310
8.296
7.991
7.977
7.511
7.502
7.263
6.843
6.830
6.574
6.561
6.209
6.200
5.938
5.927
5.300
5.175
3.787
3.751
3.639
3.627
3.612
3.601
3.591
3.580
3.573
3.560
3.260
3.252
1.586
-0.001
OMe
OMe
N
H
O
NH
Ns
20a
^{1}H NMR (600 MHz, CDCl$_3$)
2.06
2.00
0.98
1.00
1.01
0.96
1.00
1.19
1.04
3.05
2.96
2.04
1.09
0.98

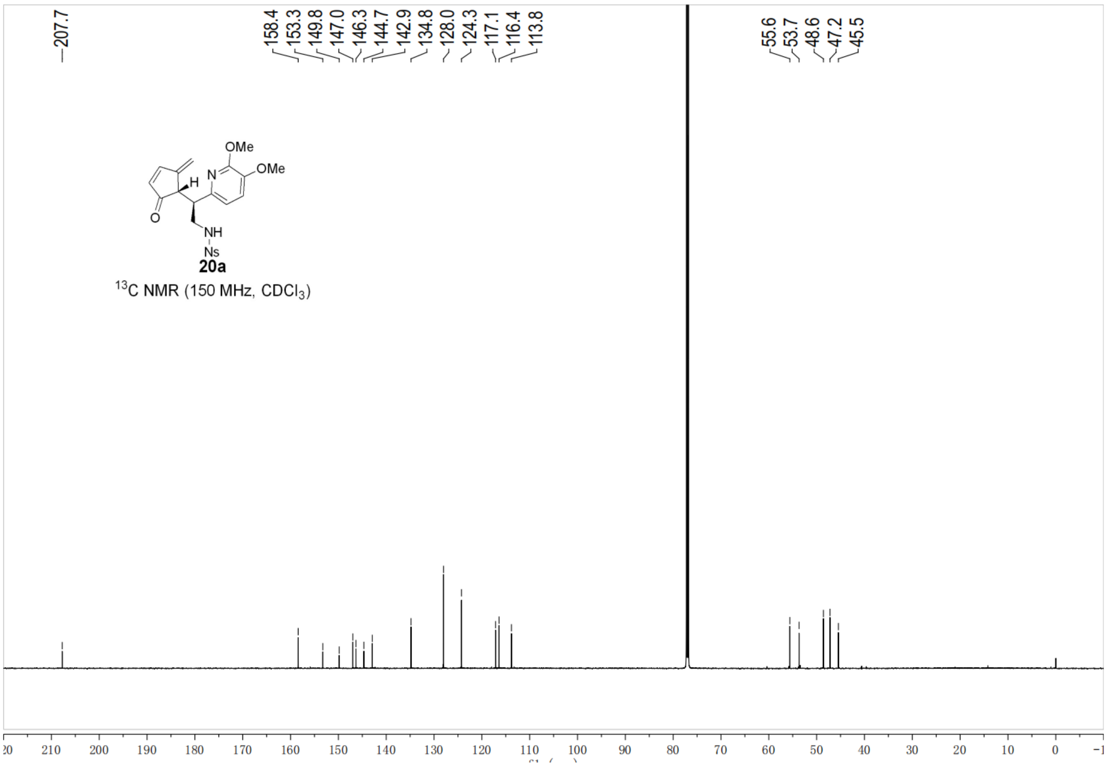

207.7
158.4
153.3
149.8
147.0
146.3
144.7
142.9
134.8
128.0
124.3
117.1
116.4
113.8
55.6
53.7
48.6
47.2
45.5
OMe
OMe
N
H
O
NH
Ns
20a
^{13}C NMR (150 MHz, CDCl$_3$)

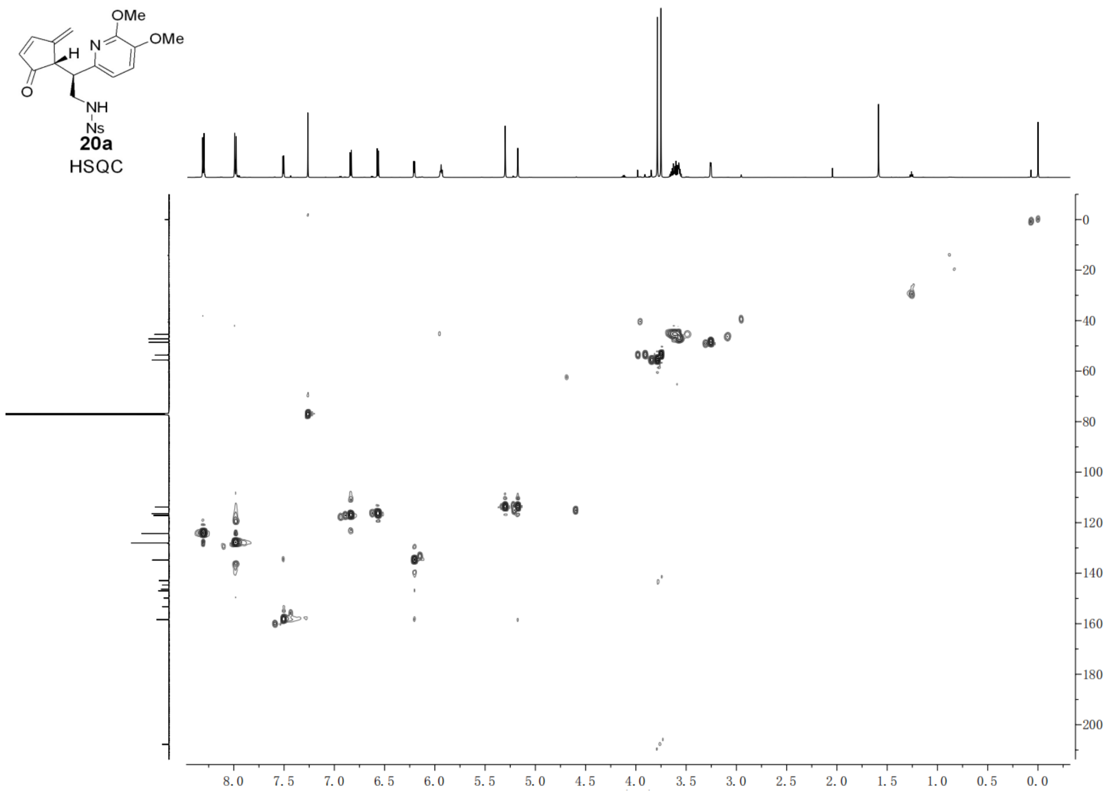
OMe
N
OMe
H
O
NH
Ns
20a
HSQC

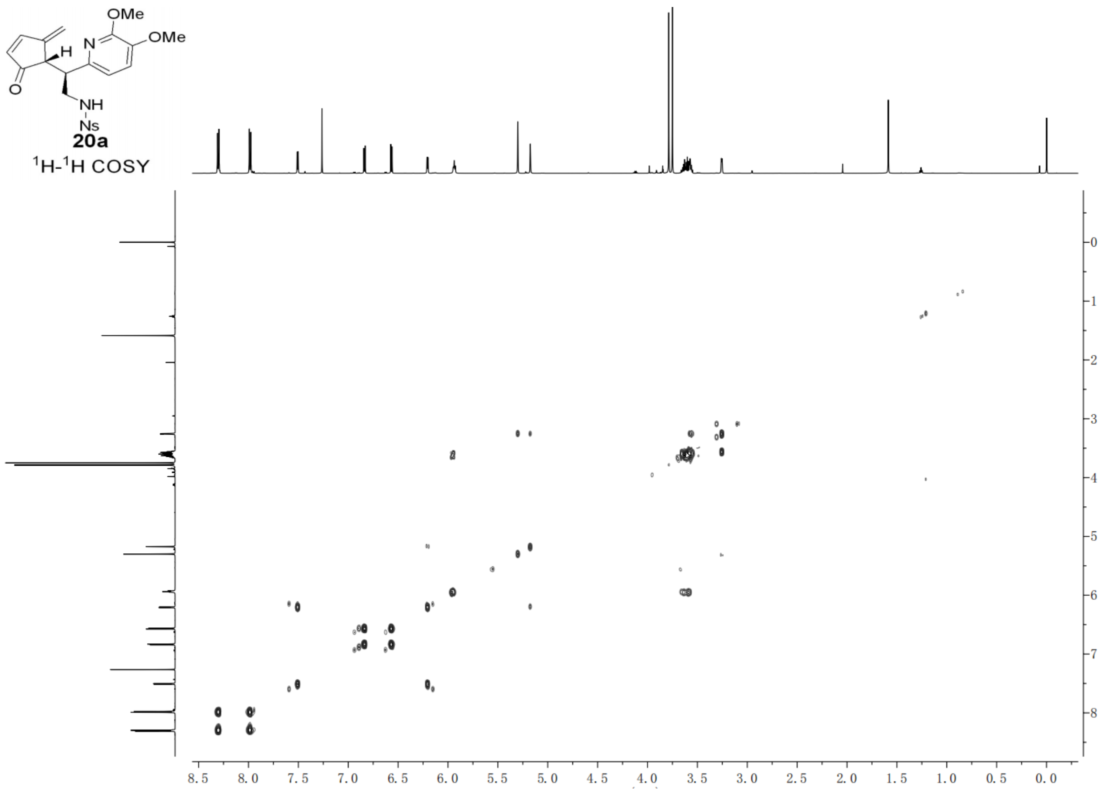
OMe
N
OMe
H
O
NH
Ns
20a
^1H-^1H COSY

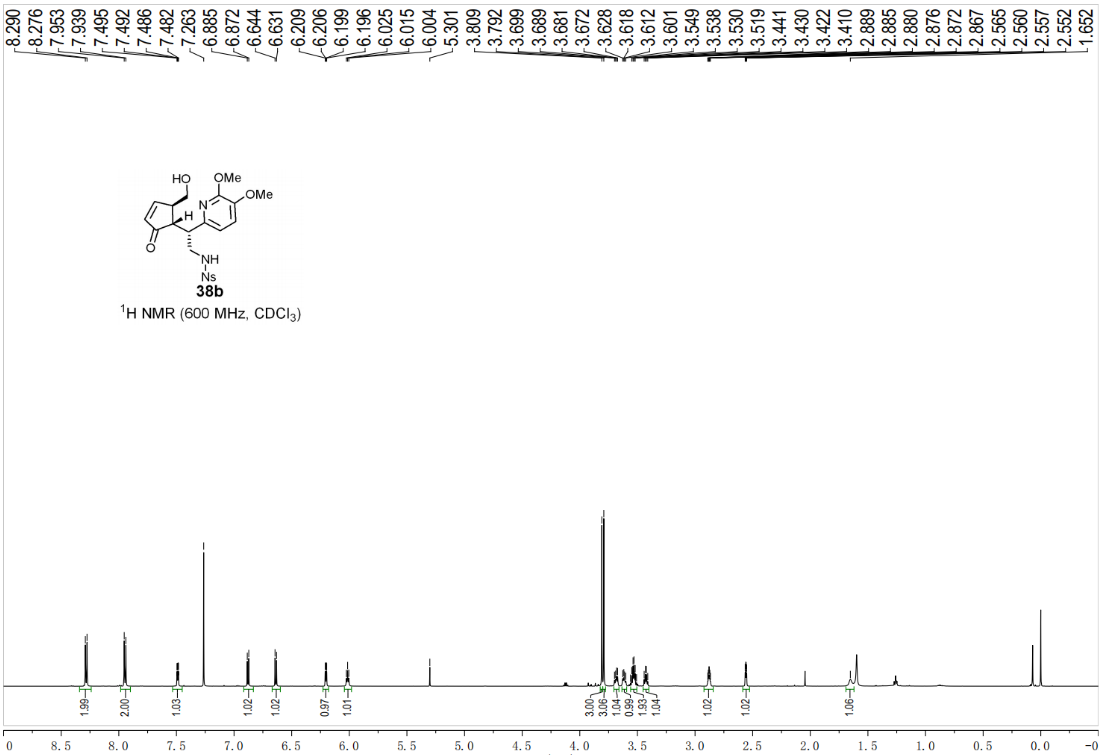
HO
OMe
N
OMe
H
O
NH
Ns
38b
^{1}H NMR (600 MHz, CDCl$_3$)
8.290 8.276 7.953 7.939 7.495 7.492 7.486 7.482 7.263 6.885 6.872 6.644 6.631 6.209 6.206 6.199 6.196 6.025 6.015 6.004 5.301 3.809 3.792 3.699 3.689 3.681 3.672 3.628 3.618 3.612 3.601 3.549 3.538 3.530 3.519 3.441 3.430 3.422 3.410 2.889 2.885 2.880 2.876 2.872 2.867 2.565 2.560 2.557 2.552 1.652
1.99 2.00 1.03 1.02 1.02 0.97 1.01 3.00 3.06 1.04 0.99 1.93 1.04 1.02 1.02 1.06

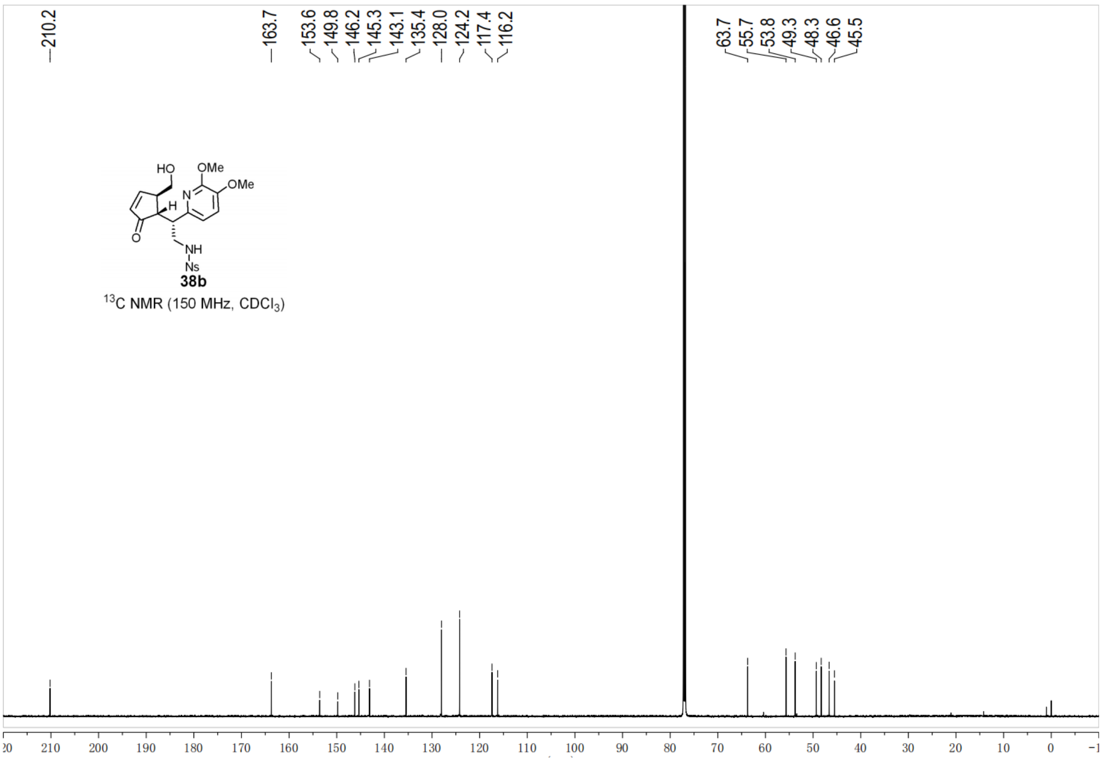
210.2 163.7 153.6 149.8 146.2 145.3 143.1 135.4 128.0 124.2 117.4 116.2 63.7 55.7 53.8 49.3 48.3 46.6 45.5
HO
OMe
N
OMe
H
O
NH
Ns
38b
^{13}C NMR (150 MHz, CDCl$_3$)

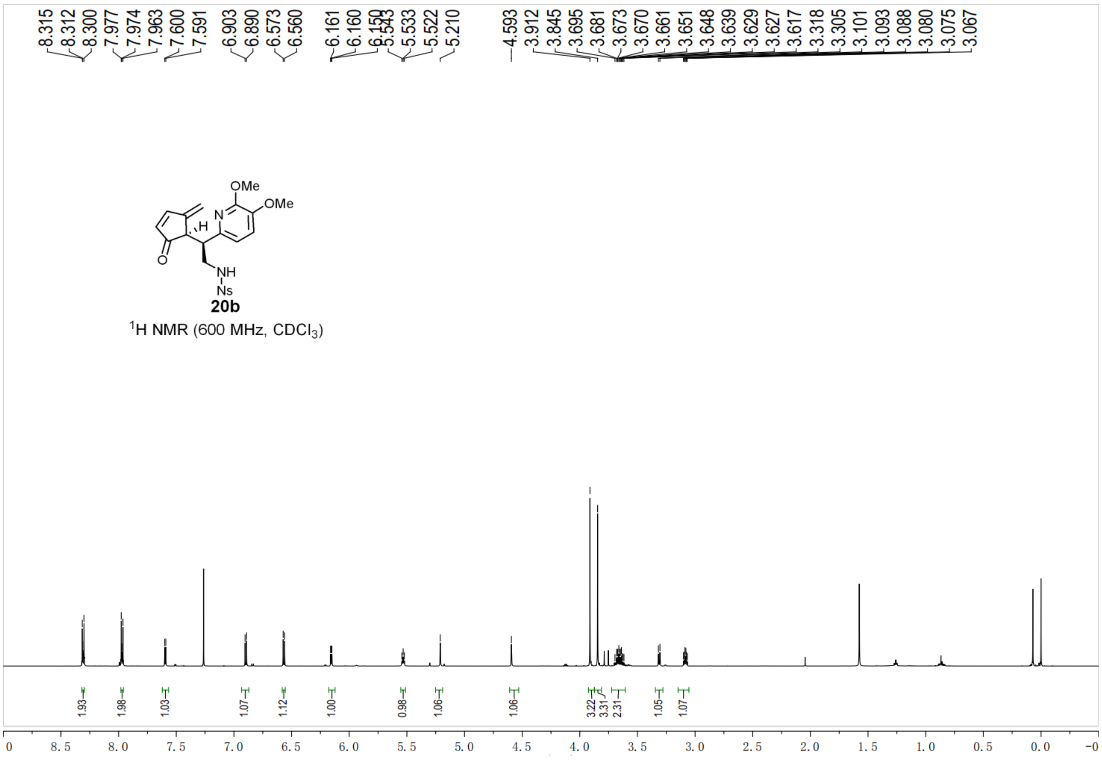
OMe
OMe
N
H
O
NH
Ns
20b
1H NMR (600 MHz, CDCl3)

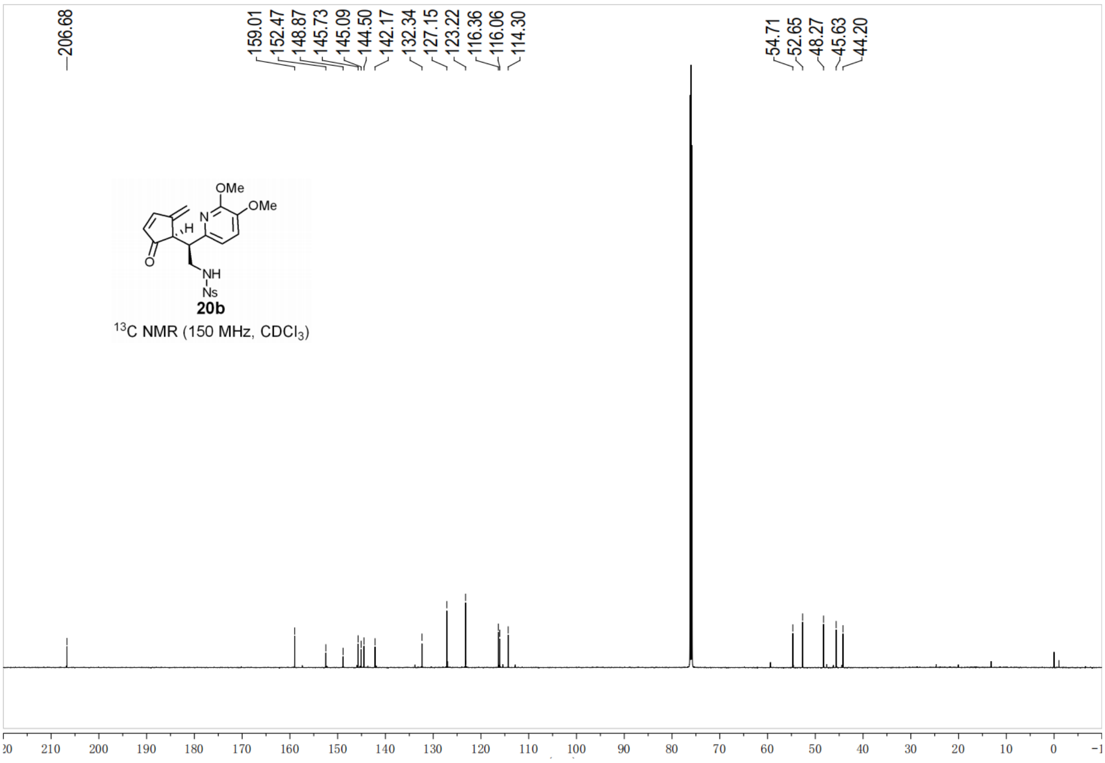
OMe
OMe
N
H
O
NH
Ns
20b
13C NMR (150 MHz, CDCl3)

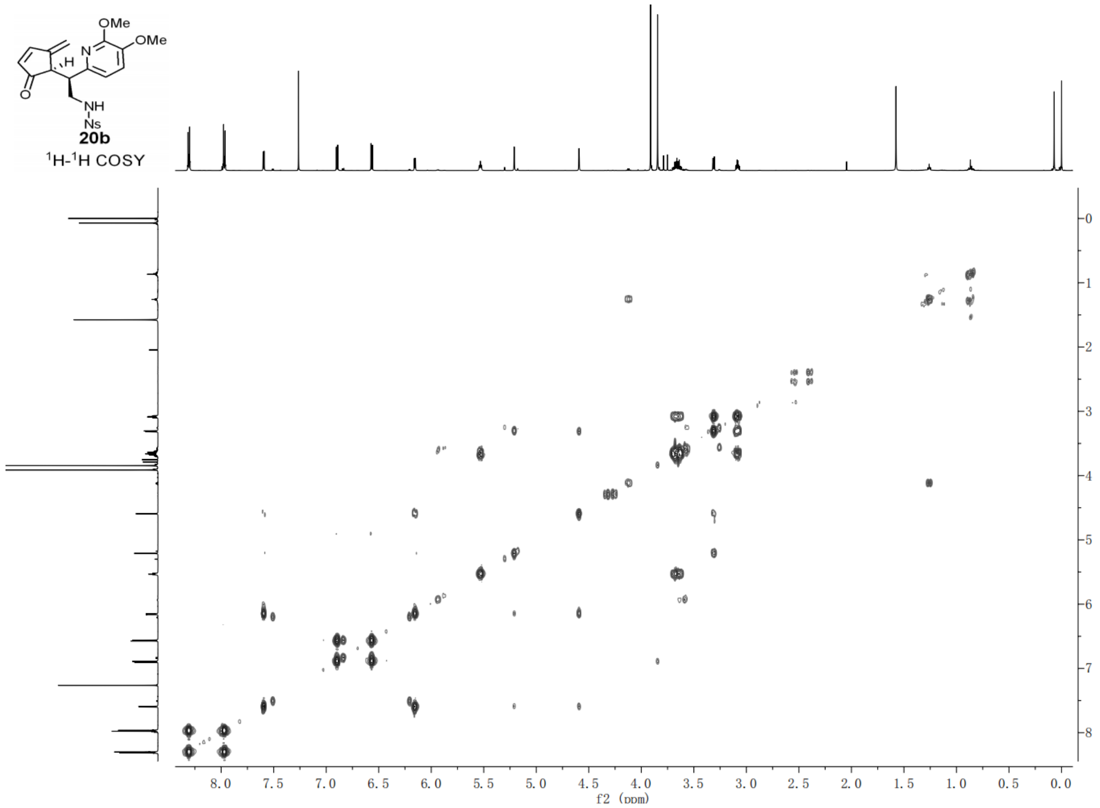

OMe
OMe
N
H
O
NH
Ns
20b
¹H-¹H COSY
8.0 7.5 7.0 6.5 6.0 5.5 5.0 4.5 4.0 3.5 3.0 2.5 2.0 1.5 1.0 0.5 0.0
f2 (ppm)
0 1 2 3 4 5 6 7 8

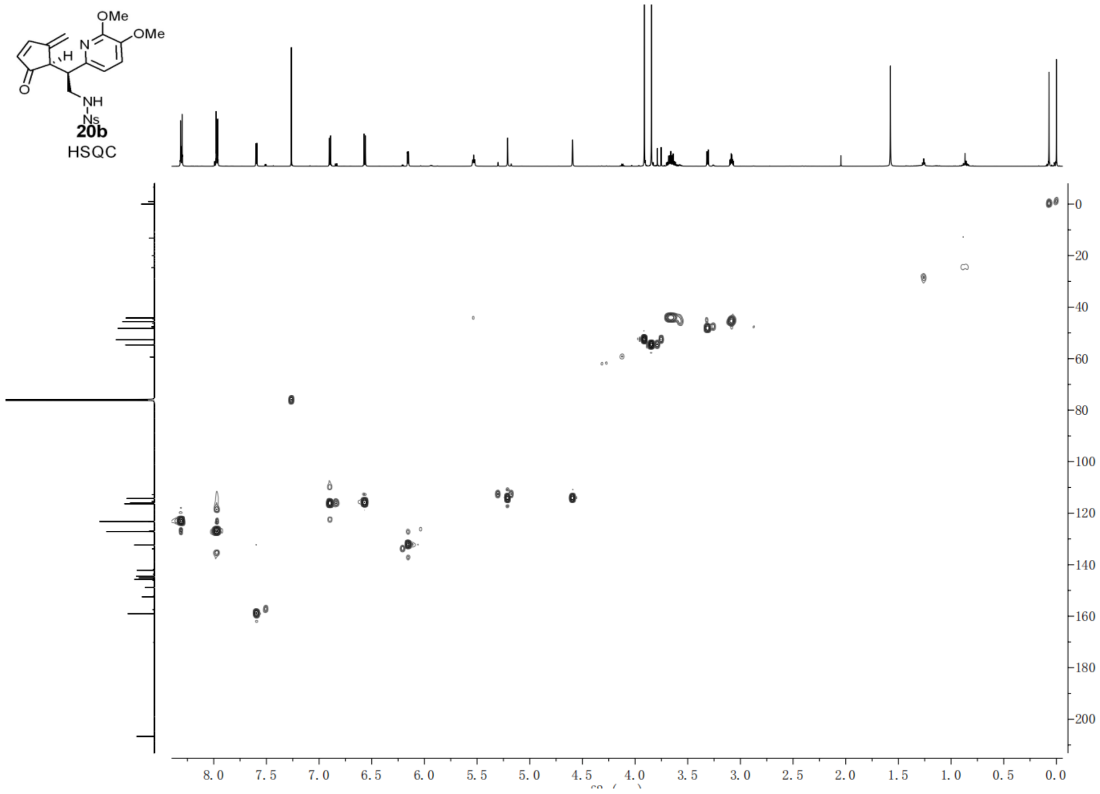

OMe
OMe
N
H
O
NH
Ns
20b
HSQC
8.0 7.5 7.0 6.5 6.0 5.5 5.0 4.5 4.0 3.5 3.0 2.5 2.0 1.5 1.0 0.5 0.0
0 20 40 60 80 100 120 140 160 180 200

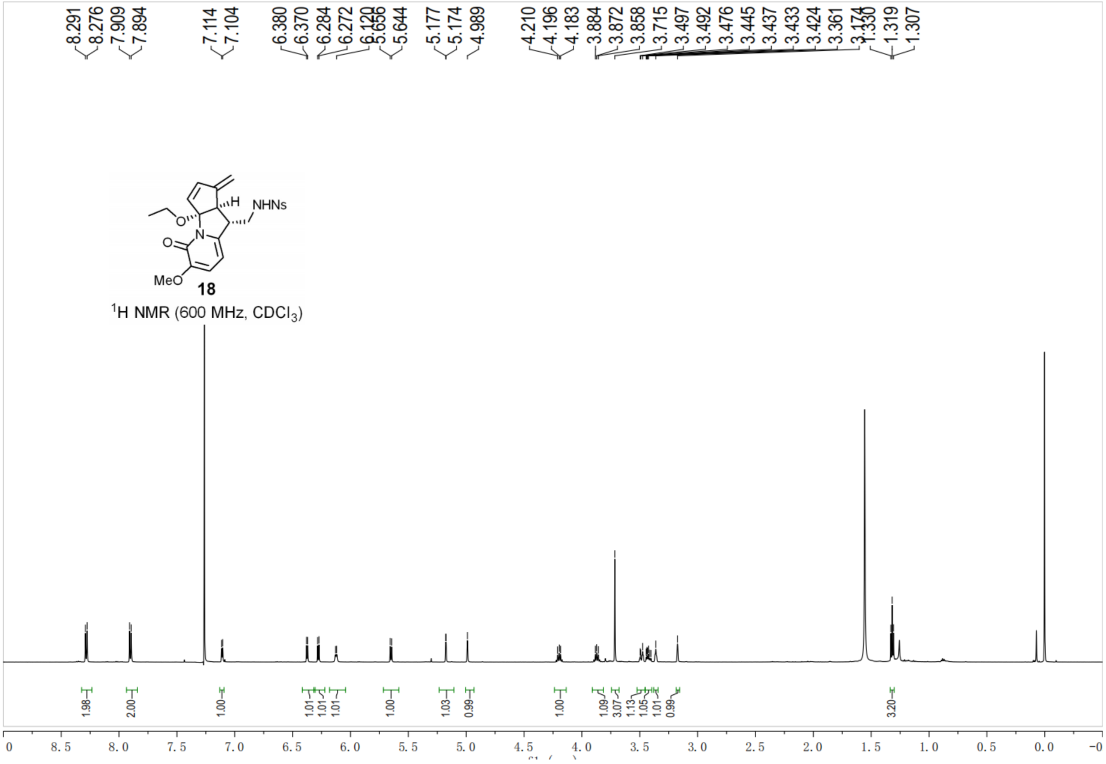

¹H NMR (600 MHz, $CDCl_3$)

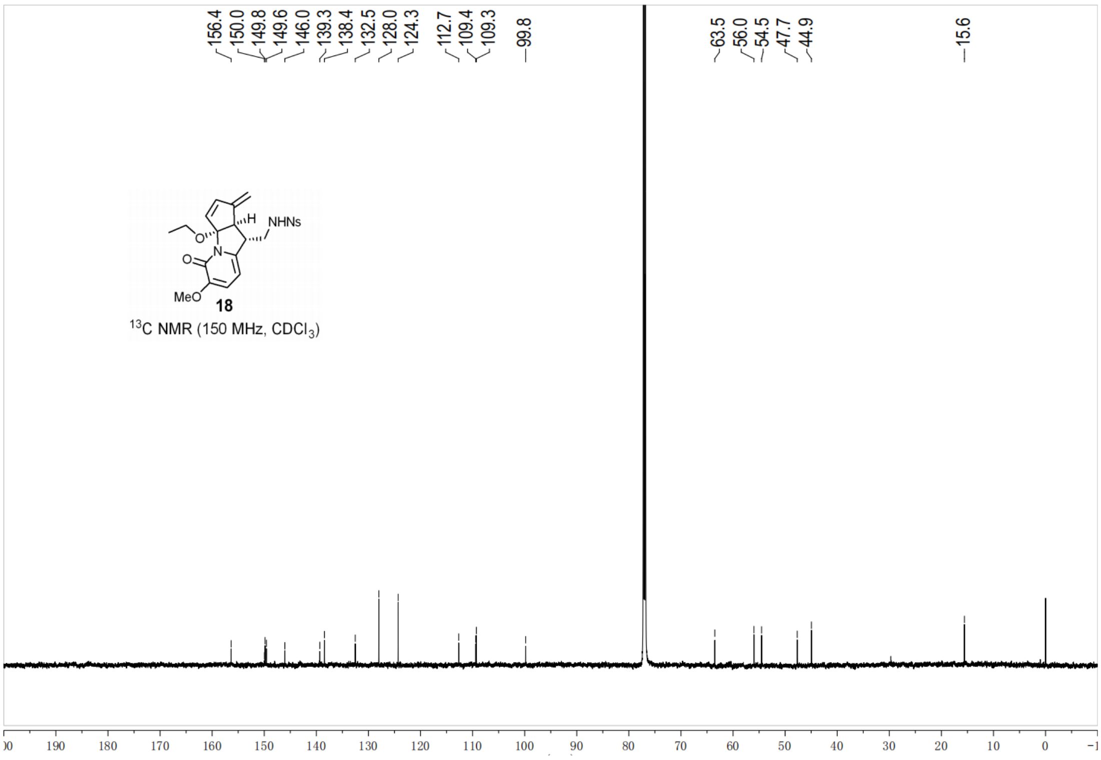

¹³C NMR (150 MHz, $CDCl_3$)

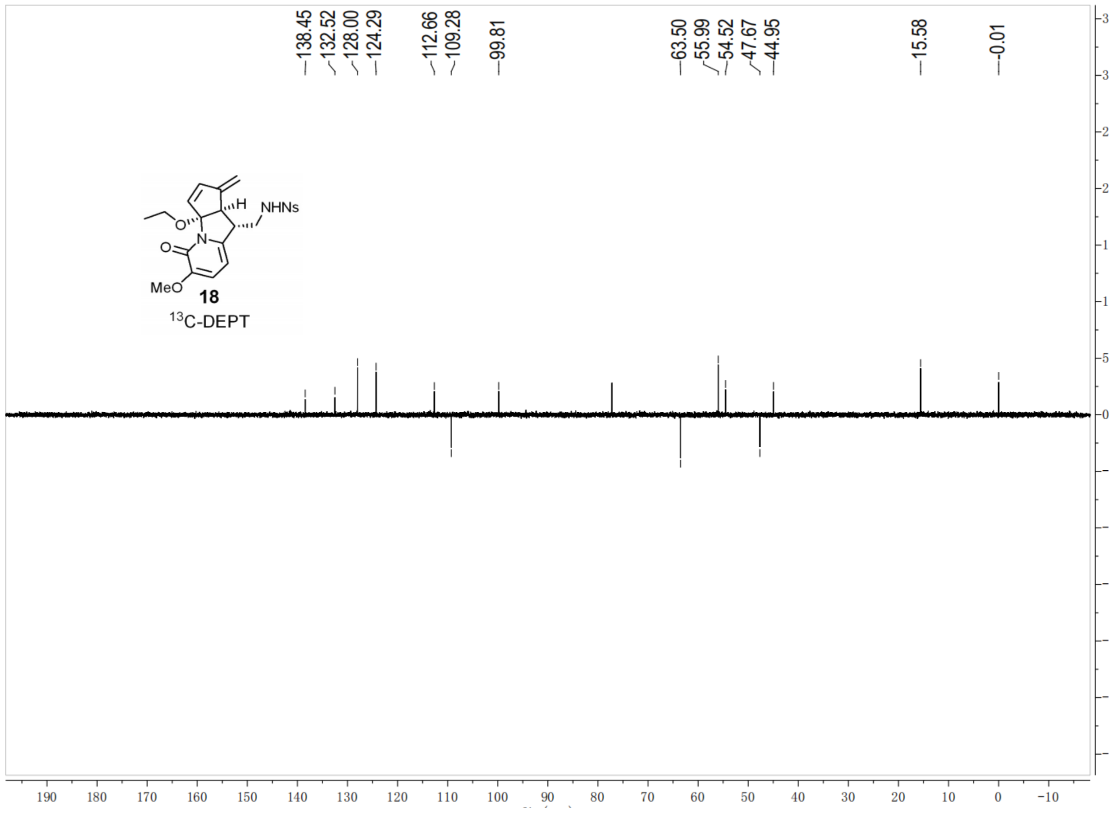
138.45
132.52
128.00
124.29
112.66
109.28
99.81
63.50
55.99
54.52
47.67
44.95
15.58
-0.01
NHNs
MeO
18
13C-DEPT

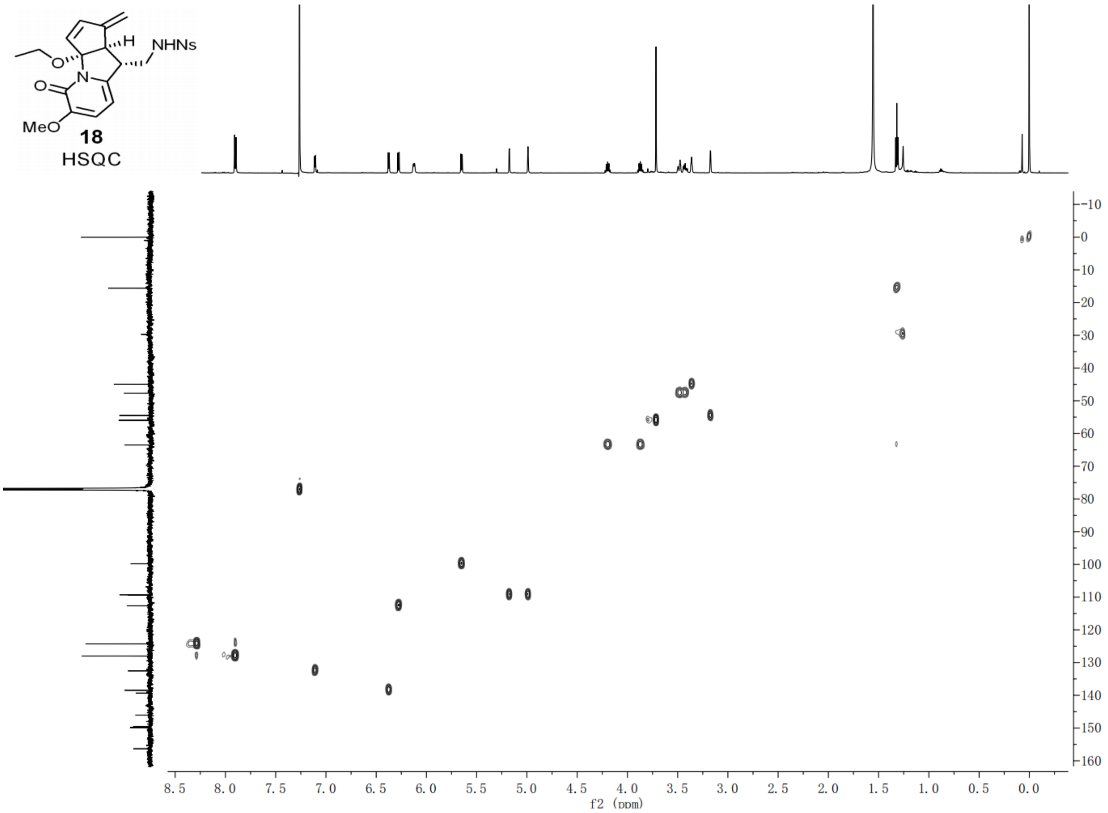
NHNs
MeO
18
HSQC
f2 (ppm)

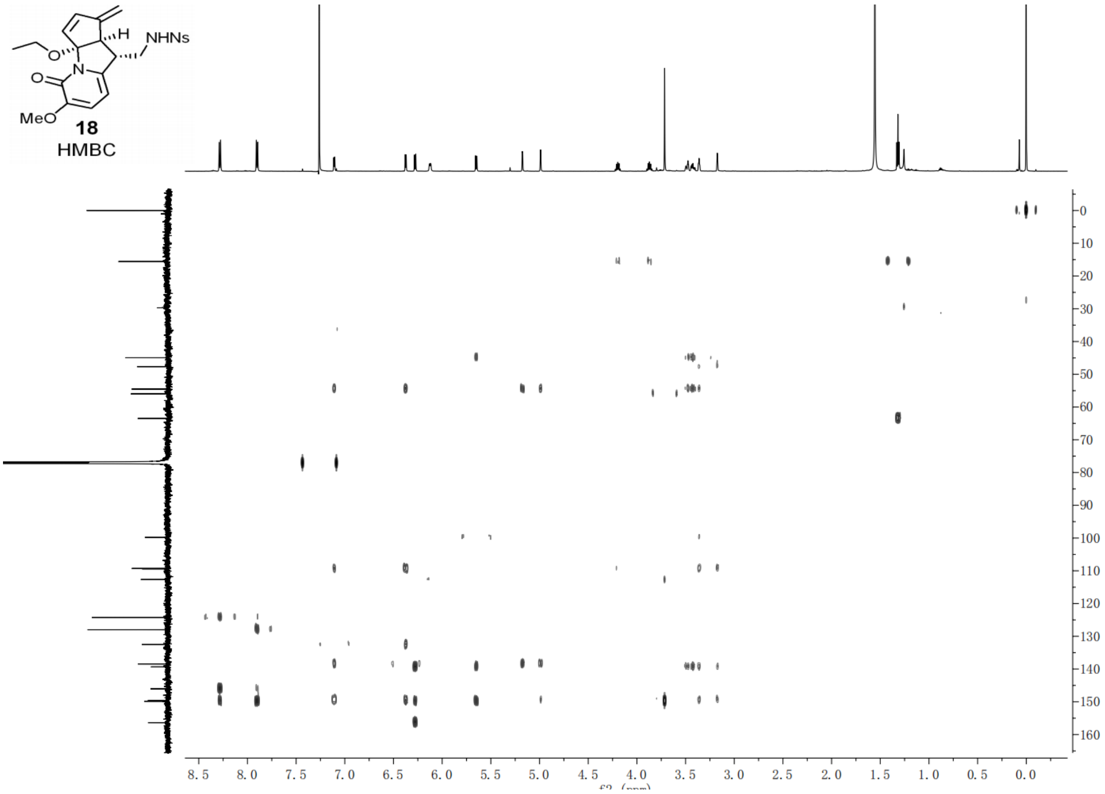
NHNs
N
O
MeO
18
HMBC
0
10
20
30
40
50
60
70
80
90
100
110
120
130
140
150
160
8.5 8.0 7.5 7.0 6.5 6.0 5.5 5.0 4.5 4.0 3.5 3.0 2.5 2.0 1.5 1.0 0.5 0.0

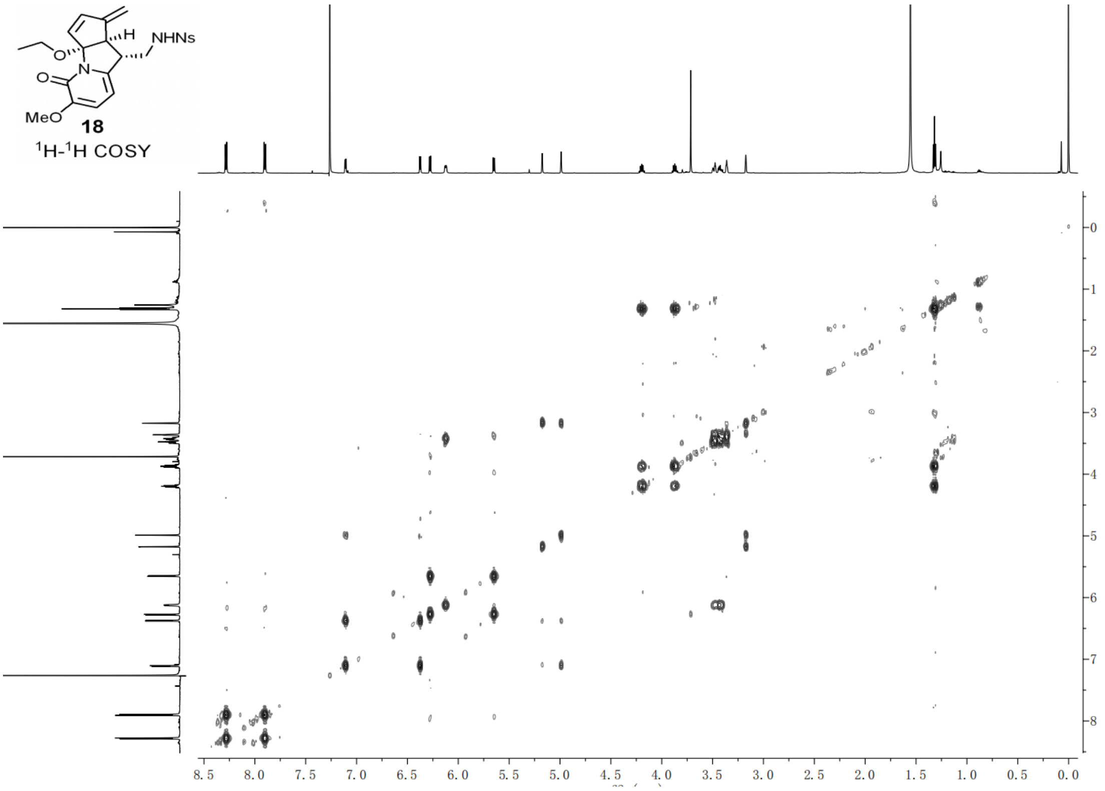
NHNs
N
O
MeO
18
1H-1H COSY
0
1
2
3
4
5
6
7
8
8.5 8.0 7.5 7.0 6.5 6.0 5.5 5.0 4.5 4.0 3.5 3.0 2.5 2.0 1.5 1.0 0.5 0.0

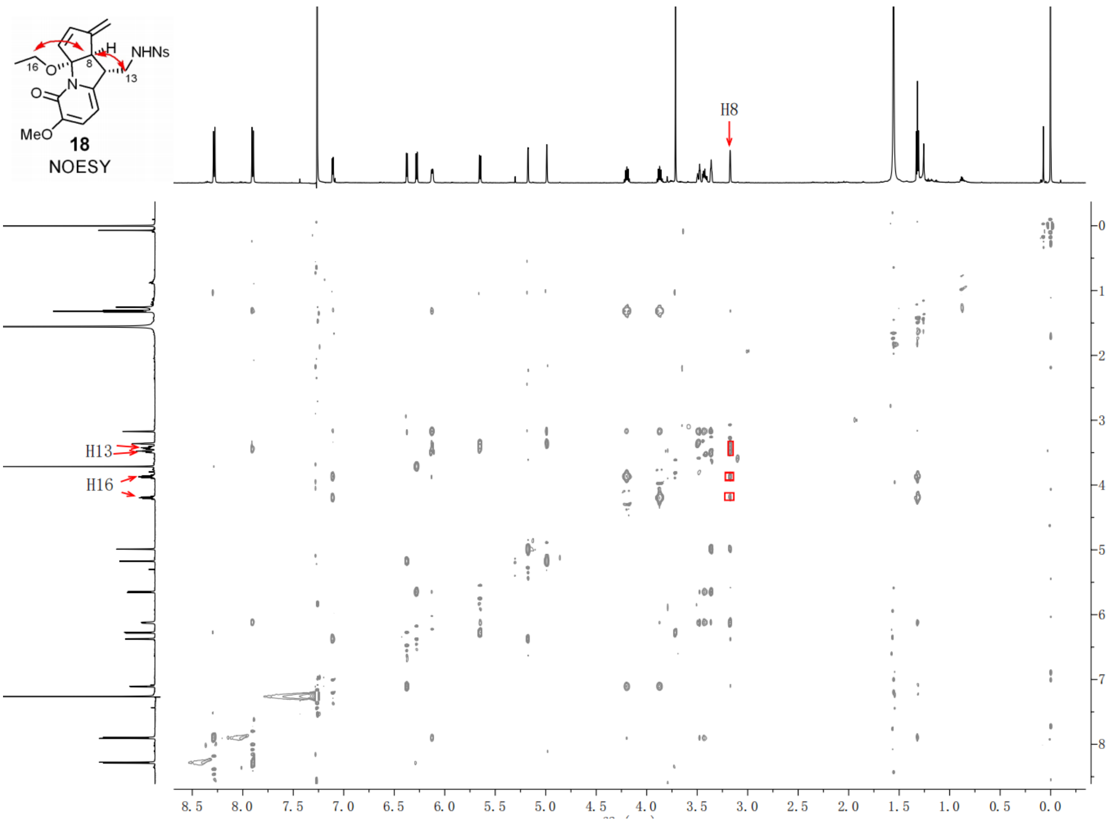
NHNs
H
8
16
13
O
N
MeO
18
NOESY
H8
H13
H16
8.5
8.0
7.5
7.0
6.5
6.0
5.5
5.0
4.5
4.0
3.5
3.0
2.5
2.0
1.5
1.0
0.5
0.0
0
1
2
3
4
5
6
7
8

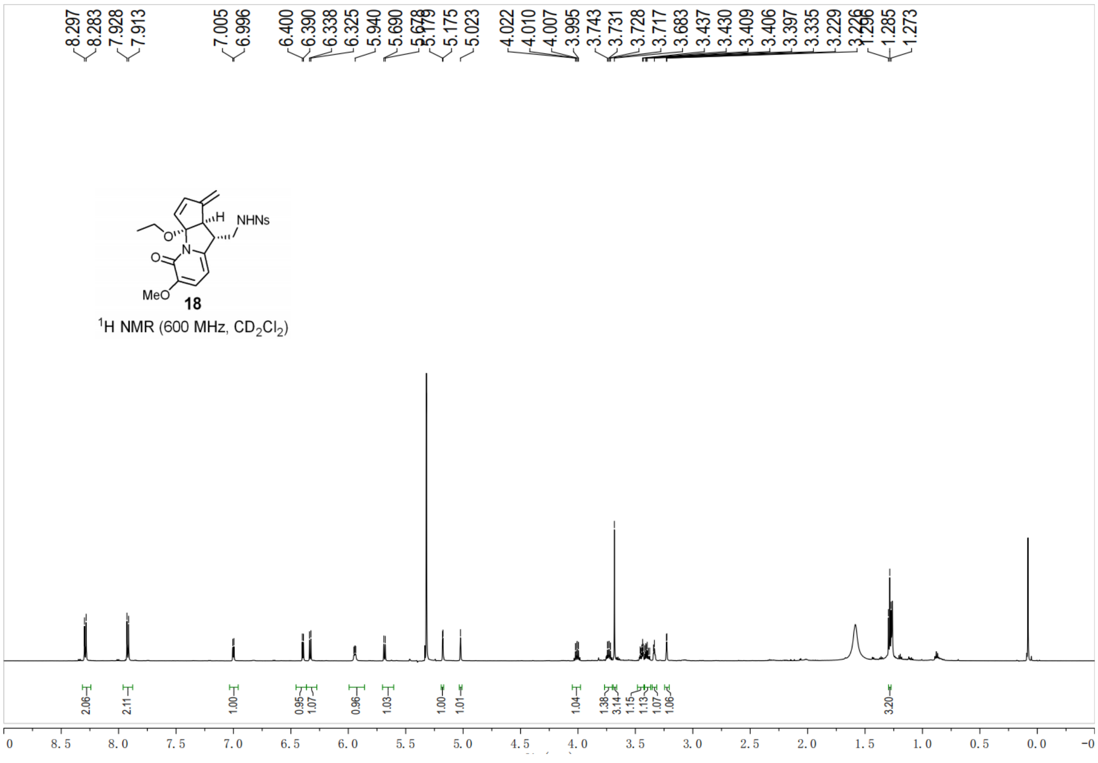
18
^{1}H NMR (600 MHz, CD_2Cl_2)

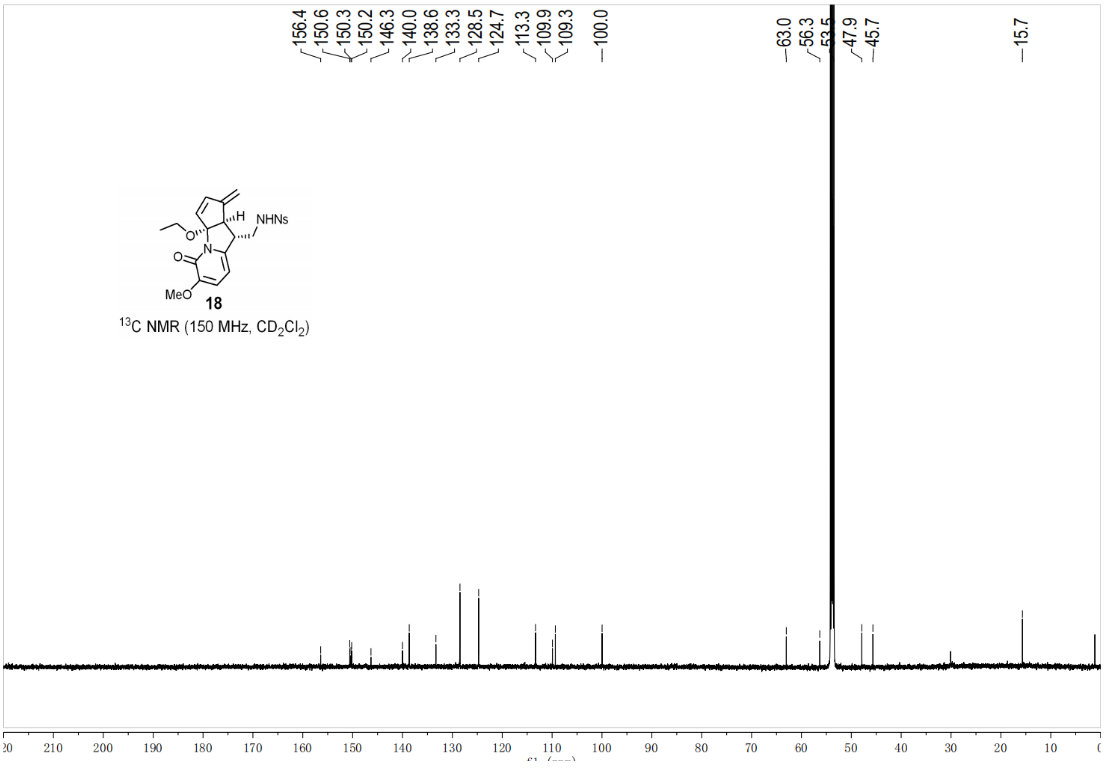
18
^{13}C NMR (150 MHz, CD_2Cl_2)

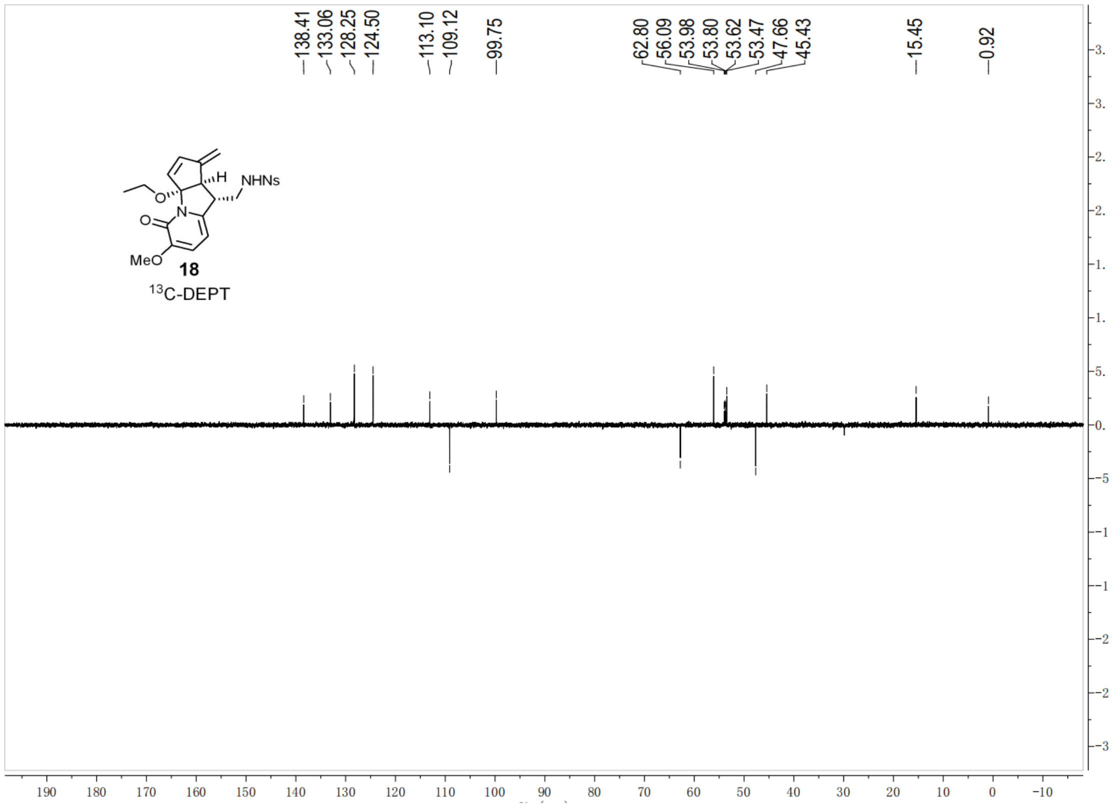
138.41
133.06
128.25
124.50
113.10
109.12
99.75
62.80
56.09
53.98
53.80
53.62
53.47
47.66
45.43
15.45
0.92
NHNs
H
O
N
MeO
18
13C-DEPT

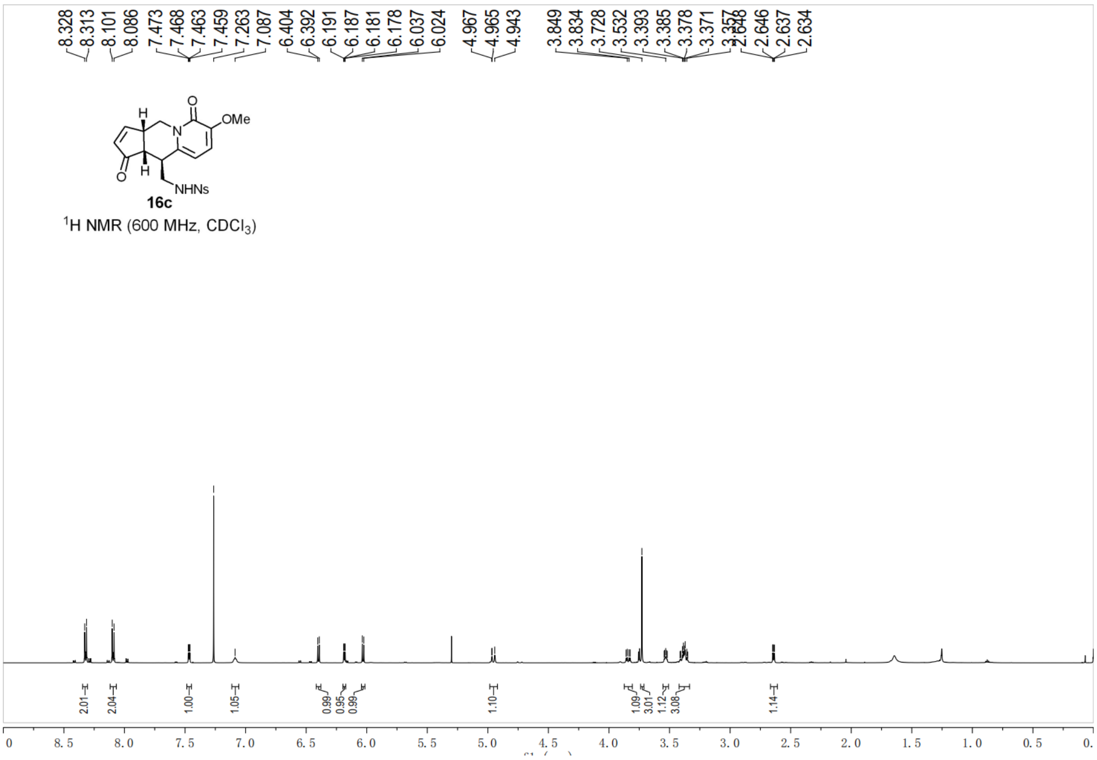
8.328
8.313
8.101
8.086
7.473
7.468
7.463
7.459
7.263
7.087
6.404
6.392
6.191
6.187
6.181
6.178
6.037
6.024
4.967
4.965
4.943
3.849
3.834
3.728
3.532
3.393
3.385
3.378
3.371
2.646
2.637
2.634
OMe
NHNs
16c
^{1}H NMR (600 MHz, $CDCl_3$)
2.01
2.04
1.00
1.05
0.99
0.95
0.99
1.10
1.09
3.01
1.12
3.08
1.14

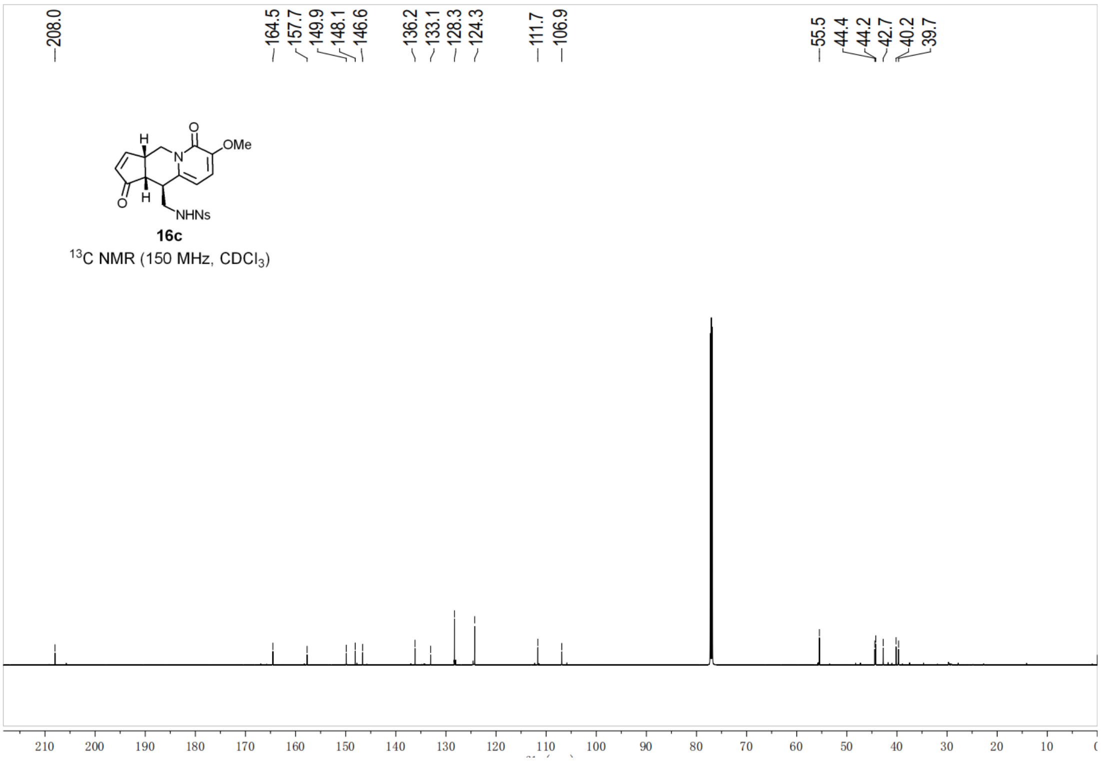
208.0
164.5
157.7
149.9
148.1
146.6
136.2
133.1
128.3
124.3
111.7
106.9
55.5
44.4
44.2
42.7
40.2
39.7
OMe
NHNs
16c
^{13}C NMR (150 MHz, $CDCl_3$)

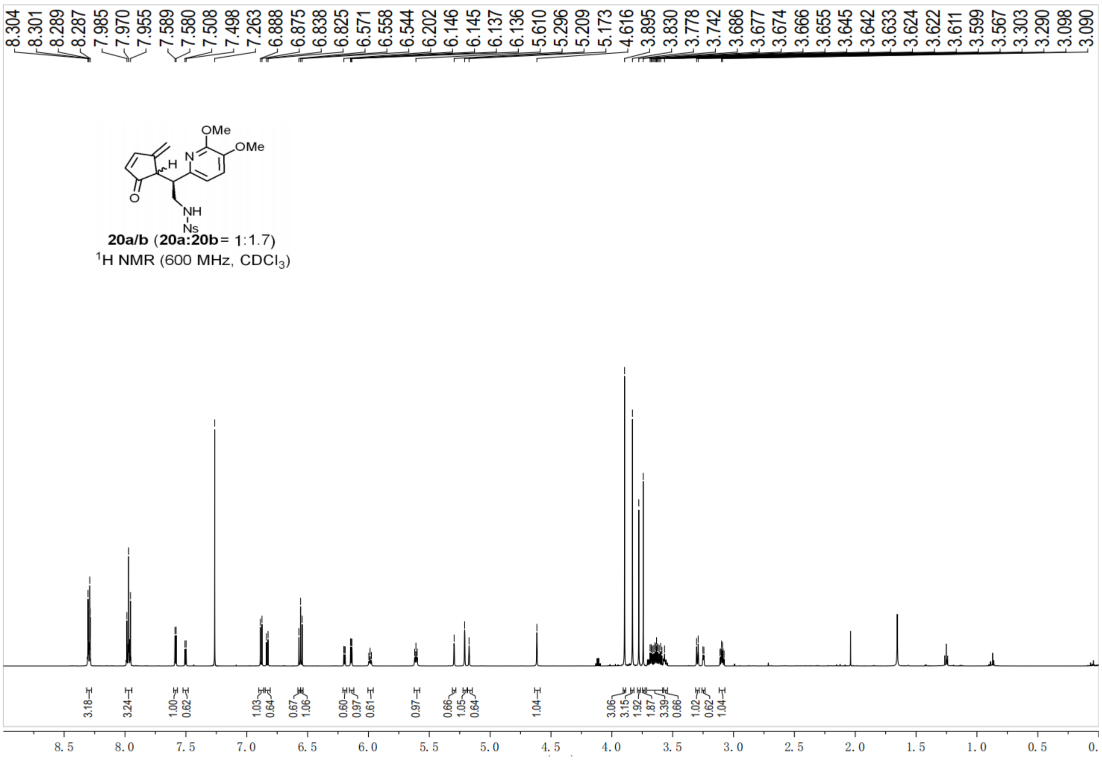
8.304 8.301 8.289 8.287 7.985 7.970 7.955 7.589 7.580 7.508 7.498 7.263 6.888 6.875 6.838 6.825 6.571 6.558 6.544 6.202 6.146 6.145 6.137 6.136 5.610 5.296 5.209 5.173 4.616 3.895 3.830 3.778 3.742 3.686 3.677 3.674 3.666 3.655 3.645 3.642 3.633 3.624 3.622 3.611 3.599 3.567 3.303 3.290 3.098 3.090
OMe
OMe
N
H
O
NH
Ns
20a/b (20a:20b= 1:1.7)
1H NMR (600 MHz, CDCl3)
3.18 3.24 1.00 0.62 1.03 0.64 0.67 1.06 0.60 0.97 0.61 0.97 0.66 1.05 0.64 1.04 3.06 3.15 1.92 1.87 3.39 0.66 1.02 0.62 1.04
8.5 8.0 7.5 7.0 6.5 6.0 5.5 5.0 4.5 4.0 3.5 3.0 2.5 2.0 1.5 1.0 0.5 0.

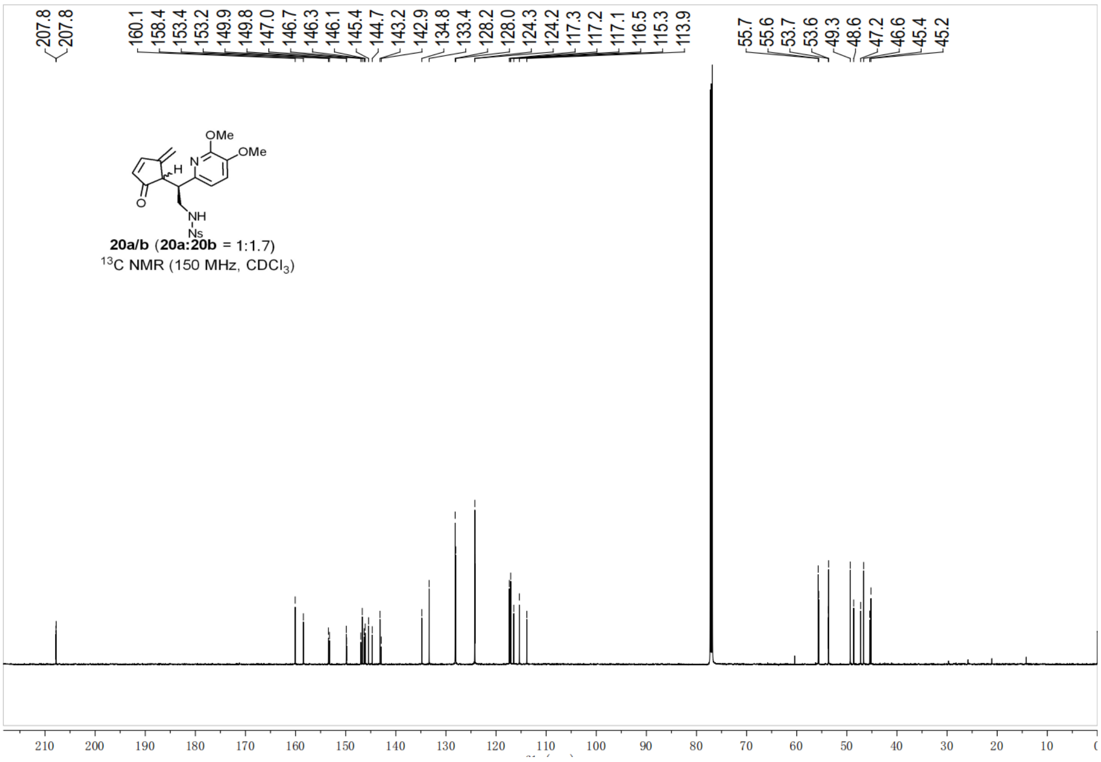
207.8 207.8
160.1 158.4 153.4 153.2 149.9 149.8 147.0 146.7 146.3 146.1 145.4 144.7 143.2 142.9 134.8 133.4 128.2 128.0 124.3 124.2 117.3 117.2 117.1 116.5 115.3 113.9
55.7 55.6 53.7 53.6 49.3 48.6 47.2 46.6 45.4 45.2
OMe
OMe
N
H
O
NH
Ns
20a/b (20a:20b = 1:1.7)
13C NMR (150 MHz, CDCl3)
210 200 190 180 170 160 150 140 130 120 110 100 90 80 70 60 50 40 30 20 10

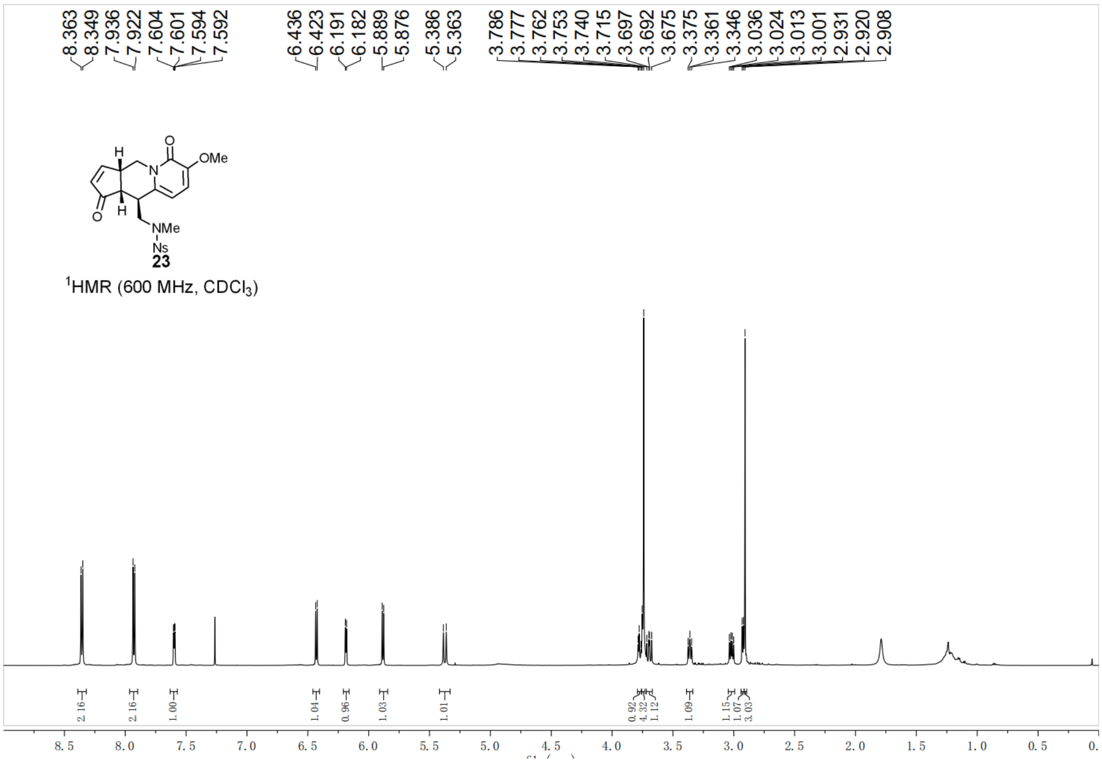
8.363
8.349
7.936
7.922
7.604
7.601
7.594
7.592
6.436
6.423
6.191
6.182
5.889
5.876
5.386
5.363
3.786
3.777
3.762
3.753
3.740
3.715
3.697
3.692
3.675
3.375
3.361
3.346
3.036
3.024
3.013
3.001
2.931
2.920
2.908
H
O
OMe
N
O
H
NMe
Ns
23
[1]HMR (600 MHz, CDCl3)
2.16
2.16
1.00
1.04
0.96
1.03
1.01
0.92
4.32
1.12
1.09
1.15
1.07
3.03
8.5
8.0
7.5
7.0
6.5
6.0
5.5
5.0
4.5
4.0
3.5
3.0
2.5
2.0
1.5
1.0
0.5

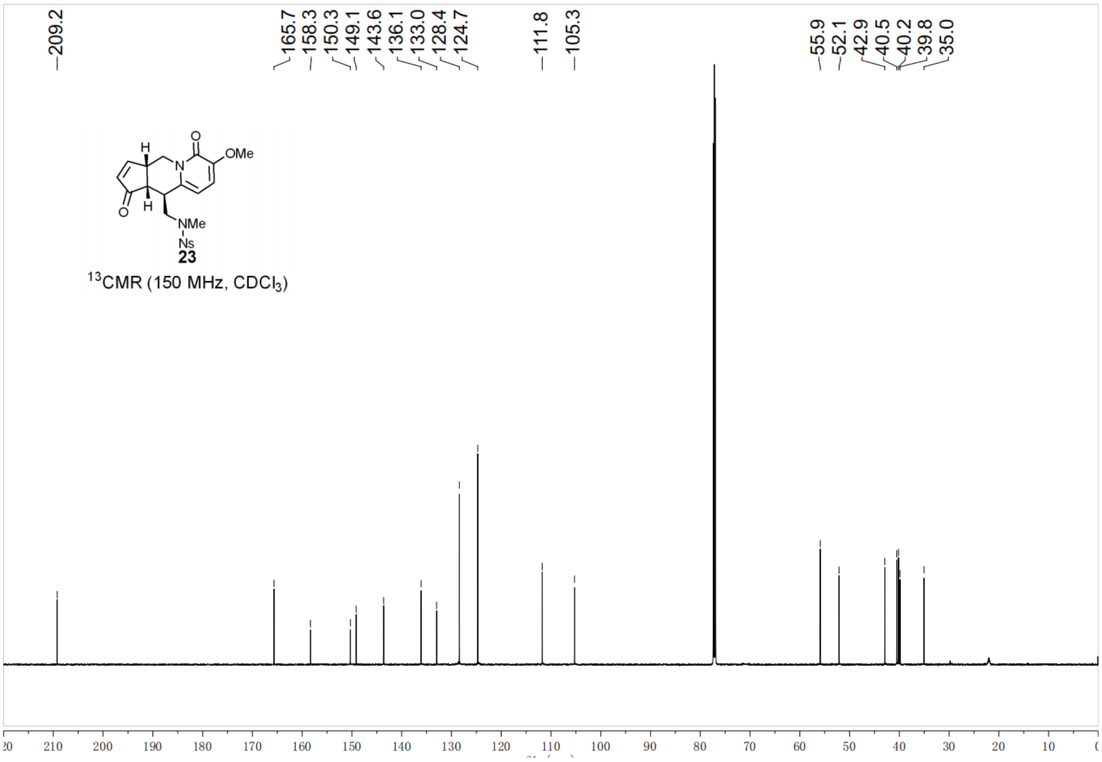
209.2
165.7
158.3
150.3
149.1
143.6
136.1
133.0
128.4
124.7
111.8
105.3
55.9
52.1
42.9
40.5
40.2
39.8
35.0
H
O
OMe
N
O
H
NMe
Ns
23
[13]CMR (150 MHz, CDCl3)
210
200
190
180
170
160
150
140
130
120
110
100
90
80
70
60
50
40
30
20
10

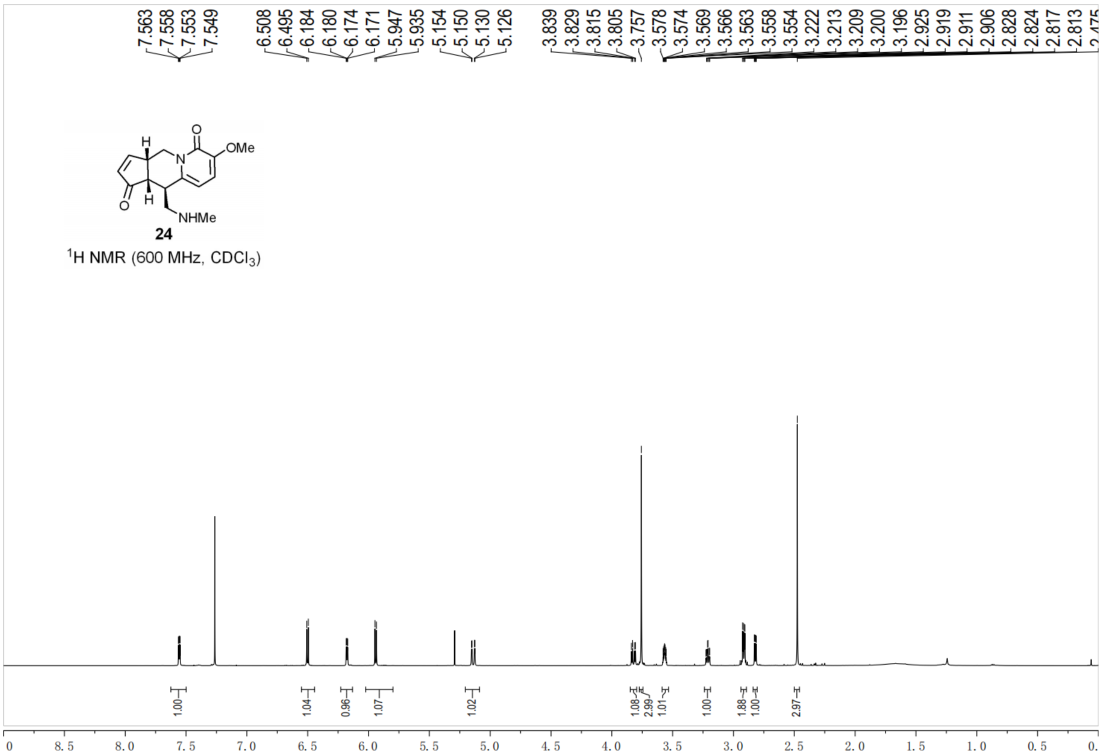
7.563
7.558
7.553
7.549
6.508
6.495
6.184
6.180
6.174
6.171
5.947
5.935
5.154
5.150
5.130
5.126
3.839
3.829
3.815
3.805
3.757
3.578
3.574
3.569
3.566
3.563
3.558
3.554
3.222
3.213
3.209
3.200
3.196
2.925
2.919
2.911
2.906
2.828
2.824
2.817
2.813
OMe
NHMe
24
1H NMR (600 MHz, CDCl3)
1.00
1.04
0.96
1.07
1.02
1.08
2.99
1.01
1.00
1.88
1.00
2.97

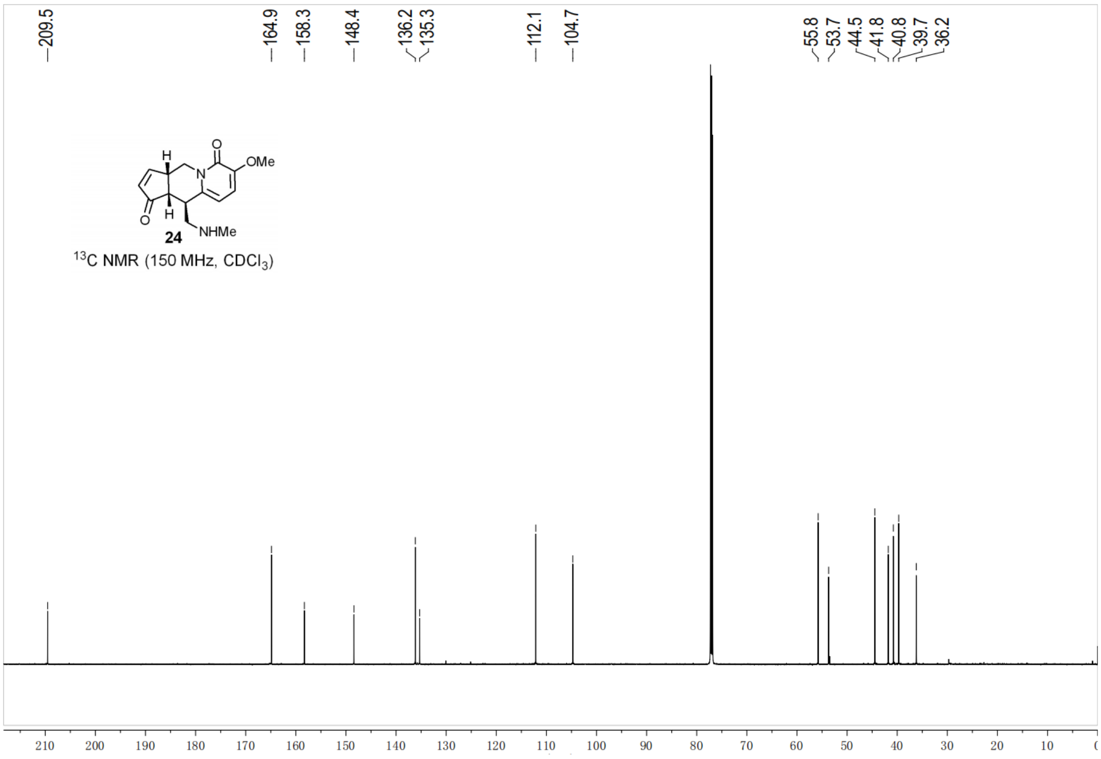
209.5
164.9
158.3
148.4
136.2
135.3
112.1
104.7
55.8
53.7
44.5
41.8
40.8
39.7
36.2
OMe
NHMe
24
13C NMR (150 MHz, CDCl3)

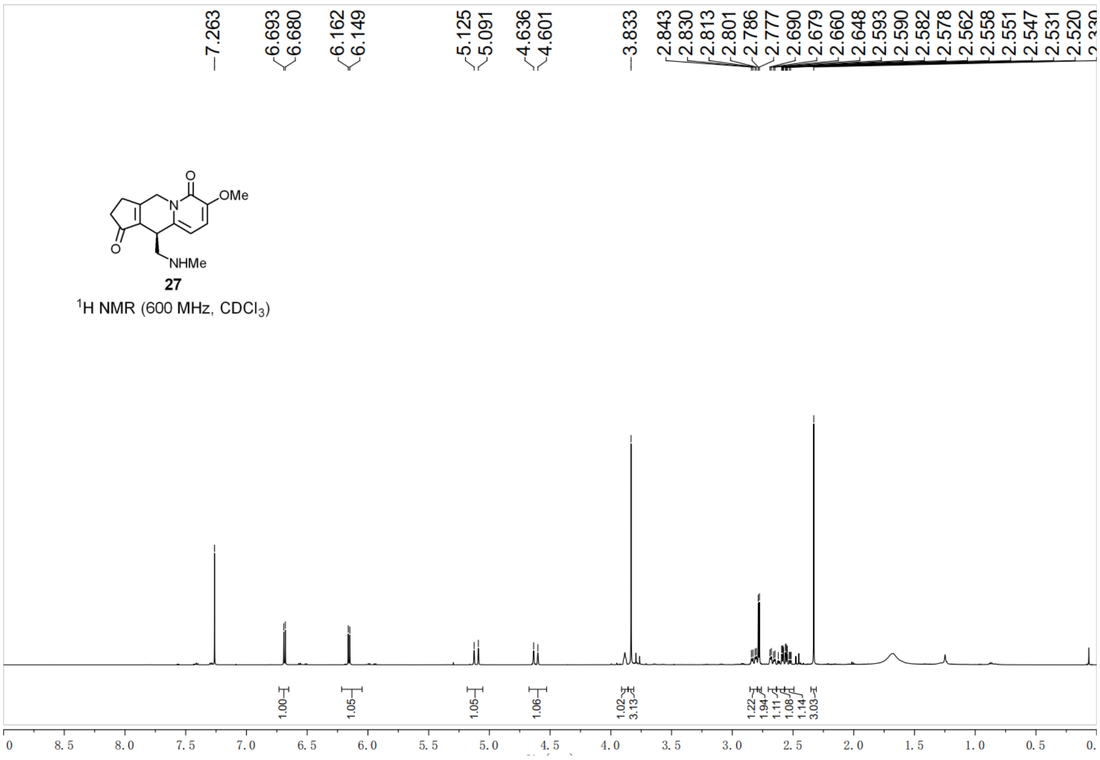
7.263
6.693
6.680
6.162
6.149
5.125
5.091
4.636
4.601
3.833
2.843
2.830
2.813
2.801
2.786
2.777
2.690
2.679
2.660
2.648
2.593
2.590
2.582
2.578
2.562
2.558
2.551
2.547
2.531
2.520
OMe
NHMe
27
1H NMR (600 MHz, CDCl3)
1.00
1.05
1.05
1.06
1.02
3.13
1.22
1.94
1.11
1.08
1.14
3.03

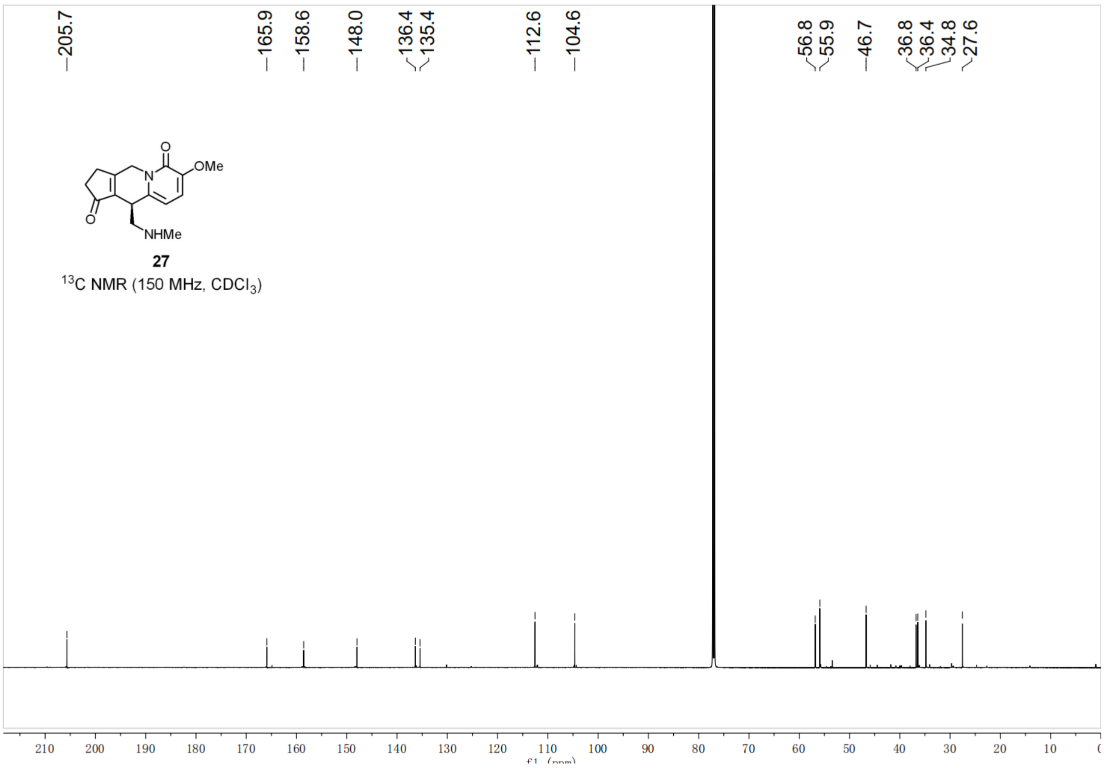
205.7
165.9
158.6
148.0
136.4
135.4
112.6
104.6
56.8
55.9
46.7
36.8
36.4
34.8
27.6
OMe
NHMe
27
13C NMR (150 MHz, CDCl3)

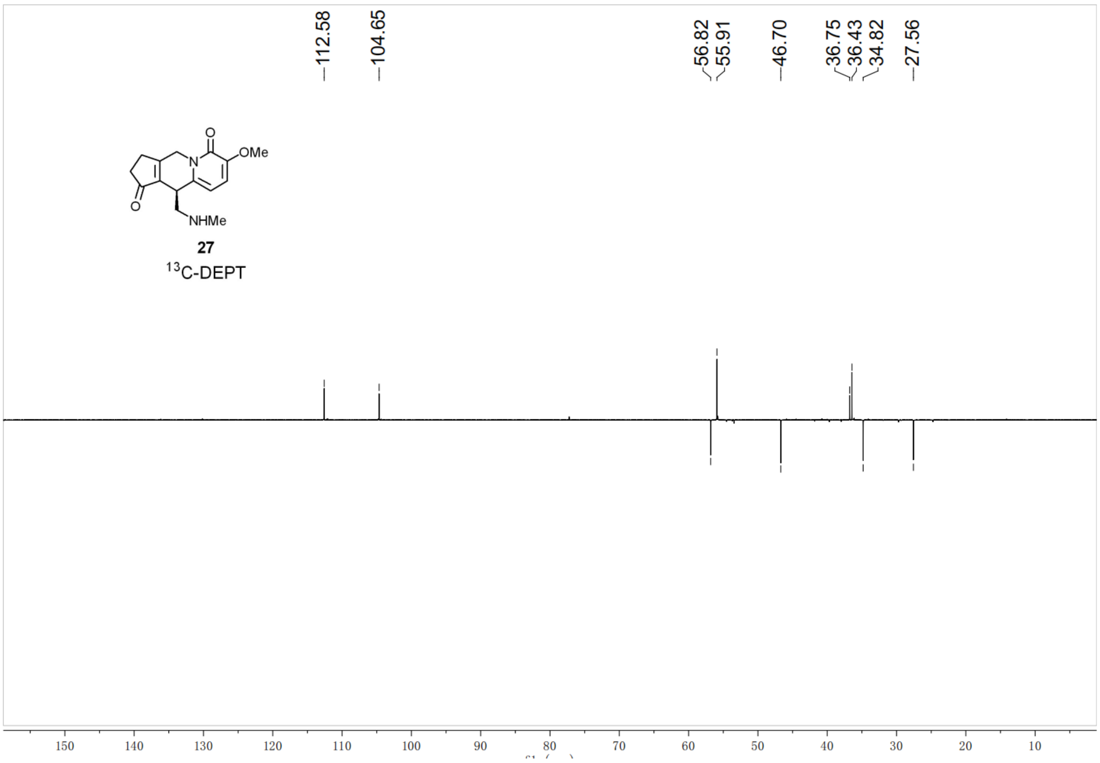

112.58
104.65
56.82
55.91
46.70
36.75
36.43
34.82
27.56
OMe
NHMe
27
^{13}C-DEPT
150
140
130
120
110
100
90
80
70
60
50
40
30
20
10

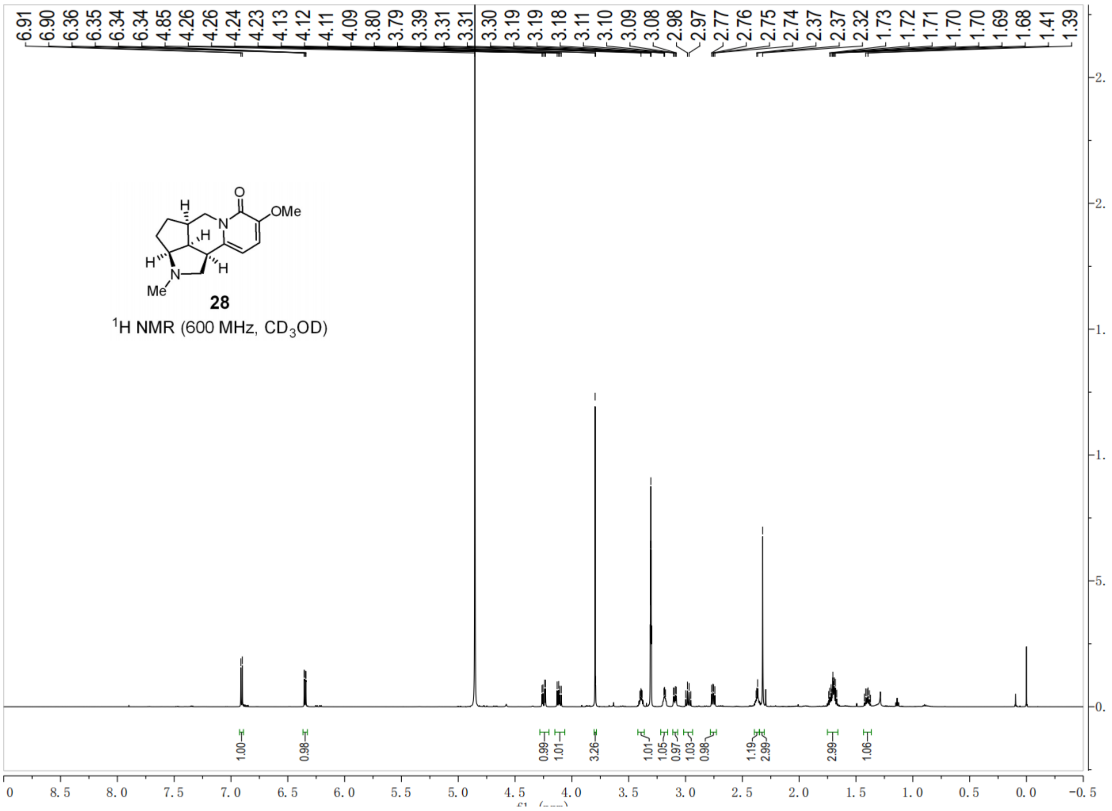

6.91
6.90
6.36
6.35
6.34
6.34
4.85
4.26
4.26
4.24
4.23
4.13
4.12
4.11
4.09
3.80
3.79
3.39
3.31
3.31
3.30
3.19
3.19
3.18
3.11
3.10
3.09
3.08
2.98
2.97
2.77
2.76
2.75
2.74
2.37
2.37
2.32
1.73
1.72
1.71
1.70
1.70
1.69
1.68
1.41
1.39
28
^{1}H NMR (600 MHz, CD$_3$OD)

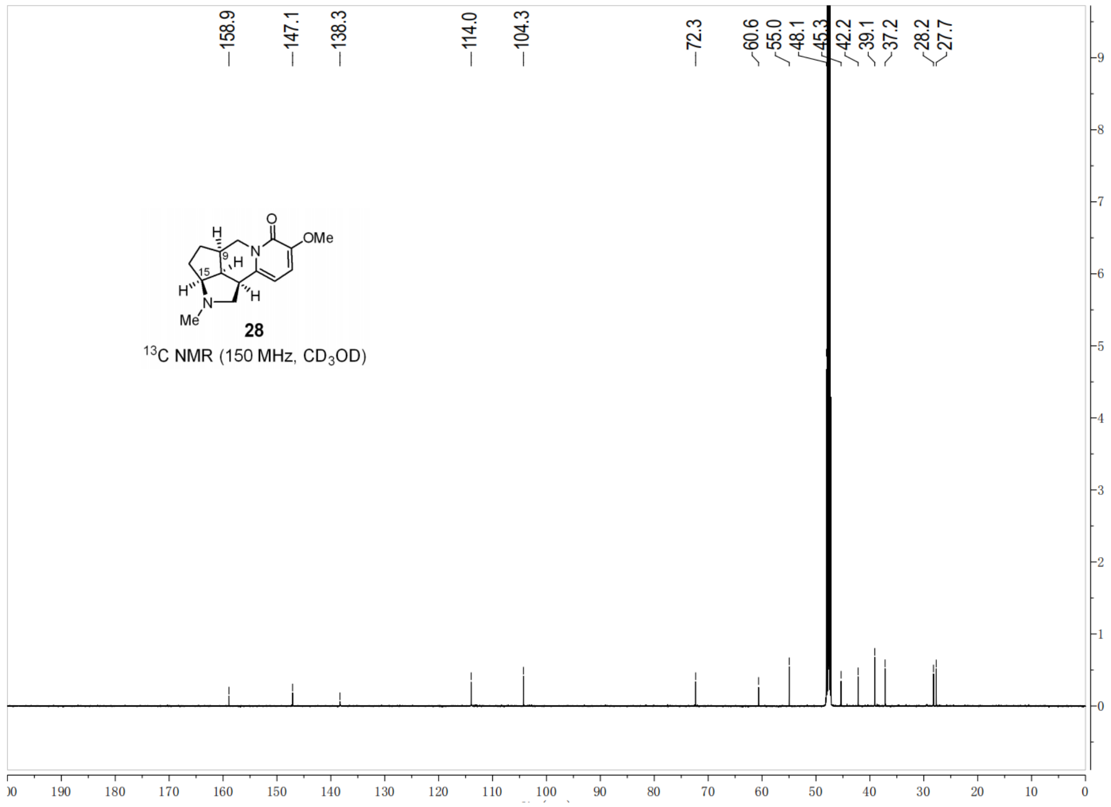

158.9
147.1
138.3
114.0
104.3
72.3
60.6
55.0
48.1
45.3
42.2
39.1
37.2
28.2
27.7
28
^{13}C NMR (150 MHz, CD$_3$OD)

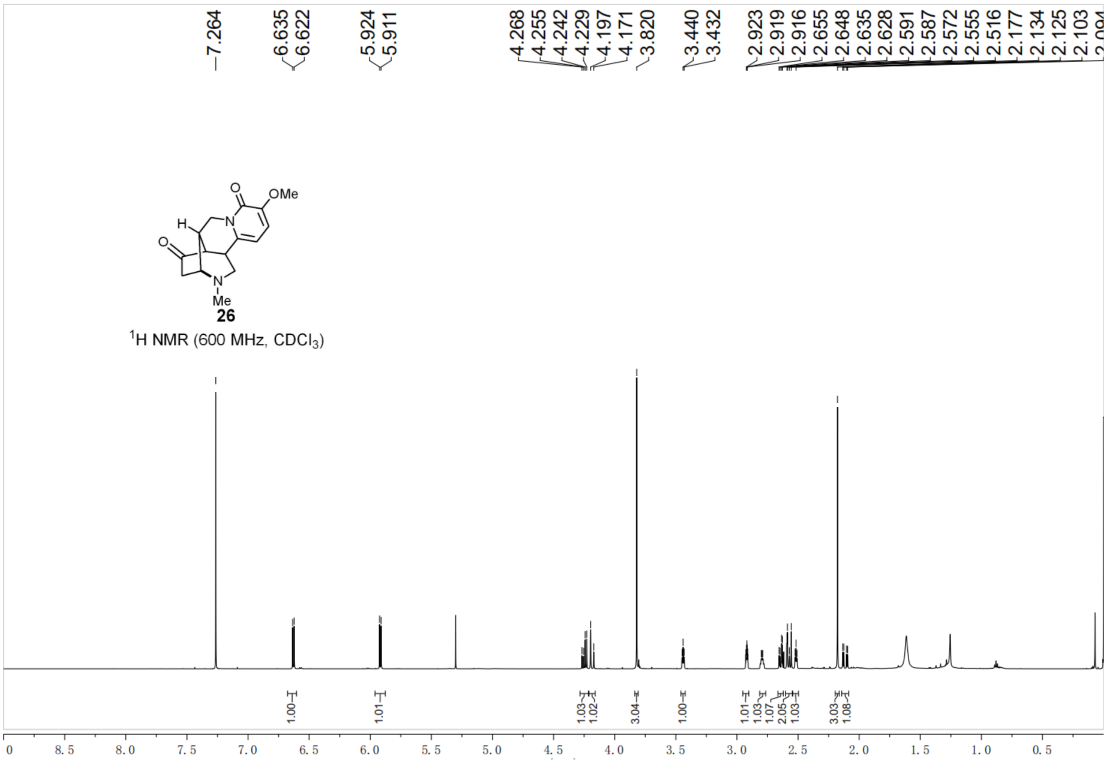
7.264
6.635
6.622
5.924
5.911
4.268
4.255
4.242
4.229
4.197
4.171
3.820
3.440
3.432
2.923
2.919
2.916
2.655
2.648
2.635
2.628
2.591
2.587
2.572
2.555
2.516
2.177
2.134
2.125
2.103
OMe
H
O
N
Me
26
1H NMR (600 MHz, CDCl3)
1.00
1.01
1.03
1.02
3.04
1.00
1.01
1.03
1.07
2.05
1.03
3.03
1.08
8.5
8.0
7.5
7.0
6.5
6.0
5.5
5.0
4.5
4.0
3.5
3.0
2.5
2.0
1.5
1.0
0.5

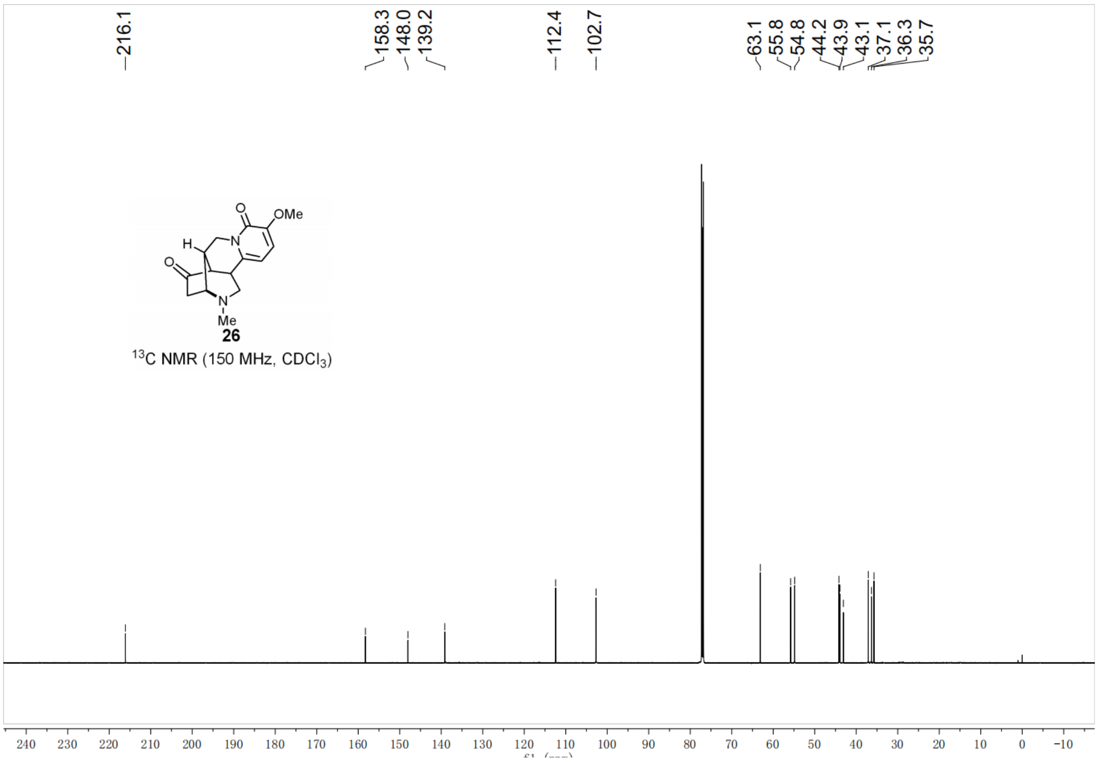
216.1
158.3
148.0
139.2
112.4
102.7
63.1
55.8
54.8
44.2
43.9
43.1
37.1
36.3
35.7
OMe
H
O
N
Me
26
13C NMR (150 MHz, CDCl3)
240
230
220
210
200
190
180
170
160
150
140
130
120
110
100
90
80
70
60
50
40
30
20
10
0
-10

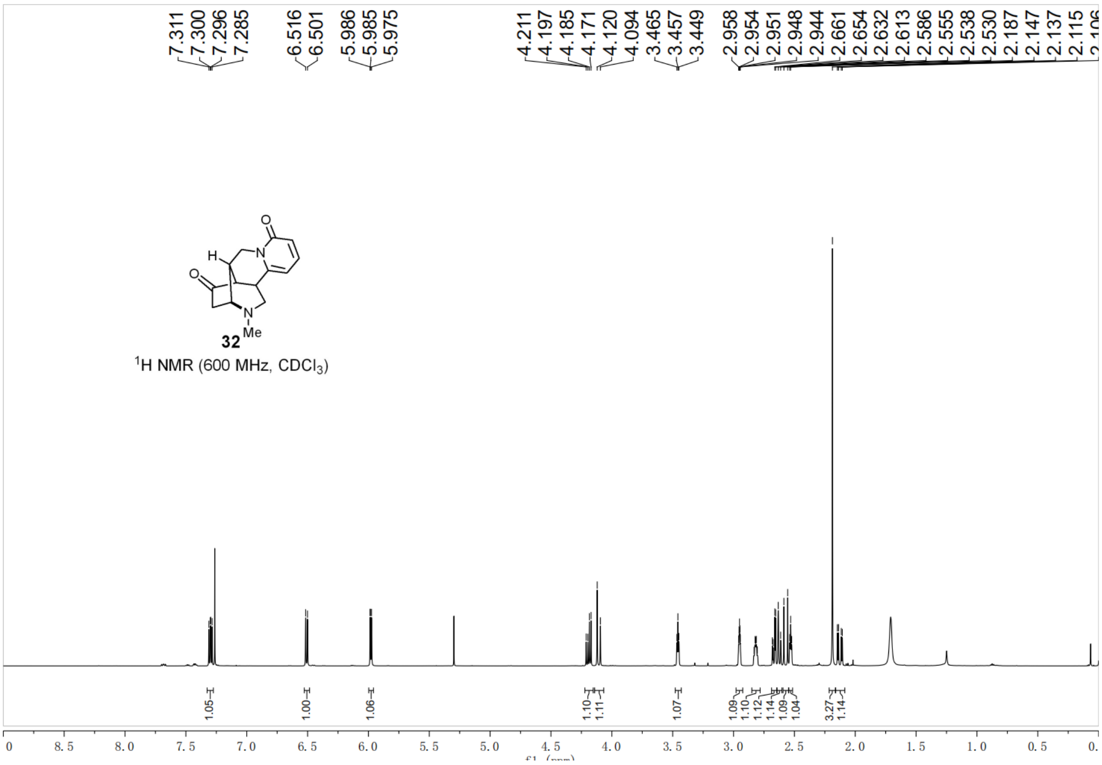
7.311
7.300
7.296
7.285
6.516
6.501
5.986
5.985
5.975
4.211
4.197
4.185
4.171
4.120
4.094
3.465
3.457
3.449
2.958
2.954
2.951
2.948
2.944
2.661
2.654
2.632
2.613
2.586
2.555
2.538
2.530
2.187
2.147
2.137
2.115
H
O
N
O
N
Me
32
¹H NMR (600 MHz, CDCl₃)

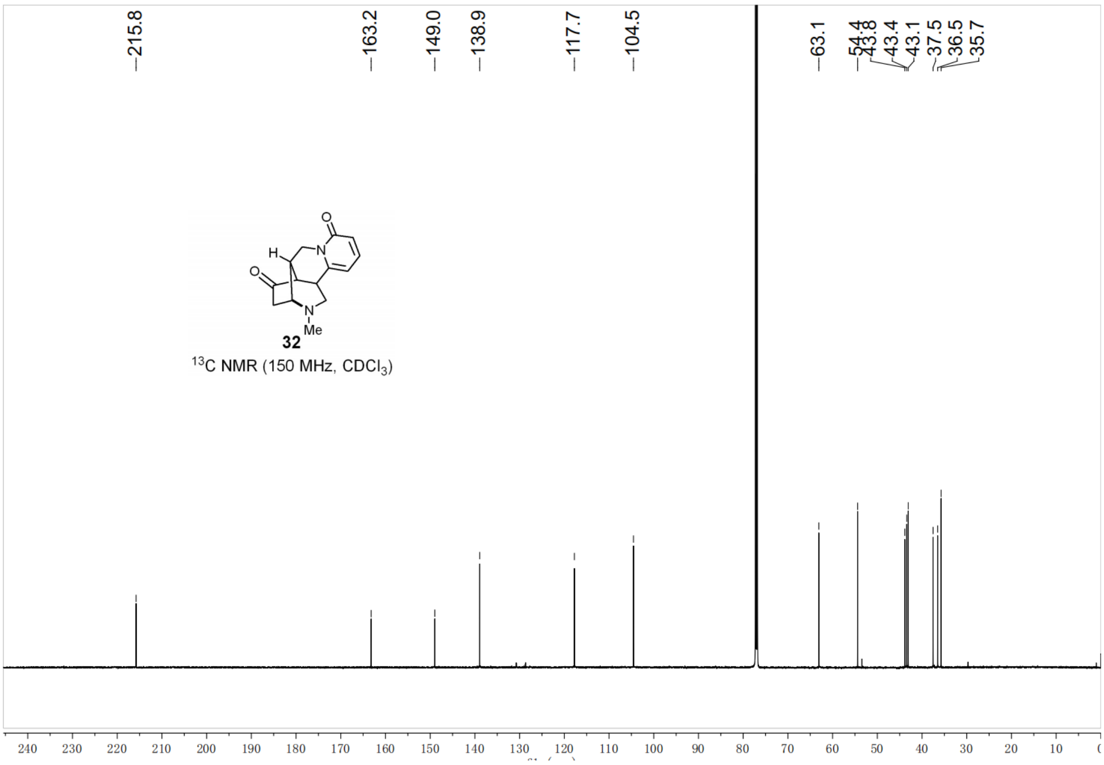
215.8
163.2
149.0
138.9
117.7
104.5
63.1
54.4
43.8
43.4
43.1
37.5
36.5
35.7
H
O
N
O
N
Me
32
¹³C NMR (150 MHz, CDCl₃)

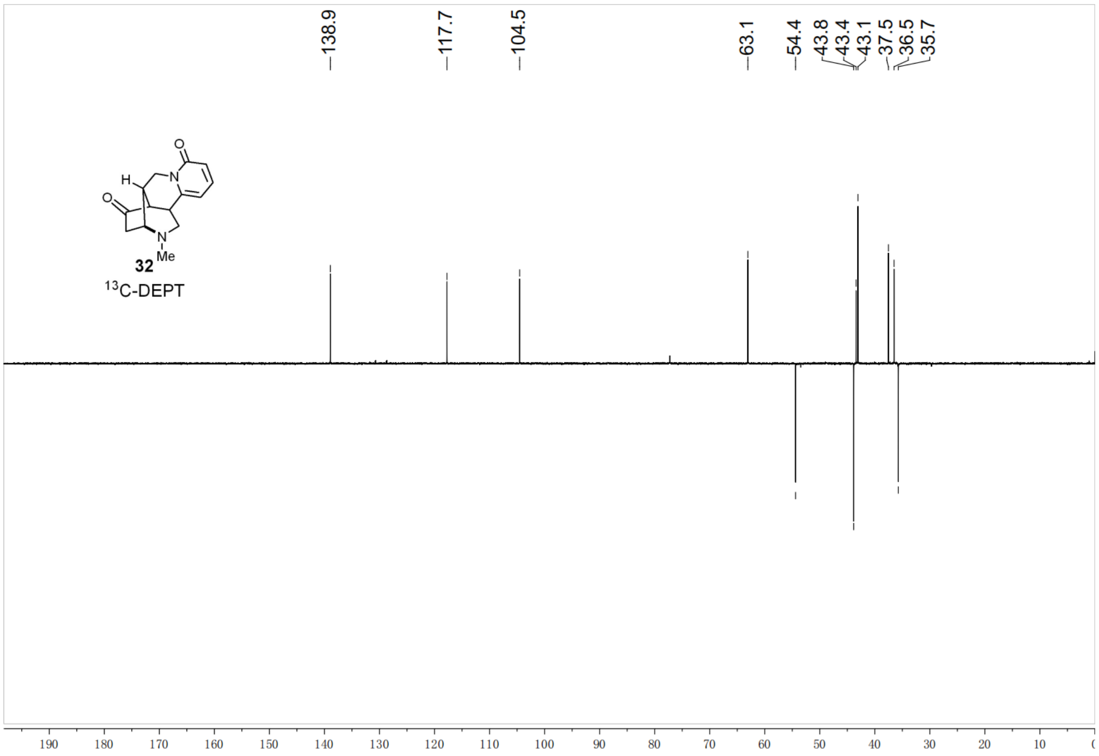
138.9
117.7
104.5
63.1
54.4
43.8
43.4
43.1
37.5
36.5
35.7
32
13C-DEPT
190 180 170 160 150 140 130 120 110 100 90 80 70 60 50 40 30 20 10

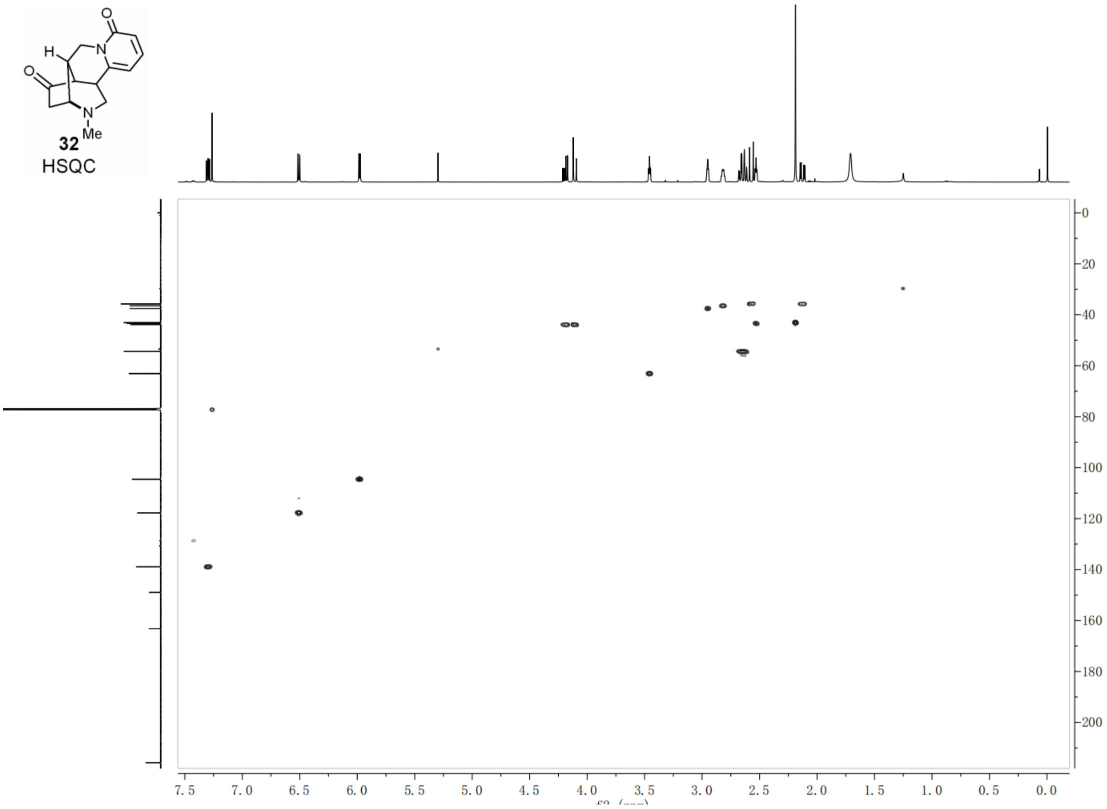
32
HSQC
0 20 40 60 80 100 120 140 160 180 200
7.5 7.0 6.5 6.0 5.5 5.0 4.5 4.0 3.5 3.0 2.5 2.0 1.5 1.0 0.5 0.0

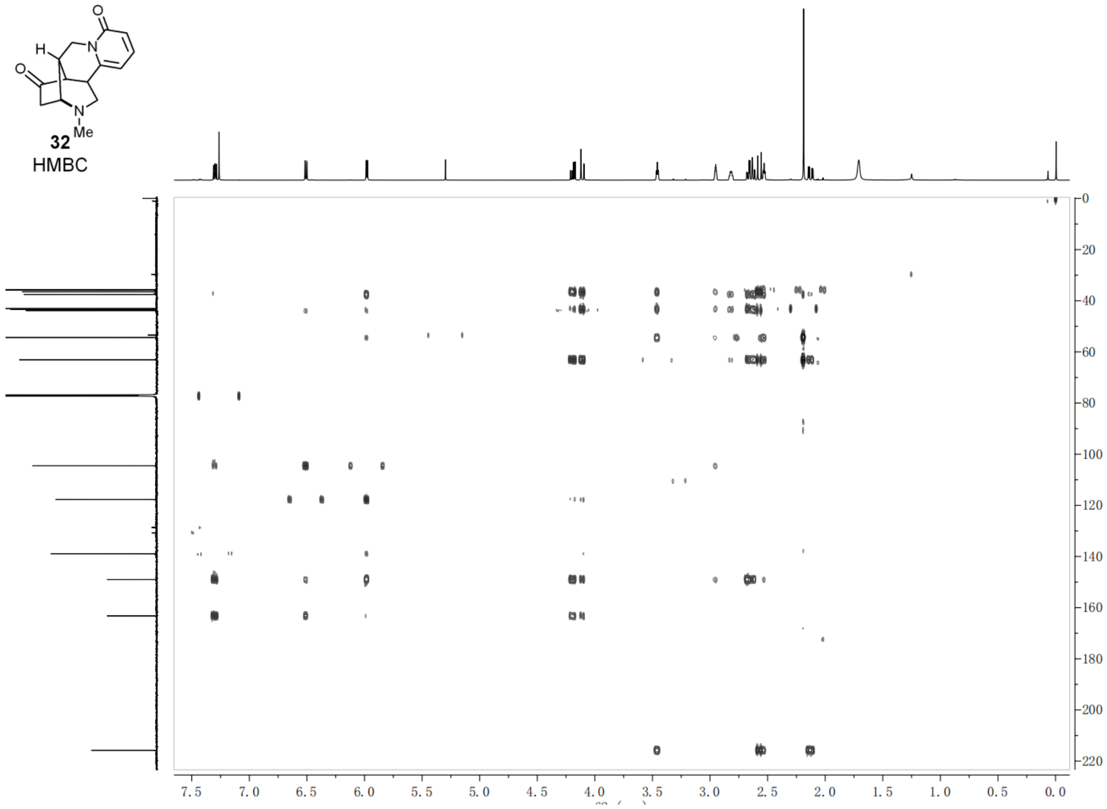
32
HMBC

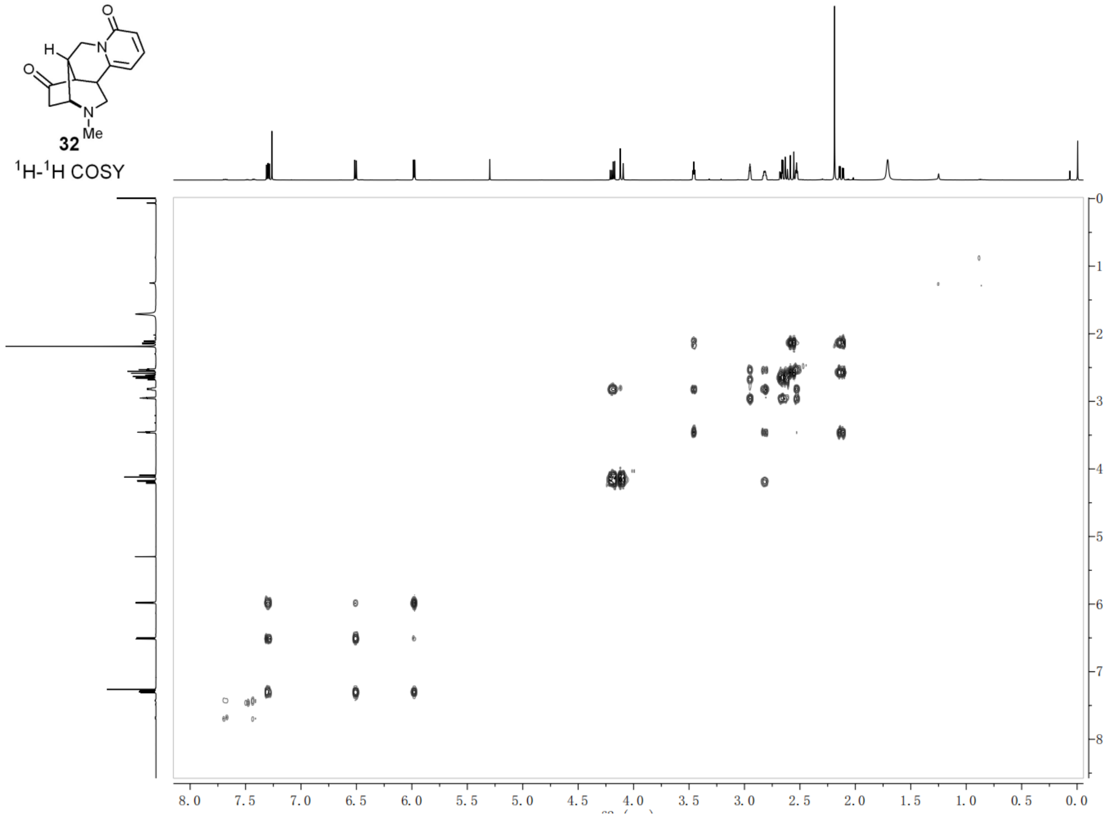
32
^{1}H-^{1}H COSY

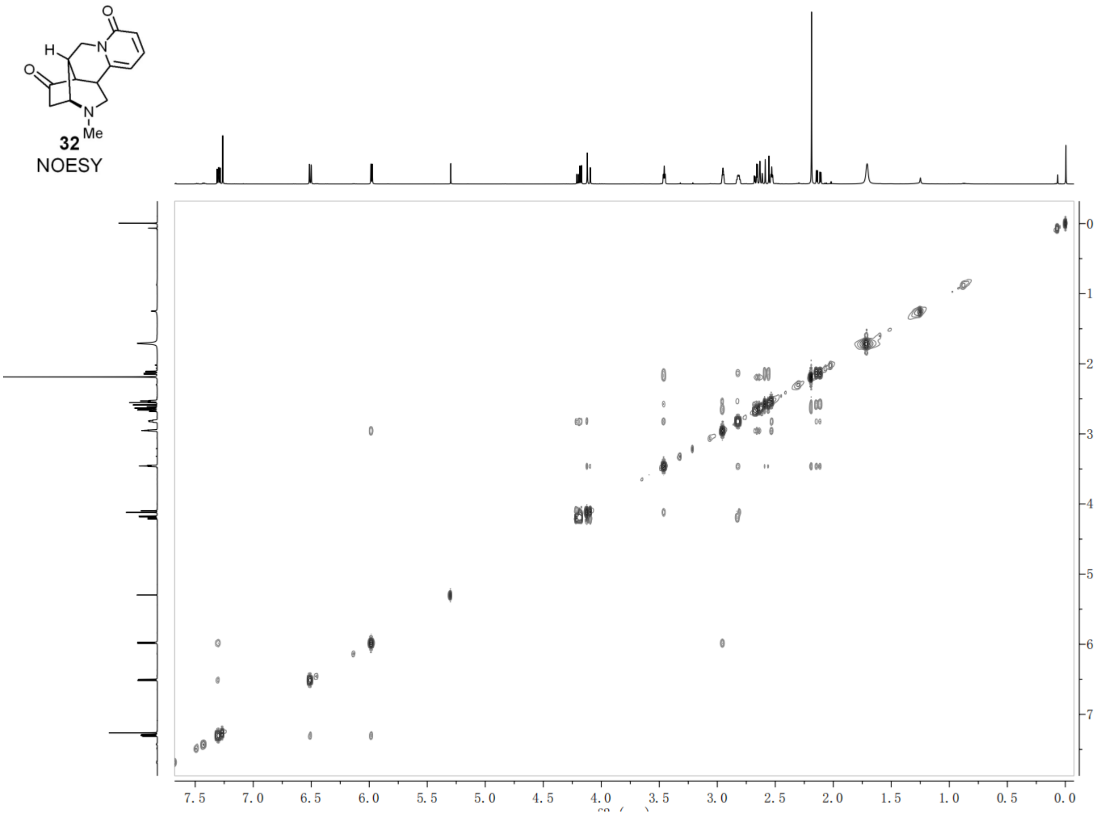
O
H
O
N
N
Me
32
NOESY
7.5
7.0
6.5
6.0
5.5
5.0
4.5
4.0
3.5
3.0
2.5
2.0
1.5
1.0
0.5
0.0
0
1
2
3
4
5
6
7

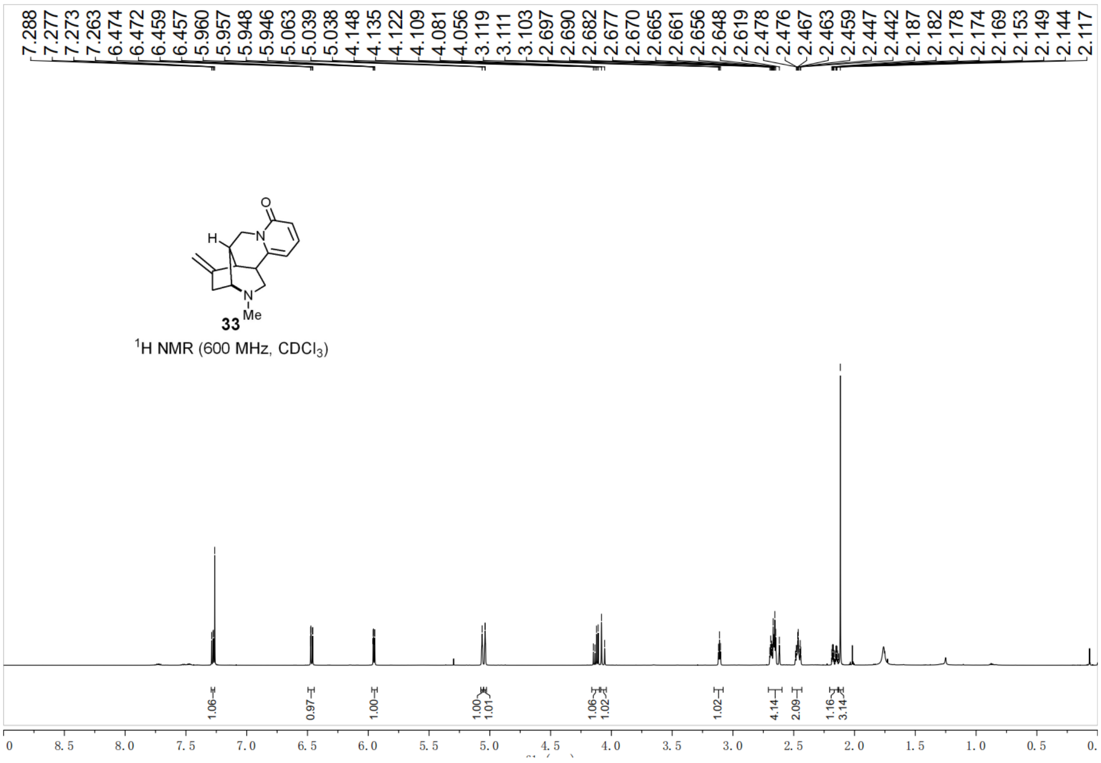
7.288
7.277
7.273
7.263
6.474
6.472
6.459
6.457
5.960
5.957
5.948
5.946
5.063
5.039
5.038
4.148
4.135
4.122
4.109
4.081
4.056
3.119
3.111
3.103
2.697
2.690
2.682
2.677
2.670
2.665
2.661
2.656
2.648
2.619
2.478
2.476
2.467
2.463
2.459
2.447
2.442
2.187
2.182
2.178
2.174
2.169
2.153
2.149
2.144
2.117
H
O
N
N
Me
33
^{1}H NMR (600 MHz, CDCl$_3$)
1.06
0.97
1.00
1.00
1.01
1.06
1.02
1.02
4.14
2.09
1.16
3.14

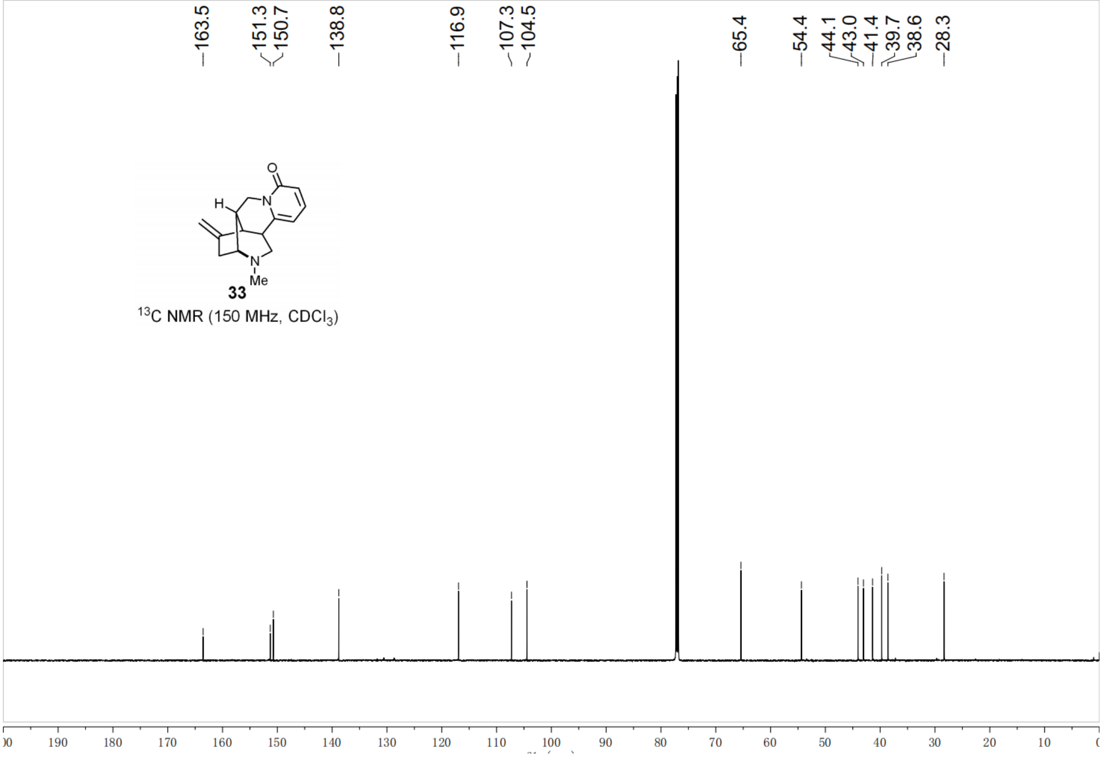
163.5
151.3
150.7
138.8
116.9
107.3
104.5
65.4
54.4
44.1
43.0
41.4
39.7
38.6
28.3
H
O
N
N
Me
33
^{13}C NMR (150 MHz, CDCl$_3$)

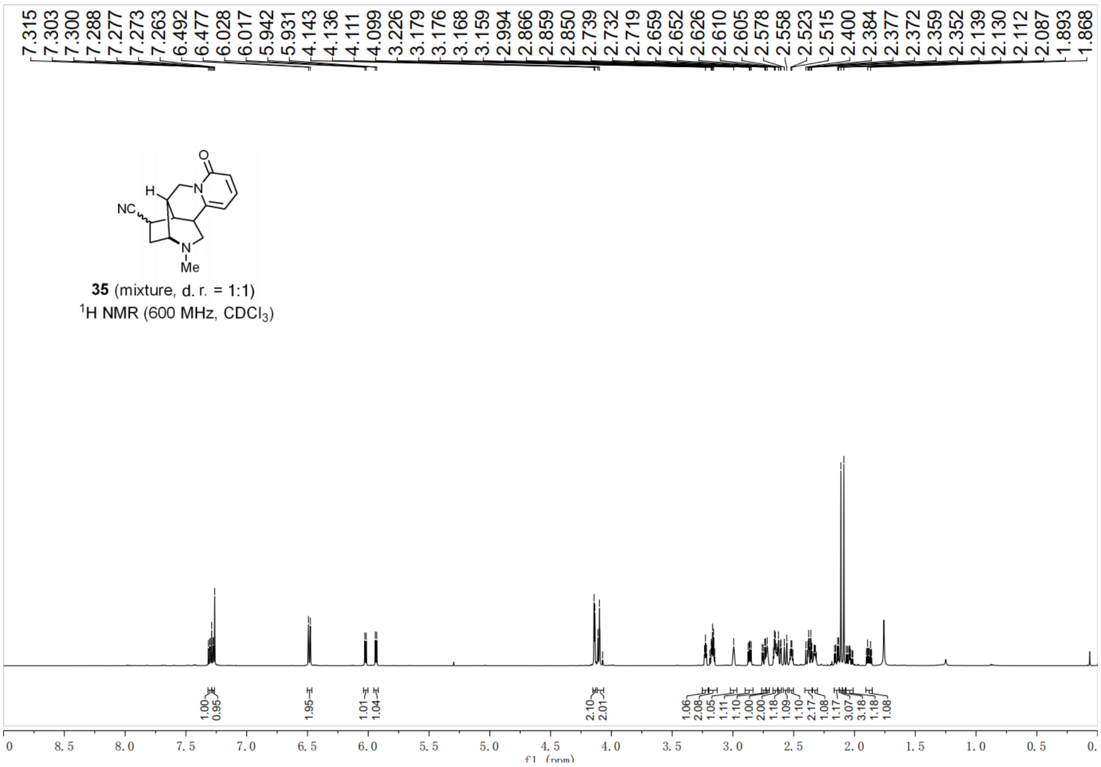
35 (mixture, d. r. = 1:1)
1H NMR (600 MHz, CDCl3)
f1 (ppm)

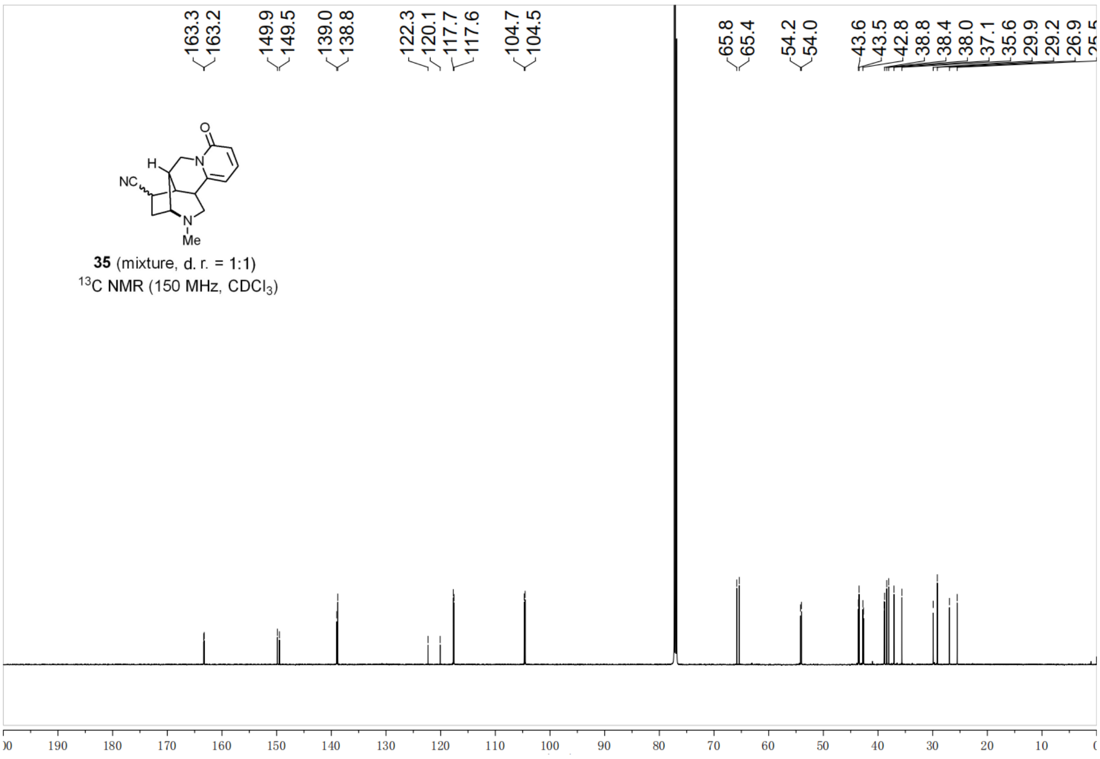
35 (mixture, d. r. = 1:1)
13C NMR (150 MHz, CDCl3)

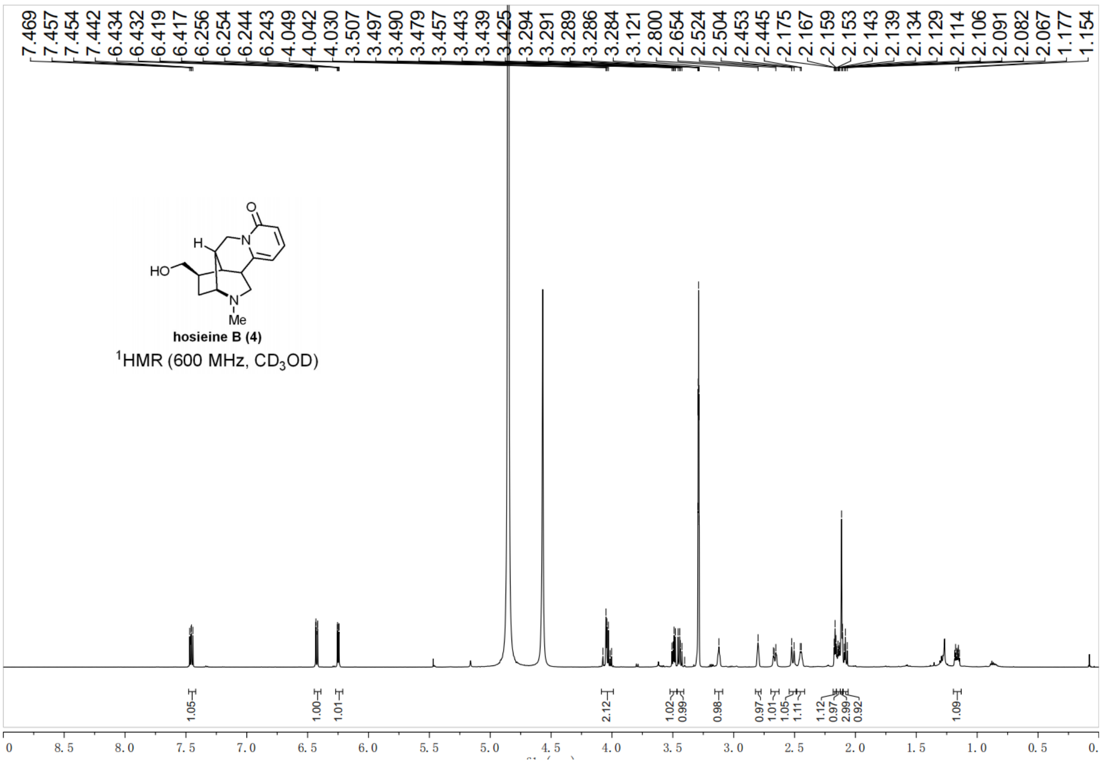
hosieine B (4)
1HMR (600 MHz, CD3OD)

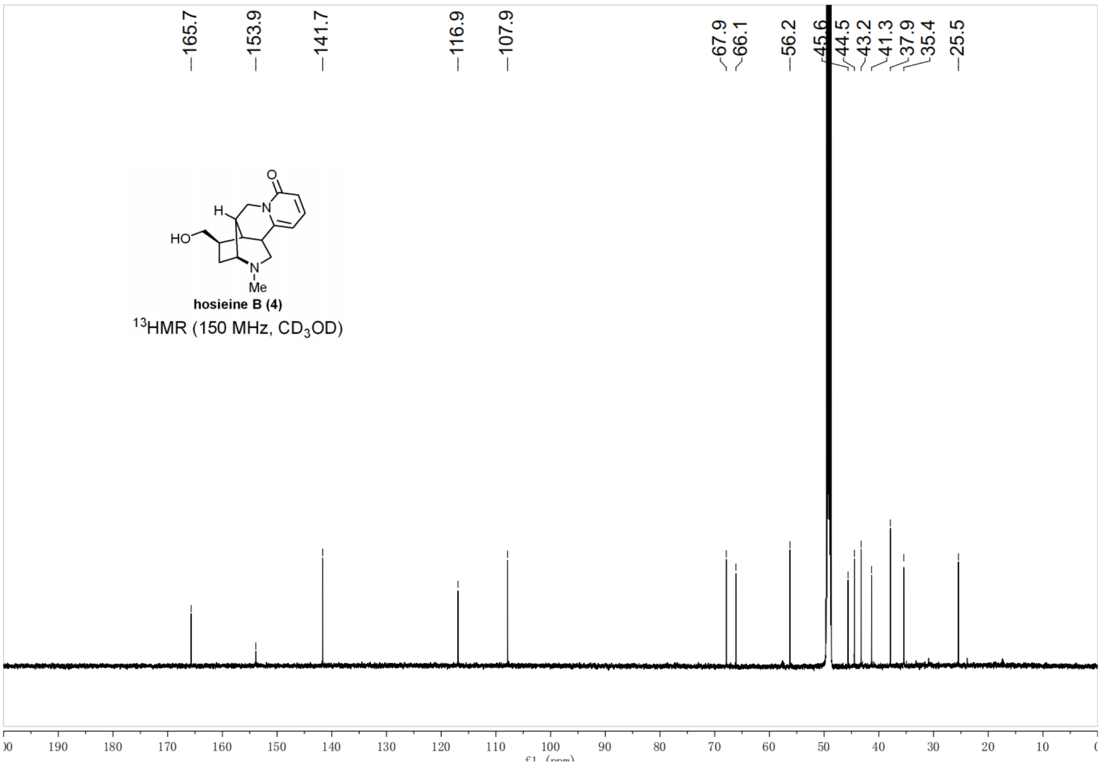
hosieine B (4)
13HMR (150 MHz, CD3OD)

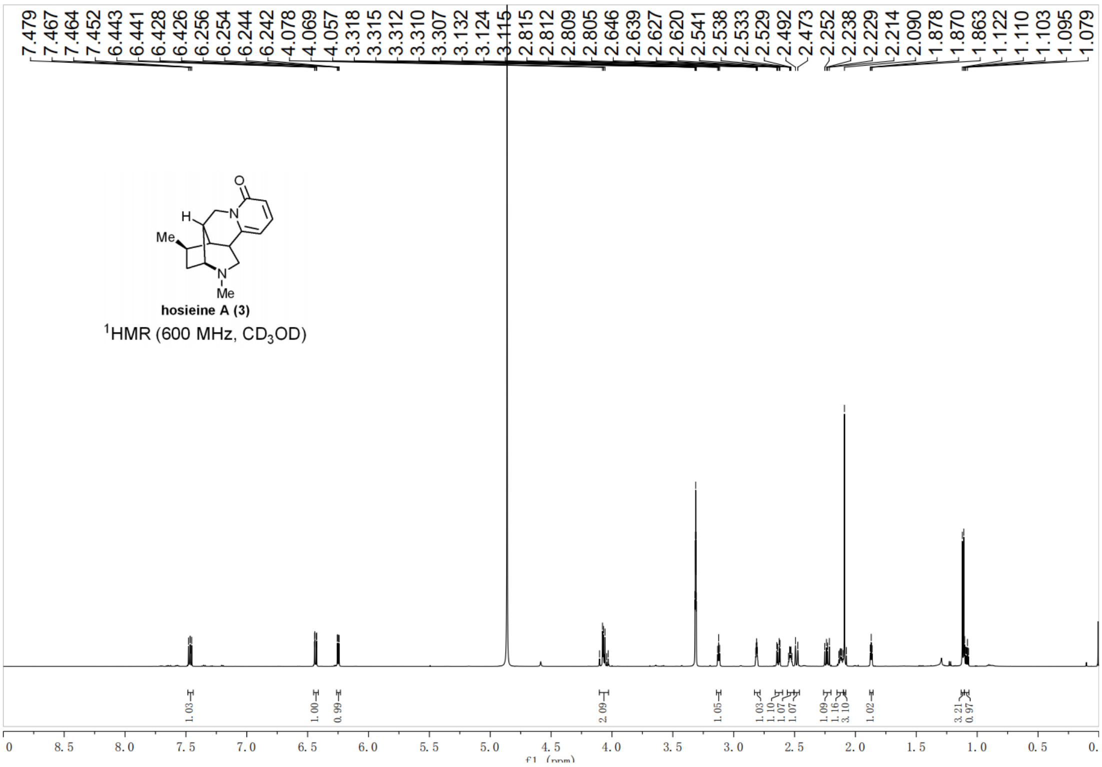
7.479 7.467 7.464 7.452 6.443 6.441 6.428 6.426 6.256 6.254 6.244 6.242 4.078 4.069 4.057 3.318 3.315 3.312 3.310 3.307 3.132 3.124 3.115 2.815 2.812 2.809 2.805 2.646 2.639 2.627 2.620 2.541 2.538 2.533 2.529 2.492 2.473 2.252 2.238 2.229 2.214 2.090 1.878 1.870 1.863 1.122 1.110 1.103 1.095 1.079
hosieine A (3)
1HMR (600 MHz, CD3OD)
1.03 1.00 0.99 2.09 1.05 1.03 1.10 1.07 1.07 1.09 1.16 3.10 1.02 3.21 0.97
f1 (ppm)

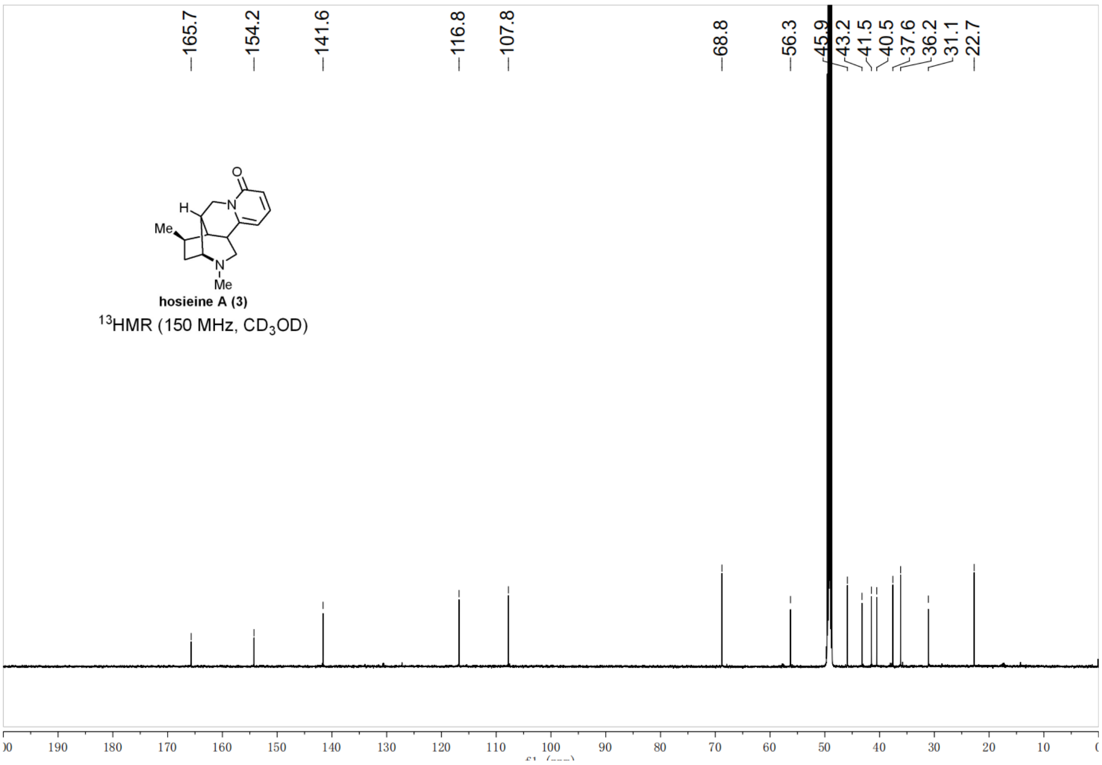
165.7 154.2 141.6 116.8 107.8 68.8 56.3 45.9 43.2 41.5 40.5 37.6 36.2 31.1 22.7
hosieine A (3)
13HMR (150 MHz, CD3OD)
f1 (ppm)

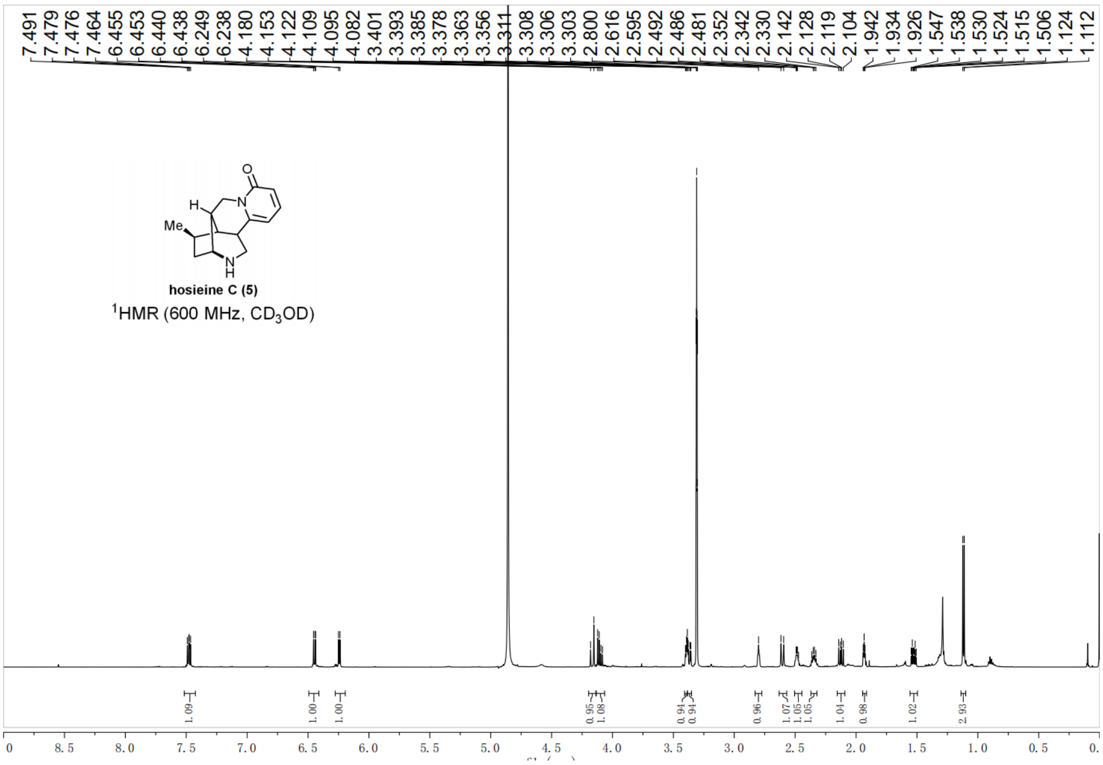
7.491 7.479 7.476 7.464 6.455 6.453 6.440 6.438 6.249 6.238 4.180 4.153 4.122 4.109 4.095 4.082 3.401 3.393 3.385 3.378 3.363 3.356 3.311 3.308 3.306 3.303 2.800 2.616 2.595 2.492 2.486 2.481 2.352 2.342 2.330 2.142 2.128 2.119 2.104 1.942 1.934 1.926 1.547 1.538 1.530 1.524 1.515 1.506 1.124 1.112
H
Me
O
N
H
hosieine C (5)
1HMR (600 MHz, CD3OD)
1.09 1.00 1.00 0.95 1.08 0.94 0.94 0.96 1.07 1.05 1.05 1.04 0.98 1.02 2.93
8.5 8.0 7.5 7.0 6.5 6.0 5.5 5.0 4.5 4.0 3.5 3.0 2.5 2.0 1.5 1.0 0.5

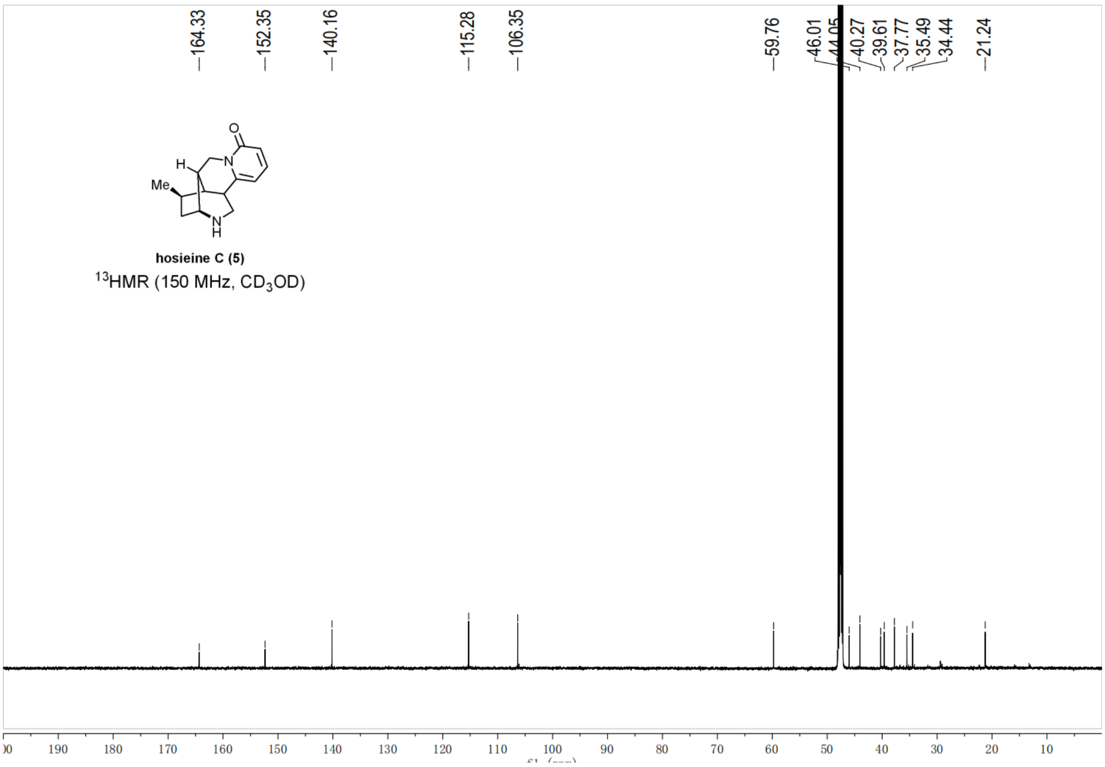
164.33 152.35 140.16 115.28 106.35 59.76 46.01 44.05 40.27 39.61 37.77 35.49 34.44 21.24
H
Me
O
N
H
hosieine C (5)
13HMR (150 MHz, CD3OD)
190 180 170 160 150 140 130 120 110 100 90 80 70 60 50 40 30 20 10
